# On the Importance of Balancing the p*K*_a_ of the Additive in β‐Ketoenamine COF Synthesis

**DOI:** 10.1002/chem.202501512

**Published:** 2025-06-18

**Authors:** Thijmen A. van Voorthuizen, Monique A. van der Veen, Louis C. P. M. de Smet, Maarten M. J. Smulders

**Affiliations:** ^1^ Laboratory of Organic Chemistry Wageningen University & Research Wageningen 6708 WE The Netherlands; ^2^ Department of Chemical Engineering Delft University of Technology Delft 2629 HZ The Netherlands

**Keywords:** covalent organic framework, DIPEA, keto‐enol tautomerism, organobase, β‐ketoenamine

## Abstract

Due to the irreversible tautomerization of imine linkages to their corresponding ketoenamines, β‐ketoenamine‐linked Covalent Organic Frameworks (COFs) are a stable type of COF that displays high surface areas. In the solvothermal synthesis of such COFs, the use of (acetic) acid is ubiquitous. However, the effect of the added acid on the COF properties (notably their surface area) has never been investigated. Building on an extensive literature overview, we systematically studied the effect of the p*K*_a_ of several added acids on COF performance characteristics and extended the investigation by including a series of (organo‐)bases with varying p*K*_a_. Interestingly, the highest BET surface areas, above 1400 m^2^/g, were found in the alkaline region of the p*K*_a_ window, with a maximum near p*K*_a_ ∼10.8 for triethylamine (TEA) and *N*,*N*‐diisopropylethylamine (DIPEA). Considering the p*K*_a_ values related to the three phenolic hydroxyl groups of 2,4,6‐triformylphloroglucinol, one of the COF building blocks, these organobases fully deprotonate two of these hydroxyl groups and partly deprotonate the third one, which optimizes the reaction rate of the β‐ketoenamine bond formation, explaining the improved COF crystallinity and associated microporosity. The largely overlooked use of organobases in the synthesis of β‐ketoenamine‐linked COFs thus offers a promising approach to improve the COF performance.

## Graphical abstract

β‐ketoenamine COFs are usually synthesized in the presence of acetic acid. Here, we report the surprising role of organobases TEA and DIPEA in achieving **TpPa** with high Brunauer‐Emmett‐Teller surface areas (>1400 m^2^/g) and high crystallinity. The similar p*K*_a_ values of TEA and DIPEA (10.8 and 11.0, resp.) and **Tp** (10.8) mark the importance of balancing the p*K*_a_ for high‐quality β‐ketoenamine COFs.

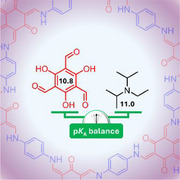

## 1 Introduction

Covalent Organic Frameworks (COFs) are porous nanomaterials that have found extensive applications, including in energy storage,^[^ ^1^ ^]^ catalysis,^[^ ^2^ ^]^ tribology,^[^ ^3^ ^]^ gas storage and separation, and sensing.^[^ ^4^, ^5^, ^6^ ^]^ Their wide applicability stems from a few key properties: their permanent porosity, crystallinity, and stability, as well as their tunability in terms of chemical linkages and functional groups. Over the past two decades, a large variety of COF linkages has been developed. Starting with the original boroxine linkage in 2005,^[^ ^7^ ^]^ over two dozen different linkages have been reported, with varying functionality, stability, and synthetic route.^[^ ^8^ ^]^ Generally, COFs display high thermal stability^[^ ^7^ ^]^ and most COFs are hydrolytically stable as well.^[^ ^9^ ^]^ Finally, the high crystallinity of COFs is the result of the use of so‐called dynamic covalent chemistry in their synthesis.^[^ ^10^, ^11^ ^]^ To synthesize a crystalline COF, the reversibility of the underlying bond‐forming reaction is essential, as it enables healing any defects that are formed initially. Examples of dynamic covalent bonds used in COF synthesis ^[^ ^11^ ^]^ include Diels‐Alder reactions^[^ ^12^ ^]^ and the formation of boronate esters,^[^ ^7^ ^]^ imine bonds,^[^ ^13^ ^]^ and vinylene bonds^[^ ^14^ ^]^ through condensation reactions. As these reactions are typically performed under conditions where the linkage formation is reversible, the thermodynamic, crystalline product is formed.

The use of dynamic covalent chemistry in COF synthesis demands a delicate balance between the reversibility of the linkage formation and the stability of the product. If ambient conditions are too close to the reversible reaction conditions, the stability may be compromised. Boronate esters, for example, form through a highly reversible reaction, but have relatively low hydrolytic stability.^[^ ^15^, ^16^ ^]^ For imine bonds, the issue of hydrolysis is less prominent as the hydrolysis reaction is acid‐catalyzed. To improve the hydrolytic stability of imines under acidic conditions, functional groups on the building blocks can be introduced. For example, judiciously placed alcohol or methoxy moieties on the monomers can stabilize the COF backbone through intramolecular hydrogen bonds,^[^ ^17^ ^]^ interlayer hydrogen bonds^[^ ^18^ ^]^ or reducing the interlayer charge repulsion.^[^ ^19^ ^]^ A methyl‐substituted aldehyde block can also further stabilize imine‐linked COFs.^[^ ^20^ ^]^ Another route is to post‐synthetically convert the imine linkage to another, more stable linkage. Following this approach, the imine linkage has been changed into an amide,^[^ ^21^ ^]^ amine,^[^ ^22^ ^]^ quinoline,^[^ ^23^ ^]^ thiazole,^[^ ^24^ ^]^ or oxazole.^[^ ^25^ ^]^ Such conversions are irreversible, making it a very attractive method to increase the stability of imine COFs after their synthesis.

**Scheme 1:**
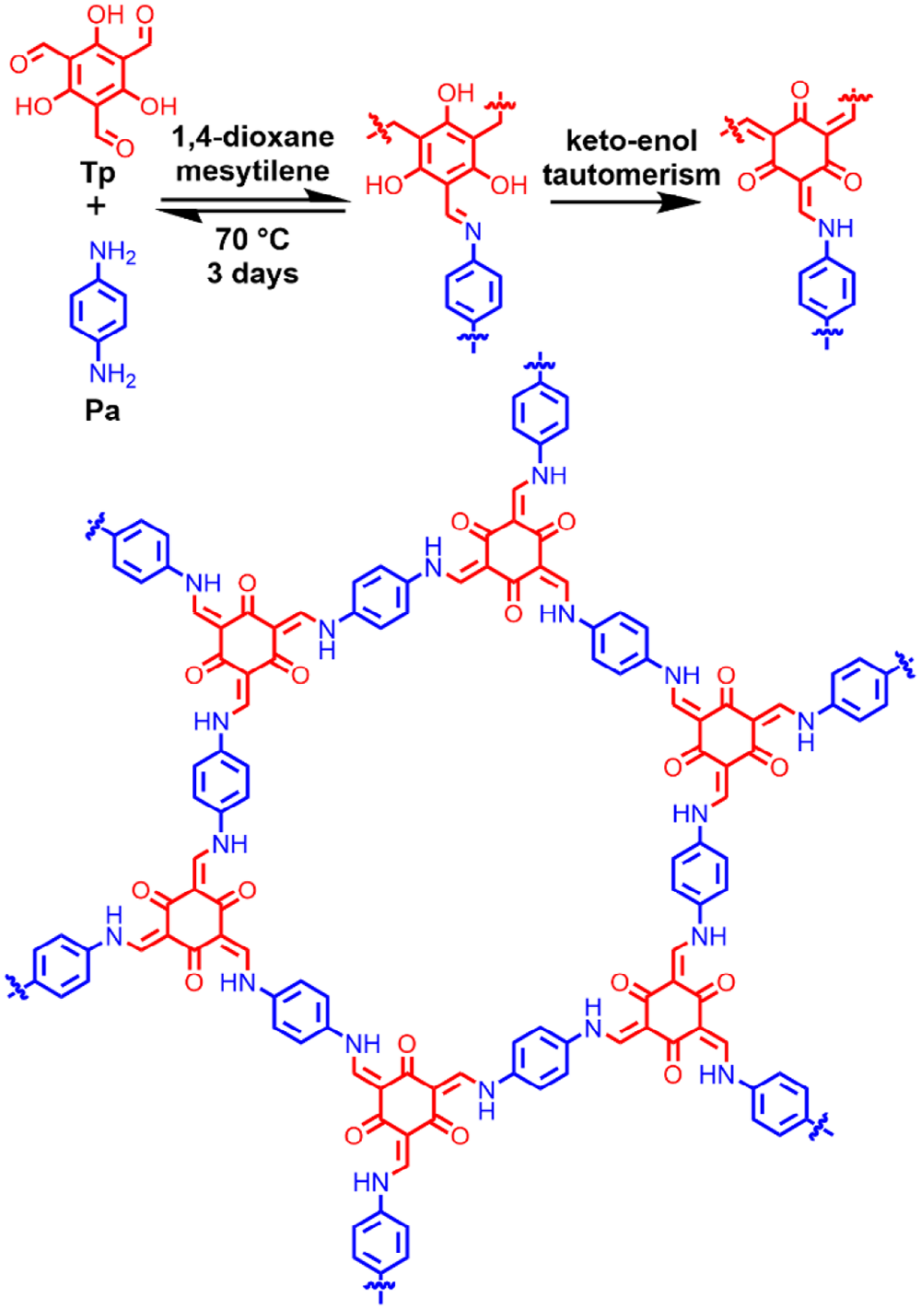
Synthesis of **TpPa** from 2,4,6‐triformylphloroglucinol (**Tp**) and 1,4‐phenylenediamine (**Pa**) in two steps: the formation of an imine linkage and the subsequent tautomerization into a β‐ketoenamine linkage. The top shows the two‐step reaction for a single linkage, while the bottom structure shows the final recurring macrocyclic COF structure that is formed.

However, for such post‐synthetic methods, reagents need access to the linkages, which is hampered by the microporous structure, potentially resulting in spatial heterogeneity of the linker conversion.^[^ ^26^ ^]^ Direct, spontaneous conversion of the imine linkage into a more stable one, without the need of additional reagents, is therefore preferable. An example of this is the β‐ketoenamine linkage, which forms from an imine linkage in the presence of a phenol on the *ortho* position relative to the aldehyde functionality through an irreversible tautomerization step (Scheme 1).^[^ ^27^ ^]^ β‐ketoenamine‐linked COFs have displayed excellent stability in boiling water, as well as strong acids and bases over multiple days.^[^ ^27^ ^]^

The β‐ketoenamine network is thus formed through covalent reversible assembly followed by a spontaneous irreversible locking step.^[^ ^28^ ^]^ However, defects that are normally corrected can now be incorporated into the framework as the irreversible locking can be faster than the error‐correction step. This leads to a lower crystallinity and surface area.^[^ ^28^ ^]^ One strategy to increase the surface area is to separate the reversible network formation and irreversible locking steps: first a defect‐free COF is formed, which is then transformed into the stable β‐ketoenamine‐linked COF. This was achieved by Daugherty et al., who first synthesized an imine‐linked COF and then used a post‐synthetic linker exchange to replace the aldehyde linker with another aldehyde linker with the hydroxyl groups needed for the tautomerization, all in the presence of acetic acid.^[^ ^29^ ^]^ In another strategy, a two‐step, one‐pot reaction was achieved by first synthesizing a urea COF, which was transformed into a β‐ketoenamine‐linked COF by heating. In this manner, **TpPa** (made from 2,4,6‐triformylphloroglucinol (**Tp**, also abbreviated as **TFP**) and 1,4‐phenylenediamine (**Pa**)) was synthesized with the highest reported surface area of 1712 m^2^/g for this specific COF.^[^ ^30^ ^]^

Mechanochemistry can also improve the crystallinity and processability of COFs,^[^ ^31^ ^]^ while further solvothermal strategies that enhance the surface area of **TpPa** COFs can involve protecting groups on the amine building blocks for rate control,^[^ ^32^ ^]^ or the use of pyrrolidine in organic solvents or KOH in water:dimethylformamide mixtures.^[^ ^26^, ^33^, ^34^, ^35^, ^36^, ^37^, ^38^, ^39^ ^]^ Pyrrolidine and pyrrolidine derivatives were found to have a positive effect on **TpPa** synthesis through the formation of an iminium intermediate with the aldehyde component.^[^ ^26^ ^]^ KOH was found to increase the solubility of **Tp** in water, which was needed to achieve high‐quality COFs.^[^ ^39^ ^]^

While there are several strategies to optimize the COF surface area, it can be generally observed that the synthesis of **TpPa** is performed in the presence of acetic acid. For imine‐linked COFs, the role of acetic acid is known and its use common, although their BET surface area can also be further increased by protecting groups,^[^ ^40^ ^]^ low surface tension solvents during solvent exchange^[^ ^41^ ^]^ or even swapping acetic acid for metal triflates.^[^ ^42^ ^]^ In contrast, the beneficial role of an acidic additive in β‐ketoenamine‐linked COFs is presumed, rather than extensively verified experimentally. What is more, the role of organobases has received hardly the same attention compared to acidic analogues for β‐ketoenamine‐linked COFs.

To obtain further insight in previous efforts regarding the use of acids and bases for their use in the synthesis of **TpPa**, which is the first discovered^[^ ^27^ ^]^ and a widely applied β‐ketoenamine‐linked COF, we analyzed the existing literature (Table S1). Out of the 252 reported syntheses (from 226 research papers), 79% are solvothermal or similar, while 12% involve mechanochemical synthesis, and 9% involve a different synthesis route, or the synthesis is not specified. In mechanochemistry, the most common additive is *p*‐toluenesulfonic acid (PTSA, 61%), where it is actually mainly employed as a coordinating agent to **Pa** rather than as an acid to achieve high surface areas.^[^ ^43^ ^]^ In solvothermal synthesis, it is most common to use an acid (86% and when an acid is employed, the acid in question is acetic acid in 98% of the cases, see Table S1). The wide use of acetic acid can likely be explained by its known catalytic effect in the formation of imine COFs.^[^ ^44^ ^]^ However, in the case of β‐ketoenamines, acids do not only promote the imine bond formation, they also accelerate the locking of the carbon‐nitrogen bond via an irreversible tautomerization. The importance of the interplay between the error‐correcting formation and breaking of the imine linkage with the irreversible locking, and its effect on COF properties underscores the need for an in‐depth investigation of the effects of the p*K*_a_ value of the additive on the surface area of **TpPa** COFs.

Therefore, given the high applicability of β‐ketoenamine‐linked COFs, it is essential to understand the effect of the p*K*_a_ of the additive on the COF synthesis, in order to obtain materials with high crystallinity and surface area. To this end, we performed a systematic study of the effect of not only organic acids, but also of bases on the surface area of β‐ketoenamine‐linked COFs, using **TpPa**, as a model COF.

## 2 Results and Discussion

To investigate the effect of the p*K*_a_ of the additive on the Brunauer‐Emmett‐Teller (BET) surface area of the COF, **TpPa** was synthesized, building on previous work by our group on imine COFs.^[^ ^20^ ^]^ The choice and amount of acid or base catalyst was varied. For the acids, the following were selected: acetic acid (p*K*_a_ 4.8), propionic acid (p*K*_a_ 4.9), formic acid (p*K*_a_ 3.7), and chloroacetic acid (p*K*_a_ 2.9). They were selected because they are small, primary carboxylic acids, similar to acetic acid, and vary significantly in their acidity. For the bases, the following were selected: pyridine (conjugate acid p*K*_a_ 5.2), 2,4,6‐trimethylpyridine (TMP, conjugate acid p*K*_a_ 7.4), triethylamine (TEA, conjugate acid p*K*_a_ 10.8), 1,8‐diazabicyclo[5.4.0]undec‐7‐ene (DBU, conjugate acid p*K*_a_ 13.5), and KOH (conjugate acid p*K*_a_ 14.7). They were selected because they vary greatly in their alkalinity, and contain a tertiary amine group (with the exception of KOH). By selecting these organobases, we prevent the formation of an iminium intermediate that was previously reported for pyrrolidine and pyrrolidine derivatives,^[^ ^26^ ^]^ which enabled us to elucidate the effect of the p*K*_a_. KOH was chosen because its use has been reported before^[^ ^39^ ^]^ (although mostly to enhance the solubility of the building blocks) and because it is a strong base.

Synthesis of the COFs started by separately dissolving triformylphloroglucinol (**Tp**) and 1,4‐diphenylenediamine (**Pa**) in 1,4‐dioxane:mesitylene 4:1 v/v and adding water and an organic acid or base to the **Pa** solution. The two solutions were then combined and stirred at 70 °C for three days, resulting in the formation of a suspension of solid, red COF particles. By stirring, the occurrence of a biphasic system (observed for samples without or with a low concentration of an acid or base) was suppressed. The COF solids were then washed with DMF (twice), ethanol, and acetone, and dried overnight in an oven at 120 °C. For a more detailed procedure, see the Supporting Information.

**Figure 1:**
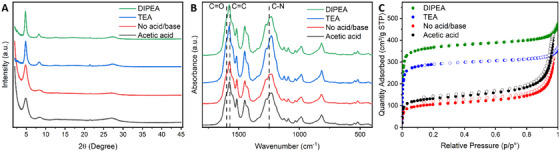
The A) PXRD patterns, B) ATR‐FTIR spectra, and C) N_2_ adsorption isotherms of selected COFs (**TpPa**, synthesized with 2 equivalents of acetic acid, TEA, or DIPEA, or without acid or base). The data on TEA, without acid/base and acetic acid are discussed in the main text near this figure, while those of DIPEA are discussed in the final paragraph of the Results and Discussion section. The spectra and N_2_ adsorption isotherms of all other COFs prepared in this study are given in the Supporting Information.

The crystallinity of the powders was studied by powder X‐ray diffraction (PXRD). The spectrum of **TpPa** synthesized with TEA shows peaks at 2*θ* = 4.5° and 7.9°, corresponding to the (1 0 0) and (1 1 0) planes, respectively (Figure 1A, and Figures S73‐S81). The unit cell dimension *a* was found to be 2.26 nm. The peak at 2*θ* = 27° is from the (0 0 1) plane, and corresponds to an inter‐sheet distance of 3.30 Å. This confirms the hexagonal lattice of the synthesized COFs and is in accordance with literature.^[^ ^27^ ^]^ In contrast, **TpPa** synthesized with formic or acetic acid or without additives shows lower crystallinity and broader peaks, while **TpPa** synthesized with KOH shows no crystallinity at all. This may point to the presence of an optimal p*K*_a_ value of the additive.

The ATR‐FTIR spectra of the various samples confirm total consumption of the starting material and β‐ketoenamine bond formation (Figure 1B and Figure S1‐S72). In more detail, for all samples, the typical aldehyde C═O stretch vibration at 1690 cm^−1^ of **Tp** has disappeared. Instead, the spectra show the characteristic vibrations of the C═C stretch, C═O stretch and C─N stretch in the keto‐form around 1578 cm^−1^, a shoulder at 1600 cm^−1^ and a peak at 1250 cm^−1^, respectively. This is in accordance with literature.^[^ ^27^ ^]^ No significant changes were observed between samples with different concentrations or types of additives (see Supporting Information for all spectra).

The morphology of selected samples (namely, **TpPa** synthesized with acetic acid and TEA at high and low concentration and without acid or base) was characterized using Scanning Electron Microscopy (SEM) (Figure S154). SEM images show a polycrystalline material, with individual crystals with sub‐micron dimensions. This is different from the petal‐shaped,^[^ ^27^ ^]^ ribbon‐shaped^[^ ^45^ ^]^ or bouquet‐like^[^ ^46^, ^47^ ^]^ morphologies reported in literature. This may be explained by the fact that the COF synthesis was performed while stirring, which may result in less fractal growth due to diffusion limitations. No difference in morphology was observed between samples of **TpPa** synthesized with acid, base, or without, and neither when the concentration of the additive was varied.

**Figure 2:**
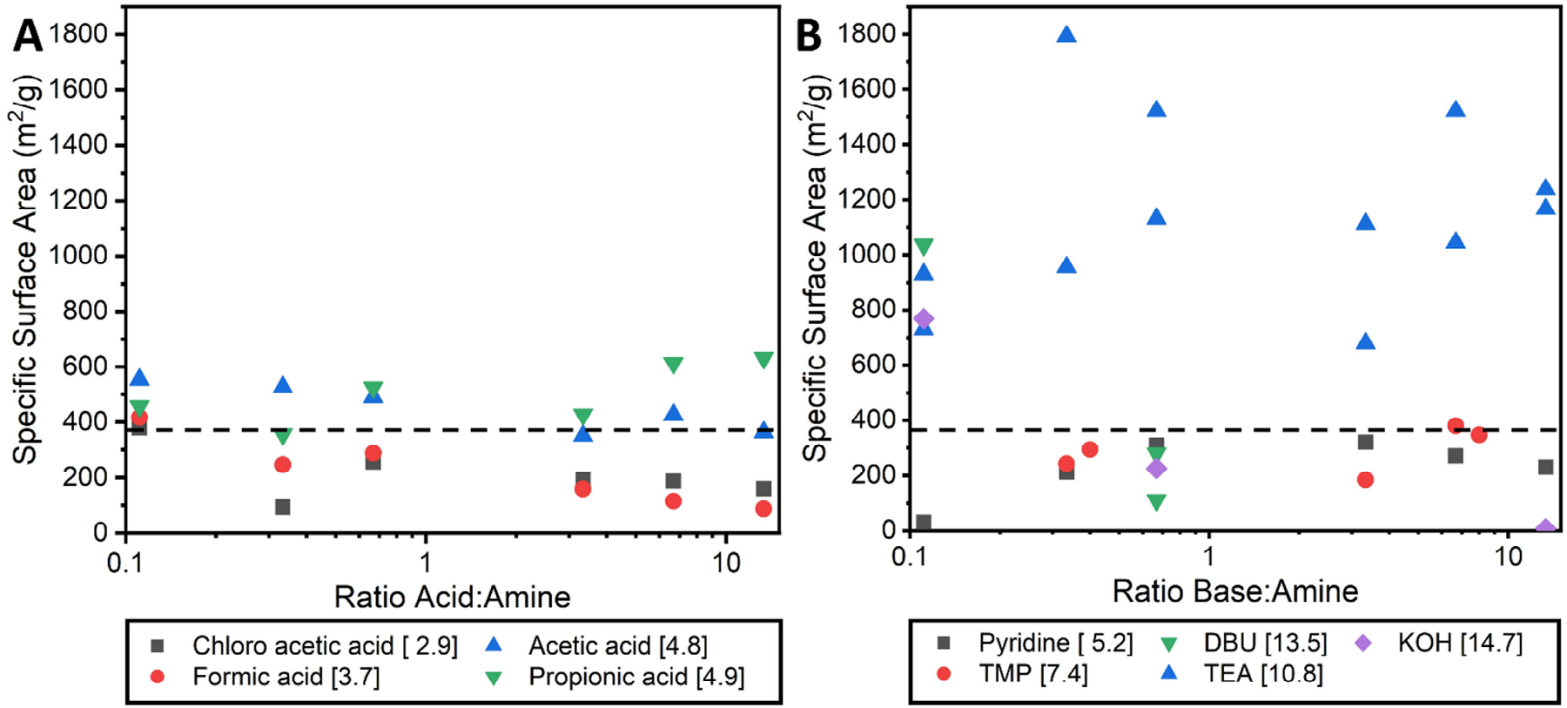
BET surface areas of **TpPa** synthesized with different concentrations of different A) acids or B) bases added [p*K*_a_ value of acid or conjugate acid]. The dashed lines indicate the average BET surface area of six samples made without the addition of acids or bases.

The permanent porosity of the COF samples was evaluated by nitrogen sorption measurements at 77 K (Figure 1C and Figures S82‐S153). The BET surface area of **TpPa** synthesized without the addition of acids is 382 ± 176 m^2^/g (dashed lines in Figure 2), which is similar to values reported before under similar conditions regarding (lack of) additive and solvent.^[^ ^48^ ^]^ The BET surface areas of the COFs made with varying acids and concentrations are shown in Figure 2A. It can be observed that when the p*K*_a_ value of the added acid decreases, the BET surface area of the COF decreases. Furthermore, when the concentration of the stronger acids increases, the BET surface area generally decreases. The micropore volume follows a similar trend. The micropore volume without the addition of acids is 0.14 ± 0.08 cm^3^/g, while the micropore volume with the use of acetic acid is 0.20 ± 0.05 cm^3^/g (averaged over the different concentrations), decreasing from 0.25 cm^3^/g to 0.15 cm^3^/g as the concentration of acetic acid increases.

**Scheme 2:**
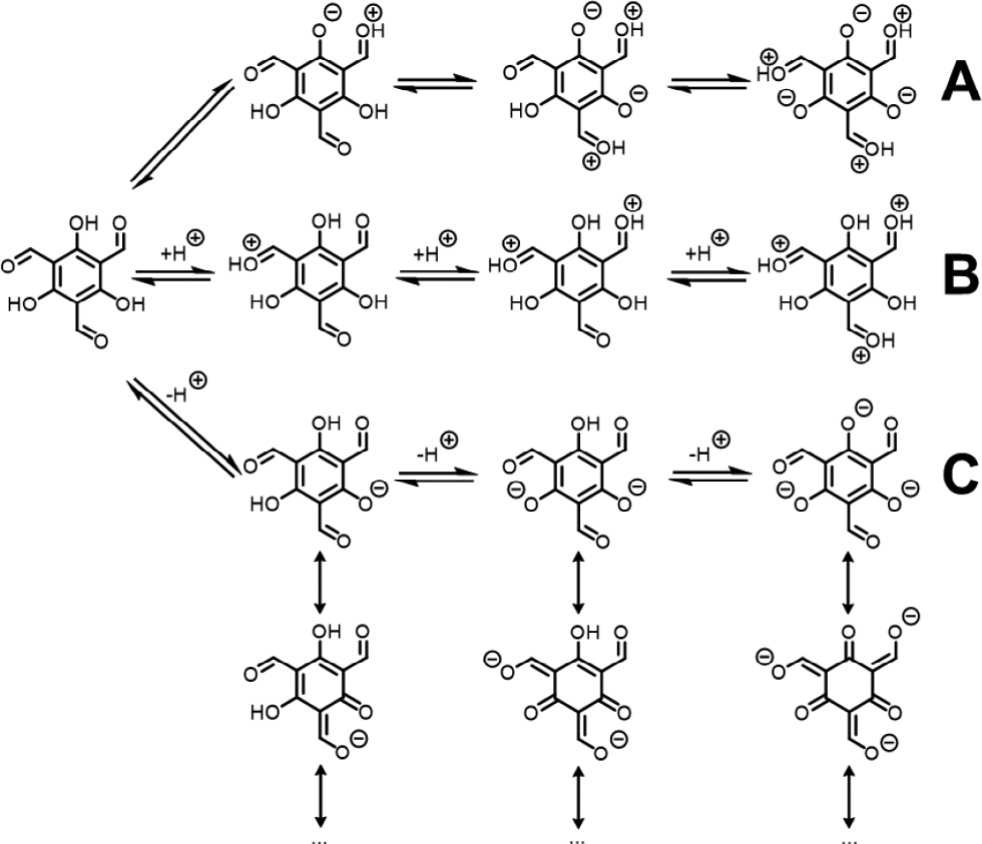
Proposed mechanism for the (de‐)activation of **Tp**. A) Self‐protonation of the aldehyde; B) Acidic protonation of the aldehyde, resulting in a more reactive species in the reaction with an amine to form the corresponding imine; C) Basic deprotonation of the phenol, leading to less reactive species in the reaction with an amine to form the corresponding imine.

To rationalize the reduced BET surface area when acid is used, we first note that the (initial) formation of **TpPa** is fast. That is, when the two monomers are brought together in solution, a red precipitate can be observed within seconds, without heating or the addition of water or acid. This is in strong contrast with imine COFs, for which the related building blocks can be dissolved together without formation of a COF network and only form a yellow precipitate after (acetic) acid is added.^[^ ^44^ ^]^ When forming the β‐ketoenamine‐linked COF, there is no intermediate yellow imine‐related phase observable. The immediate formation of the red precipitate suggests that the tautomerization is fast compared to the imine formation. This severely limits the opportunity the imine linkages have to revert and heal defects. In practice, the formation consists of one, irreversible step. To prevent the kinetic trapping of defects in the network, the formation rate should be reduced, so that it forms with fewer defects.^[^ ^28^, ^49^ ^]^ However, the β‐ketoenamine‐linked COF forming **Tp** (Scheme 2) is more reactive toward amines than its nonhydroxylated counterpart, which would only produce imine COFs. This is because the phenols can, by a proton transfer and subsequent delocalization of electrons, activate the carbonyl intermolecularly (Scheme 2A), with the precipitate forming in seconds. Though acids can reduce the reactivity of the amine building block via protonation, concomitantly they also further activate the carbonyl (Scheme 2B). Hence, (more of) a stronger acid leads to a faster and less organized network formation, resulting in a less ordered structure, causing a decreased BET surface area.

As the phenolic hydrogens of **Tp** are acidic, they can be deprotonated by a strong enough base. The deprotonated phenols can no longer self‐activate the carbonyl (Scheme 2C). This should lower the formation rate and result in a more crystalline COF with less defects. This rationalization prompted us to investigate the effect of organobases on the BET surface area of **TpPa**.

It was observed that pyridine and TMP, which have a neutral or slightly acidic p*K*_a_ of the conjugated acid, resulted in **TpPa** with lower BET surface areas compared to **TpPa** synthesized without added acids/bases (Figure 2B). The very alkaline DBU and KOH resulted in low BET surface area **TpPa** as well, except at very low concentrations. In contrast, and interestingly, TEA, with a moderate p*K*_a_ value of 10.8, resulted in **TpPa** with high BET surface areas above 1400 m^2^/g, which is among the highest reported for **TpPa**. Also, the micropore volume found for **TpPa** synthesized with TEA is 0.49 ± 0.12 cm^3^/g, a significant increase compared to the conditions without additive or with acetic acid.

**Scheme 3:**
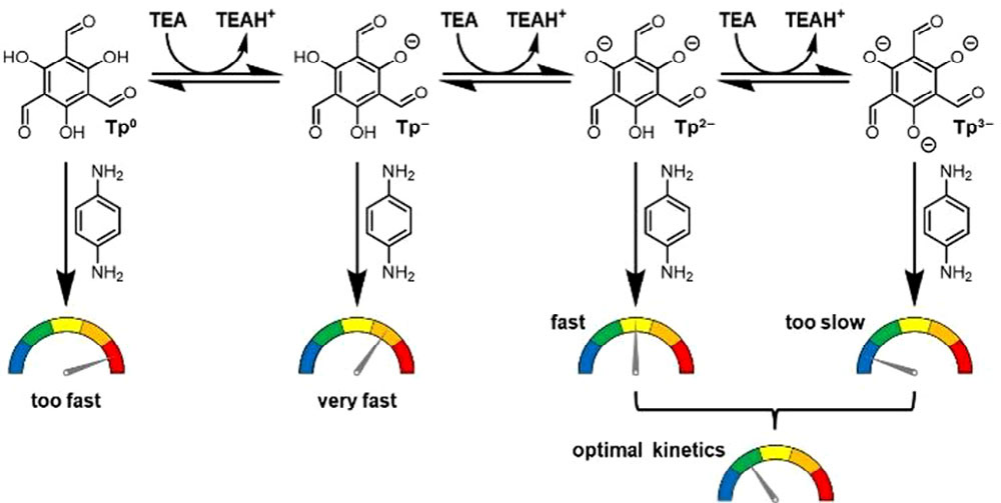
Proposed mechanism for the deactivation of **Tp** by TEA (DIPEA follows the same path). The p*K*_a_ values of **Tp^0^**, **Tp^1‐^,** and **Tp^2‐^** are 4.0, 7.8, and 10.8, respectively (Figure S155). Because the p*K*_a_ value of **Tp^2‐^** matches that of TEA, **Tp** will be present as both **Tp^2‐^** and **Tp^3‐^** (which is fully deactivated). Control over the population of **Tp^2‐^** and **Tp^3‐^** leads to optimal kinetics.

We postulate that bases with a low p*K*_a_ for the conjugate acid (such as pyridine and TMP) do not sufficiently slow down the network formation, leading to similar results as those of acids (Scheme 3). At high concentrations, bases with high p*K*_a_ conjugate acids (in our study: DBU and KOH) lead to very low yields and amorphous products (Figures 2B, and S81), indicating that these decelerate the network formation too much (Scheme 3). These results indicate an optimal p*K*_a_ value that allows to achieve a high BET surface area and crystallinity for **TpPa**.

We expect this optimum is caused by the number of phenolic hydroxyl groups of **Tp** that are deactivated by the added base in the COF synthesis. As **Tp** has three phenols, there are three corresponding p*K*_a_ values. A pH titration was performed to determine these p*K*_a_ values. To this end, **Tp** was dissolved in water with an excess of NaOH and titrated with an HCl solution. The p*K*_a_ values were found to be 4.0, 7.8, and 10.8 (Figure S155). This final p*K*_a_ value is similar to the p*K*_a_ value of TEA. This means that with the addition of TEA, part of the **Tp** will be fully deprotonated and thereby deactivated (Scheme 3). The deactivation of part of the **Tp** in solution is what regulates the effective **Tp** concentration, similar to the protecting group strategy as shown by Vitaku et al.^[^ ^32^ ^]^ As linkage formation within the 2D structure (internal linking) is concentration independent, in contrast to monomer addition, decreasing the effective concentration via deprotonation of (on average) 2.5 out of 3 aldehyde‐activating phenols of **Tp**, will favor internal linking over monomer addition and will result in more crystalline COFs with higher BET surface areas.^[^ ^28^ ^]^ The previously reported results of **TpPa** synthesized by pyrrolidine (conjugate acid p*K*_a_ 11.3) can partially be explained by this p*K*_a_ optimum, although the main mechanism involves the formation of an iminium intermediate.^[^ ^26^ ^]^

**Figure 3:**
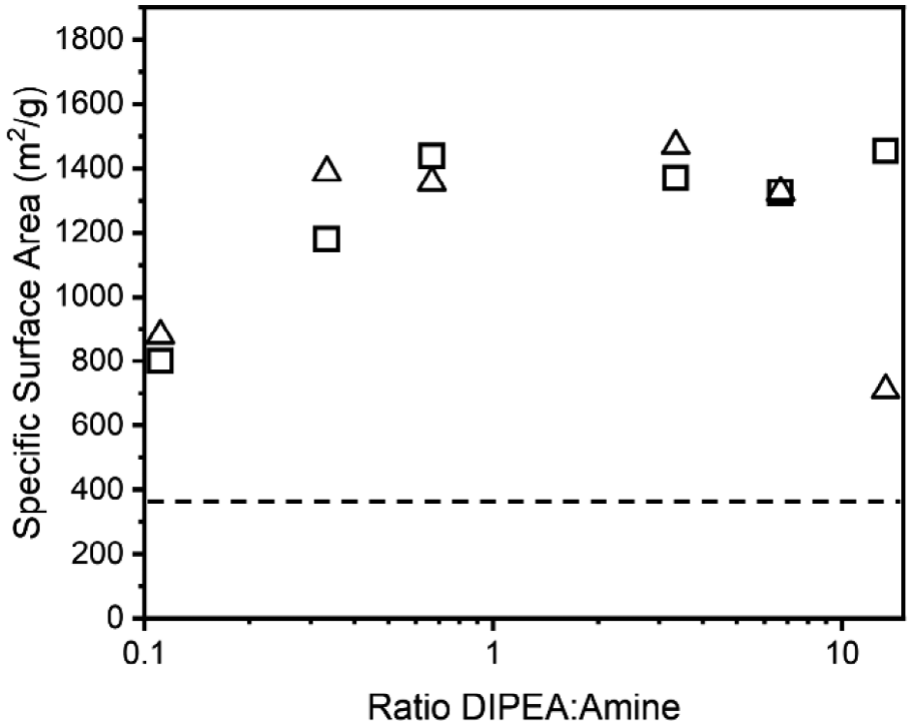
BET surface areas of **TpPa** synthesized with varying concentrations of DIPEA. The dashed line indicates the average BET surface area of six samples made without the addition of acids or bases. For clarity, the data points for duplicate syntheses are shown by a different symbol.

To confirm that the p*K*_a_ of the additive indeed should match the p*K*_a_ of **Tp**, we selected diisopropylethylamine (DIPEA), another readily available organobase with a p*K*_a_ value of the conjugate acid that is very similar to that of TEA (i.e., 11.0). Also, similar to TEA, DIPEA is not capable to form an iminium intermediate, in contrast to pyrrolidine. **TpPa** was synthesized at different DIPEA concentrations. As for TEA, also for DIPEA relatively sharp peaks are visible in the PXRD spectrum (Figure 1A, and Figures S79, S80), suggesting high crystallinity. From the BET surface areas (Figure 3, and Figures S136–S147), it can be concluded that **TpPa** synthesized with DIPEA has equally high, if not more consistent, BET surface areas compared to **TpPa** synthesized with TEA (Figure 3). The micropore volume found for **TpPa** synthesized with DIPEA is on average 0.57 ± 0.37 cm^3^/g, comparable to values found for **TpPa** synthesized with TEA. These results for DIPEA thus fully support our hypothesis that the p*K*_a_ of the additive should match with that of **Tp**.

## 3 Conclusion

We have performed a systematic study investigating the effect of the p*K*_a_ of various acidic and basic additives on the BET surface area and micropore volume of the **TpPa** COFs. We have found that not acetic acid, while used ubiquitously in **TpPa** synthesis, but the organobases TEA and DIPEA work best in achieving crystalline COFs with large micropore volumes and BET surface areas that are among the highest reported. By deprotonating on average 2.5 out of 3 aldehyde‐activating phenols of **Tp**, these two organobase additives act as rate controllers. This way, internal linking is promoted over linker addition, reducing the chance of kinetically trapped defects, resulting in a more crystalline product with a high BET surface area.

To conclude, we show by the example of **TpPa** that fine‐tuning the p*K*_a_ value of the acid/base additive to those of the COF building blocks, proved to be key in enhancing the material properties. As β‐ketoamine‐linked COFs represent a significant and highly stable class of COFs, we consider this approach as an important step in advancing the control in their synthesis, further stimulating the development of porous nanomaterials across a wide range of applications.

## Supporting Information

Full experimental details of the COF synthesis and characterization, the titration of **Tp,** as well as the results of our literature review can be found in the Supporting Information.

## Conflict of Interest

The authors declare no conflict of interest.

## Supporting information



Supporting Information

## Data Availability

The data that support the findings of this study are available from the corresponding author upon reasonable request.
