# Supplementary material for: On the Importance of Balancing the pK a of the Additive in β‐Ketoenamine COF Synthesis
Source: Chemistry. 2025 Jun 18;31(39):e202501512. doi: 10.1002/chem.202501512 (PMC12258664; doi:10.1002/chem.202501512)
Supplement: Supplementary file 1 — Supporting Information [file CHEM-31-e202501512-s001.pdf]

Supporting Information

On the Importance of Balancing the pK_a of the Additive in β-Ketoenamine COF Synthesis

Thijmen A. van Voorthuizen,[a] Monique A. van der Veen,[b] Louis C. P. M. de Smet,*[a] Maarten M. J. Smulders*[a]

[a] Laboratory of Organic Chemistry, Wageningen University, Stippeneng 4, 6708 WE Wageningen, The Netherlands

[b] Department of Chemical Engineering, Delft University of Technology, Van der Maasweg 9, 2629 HZ Delft, The Netherlands

* louis.desmet@wur.nl; maarten.smulders@wur.nl

Contents

1. Literature review S3
2. General Information S14
3. Synthetic Procedures S15
4. ATR-FTIR spectra S16
   4.1 Chloroacetic acid S16
   4.2 Formic acid S17
   4.3 Acetic acid S19
   4.4 Propionic acid S20
   4.5 No acid/base S22
   4.6 Pyridine S23
   4.7 2,4,6-trimethylpyridine S25
   4.8 TEA S26
   4.9 DIPEA S29
   4.10 DBU S32
   4.11 KOH S33
5. PXRD S35
6. N_2 sorption isotherms/BET graphs S37
   6.1 Chloroacetic acid S37
   6.2 Formic acid S43
   6.3 Acetic acid S49
   6.4 Propionic acid S55
   6.5 No acid/base S61
   6.6 Pyridine S67
   6.7 2,4,6-trimethylpyridine S73
   6.8 TEA S79
   6.9 DIPEA S91
   6.10 DBU S103
   6.11 KOH S106
7. SEM S109
8. Titration of **Tp** S110
9. References S111

1. Literature review

Table S1: An extensive overview of the synthetic conditions and achieved surface areas of **TpPa** reported in literature (survey performed on March 4, 2025).

Table columns: **1**: Synthetic method; **2**: Acid, base or neither used; **3**: Specific acid or base used; **4**: Equivalents of the acid or base (compared to 1 equivalent **Tp**); **5**; Solvent; **6**: BET specific surface area in m^2/g; **7**: First author; **8**: Year of publication; **9**: Journal of publication; **10**: DOI. Abbreviations used: **ST**: Solvothermal synthesis; **MW**: Microwave assisted; **PI**: Plasma induced; **SG**: Substrate growth; **MC**: Mechanochemistry; **IS**: Interfacial synthesis; **Misc.**: Miscellaneous; **NR**: Not reported; **A**: Acid; **B**: Base; **NVR**: No value reported; **PTSA**: p-toluenesulfonic acid; **[BSMIm]HSO$_4$**: 1-(4-sulfonic acid)-butyl-3-methylimidazolium hydrogen sulfate; **DMF**: *N,N*-dimethylformamide; **DMAc**: *N,N*-Dimethylacetamide; **DMSO**: dimethyl sulfoxide; **NMP**: *N*-methyl-2-pyrrolidone; **o-DCB**: o-dichlorobenzene; **PEG**: polyethylene glycol; **THF**: tetrahydrofuran .

1	2	3	4	5	6	7	8	9	10
ST	A	Acetic acid	5	Mesitylene:1,4-dioxane 1:1	535	Kandambeth	2012	J. Am. Chem. Soc.	10.1021/ja308278w
ST	A	Acetic acid	5	Mesitylene:1,4-dioxane 1:1	535	Chandra	2013	J. Am. Chem. Soc.	10.1021/ja408121p
ST	A	Acetic acid	3.1	1,4-dioxane	365	DeBlase	2013	J. Am. Chem. Soc	10.1021/ja409421d
ST	A	Acetic acid	5	Mesitylene:1,4-dioxane 1:1	484	Pachfule	2014	J. Mater. Chem. A	10.1039/c4ta00284a
ST	A	Acetic acid	5	Mesitylene:1,4-dioxane 1:1	984	Biswal	2015	J. Mater. Chem. A	10.1039/c5ta07998e
ST	A	Acetic acid	5	Mesitylene:1,4-dioxane 1:1	479	Pachfule	2015	Chem. Commun.	10.1039/c3cc49176e
ST	A	Acetic acid	5	Mesitylene:1,4-dioxane 1:1	801	Biswal	2016	Chem. Eur. J.	10.1002/chem.201504836
ST	A	Acetic acid	15.6	1,4-dioxanne	385	Khattak	2016	J. Mater. Chem. A	10.1039/c6ta05784e
ST	A	Acetic acid	5.3	1,4-dioxane	834	Peng	2016	Chem. Mater.	10.1021/acs.chemmater.6b01954
ST	A	Acetic acid	105	Water	633	Thote	2016	IUCrJ	10.1107/s2052252516013762
ST	A	Acetic acid	NR	Mesitylene:1,4-dioxane 1:1	NR	Chandra	2017	Chem. Mater.	10.1021/acs.chemmater.6b04178
ST	A	Acetic acid	10	Mesitylene: dioxane 1:1	1277	Vitaku	2017	J. Am. Chem. Soc.	10.1021/jacs.7b06913
ST	A	Acetic acid	5	Mesitylene: dioxane 1:1	NR	Biswal	2018	Sep. Sci. Technol.	10.1080/01496395.2018.1443136
ST	A	Acetic acid	5	Mesitylene: dioxane 1:1	832	Han	2018	Nat. Commun.	10.1038/s41467-018-03689-9
ST	A	Acetic acid	5	Mesitylene: dioxane 1:1	NR	Li	2018	J. Mater. Chem. A	10.1039/c8ta03275k
ST	A	Acetic acid	NR	1,4-dioxane	166	Wang	2018	ACS Appl. Mater. Interfaces	10.1021/acsami.8b06229

ST	A	Acetic acid	0.2	1,4-dioxane	742	Yang	2018	J. Membr. Sci.	10.1016/j.memsci.2018.05.036
ST	A	Acetic acid	5	Mesitylene: dioxane 1:1	518	Cheng	2019	J. Membr. Sci.	10.1016/j.memsci.2018.05.036
ST	A	Acetic acid	5	DMF	450	Gao	2019	J. Mater. Chem. A	10.1039/c9ta07319a
ST	A	Acetic acid	10	Mesitylene: dioxane 1:1	625	Liu	2019	Chem. Eng. J.	10.1016/j.cej.2018.09.081
ST	A	Acetic acid	5	Mesitylene: dioxane 1:1	NR	Ming	2019	Angew. Chem. Int. Ed.	10.1002/ange.201912344
ST	A	Acetic acid	5	Mesitylene: dioxane 1:1	840	Pérez-Carvajal	2019	Adv. Energy Mater.	10.1002/aenm.201901535
ST	A	Acetic acid	5	Mesitylene: dioxane 1:1	542	Sheng	2019	ChemCatChem	10.1002/cctc.201900058
ST	A	Acetic acid	40	Mesitylene: dioxane 1:1	NR	Shi	2019	J. Membr. Sci.	10.1016/j.memsci.2019.01.034
ST	A	Acetic acid	5	Mesitylene: dioxane 1:1	NR	Wang	2019	J. Mater. Chem. A	10.1039/c8ta11883c
ST	A	Acetic acid	5	Mesitylene:1,4-dioxane 1:1	842	Wu	2019	J. Membr. Sci.	10.1016/j.memsci.2019.01.040
ST	A	Acetic acid	5	Butyl alcohol:1,2-dichlorobenzene 1:1	765	Yang	2019	J. Solid State Chem.	10.1016/j.jssc.2019.120979
ST	A	Acetic acid	5	Mesitylene:1,4-dioxane 1:1	669	Zhang	2019	J. Am. Chem. Soc.	10.1021/jacs.9b01561
ST	A	Acetic acid	5	Mesitylene:1,4-dioxane 1:1	NR	Zhang	2019	Polymers	10.3390/polym11040708
ST	A	Acetic acid	24	Mesitylene:1,4-dioxane 1:1	519	Chen	2020	J. Mater. Chem. A	10.1039/d0ta04488a
ST	A	Acetic acid	5	1,4-dioxane	NR	Dong	2020	Chem. Eng. J.	10.1016/j.cej.2019.122342
ST	A	Acetic acid	10	Mesitylene:1,4-dioxane 1:1	475	Khaing	2020	Inorg. Chem.	10.1021/acs.inorgchem.0c00422
ST	A	Acetic acid	15	Mesitylene:1,4-dioxane 1:1	542	Li	2020	Appl. Catal. B: Environ.	10.1016/j.apcatb.2020.118586
ST	A	Acetic acid	2.5	Mesitylene:1,4-dioxane 1:1	204	Ma	2020	Microporous and Mesoporous Mater.	10.1016/j.micromeso.2020.110287
ST	A	Acetic acid	5	Mesitylene:1,4-dioxane 1:1	817	Wang	2020	ACS Appl. Mater. Interfaces	10.1021/acsami.0c00101
ST	A	Acetic acid	38.7	Mesitylene:1,4-dioxane:water 3:3:1	600	Wang	2020	Ind. Eng. Chem. Res.	10.1021/acs.iecr.0c03797
ST	A	Acetic acid	82.2	Mesitylene:1,4-dioxane:water 3:3:1	461	Wang	2020	Ind. Eng. Chem. Res.	10.1021/acs.iecr.0c03798
ST	A	Acetic acid	241.7	Mesitylene:1,4-dioxane:water 3:3:1	82	Wang	2020	Ind. Eng. Chem. Res.	10.1021/acs.iecr.0c03799
ST	A	Acetic acid	5	Mesitylene:1,4-dioxane 1:1	469	Wen	2020	Anal. Methods	10.1039/c9ay02755f
ST	A	Acetic acid	12.9	o-DCB:DMAc 9:1	NR	Zhang	2020	Nanoscale	10.1039/d0nr06854c
ST	A	Acetic acid	5	Mesitylene:1,4-dioxane 1:1	NR	Zhang	2020	Microchim. Acta	10.1007/s00604-020-04435-z

ST	A	Acetic acid	5	Mesitylene:1,4-dioxane 1:1	452	Zhong	2020	Appl. Surf. Sci.	10.1016/j.apsusc.2019.144403
ST	A	Acetic acid	5	Mesitylene:1,4-dioxane 1:1	879	Cao	2021	Appl. Catal. B: Environ.	10.1016/j.apcatb.2021.120238
ST	A	Acetic acid	46	DMSO	NVR	Chang	2021	Eur. Food Res. Technol.	10.1007/s00217-021-03830-x
ST	A	Acetic acid	30	Mesitylene:1,4-dioxane 1:1	132	Gopalakrishan	2021	ACS Appl. Energy Mater.	10.1021/acsaem.1c00862
ST	A	Acetic acid	4.5	Mesitylene:1,4-dioxane 1:1	698	Guo	2021	Chem. Eng. J.	10.1016/j.cej.2020.127011
ST	A	Acetic acid	10	Mesitylene:1,4-dioxane 1:1	227	Li	2021	New J. Chem.	10.1039/d1nj03133c
ST	A	Acetic acid	5	Mesitylene:1,4-dioxane 1:1	313	Liang	2021	ACS Appl. Nano Mater.	10.1021/acsanm.1c00199
ST	A	Acetic acid	5	Mesitylene:1,4-dioxane 1:1	543	Liu	2021	Environ. Sci. Technol.	10.1021/acs.est.0c07857
ST	A	Acetic acid	5	Mesitylene:1,4-dioxane 1:1	499	Singh	2021	J. CO2 Util.	10.1016/j.jcou.2021.101716
ST	A	Acetic acid	5	Mesitylene:1,4-dioxane 1:1	421	Wang	2021	Angew. Chem. Int. Ed.	10.1002/anie.202106346
ST	A	Acetic acid	5	Mesitylene:1,4-dioxane 1:1	578	Wang	2021	Angew. Chem. Int. Ed.	10.1002/anie.202106346
ST	A	Acetic acid	10	Mesitylene:1,4-dioxane 1:1	368	Wang	2021	Small	10.1002/smll.202101017
ST	A	Acetic acid	7.5	1,4-dioxane	715	Wang	2021	Chem. Commun.	10.1039/d0cc06519f
ST	A	Acetic acid	5	Mesitylene:1,4-dioxane 1:1	NR	Wen	2021	ACS Appl. Bio Mater.	10.1021/acsabm.1c00315
ST	A	Acetic acid	1	1,4-dioxane	NR	Wu	2021	Anal. Sci.	10.2116/analsci.21c001
ST	A	Acetic acid	NR	1,4-dioxane	430	Wu	2021	Sci. China	10.1007/s11426-021-1088-2
ST	A	Acetic acid	0.6	1,4-dioxane	NVR	Wu	2021	J. Radioanal. Nucl. Chem.	10.1007/s10967-021-07971-x
ST	A	Acetic acid	5	Mesitylene:1,4-dioxane 1:1	NR	Xue	2021	ACS Appl. Mater. Interfaces	10.1021/acsami.1c18238
ST	A	Acetic acid	10	Mesitylene:1,4-dioxane 1:1	841	Yin	2021	Chem. Eng. J.	10.1016/j.cej.2021.129984
ST	A	Acetic acid	6	Mesitylene:1,4-dioxane 1:1	587	Yuan	2021	Chem. Res. Chin. Univ.	10.1007/s40242-021-1064-z
ST	A	Acetic acid	5	Mesitylene:1,4-dioxane 1:1	NR	Yuan	2021	Food Chem.	10.1016/j.foodchem.2020.128299
ST	A	Acetic acid	51.8	Mesitylene:1,4-dioxane 1:1	407	Zhang	2021	Front. Chem. Sci. Eng.	10.1007/s11705-021-2104-4
ST	A	Acetic acid	5	Mesitylene:1,4-dioxane 1:1	111	Zhu	2021	Chem. Mater.	10.1021/acs.chemmater.0c04237
ST	A	Acetic acid	5	Mesitylene:1,4-dioxane 1:1	376	Zhu	2021	Chem. Mater.	10.1021/acs.chemmater.0c04237
ST	A	Acetic acid	10	Mesitylene:1,4-dioxane 1:1	NR	Cai	2022	Anal. Chem.	10.1021/acs.analchem.1c05002

ST	A	Acetic acid	5	Mesitylene:1,4-dioxane 1:1	NR	Cen	2022	Ind. Eng. Chem. Res.	10.1021/acs.iecr.2c01635
ST	A	Acetic acid	5	Mesitylene:1,4-dioxane 1:1	996	Chen	2022	Angew. Chem. Int. Ed.	10.1002/ange.202114071
ST	A	Acetic acid	90	Mesitylene:1,4-dioxane 1:1	576	Chen	2022	J. Alloys Compd.	10.1016/j.jallcom.2022.166524
ST	A	Acetic acid	5	Mesitylene:1,4-dioxane 1:1	564	Deng	2022	Microchem. J.	10.1016/j.microc.2022.107924
ST	A	Acetic acid	5	Mesitylene:1,4-dioxane 1:1	486	Kong	2022	Molecules	10.3390/molecules27248704
ST	A	Acetic acid	5	Mesitylene:1,4-dioxane 1:1	555	Liu	2022	ChemElectroChem	10.1002/celc.202200123
ST	A	Acetic acid	1.7	Mesitylene:1,4-dioxane 1:1	1004	Liu	2022	Sep. Purif. Technol.	10.1016/j.seppur.2022.120644
ST	A	Acetic acid	5	Mesitylene:1,4-dioxane 1:1	926	Sun	2022	J. Mater. Sci. Technol.	10.1016/j.jmst.2021.12.065
ST	A	Acetic acid	5	Mesitylene:1,4-dioxane 1:1	NR	Tahir	2022	J. Mater. Sci. Technol.	10.1002/er.8364
ST	A	Acetic acid	10	Mesitylene:1,4-dioxane 1:1	227	Wang	2022	Langmuir	10.1021/acs.langmuir.2c00203
ST	A	Acetic acid	5	Mesitylene:1,4-dioxane 1:1	684	Wang	2022	ACS Sustainable Chem. Eng.	10.1021/acssuschemeng.1c06318
ST	A	Acetic acid	2.5	Mesitylene:1,4-dioxane:acetonitrile	798	Wu	2022	J. Membr. Sci.	10.1016/j.memsci.2022.120799
ST	A	Acetic acid	1	Choline chloride:oxalic acid dihydrate 1:1	152	Xiao	2022	Environ. Res.	10.1016/j.envres.2022.113977
ST	A	Acetic acid	10	DMF	919	Yan	2022	Small	10.1002/smll.202201340
ST	A	Acetic acid	5	Mesitylene:1,4-dioxane 1:1	490	Yan	2022	Chem. Eng. J.	10.1016/j.cej.2021.133404
ST	A	Acetic acid	5	Mesitylene:1,4-dioxane 1:1	652	Yang	2022	J. Membr. Sci.	10.1016/j.memsci.2022.120944
ST	A	Acetic acid	8	Mesitylene:1,4-dioxane 1:3	157	Yang	2022	Chem. Eng. J.	10.1016/j.cej.2022.138040
ST	A	Acetic acid	NR	1,4-dioxane:water	383	Yang	2022	J. Membr. Sci.	10.1016/j.memsci.2022.120944
ST	A	Acetic acid	NR	1,4-dioxane:water	322	Yang	2022	Chin. J. Polym. Sci.	10.1007/s10118-022-2676-6
ST	A	Acetic acid	NR	1,4-dioxane:water	73	Yang	2022	Chin. J. Polym. Sci.	10.1007/s10118-022-2676-6
ST	A	Acetic acid	5	DMF	569	Yao	2022	J. Colloid Interface Sci.	10.1016/j.jcis.2021.10.183
ST	A	Acetic acid	7.5	Mesitylene:1,4-dioxane 1:1	1031	Zhang	2022	J. Sep. Sci.	10.1002/jssc.202100905
ST	A	Acetic acid	6	NMP:o-DCB 4:1	1712	Zhang	2022	Nature	10.1038/s41586-022-04443-4
ST	A	Acetic acid	5	Mesitylene:1,4-dioxane 1:1	NR	Zhang	2022	Mater. Today Chem.	10.1016/j.mtchem.2022.101150
ST	A	Acetic acid	5	Mesitylene:1,4-dioxane 1:1	NR	Zhang	2022	Front. Mater.	10.3389/fmats.2021.820671

ST	A	Acetic acid	NR	Glycerol	NR	Zhang	2022	Chem. Mater.	10.1021/acs.chemmater.2c00834
ST	A	Acetic acid	NR	Glycerol:ethanol (various ratios)	NR	Zhang	2022	Chem. Mater.	10.1021/acs.chemmater.2c00834
ST	A	Acetic acid	NR	isopropanol	NR	Zhang	2022	Chem. Mater.	10.1021/acs.chemmater.2c00835
ST	A	Acetic acid	NR	ethylene glycol	NR	Zhang	2022	Chem. Mater.	10.1021/acs.chemmater.2c00836
ST	A	Acetic acid	NR	PEG-400	NR	Zhang	2022	Chem. Mater.	10.1021/acs.chemmater.2c00837
ST	A	Acetic acid	NR	PPG-8000	NR	Zhang	2022	Chem. Mater.	10.1021/acs.chemmater.2c00838
ST	A	Acetic acid	NR	Ethanol	NR	Zhang	2022	Chem. Mater.	10.1021/acs.chemmater.2c00839
ST	A	Acetic acid	5.3	1,4-dioxane	53	Zhao	2022	Spectrochim. Acta A	10.1016/j.saa.2021.120643
ST	A	Acetic acid	5	Mesitylene:1,4-dioxane 1:1	778	Zhou	2022	Nanomaterials	10.3390/nano12142482
ST	A	Acetic acid	2	1,4-dioxane	339	Chu	2023	ACS Catal.	10.1021/acscatal.3c03857
ST	A	Acetic acid	40	Water:Toluene 12:14	402	Fan	2023	ACS Nano	10.1021/acsnano.2c12774
ST	A	Acetic acid	8	Mesitylene:1,4-dioxane 1:1	NR	He	2023	Appl. Catal. A	10.1016/j.apcata.2023.119320
ST	A	Acetic acid	NR	Water	1199	Hu	2023	J. Am. Chem. Soc.	10.1021/jacs.3c10053
ST	A	Acetic acid	9	Mesitylene:1,4-dioxane 1:1	NR	Jaryal	2023	Mater. Today: Proc.	10.1016/j.matpr.2022.12.093
ST	A	Acetic acid	NR	Mesitylene:1,4-dioxane 1:1	880	Lee	2023	Adv. Energy Mater.	10.1002/aenm.202300442
ST	A	Acetic acid	5	Mesitylene:1,4-dioxane 1:1	12	Li	2023	ACS Appl. Mater. Interfaces	10.1021/acsami.3c09459
ST	A	Acetic acid	NR	Water	1007	Martínez-Visus	2023	Chem. Eur. J.	10.1002/chem.202203907
ST	A	Acetic acid	10	Mesitylene:1,4-dioxane 1:1	498	Ranasinghe Arachchige	2023	ACS Appl. Nano Mater.	10.1021/acsanm.3c00442
ST	A	Acetic acid	NR	Mesitylene:1,4-dioxane 1:1	903	Wang	2023	J. Hazard. Mater.	10.1016/j.jhazmat.2023.132522
ST	A	Acetic acid	40	Mesitylene:1,4-dioxane 1:1	927	Wang	2023	eScience	10.1016/j.esci.2023.100154
ST	A	Acetic acid	3	Mesitylene:1,4-dioxane 1:1	774	Weng	2023	JACS Au	10.1021/jacsau.3c00554
ST	A	Acetic acid	5	Mesitylene:1,4-dioxane 1:1	181	Xia	2023	Colloids Surf. A	10.1016/j.colsurfa.2023.131124
ST	A	Acetic acid	5	Mesitylene:1,4-dioxane 1:1	800	Xin	2023	J. Membr. Sci.	10.1016/j.memsci.2022.121109
ST	A	Acetic acid	5	Mesitylene:1,4-dioxane 1:1	837	Xiong	2023	Appl. Catal. B: Environ.	10.1016/j.apcatb.2022.122135
ST	A	Acetic acid	10	DMF	743	Yan	2023	Nano-Micro Lett.	10.1007/s40820-023-01100-x

ST	A	Acetic acid	5	Mesitylene:1,4-dioxane 1:1	NR	Yang	2023	Biosens. Bioelectron.	10.1016/j.bios.2022.115017
ST	A	Acetic acid	8	Mesitylene:1,4-dioxane 1:3	NR	Yang	2023	Inorg. Chem. Front.	10.1039/d3qi00217a
ST	A	Acetic acid	10	Mesitylene:1,4-dioxane 1:1	NVR	Ye	2023	Nat. Commun.	10.1038/s41467-023-37296-0
ST	A	Acetic acid	NR	Mesitylene:1,4-dioxane 1:1	660	Yuan	2023	Nat. Chem.	10.1038/s41557-023-01273-3
ST	A	Acetic acid	11.8	Mesitylene:1,4-dioxane 1:1	330	Zhao	2023	J. Mater. Chem. A	10.1039/d3ta06222h
ST	A	Acetic acid	12	Mesitylene:1,4-dioxane 1:1	689	Zhong	2023	Chem. Eng. J.	10.1016/j.cej.2023.143415
ST	A	Acetic acid	5	Mesitylene:1,4-dioxane 1:1	513	Fan	2024	J. Colloid Interface Sci.	10.1016/j.jcis.2024.06.087
ST	A	Acetic acid	10	Mesitylene:1,4-dioxane 1:1	NR	Gai	2024	Talanta	10.1016/j.talanta.2024.126407
ST	A	Acetic acid	12.5	Mesitylene:1,4-dioxane 1:1	543	Li	2024	Colloids Surf. A	10.1016/j.colsurfa.2024.134792
ST	A	Acetic acid	149	1,4-dioxane	687	Li	2024	Environ. Sci. Pollut. Res.	10.1007/s11356-024-32312-2
ST	A	Acetic acid	5	Mesitylene:1,4-dioxane 1:1	695	Li	2024	Int. J. Hydrog. Energy	10.1016/j.ijhydene.2024.02.118
ST	A	Acetic acid	5	Mesitylene:1,4-dioxane 1:1	407	Ma	2024	ChemSusChem	10.1002/cssc.202400987
ST	A	Acetic acid	113	Mesitylene:1,4-dioxane 1:1	877	Mei	2024	Small	10.1002/smll.202403521
ST	A	Acetic acid	10	Mesitylene:1,4-dioxane 1:1	504	Wang	2024	J. Membr. Sci.	10.1016/j.memsci.2024.122470
ST	A	Acetic acid	69	Mesitylene:1,4-dioxane 1:1	NR	Wu	2024	J. Colloid Interface Sci.	10.1016/j.jcis.2024.02.038
ST	A	Acetic acid	5	Mesitylene:1,4-dioxane 1:1	49	Wu	2024	J. Membr. Sci.	10.1016/j.memsci.2024.122854
ST	A	Acetic acid	5	Mesitylene:1,4-dioxane 1:1	726	Xia	2024	Catal. Sci. Technol.	10.1039/d3cy01571h
ST	A	Acetic acid	120	Mesitylene:1,4-dioxane 1:1	NR	Xia	2024	J. Chromatogr. A	10.1016/j.chroma.2024.464998
ST	A	Acetic acid	290	Glycerol	87	Xu	2024	J. Membr. Sci.	10.1016/j.memsci.2024.122678
ST	A	Acetic acid	5	Mesitylene:1,4-dioxane 1:1	470	Yuan	2024	Environ. Pollut.	10.1016/j.envpol.2024.124191
ST	A	Acetic acid	5	Mesitylene:1,4-dioxane 1:1	216	Zhao	2024	J. Membr. Sci.	10.1016/j.memsci.2024.122676
ST	A	Acetic acid	5	1,4-dioxane	NR	Zhao	2024	Desalination	10.1016/j.desal.2024.117819
ST	A	Acetic acid	10	Mesitylene:1,4-dioxane 1:1	485	Zhao	2024	J. Colloid Interface Sci.	10.1016/j.jcis.2023.09.060
ST	A	Acetic acid	5	DMF:Mesitylene:1,4-dioxane 2:1:1	529	Zhao	2024	Ceram. Int.	10.1016/j.ceramint.2023.11.284
ST	A	Acetic acid	5	Mesitylene:1,4-dioxane 1:1	612	He	2024	Appl. Catal. B: Environ.	10.1016/j.apcatb.2024.123916

ST	A	Acetic acid	5	DMF	744	Wang	2024	Appl. Catal. B: Environ.	10.1016/j.apcatb.2024.124366
ST	A	Acetic acid	5	Mesitylene:1,4-dioxane 1:1	307	Dong	2024	ACS Catal.	10.1021/acscatal.4c04968
ST	A	Acetic acid	NVR	Mesitylene:1,4-dioxane	843	Zhao	2024	Chem. Eur. J.	10.1002/chem.202400377
ST	A	Acetic acid	11.6	DMF	NVR	Peng	2024	J. Mater. Chem. A	10.1039/d4ta04418e
ST	A	Acetic acid	6	Mesitylene:1,4-dioxane 1:1	659	Fan	2024	J. Solid State Chem.	10.1016/j.jssc.2024.125017
ST	A	Acetic acid	15.8	Mesitylene:1,4-dioxane 1:1	458	Zha	2024	Nano Res.	10.1007/s12274-024-6523-7
ST	A	Acetic acid	6	o-dichlorobenzene:butanol 1:1	850	Wang	2025	Molecules	10.3390/molecules30051004
ST	A	Acetic acid	7.5	Mesitylene:1,4-dioxane 1:1	NVR	Ouyang	2025	Angew. Chem.	10.1002/anie.202418790
ST	A	Acetic acid	6	Mesitylene:1,4-dioxane 1:1	532	Yang	2025	Int. J. Mol. Sci.	10.3390/ijms26051957
ST	A	Acetic acid	17.9	Mesitylene:1,4-dioxane 1:1	628	Zheng	2025	Nat. Commun.	10.1038/s41467-025-57166-1
ST	A	Acetic acid	17.9	Mesitylene:1,4-dioxane 1:1	933	Zheng	2025	Nat. Commun.	10.1038/s41467-025-57166-1
ST	A	Acetic acid	9	Mesitylene:1,4-dioxane 1:1	NVR	Zheng	2025	Nat. Commun.	10.1038/s41467-025-57166-1
ST	A	Acetic acid	9.6	Mesitylene:1,4-dioxane 1:1	624	Huang	2025	Adv. Funct. Mater.	10.1002/adfm.202413943
ST	A	Acetic acid	12.9	Mesitylene:1,4-dioxane 1:1	NVR	Zhou	2025	Anal. Bioanal. Chem.	10.1007/s00216-024-05687-x
ST	A	Acetic acid	8	Mesitylene:1,4-dioxane 1:4	719	Yan	2025	Angew. Chem.	10.1002/anie.202422851
ST	A	Acetic acid	5	Mesitylene:1,4-dioxane 1:1	107	Shi	2025	Chem. Eng. J.	10.1016/j.cej.2024.159188
ST	A	Acetic acid	290	Glycerol	68	Yuan	2025	Sep. Purif. Technol.	10.1016/j.seppur.2024.128315
ST	A	[BSMIm]HSO_4	31.6	[BSMIm]HSO_4	446	Dong	2019	Mater. Chem. Phys.	10.1016/j.matchemphys.2019.01.032
ST	A	PTSA	9.7	Water	NR	Mao	2024	Energies	10.3390/en17071559
ST	B	(R)-2-methylpyrrolidine	0.3	Mesitylene:1,4-dioxane 1:1	1105	Liu	2021	Environ. Sci. Technol.	10.1021/acs.est.0c07857
ST	B	(S)-2-methylpyrrolidine	0.3	Mesitylene:1,4-dioxane 1:1	1098	Li	2022	Angew. Chem. Int. Ed.	10.1002/anie.202115044
ST	B	KOH	3	DMF:Water 2:3	1247	Zhang	2020	Green Chem.	10.1039/c9gc04033a
ST	B	KOH	0.6	Water	154	Liu	2021	J. Ind. Eng. Chem.	10.1016/j.jiec.2021.03.012
ST	B	KOH	0.6	Water	NR	Chen	2022	Prog. Org. Coat.	10.1016/j.porgcoat.2022.107164
ST	B	Pyrrolidine	4	1,4-dioxane	684	Wang	2021	Chem. Commun.	10.1039/d0cc06519f

ST	B	Pyrrolidine	42.7	o-DCB:n-Butylalcohol 9:1	532	Liu	2022	ChemCatChem	10.1002/cctc.202101800
ST	B	Pyrrolidine	4	1,4-dioxane	868	Weng	2022	Nat. Commun.	10.1038/s41467-022-33501-8
ST	B	Pyrrolidine	4	1,4-dioxane	932	Xia	2022	Mater. Today Energy	10.1016/j.mtener.2022.101135
ST	B	Pyrrolidine	4	1,4-dioxane	450	Lv	2023	Water Res.	10.1016/j.watres.2023.119892
ST	B	Pyrrolidine	0.3	o-DCB:n-Butylalcohol 9:1	731	Zhao	2023	Chem. Eng. J.	10.1016/j.cej.2022.140531
ST	B	KOH	3	DMF:Water 2:3	334	Dong	2024	ACS Catal.	10.1021/acscatal.4c04968
ST	B	Pyrrolidine	4.6	1,4-dioxane	275	Weng	2024	J. Am. Chem. Soc.	10.1021/jacs.4c01097
ST	B	Pyrrolidine	4.6	1,4-dioxane	220	Weng	2024	J. Am. Chem. Soc.	10.1021/jacs.4c01097
ST	-	-	-	Ethanol	NR	Ma	2017	The Analyst	10.1039/c7an01027c
ST	-	-	-	Ethanol	578	Ma	2019	Nanoscale	10.1039/c9nr00392d
ST	-	-	-	DCM:chloroform	NR	Shi	2019	Chem. Commun.	10.1039/c9cc07809f
ST	-	-	-	Ethanol	905	Liu	2020	ACS Appl. Mater. Interfaces	10.1021/acsami.0c14486
ST	-	-	-	Mesitylene:1,4-dioxane:water 3:3:1	464	Wang	2020	Ind. Eng. Chem. Res.	10.1021/acs.iecr.0c03797
ST	-	-	-	Water	740	Ma	2022	New J. Chem.	10.1039/d1nj06222k
ST	-	-	-	Ethanol	171	Xu	2022	Chin. J. Chem. Eng.	10.1016/j.cjche.2022.02.014
ST	-	-	-	Choline chloride:urea	295	Talekar	2023	Chem. Eng. J.	10.1016/j.cej.2022.141058
ST	-	-	-	Mesitylene:1,4-dioxane 1:1	NVR	Singh	2024	ACS Food Sci. Technol.	10.1021/acsfoodscitech.4c00565
ST	-	-	-	DMF	443	Yang	2024	Angew. Chem.	10.1002/anie.202404077
ST	-	-	-	Ethanol	157	Zheng	2025	Nat. Commun.	10.1038/s41467-025-57166-1
ST	-	-	-	Ethanol	112	Zheng	2025	Nat. Commun.	10.1038/s41467-025-57166-1
ST, MW	A	Acetic acid	10	Mesitylene:1,4-dioxane 1:1	725	Wei	2015	Chem. Commun.	10.1039/c5cc04680g
ST, MW	A	Acetic acid	10	Mesitylene: dioxane 1:1	1470	Vitaku	2017	J. Am. Chem. Soc.	10.1021/jacs.7b06913
ST, MW	A	Acetic acid	NR	Water	928	Martínez-Visus	2023	Chem. Eur. J.	10.1002/chem.202203907
ST, MW	A	Acetic acid	40	Mesitylene:1,4-dioxane 4:1	925	Yue	2023	Nano Res.	10.1007/s12274-022-5332-0
ST, MW	A	Acetic acid	20	Mesitylene:1,4-dioxane 1:1	NR	Tong	2024	Chem. Eng. J.	10.1016/j.cej.2024.154262

ST, PI	-	-	-	DMF	59	Zhou	2023	Food Chem.	10.1016/j.foodchem.2023.135883
ST, SG	A	Acetic acid	0.5	Mesitylene:1,4-dioxane 1:1	447	Pachfule	2015	Chem. Commun.	10.1039/c5cc04130a
ST, SG	A	Acetic acid	12	Mesitylene:1,4-dioxane 1:1	553	Gou	2016	RSC Adv.	10.1039/c6ra04859e
ST, SG	A	Acetic acid	NR	DCM	1120	Ding	2021	Adv. Funct. Mater.	10.1002/adfm.202106507
ST, SG	A	Acetic acid	NR	DCM	1153	Ding	2021	Adv. Funct. Mater.	10.1002/adfm.202106507
ST, SG	A	Acetic acid	4.4	THF	NR	Guo	2023	Mater. Horiz.	10.1039/d3mh00957b
ST, SG	A	Acetic acid	36	DCM	801	Lin	2024	J. Chromatogr. A	10.1016/j.chroma.2024.464854
ST, SG	A	Octanoic acid	-	Octanoic acid	610	Khan	2020	J. Am. Chem. Soc.	10.1021/jacs.0c04589
MC	A	Acetic acid	5	Mesitylene:1,4-dioxane 1:1	484	Pachfule	2014	Chem. Commun.	10.1039/c3cc49176e
MC	A	benzenesulfonic acid	3	Water	453	Karak	2018	J. Am. Chem. Soc.	10.1021/jacs.7b13560
MC	A	hydrochloric acid	3	Water	20	Karak	2018	J. Am. Chem. Soc.	10.1021/jacs.7b13563
MC	A	nitrobenzenesulfonic acid	3	Water	154	Karak	2018	J. Am. Chem. Soc.	10.1021/jacs.7b13559
MC	A	o-aminobenzenesulfonic acid	3	Water	75	Karak	2018	J. Am. Chem. Soc.	10.1021/jacs.7b13562
MC	A	p-phenolsulfonic acid	3	Water	658	Karak	2018	J. Am. Chem. Soc.	10.1021/jacs.7b13561
MC	A	PTSA	8.3	-	1432	Karak	2017	J. Am. Chem. Soc.	10.1021/jacs.6b08815
MC	A	PTSA	8.3	-	1201	Karak	2017	J. Am. Chem. Soc.	10.1021/jacs.6b08815
MC	A	PTSA	3	Water	832	Karak	2018	J. Am. Chem. Soc.	10.1021/jacs.7b13558
MC	A	PTSA	9	Water	41	Tang	2019	Sep. Purif. Technol.	10.1016/j.seppur.2019.04.069
MC	A	PTSA	8.3	Water	NR	Wang	2019	Anal. Chem.	10.1021/acs.analchem.9b03534
MC	A	PTSA	8.3	-	589	Wang	2019	Inorg. Chem.	10.1021/acs.inorgchem.9b01106
MC	A	PTSA	30	Water	327	Dong	2021	ACS Catal.	10.1021/acscatal.1c03441
MC	A	PTSA	0.2	-	NR	Jiang	2021	Ind. Eng. Chem. Res.	10.1021/acs.iecr.1c02366
MC	A	PTSA	9.2	Water	908	Shang	2021	ACS Sustainable Chem. Eng.	10.1021/acssuschemeng.1c05162
MC	A	PTSA	8.3	Water	161	Dong	2022	Chin. J. Catal.	10.1016/S1872-2067(22)64094-4
MC	A	PTSA	8.3	-	232	Singh	2022	Nano Energy	10.1016/j.nanoen.2021.106690

MC	A	PTSA	8.3	Water	211	Wang	2022	Chem. Eng. J.	10.1016/j.cej.2022.136883
MC	A	PTSA	3.6	DMSO	NR	Jaryal	2023	Mater. Today: Proc.	10.1016/j.matpr.2022.12.093
MC	A	PTSA	8.3	-	960	Martínez-Visus	2023	Chem. Eur. J.	10.1002/chem.202203907
MC	A	PTSA	9.2	Water	1261	Si	2023	J. Alloys Compd.	10.1016/j.jallcom.2023.172218
MC	A	PTSA	7.5	Water	211	Wang	2023	Appl. Surf. Sci.	10.1016/j.apsusc.2023.158383
MC	A	PTSA	7.7	Water	NR	Zhang	2024	J. Colloid Interface Sci.	10.1016/j.jcis.2024.07.161
MC	A	PTSA	8.3	Water	211	Zhang	2024	J. Mater. Chem. A	10.1039/d3ta06724f
MC	A	PTSA	8.3	-	428	Dong	2024	ACS Catal.	10.1021/acscatal.4c04968
MC	-	-	-	-	61	Biswal	2013	J. Am. Chem. Soc.	10.1021/ja4017842
MC	-	-	-	-	NVR	Bu	2021	Inorg. Chem. Front.	10.1039/d1qi00847a
MC	-	-	-	-	NR	Li	2021	Prog. Org. Coat.	10.1016/j.porgcoat.2021.106299
MC	-	-	-	Mesitylene:1,4-dioxane:water 2:2:1	290	Gao	2022	Cell Rep. Phys. Sci.	10.1016/j.xcrp.2022.101153
MC	-	-	-	Mesitylene:1,4-dioxane 1:1	95	Guo	2023	Dalton Trans.	10.1039/d3dt02116e
MC	-	-	-	Mesitylene:1,4-dioxane 1:1	29	Jiang	2025	Small	10.1002/smll.202409079
IS	A	Acetic acid	48	Mesitylene:1,4-dioxane 3:1 and Water:1,4-dioxane 6:1	NR	Chen	2024	Desalination	10.1016/j.desal.2024.117841
IS	A	Octanoic acid		Mestylene and water	45	Wu	2022	J. Membr. Sci.	10.1016/j.memsci.2022.120799
IS	A	Octanoic acid		Mestylene and water:acetonitrile	110	Wu	2022	J. Membr. Sci.	10.1016/j.memsci.2022.120800
IS	A	Octanoic acid		Mestylene and water:acetonitrile	610	Wu	2022	J. Membr. Sci.	10.1016/j.memsci.2022.120801
IS	A	Octanoic acid		Mestylene and water:acetonitrile	433	Wu	2022	J. Membr. Sci.	10.1016/j.memsci.2022.120802
IS	A	Octanoic acid		Mestylene and water:acetonitrile	382	Wu	2022	J. Membr. Sci.	10.1016/j.memsci.2022.120803
IS	A	PTSA	6	n-hexane and water	NR	Wang	2019	J. Membr. Sci.	10.1016/j.memsci.2019.05.082
IS	A	PTSA	NR	Saturated aqueous $Na_2S_2O_3$ solution and DCM and water	NR	Li	2020	Electrochim. Acta	10.1016/j.electacta.2020.137212
IS	-	-	-	Water and DCM	1163	Zhang	2023	J. Am. Chem. Soc.	10.1021/jacs.3c06764
IS	-	-	-	Water and n-hexane	595	Liang	2024	Sep. Purif. Technol.	10.1016/j.seppur.2024.126757
Misc	A	Acetic acid	NR	DMA:water 9:1	632	Singh	2018	NPG Asia Mater.	10.1038/am.2017.209

Misc	A	Acetic acid	290	Water	62	Liu	2023	Nat. Commun.	10.1038/s41467-023-42833-y
Misc	A	Acetic acid	290	Water	96	Liu	2023	Nat. Commun.	10.1038/s41467-023-42833-y
Misc	A	Acetic acid	290	Water	508	Liu	2023	Nat. Commun.	10.1038/s41467-023-42833-y
Misc	A	Acetic acid	290	Water	423	Liu	2023	Nat. Commun.	10.1038/s41467-023-42833-y
NR	NR	NR	NR	NR	569	Yao	2020	J. Mater. Chem. A	10.1039/d0ta02202k
NR	NR	NR	NR	NR	789	Cao	2021	J. Membr. Sci.	10.1016/j.memsci.2021.119319
NR	NR	NR	NR	NR	776	Chen	2021	Res.	doi:10.34133/2021/9798564
NR	NR	NR	NR	NR	326	Li	2021	J. Colloid Interface Sci.	10.1016/j.jcis.2021.01.105
NR	NR	NR	NR	NR	513	Tahir	2023	Electrochim. Acta	10.1016/j.electacta.2023.143127
NR	NR	NR	NR	NR	322	Ma	2024	Adv. Funct. Mater.	10.1002/adfm.202312203
NR	NR	NR	NR	NR	828	Huang	2024	Microchem. J.	10.1016/j.microc.2024.111492

2. General Information

Materials

1,4-Phenylenediamine (>98.0%), 2,4,6-triformylphloroglucinol (>98.0%), and propionic acid (>99.0%) were purchased from TCI Europe N.V. DMF (≥99.8%, HPLC) was purchased from Biosolve. Ethanol (≥99.8%, HPLC), and acetone (≥99.5%, ACS Reagent) were purchased from Honeywell. Mesitylene (99%, extra pure), chloroacetic acid (99%), and formic acid (99%) were purchased from Acros Organics B.V.B.A. Pyridine (99.8%), 2,4,6-trimethylpyridine (99%, puriss. p.a.), DBU (synthesis grade), 1,4-dioxane (≥99.0%, ACS Reagent), and KOH pellets were purchased from Sigma Aldrich. Glacial acetic acid (analytical reagent grade) was purchased from Fischer Scientific. Triethylamine (99%) was purchased from Thermo Scientific. DIPEA (≥99%) was purchased from Carl Roth. All chemicals were used without further purifications.

Instrumentation

ATR-FTIR spectra were obtained on a Bruker Tensor II spectrometer with a Platinum attenuated total reflection (ATR) accessory. The samples were applied as powder on top of the crystal. For each sample 32 scans were recorded with a resolution of 4 cm^{-1} in the range of 400 cm^{-1} to 4000 cm^{-1}.

Nitrogen adsorption-desorption measurements were performed on a MicroActive for Tristar II Plus 2.01 at 77.350 K. Before the measurement, the samples were degassed at 120 °C overnight. Surface areas were calculated from the adsorption data using Brunauer-Emmet-Teller (BET) methods and Rouquerol criteria. More specifically, the maximum in the $n(1\text{-}p/p°)$ vs $p/p°$ plot was chosen as the right boundary of the linearization range. The left boundary was chosen so that the first three Roquerol criteria were met and that the difference between $(1/(\sqrt{C}+1)$ and the $p/p°$ associated with n_m was either less than 10% (in accordance with the 4th Roquerol criterium), or was minimized. The pore-size distribution curves were obtained from the adsorption branches using the method “N2 – Cylindrical Pores – Oxide Surface”. The regularization was generally kept at 0, or an optimum between goodness of fit and smoothness of the pore size distribution was aimed for. This data was also used to determine the micropore volumes.

Scanning Electron Microscopy (SEM) analysis was conducted at room temperature using a JEOL JAMP-9500F field emission scanning Auger microprobe system. SEM images were acquired with a beam energy of 2 keV to optimize resolution and minimize sample damage.

For powder X-ray diffraction (PXRD) analysis samples were thinly applied on a zero-background (553)-silicon wafer. The diffractograms were measured on a Panalytical Empyrean diffractometer in Bragg-Brentano geometry using CuKα radiation from a sealed LFF tube (operating voltage of 45kV, current 40mA) and a PIXcel3D 1×1 detector. A continuous scan was made in the $2° < 2\theta < 45°$ range with a step size of 0.013°.

Origin2020b (64-bit) version 9.7.5.184 was used to analyze, fit and plot all data.

3. Synthetic Procedures

TpPa COF:

The procedure is based on previous imine work within our group[S1] and adapted by the use of **Tp** and the separate dissolution of **Tp** and **Pa**.

1,4-Phenylenediamine (**Pa**, 125 mg, 1.16 mmol, 1.5 equivalents) was added to a 50 mL round-bottom flask and dissolved in 3.0 mL 1,4-dioxane:mesitylene 4:1 v/v while heated to 70 °C. 1.2 mL water (86.5 equivalents) and the acid or base (⅓, 1, 2, 10, 20 or 40 equivalents) were added to the solution (see Table **S2** for the exact quantities of used additive). In parallel, 2,4,6-triformylphloroglucinol (**Tp**, 162 mg, 0.771 mmol, 1 equivalent) was added to a small beaker and dissolved in 4.0 mL of 1,4-dioxane:mesitylene 4:1 v/v under heating. The **Tp** solution was poured into the **Pa** solution, a condenser was affixed to the set-up and the mixture was heated to 70 °C under stirring for 3 days under an ambient atmosphere and atmospheric pressure.

The precipitate was collected by Büchner filtration and the flask rinsed with DMF. The solids were dispersed in 125 mL DMF and heated to 90 °C under stirring for 30 minutes, after which the solids were collected by Büchner filtration. This was repeated for DMF, ethanol and acetone. Finally, the solids were dried overnight in an oven at 120 °C.

Table S2: Overview of used quantities of the indicated additive (acid or base) at the specified equivalence in the synthesis of the **TpPa** frameworks.

**Equivalent**	**⅓**	**1**	**2**	**10**	**20**	**40**
Chloroacetic acid (mg)	24.0	72.9	145.7	728.6	1457.2	2914.4
Formic acid (µL)	9.6	29.1	58.2	291	582	1164
Acetic acid (µL)	14.6	44.1	88.2	441	882	1764
Propionic acid (µL)	19.0	57.7	115.4	577	1154	2308
Pyridine (µL)	20.5	62.1	124.2	621	1242	2484
Trimethylpyridine (µL)	33.8	102.3	205	1023	2047	4093
Triethylamine (µL)	35.5	107.5	215	1075	2149	4299
Diisopropylethylamine (µL)	44.3	134.3	269	1343	2686	5372
DBU (µL)	38.0	115.3	231	1153	2306	4612
KOH (mg)	14.3	43.3	86.5	432.6	865.1	1730.3

4. ATR-FTIR spectra

4.1 Chloroacetic acid

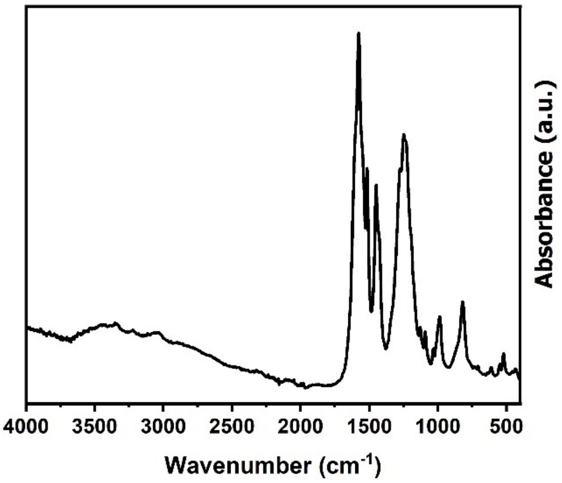

Figure S1: ATR-FTIR spectrum of **TpPa** synthesized with ⅓ equivalents of chloroacetic acid. The keto C=O stretch is at 1601 cm^{-1}, the keto C=C stretch is at 1581 cm^{-1} and the enamine C–N stretch is at 1252 cm^{-1}.

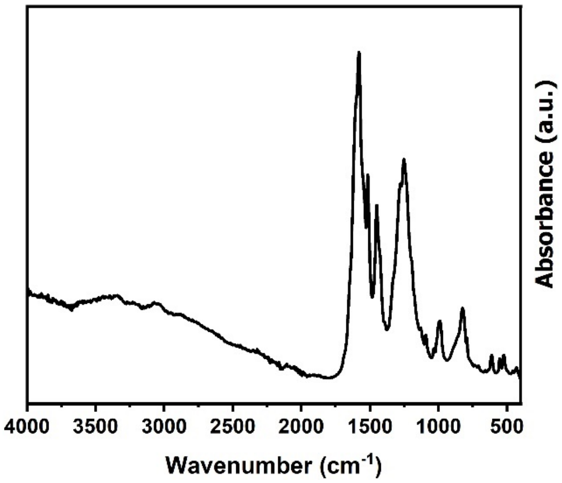

Figure S2: ATR-FTIR spectrum of **TpPa** synthesized with 1 equivalent of chloroacetic acid. The keto C=O stretch is at 1605 cm^{-1}, the keto C=C stretch is at 1585 cm^{-1} and the enamine C–N stretch is at 1255 cm^{-1}.

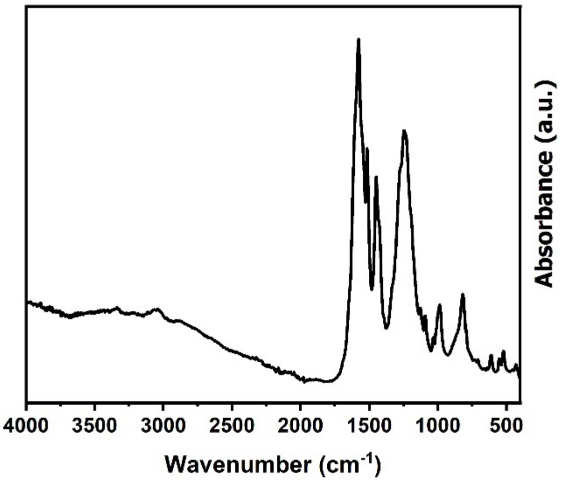

Figure S3: ATR-FTIR spectrum of **TpPa** synthesized with 2 equivalents of chloroacetic acid. The keto C=O stretch is at 1603 cm^{-1}, the keto C=C stretch is at 1579 cm^{-1} and the enamine C–N stretch is at 1252 cm^{-1}.

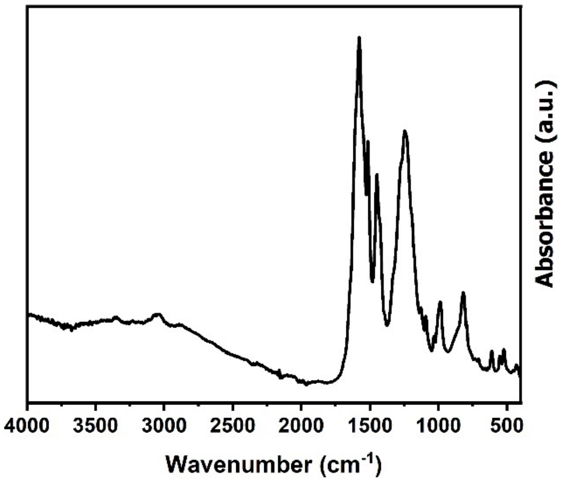

Figure S4: ATR-FTIR spectrum of **TpPa** synthesized with 10 equivalents of chloroacetic acid. The keto C=O stretch is at 1603 cm^{-1}, the keto C=C stretch is at 1578 cm^{-1} and the enamine C–N stretch is at 1250 cm^{-1}.

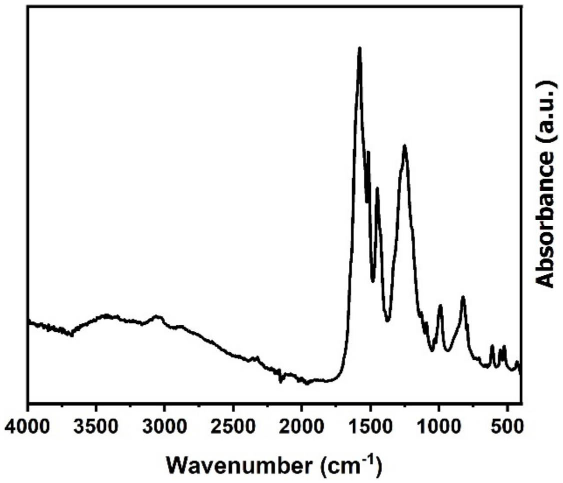

Figure S5: ATR-FTIR spectrum of **TpPa** synthesized with 20 equivalents of chloroacetic acid. The keto C=O stretch is at 1603 cm^{-1}, the keto C=C stretch is at 1579 cm^{-1} and the enamine C–N stretch is at 1254 cm^{-1}.

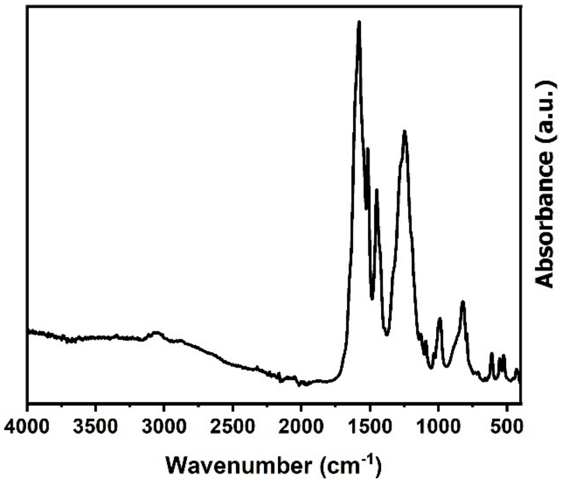

Figure S6: ATR-FTIR spectrum of **TpPa** synthesized with 40 equivalents of chloroacetic acid. The keto C=O stretch is at 1607 cm^{-1}, the keto C=C stretch is at 1578 cm^{-1} and the enamine C–N stretch is at 1255 cm^{-1}.

4.2 Formic acid

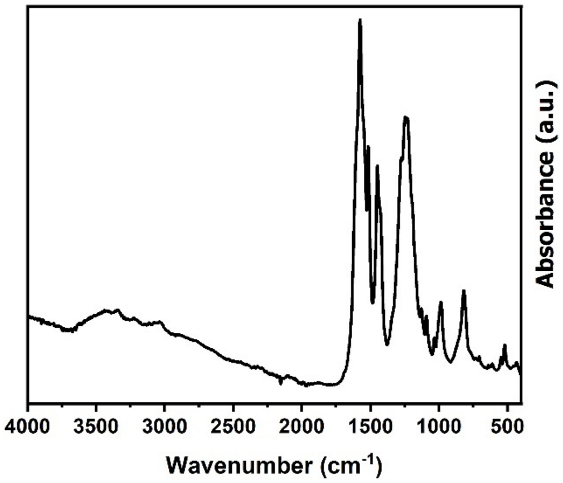

Figure S7: ATR-FTIR spectrum of **TpPa** synthesized with ⅓ equivalents of formic acid. The keto C=O stretch is at 1605 cm^{-1}, the keto C=C stretch is at 1579 cm^{-1} and the enamine C–N stretch is at 1252 cm^{-1}.

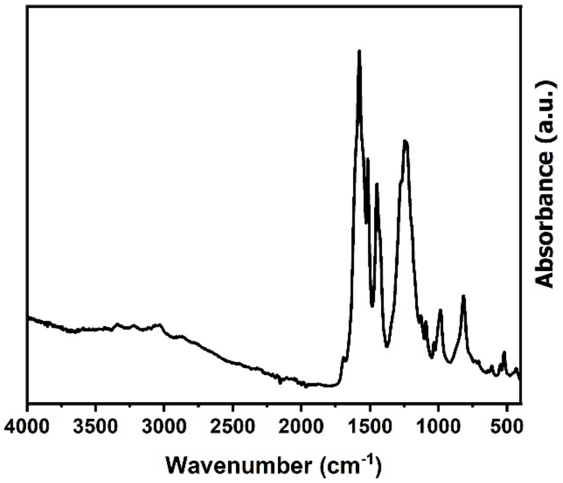

Figure S8: ATR-FTIR spectrum of **TpPa** synthesized with 1 equivalent of formic acid. The keto C=O stretch is at 1605 cm^{-1}, the keto C=C stretch is at 1579 cm^{-1} and the enamine C–N stretch is at 1252 cm^{-1}.

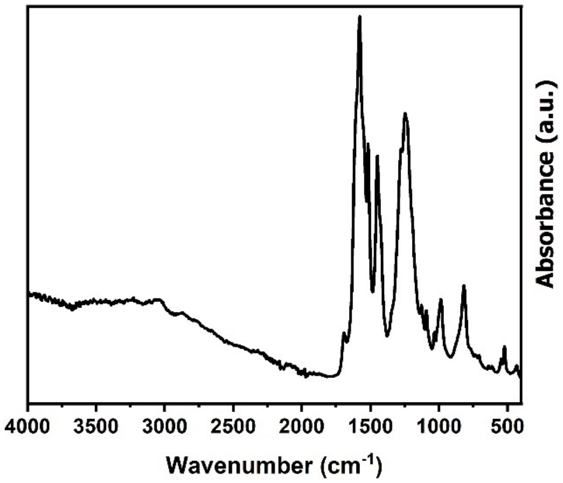

Figure S9: ATR-FTIR spectrum of **TpPa** synthesized with 2 equivalents of formic acid. The keto C=O stretch is at 1605 cm^{-1}, the keto C=C stretch is at 1578 cm^{-1} and the enamine C–N stretch is at 1248 cm^{-1}.

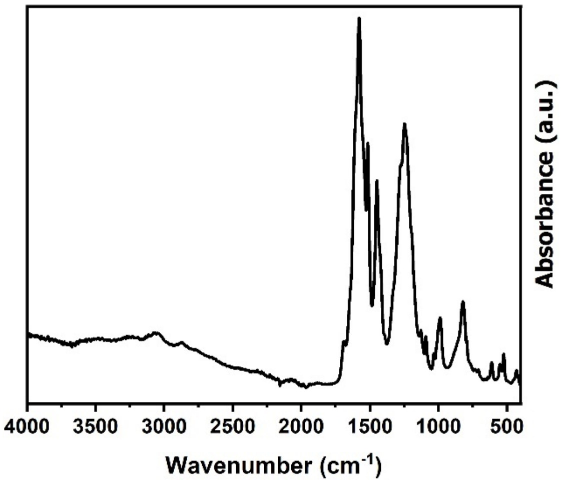

Figure S10: ATR-FTIR spectrum of **TpPa** synthesized with 10 equivalents of formic acid. The keto C=O stretch is at 1603 cm^{-1}, the keto C=C stretch is at 1579 cm^{-1} and the enamine C–N stretch is at 1252 cm^{-1}.

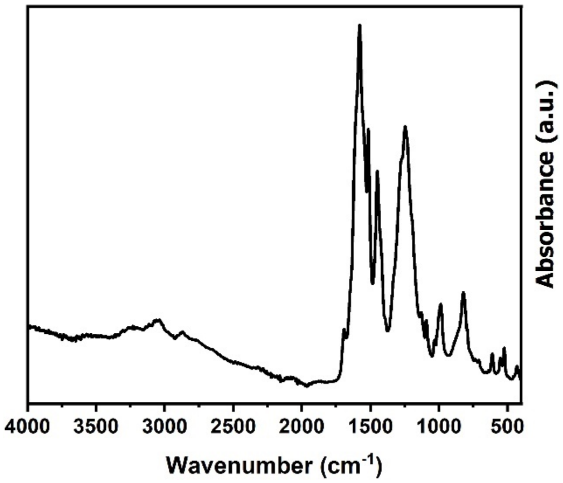

Figure S11: ATR-FTIR spectrum of **TpPa** synthesized with 20 equivalents of formic acid. The keto C=O stretch is at 1605 cm^{-1}, the keto C=C stretch is at 1583 cm^{-1} and the enamine C–N stretch is at 1252 cm^{-1}.

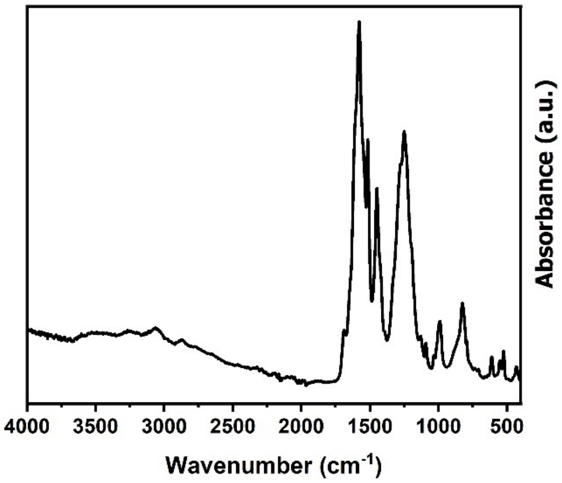

Figure S12: ATR-FTIR spectrum of **TpPa** synthesized with 40 equivalents of formic acid. The keto C=O stretch is at 1605 cm^{-1}, the keto C=C stretch is at 1578 cm^{-1} and the enamine C–N stretch is at 1252 cm^{-1}.

4.3 Acetic acid

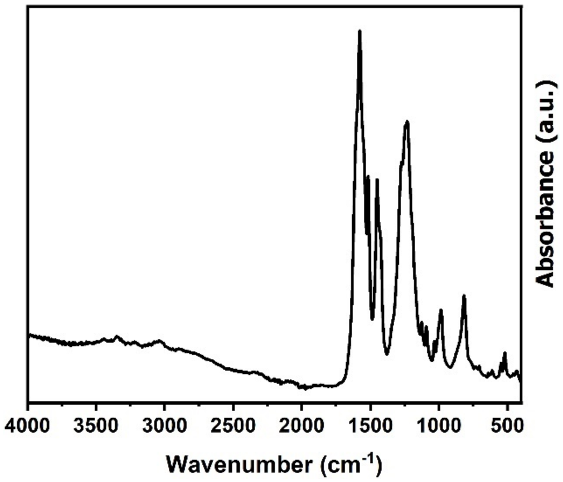

Figure S13: ATR-FTIR spectrum of **TpPa** synthesized with ⅓ equivalents of acetic acid. The keto C=O stretch is at 1601 cm^{-1}, the keto C=C stretch is at 1578 cm^{-1} and the enamine C–N stretch is at 1250 cm^{-1}.

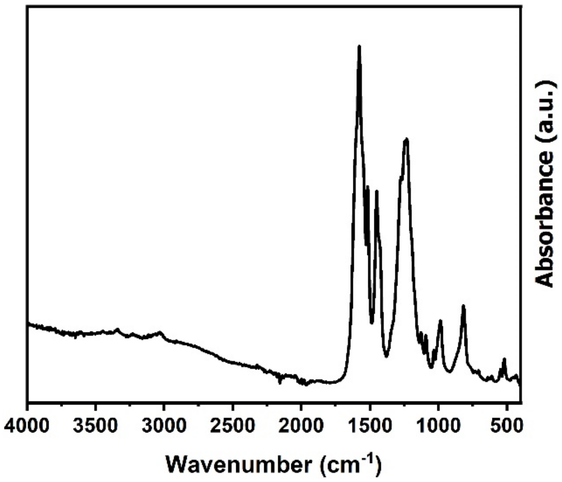

Figure S14: ATR-FTIR spectrum of **TpPa** synthesized with 1 equivalent of acetic acid. The keto C=O stretch is at 1601 cm^{-1}, the keto C=C stretch is at 1578 cm^{-1} and the enamine C–N stretch is at 1248 cm^{-1}.

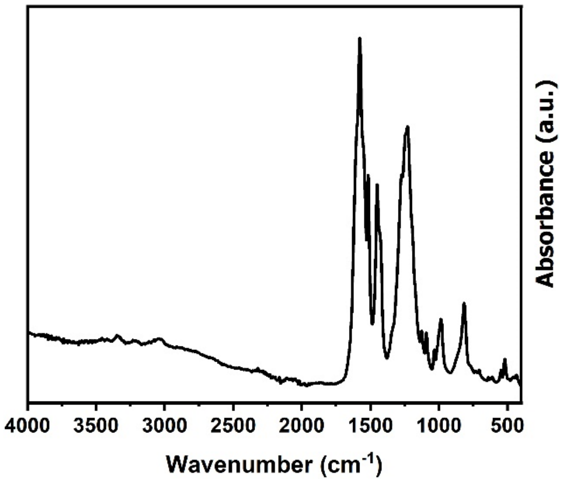

Figure S15: ATR-FTIR spectrum of **TpPa** synthesized with 2 equivalents of acetic acid. The keto C=O stretch is at 1601 cm^{-1}, the keto C=C stretch is at 1578 cm^{-1} and the enamine C–N stretch is at 1248 cm^{-1}.

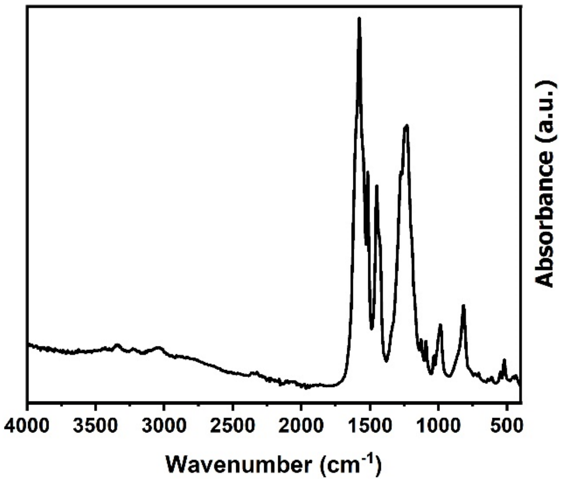

Figure S16: ATR-FTIR spectrum of **TpPa** synthesized with 10 equivalents of acetic acid. The keto C=O stretch is at 1603 cm^{-1}, the keto C=C stretch is at 1578 cm^{-1} and the enamine C–N stretch is at 1252 cm^{-1}.

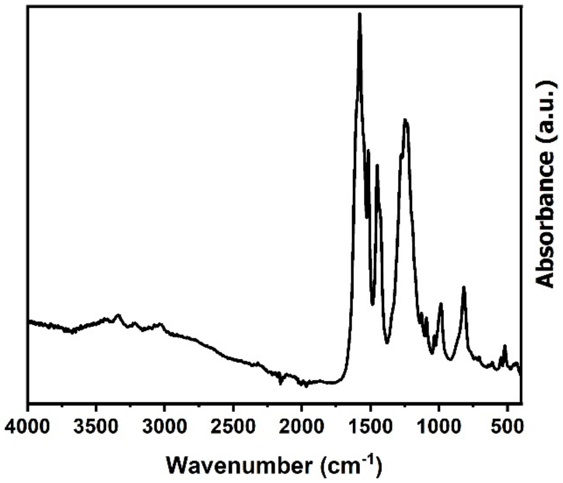

Figure S17: ATR-FTIR spectrum of **TpPa** synthesized with 20 equivalents of acetic acid. The keto C=O stretch is at 1603 cm^{-1}, the keto C=C stretch is at 1576 cm^{-1} and the enamine C–N stretch is at 1255 cm^{-1}.

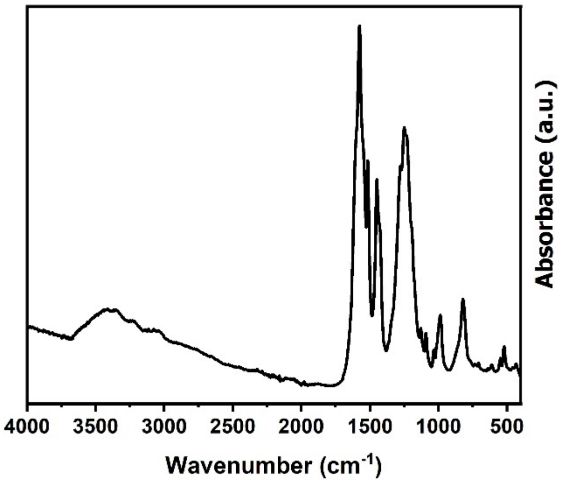

Figure S18: ATR-FTIR spectrum of **TpPa** synthesized with 40 equivalents of acetic acid. The keto C=O stretch is at 1601 cm^{-1}, the keto C=C stretch is at 1576 cm^{-1} and the enamine C–N stretch is at 1250 cm^{-1}.

4.4 Propionic acid

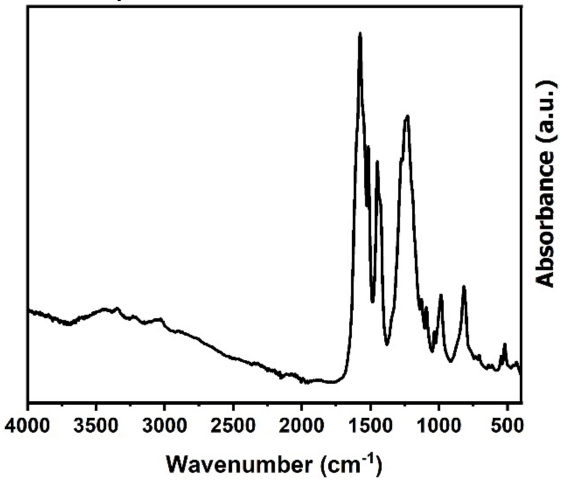

Figure S19: ATR-FTIR spectrum of **TpPa** synthesized with ⅓ equivalents of propionic acid. The keto C=O stretch is at 1602 cm^{-1}, the keto C=C stretch is at 1578 cm^{-1} and the enamine C–N stretch is at 1250 cm^{-1}.

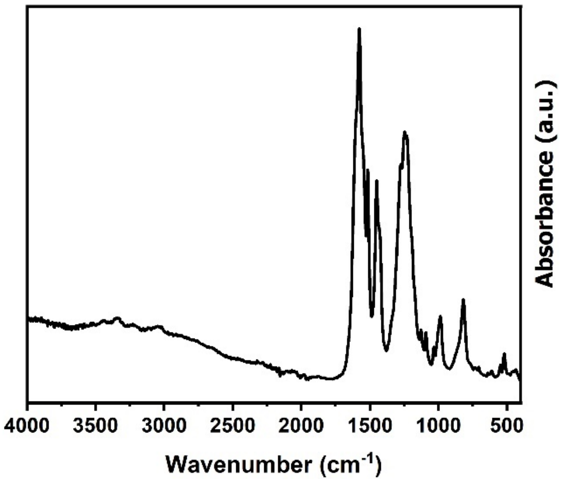

Figure S20: ATR-FTIR spectrum of **TpPa** synthesized with 1 equivalent of propionic acid. The keto C=O stretch is at 1601 cm^{-1}, the keto C=C stretch is at 1579 cm^{-1} and the enamine C–N stretch is at 1254 cm^{-1}.

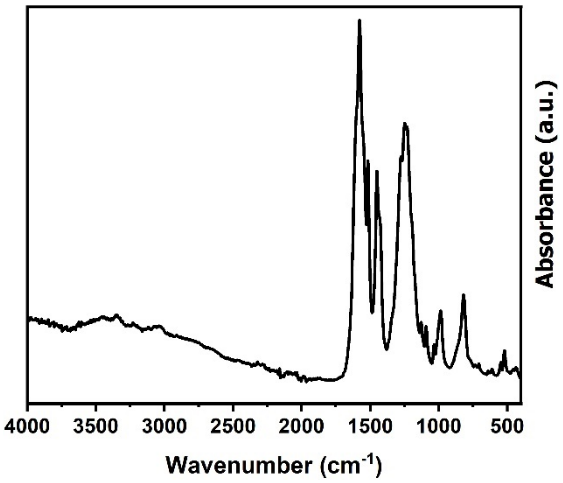

Figure S21: ATR-FTIR spectrum of **TpPa** synthesized with 2 equivalents of propionic acid. The keto C=O stretch is at 1601 cm^{-1}, the keto C=C stretch is at 1576 cm^{-1} and the enamine C–N stretch is at 1248 cm^{-1}.

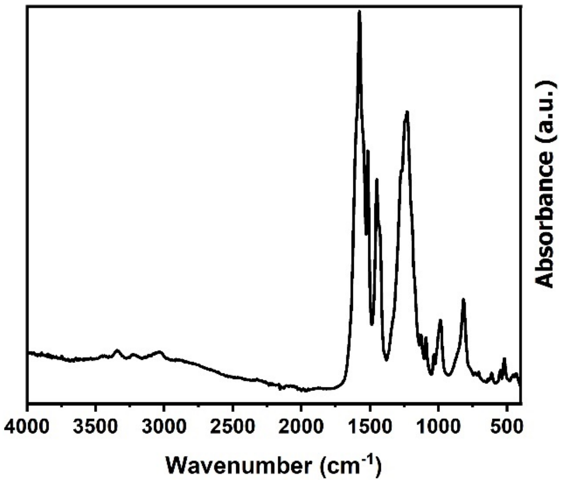

Figure S22: ATR-FTIR spectrum of **TpPa** synthesized with 10 equivalents of propionic acid. The keto C=O stretch is at 1601 cm^{-1}, the keto C=C stretch is at 1578 cm^{-1} and the enamine C–N stretch is at 1250 cm^{-1}.

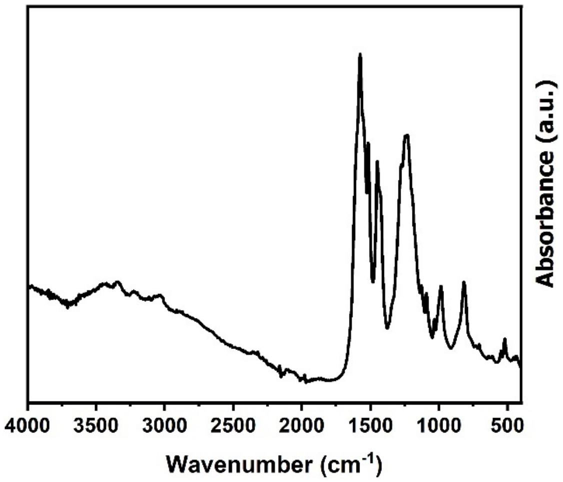

Figure S23: ATR-FTIR spectrum of **TpPa** synthesized with 20 equivalents of propionic acid. The keto C=O stretch is at 1601 cm^{-1}, the keto C=C stretch is at 1579 cm^{-1} and the enamine C–N stretch is at 1250 cm^{-1}.

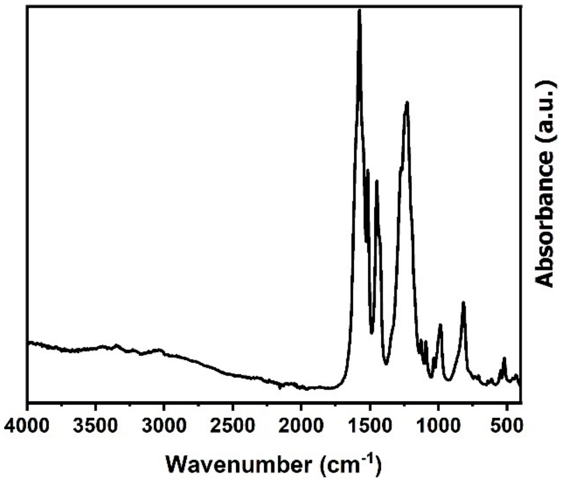

Figure S24: ATR-FTIR spectrum of **TpPa** synthesized with 40 equivalents of propionic acid. The keto C=O stretch is at 603 cm^{-1}, the keto C=C stretch is at 1578 cm^{-1} and the enamine C–N stretch is at 1255 cm^{-1}.

4.5 No acid/base

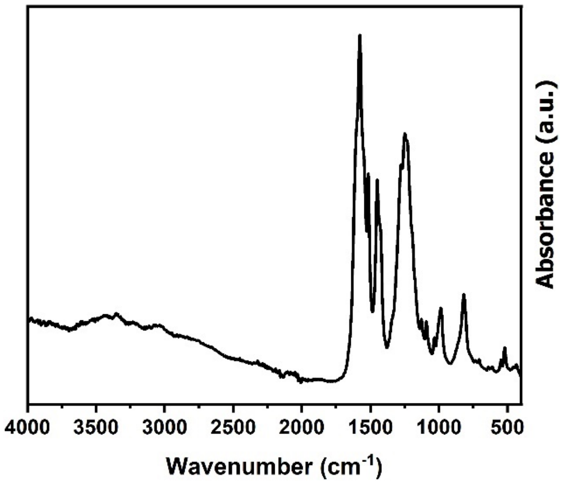

Figure S25: ATR-FTIR spectrum of **TpPa** synthesized without acid or base, sample 1 of 6. The keto C=O stretch is at 1605 cm^{-1}, the keto C=C stretch is at 1579 cm^{-1} and the enamine C–N stretch is at 1254 cm^{-1}.

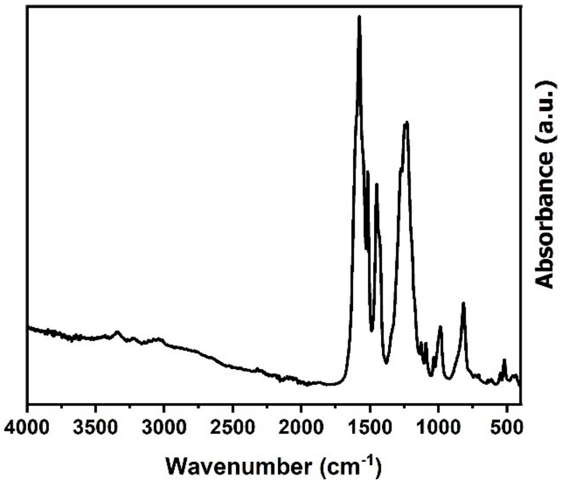

Figure S26: ATR-FTIR spectrum of **TpPa** synthesized without acid or base, sample 2 of 6. The keto C=O stretch is at 1600 cm^{-1}, the keto C=C stretch is at 1573 cm^{-1} and the enamine C–N stretch is at 1249 cm^{-1}.

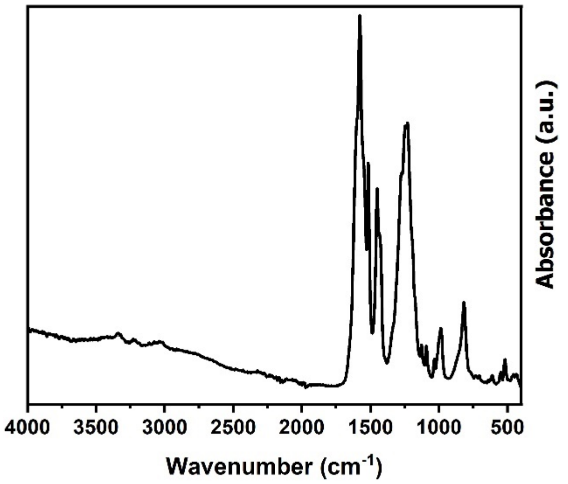

Figure S27: ATR-FTIR spectrum of **TpPa** synthesized without acid or base, sample 3 of 6. The keto C=O stretch is at 1599 cm^{-1}, the keto C=C stretch is at 1575 cm^{-1} and the enamine C–N stretch is at 1252 cm^{-1}.

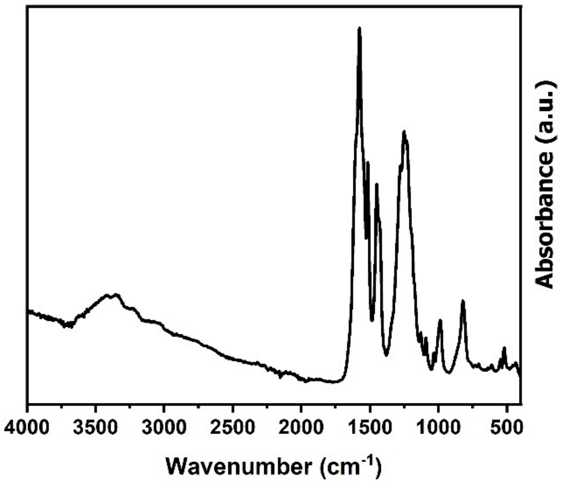

Figure S28: ATR-FTIR spectrum of **TpPa** synthesized without acid or base, sample 4 of 6. The keto C=O stretch is at 1600 cm^{-1}, the keto C=C stretch is at 1575 cm^{-1} and the enamine C–N stretch is at 1246 cm^{-1}.

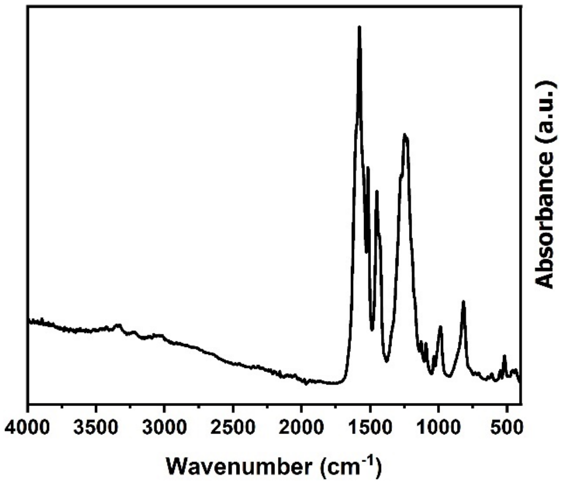

Figure S29: ATR-FTIR spectrum of **TpPa** without acid or base, sample 5 of 6. The keto C=O stretch is at 1596 cm^{-1}, the keto C=C stretch is at 1575 cm^{-1} and the enamine C–N stretch is at 1246 cm^{-1}.

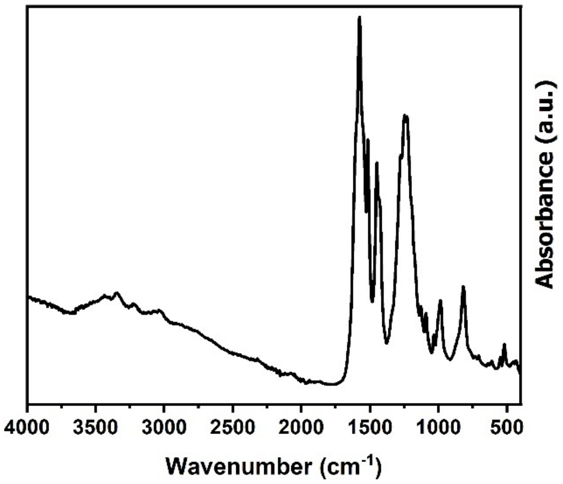

Figure S30: ATR-FTIR spectrum of **TpPa** synthesized without acid or base, sample 6 of 6. The keto C=O stretch is at 1597 cm^{-1}, the keto C=C stretch is at 1578 cm^{-1} and the enamine C–N stretch is at 1246 cm^{-1}.

4.6 Pyridine

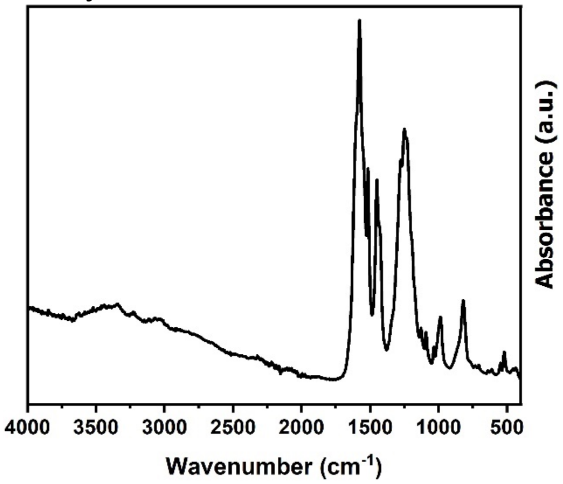

Figure S31: ATR-FTIR spectrum of **TpPa** synthesized with ⅓ equivalents of pyridine. The keto C=O stretch is at 1599 cm^{-1}, the keto C=C stretch is at 1575 cm^{-1} and the enamine C–N stretch is at 1251 cm^{-1}.

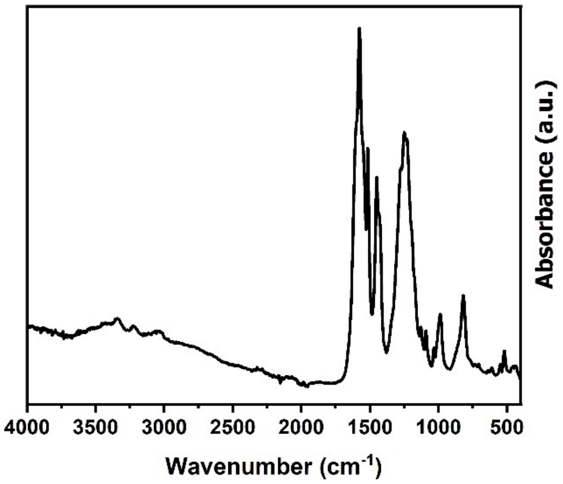

Figure S32: ATR-FTIR spectrum of **TpPa** synthesized with 1 equivalent of pyridine. The keto C=O stretch is at 1599 cm^{-1}, the keto C=C stretch is at 1576 cm^{-1} and the enamine C–N stretch is at 1248 cm^{-1}.

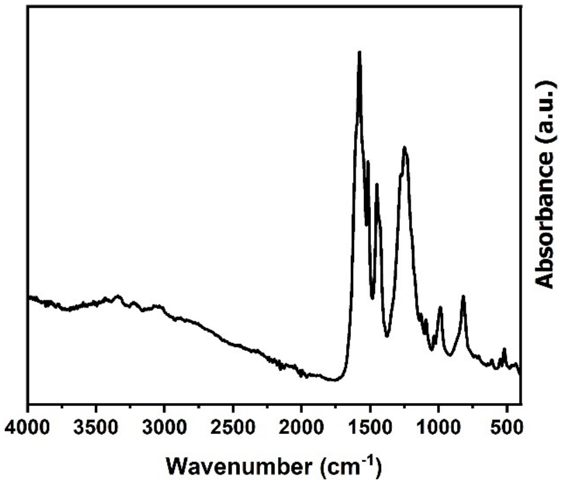

Figure S33: ATR-FTIR spectrum of **TpPa** synthesized with 2 equivalents of pyridine. The keto C=O stretch is at 1599 cm^{-1}, the keto C=C stretch is at 1573 cm^{-1} and the enamine C–N stretch is at 1251 cm^{-1}.

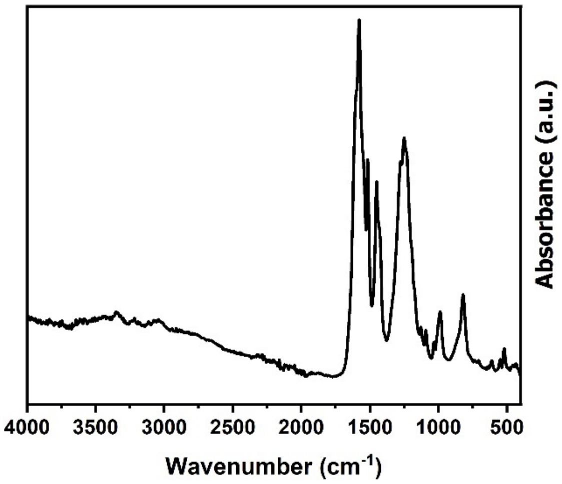

Figure S34: ATR-FTIR spectrum of **TpPa** synthesized with 10 equivalents of pyridine. The keto C=O stretch is at 1599 cm^{-1}, the keto C=C stretch is at 1575 cm^{-1} and the enamine C–N stretch is at 1248 cm^{-1}.

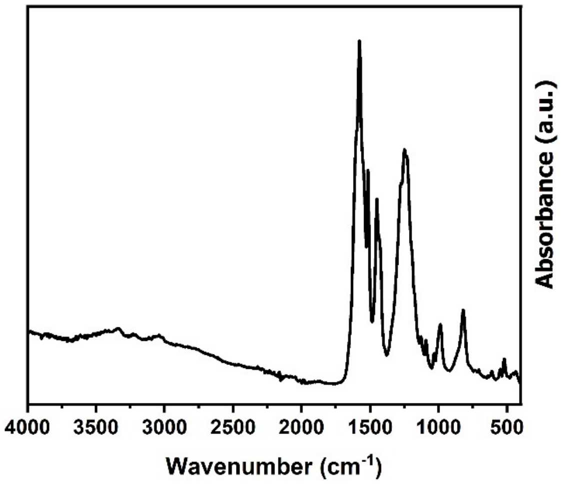

Figure S35: ATR-FTIR spectrum of **TpPa** synthesized with 20 equivalents of pyridine. The keto C=O stretch is at 1599 cm^{-1}, the keto C=C stretch is at 1576 cm^{-1} and the enamine C–N stretch is at 1248 cm^{-1}.

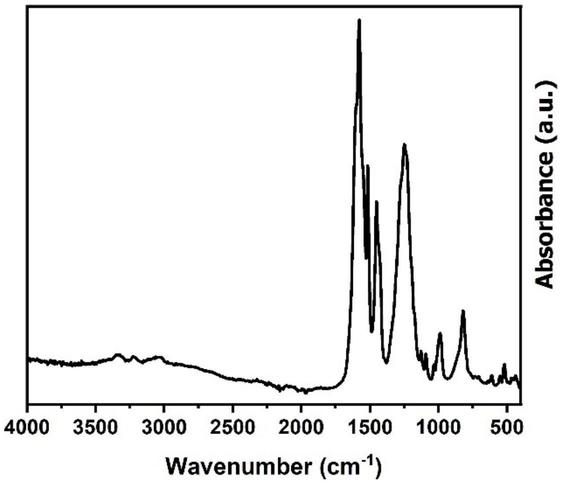

Figure S36: ATR-FTIR spectrum of **TpPa** synthesized with 40 equivalents of pyridine. The keto C=O stretch is at 1598 cm^{-1}, the keto C=C stretch is at 1576 cm^{-1} and the enamine C–N stretch is at 1251 cm^{-1}.

4.7 2,4,6-trimethylpyridine

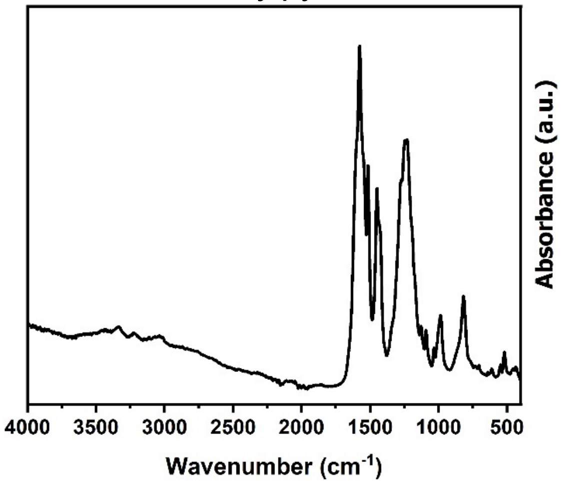

Figure S37: ATR-FTIR spectrum of **TpPa** synthesized with ⅓ equivalents of 2,4,6-trimethylpyridine. The keto C=O stretch is at 1600 cm^{-1}, the keto C=C stretch is at 1573 cm^{-1} and the enamine C–N stretch is at 1252 cm^{-1}.

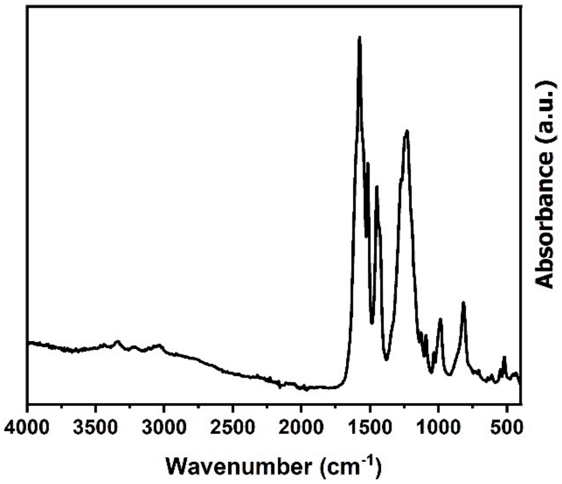

Figure S38: ATR-FTIR spectrum of **TpPa** synthesized with 1 equivalent of 2,4,6-trimethylpyridine. The keto C=O stretch is at 1600 cm^{-1}, the keto C=C stretch is at 1576 cm^{-1} and the enamine C–N stretch is at 1251 cm^{-1}.

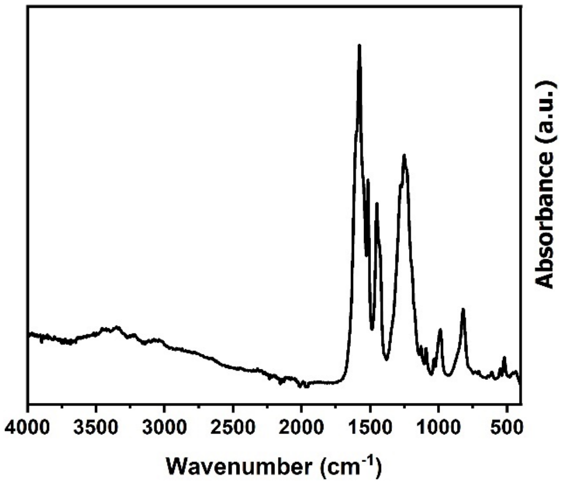

Figure S39: ATR-FTIR spectrum of **TpPa** synthesized with 2 equivalents of 2,4,6-trimethylpyridine. The keto C=O stretch is at 1597 cm^{-1}, the keto C=C stretch is at 1575 cm^{-1} and the enamine C–N stretch is at 1252 cm^{-1}.

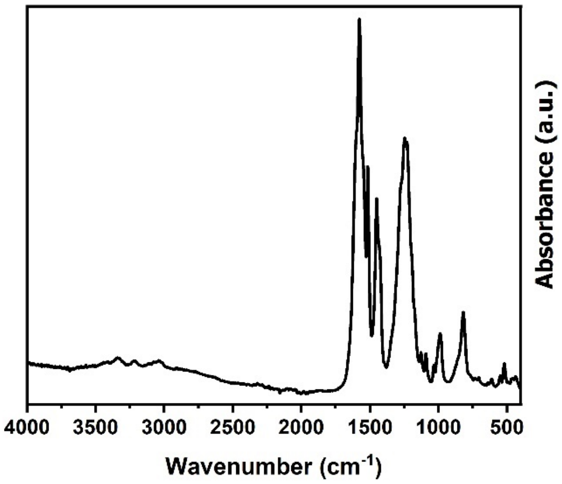

Figure S40: ATR-FTIR spectrum of **TpPa** synthesized with 10 equivalents of 2,4,6-trimethylpyridine. The keto C=O stretch is at 1599 cm^{-1}, the keto C=C stretch is at 1575 cm^{-1} and the enamine C–N stretch is at 1251 cm^{-1}.

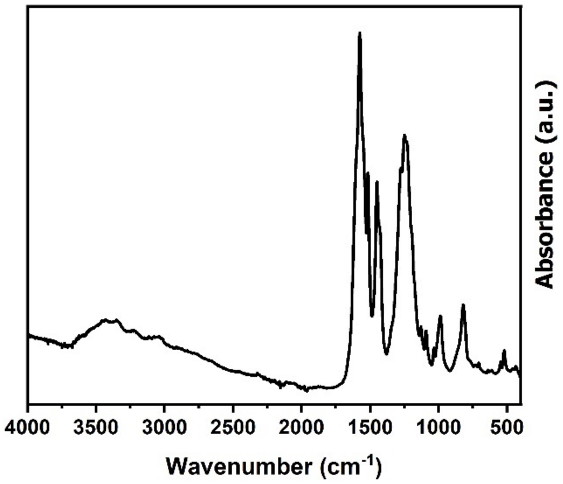

Figure S41: ATR-FTIR spectrum of **TpPa** synthesized with 20 equivalents of 2,4,6-trimethylpyridine. The keto C=O stretch is at 1599 cm^{-1}, the keto C=C stretch is at 1575 cm^{-1} and the enamine C–N stretch is at 1248 cm^{-1}.

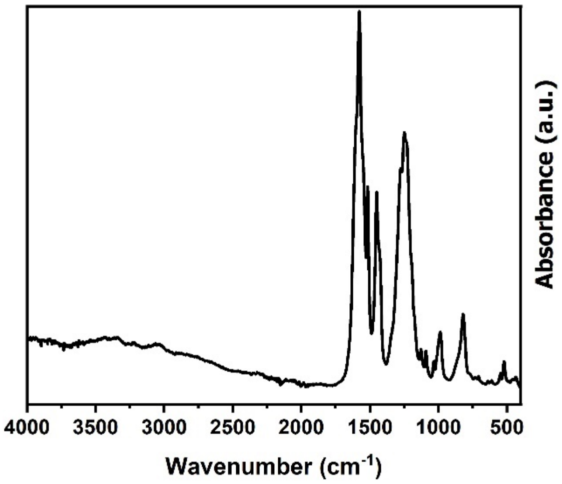

Figure S42: ATR-FTIR spectrum of **TpPa** synthesized with 40 equivalents of 2,4,6-trimethylpyridine. The keto C=O stretch is at 1600 cm^{-1}, the keto C=C stretch is at 1575 cm^{-1} and the enamine C–N stretch is at 1251 cm^{-1}.

4.8 TEA

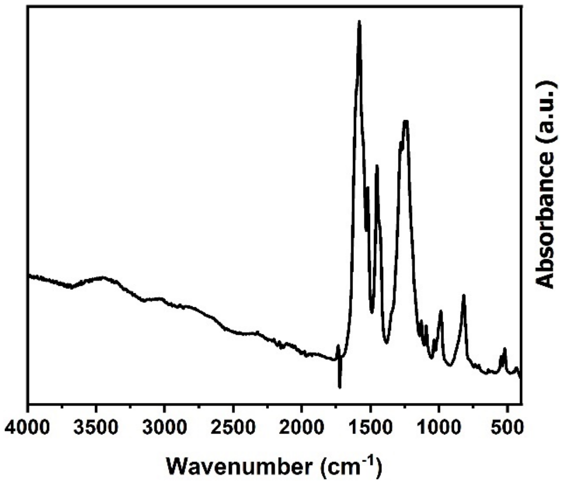

Figure S43: ATR-FTIR spectrum of **TpPa** synthesized with ⅓ equivalents of TEA. The keto C=O stretch is at 1601 cm^{-1}, the keto C=C stretch is at 1579 cm^{-1} and the enamine C–N stretch is at 1252 cm^{-1}.

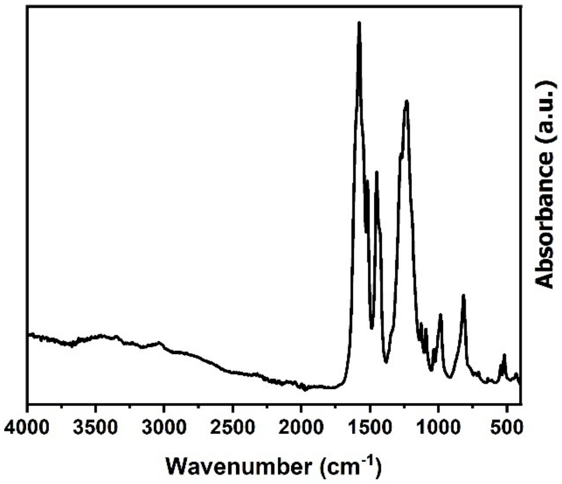

Figure S44: ATR-FTIR spectrum of **TpPa** synthesized with ⅓ equivalents of TEA (duplicate synthesis). The keto C=O stretch is at 1600 cm^{-1}, the keto C=C stretch is at 1573 cm^{-1} and the enamine C–N stretch is at 1251 cm^{-1}.

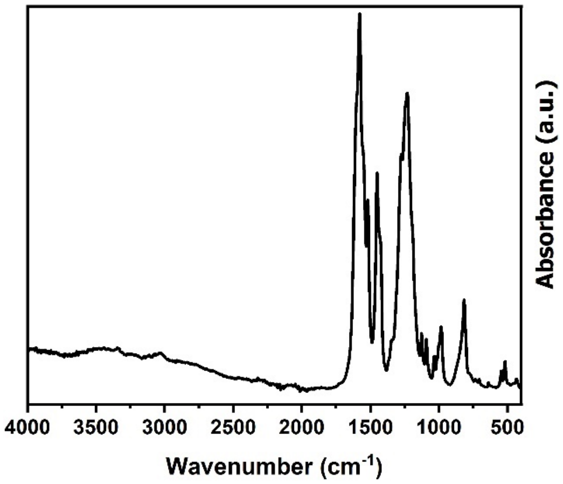

Figure S45: ATR-FTIR spectrum of **TpPa** synthesized with 1 equivalent of TEA. The keto C=O stretch is at 1599 cm^{-1}, the keto C=C stretch is at 1579 cm^{-1} and the enamine C–N stretch is at 1248 cm^{-1}.

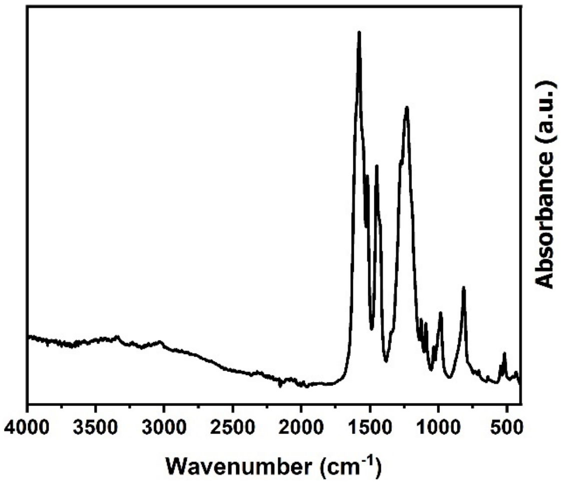

Figure S46: ATR-FTIR spectrum of **TpPa** synthesized with 1 equivalent of TEA (duplicate synthesis). The keto C=O stretch is at 1600 cm^{-1}, the keto C=C stretch is at 1579 cm^{-1} and the enamine C–N stretch is at 1246 cm^{-1}.

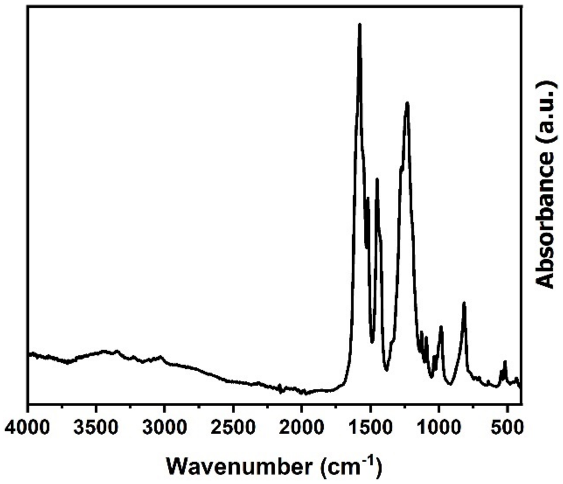

Figure S47: ATR-FTIR spectrum of **TpPa** synthesized with 2 equivalents of TEA. The keto C=O stretch is at 1601 cm^{-1}, the keto C=C stretch is at 1581 cm^{-1} and the enamine C–N stretch is at 1246 cm^{-1}.

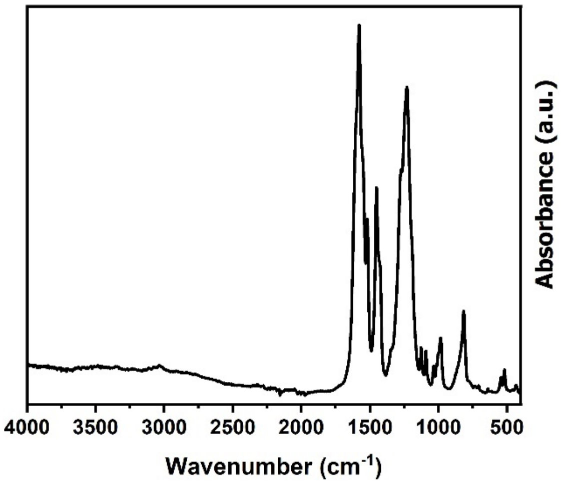

Figure S48: ATR-FTIR spectrum of **TpPa** synthesized with 2 equivalents of TEA (duplicate synthesis). The keto C=O stretch is at 1602 cm^{-1}, the keto C=C stretch is at 1573 cm^{-1} and the enamine C–N stretch is at 1256 cm^{-1}.

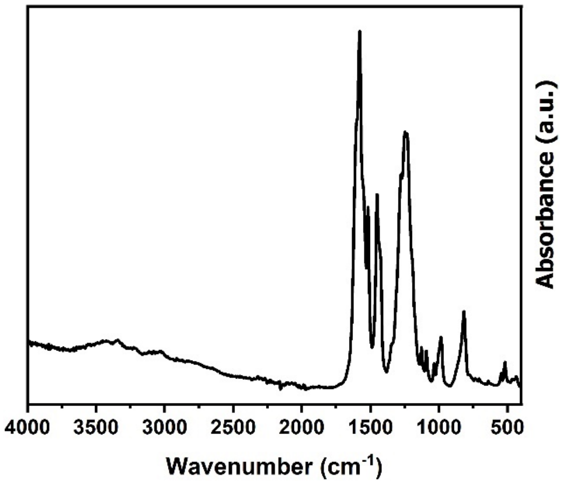

Figure S49: ATR-FTIR spectrum of **TpPa** synthesized with 10 equivalents of TEA. The keto C=O stretch is at 1600 cm^{-1}, the keto C=C stretch is at 1576 cm^{-1} and the enamine C–N stretch is at 1246 cm^{-1}.

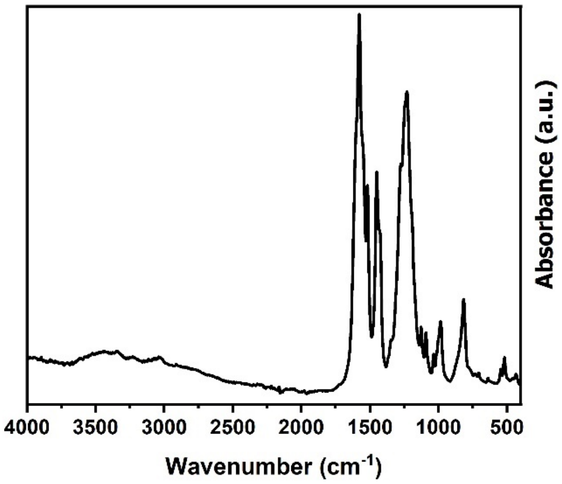

Figure S50: ATR-FTIR spectrum of **TpPa** synthesized with 10 equivalents of TEA (duplicate synthesis). The keto C=O stretch is at 1599 cm^{-1}, the keto C=C stretch is at 1573 cm^{-1} and the enamine C–N stretch is at 1248 cm^{-1}.

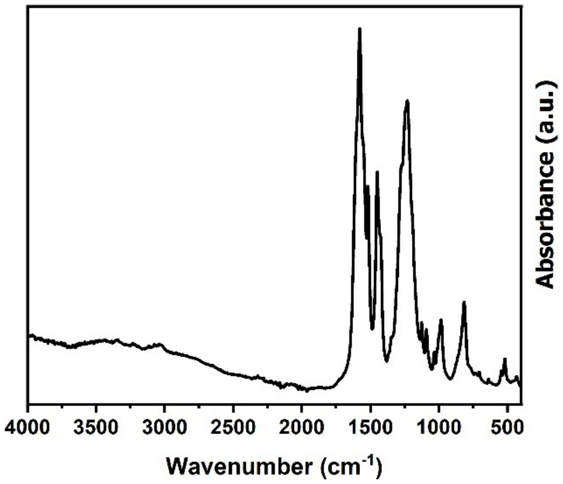

Figure S51: ATR-FTIR spectrum of **TpPa** synthesized with 20 equivalents of TEA. The keto C=O stretch is at 1600 cm^{-1}, the keto C=C stretch is at 1573 cm^{-1} and the enamine C–N stretch is at 1251 cm^{-1}.

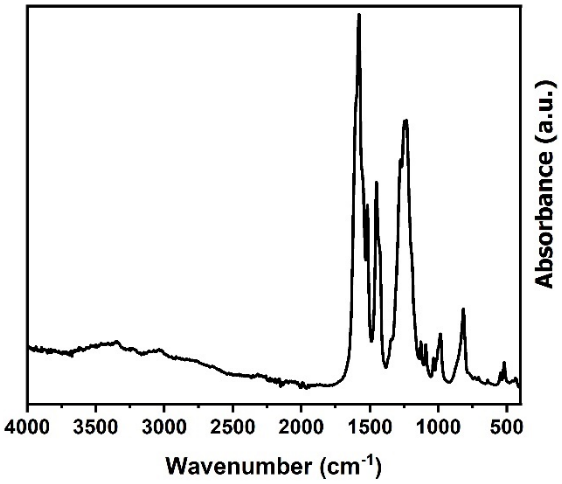

Figure S52: ATR-FTIR spectrum of **TpPa** synthesized with 20 equivalents of TEA (duplicate synthesis). The keto C=O stretch is at 1600 cm^{-1}, the keto C=C stretch is at 1573 cm^{-1} and the enamine C–N stretch is at 1249 cm^{-1}.

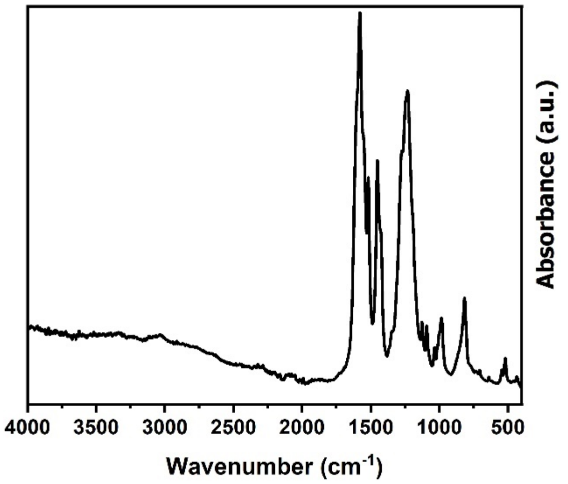

Figure S53: ATR-FTIR spectrum of **TpPa** synthesized with 40 equivalents of TEA. The keto C=O stretch is at 1601 cm^{-1}, the keto C=C stretch is at 1579 cm^{-1} and the enamine C–N stretch is at 1248 cm^{-1}.

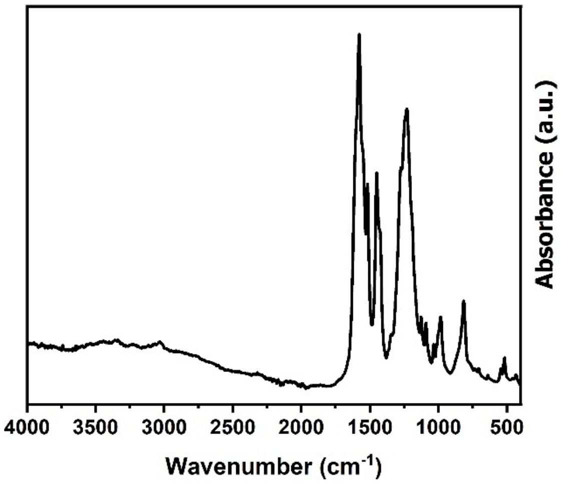

Figure S54: ATR-FTIR spectrum of **TpPa** synthesized with 40 equivalents of TEA (duplicate synthesis). The keto C=O stretch is at 1600 cm^{-1}, the keto C=C stretch is at 1576 cm^{-1} and the enamine C–N stretch is at 1249 cm^{-1}.

4.9 DIPEA

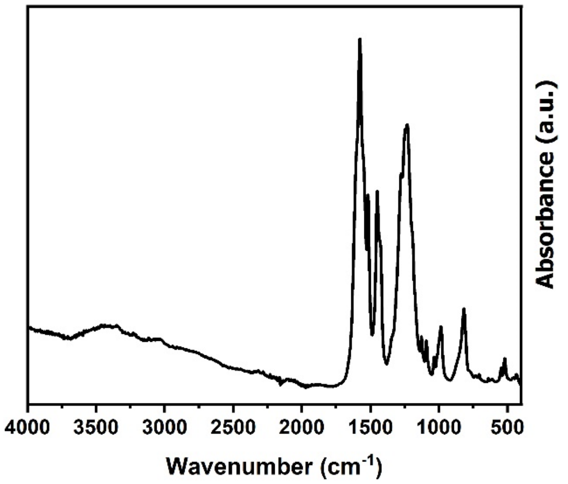

Figure S55: ATR-FTIR spectrum of **TpPa** synthesized with ⅓ equivalents of DIPEA. The keto C=O stretch is at 1600 cm^{-1}, the keto C=C stretch is at 1576 cm^{-1} and the enamine C–N stretch is at 1245 cm^{-1}.

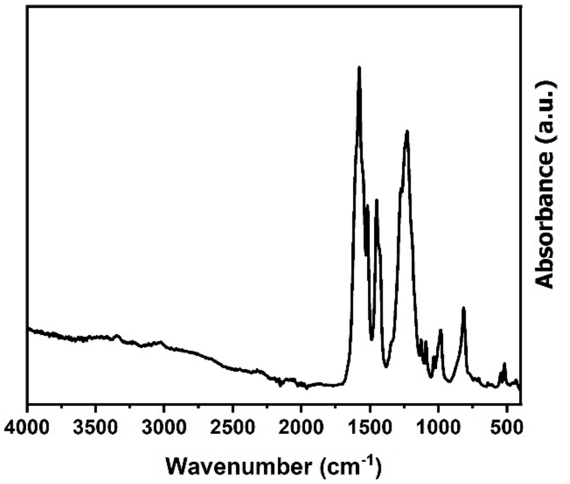

Figure S56: ATR-FTIR spectrum of **TpPa** synthesized with ⅓ equivalents of DIPEA (duplicate synthesis). The keto C=O stretch is at 1599 cm^{-1}, the keto C=C stretch is at 1578 cm^{-1} and the enamine C–N stretch is at 1245 cm^{-1}.

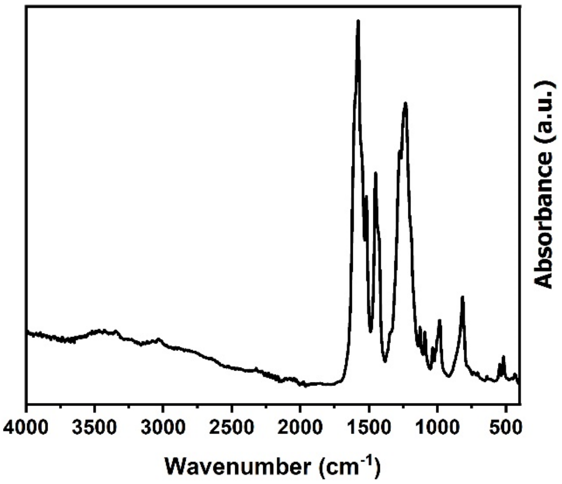

Figure S57: ATR-FTIR spectrum of **TpPa** synthesized with 1 equivalent of DIPEA. The keto C=O stretch is at 1602 cm^{-1}, the keto C=C stretch is at 1579 cm^{-1} and the enamine C–N stretch is at 1246 cm^{-1}.

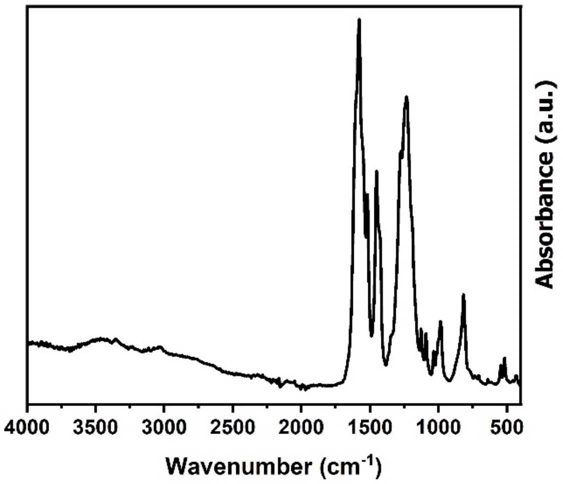

Figure S58: ATR-FTIR spectrum of **TpPa** synthesized with 1 equivalent of DIPEA (duplicate synthesis). The keto C=O stretch is at 1601 cm^{-1}, the keto C=C stretch is at 1578 cm^{-1} and the enamine C–N stretch is at 1245 cm^{-1}.

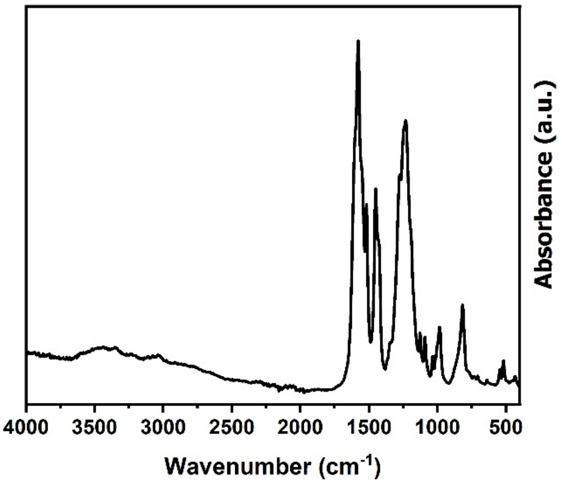

Figure S59: ATR-FTIR spectrum of **TpPa** synthesized with 2 equivalents of DIPEA. The keto C=O stretch is at 1600 cm^{-1}, the keto C=C stretch is at 1578 cm^{-1} and the enamine C–N stretch is at 1245 cm^{-1}.

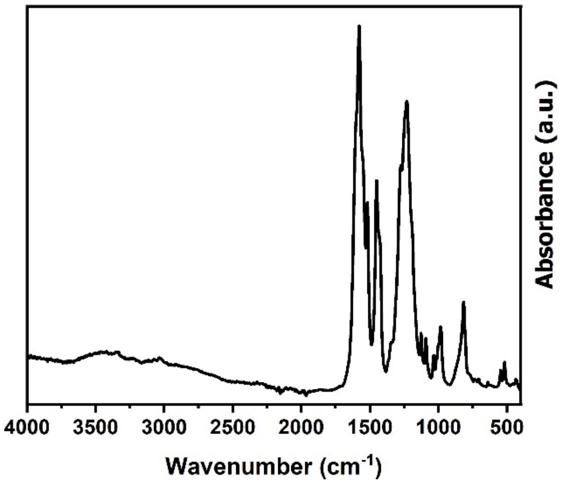

Figure S60: ATR-FTIR spectrum of **TpPa** synthesized with 2 equivalents of DIPEA (duplicate synthesis). The keto C=O stretch is at 1600 cm^{-1}, the keto C=C stretch is at 1578 cm^{-1} and the enamine C–N stretch is at 1242 cm^{-1}.

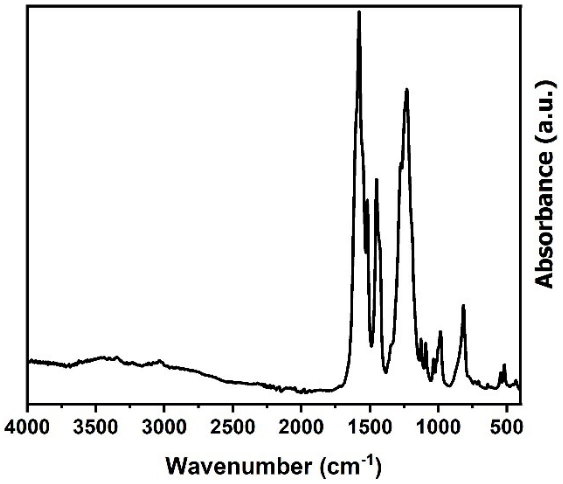

Figure S61: ATR-FTIR spectrum of **TpPa** synthesized with 10 equivalents of DIPEA. The keto C=O stretch is at 1601 cm^{-1}, the keto C=C stretch is at 1578 cm^{-1} and the enamine C–N stretch is at 1245 cm^{-1}.

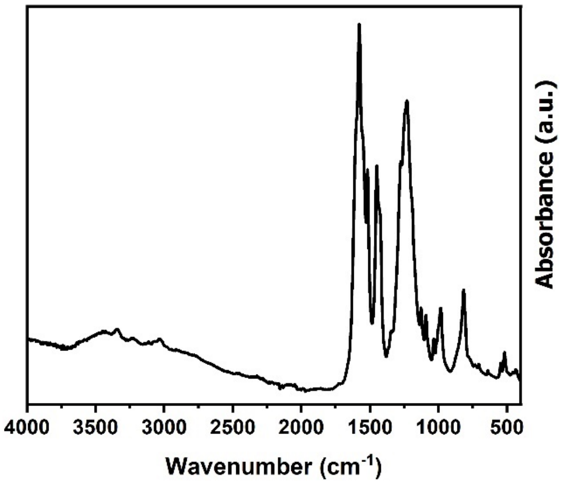

Figure S62: ATR-FTIR spectrum of **TpPa** synthesized with 10 equivalents of DIPEA (duplicate synthesis). The keto C=O stretch is at 1601 cm^{-1}, the keto C=C stretch is at 1576 cm^{-1} and the enamine C–N stretch is at 1246 cm^{-1}.

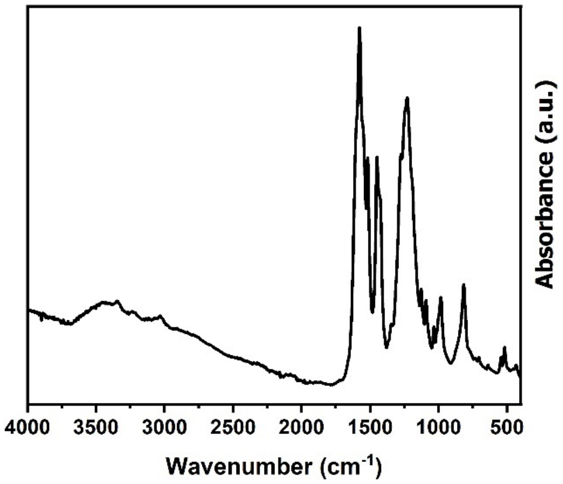

Figure S63: ATR-FTIR spectrum of **TpPa** synthesized with 20 equivalents of DIPEA. The keto C=O stretch is at 1599 cm^{-1}, the keto C=C stretch is at 1576 cm^{-1} and the enamine C–N stretch is at 1245 cm^{-1}.

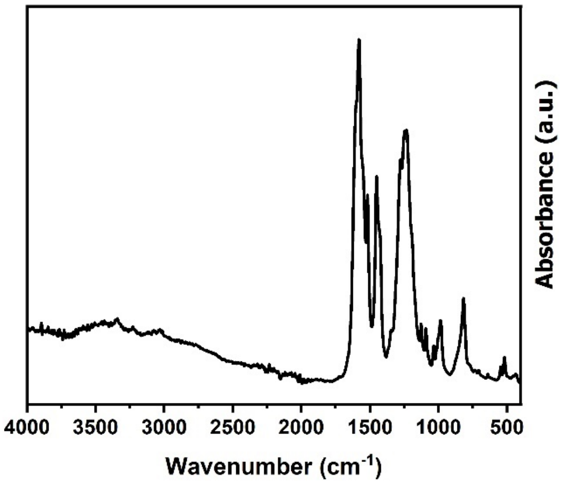

Figure S64: ATR-FTIR spectrum of **TpPa** synthesized with 20 equivalents of DIPEA (duplicate synthesis). The keto C=O stretch is at 1600 cm^{-1}, the keto C=C stretch is at 1579 cm^{-1} and the enamine C–N stretch is at 1246 cm^{-1}.

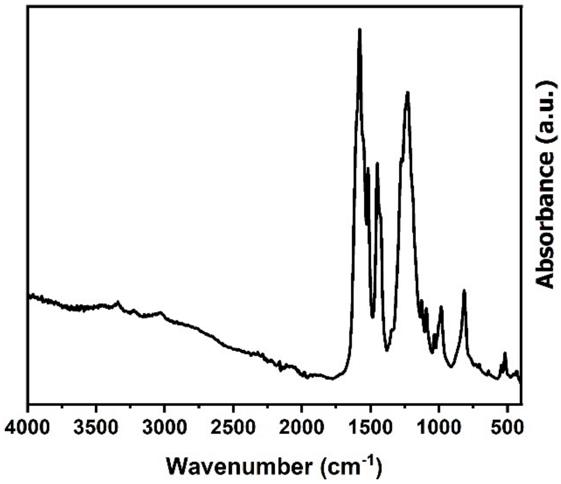

Figure S65: ATR-FTIR spectrum of **TpPa** synthesized with 40 equivalents of DIPEA. The keto C=O stretch is at 1599 cm^{-1}, the keto C=C stretch is at 1578 cm^{-1} and the enamine C–N stretch is at 1244 cm^{-1}.

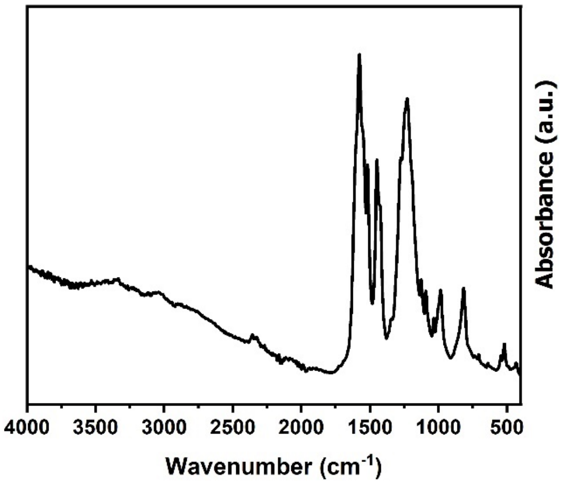

Figure S66: ATR-FTIR spectrum of **TpPa** synthesized with 40 equivalents of DIPEA (duplicate synthesis). The keto C=O stretch is at 1599 cm^{-1}, the keto C=C stretch is at 1578 cm^{-1} and the enamine C–N stretch is at 1244 cm^{-1}.

4.10 DBU

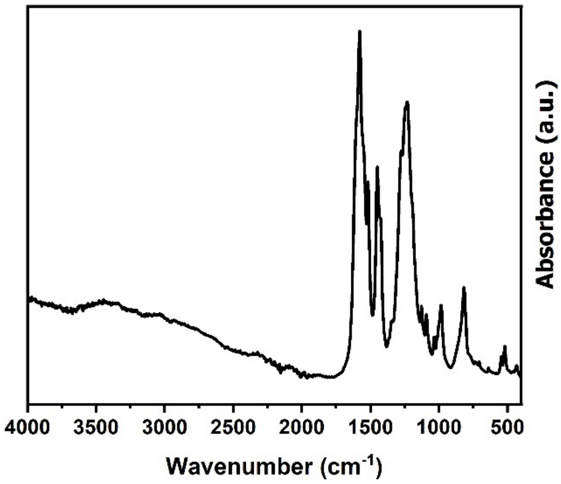

Figure S67: ATR-FTIR spectrum of **TpPa** synthesized with ⅓ equivalents of DBU. The keto C=O stretch is at 1603 cm^{-1}, the keto C=C stretch is at 1578 cm^{-1} and the enamine C–N stretch is at 1250 cm^{-1}.

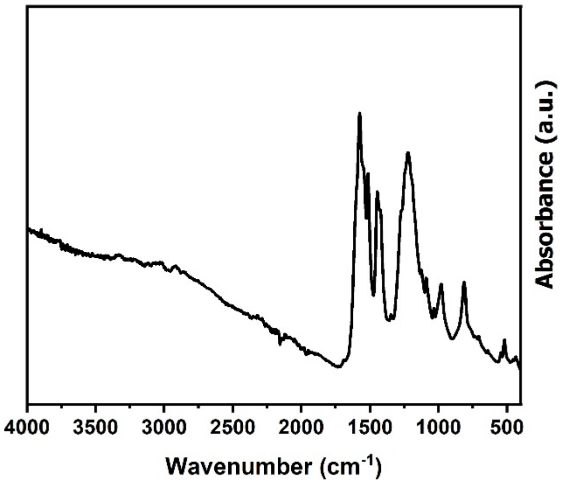

Figure S68: ATR-FTIR spectrum of **TpPa** synthesized with 2 equivalents of DBU. The keto C=O stretch is at 1599 cm^{-1}, the keto C=C stretch is at 1572 cm^{-1} and the enamine C–N stretch is at 1248 cm^{-1}.

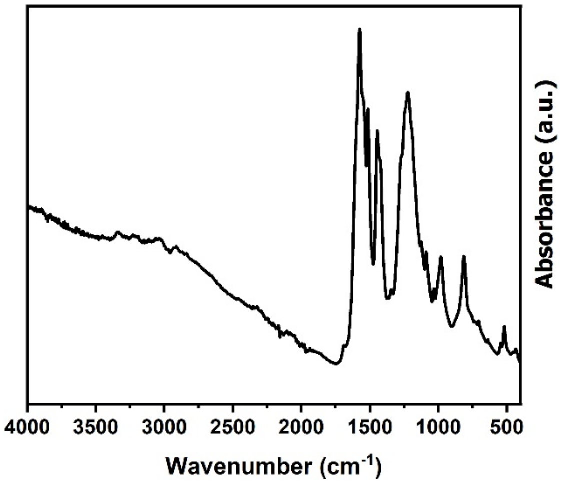

Figure S69: ATR-FTIR spectrum of **TpPa** synthesized with 2 equivalents of DBU (duplicate synthesis). The keto C=O stretch is at 1599 cm^{-1}, the keto C=C stretch is at 1573 cm^{-1} and the enamine C–N stretch is at 1248 cm^{-1}.

4.11 KOH

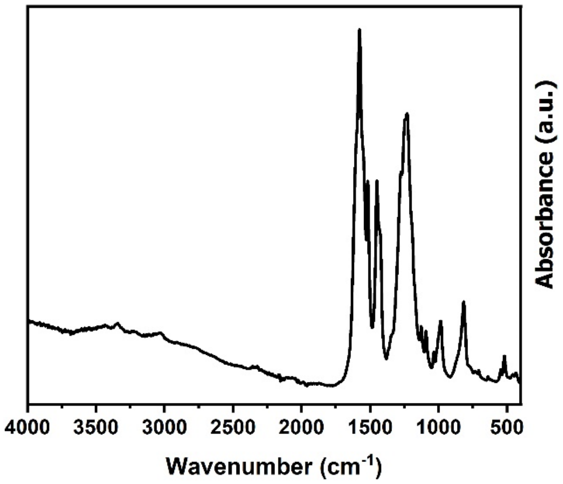

Figure S70: ATR-FTIR spectrum of **TpPa** synthesized with ⅓ equivalents of KOH. The keto C=O stretch is at 1601 cm^{-1}, the keto C=C stretch is at 1573 cm^{-1} and the enamine C–N stretch is at 1252 cm^{-1}.

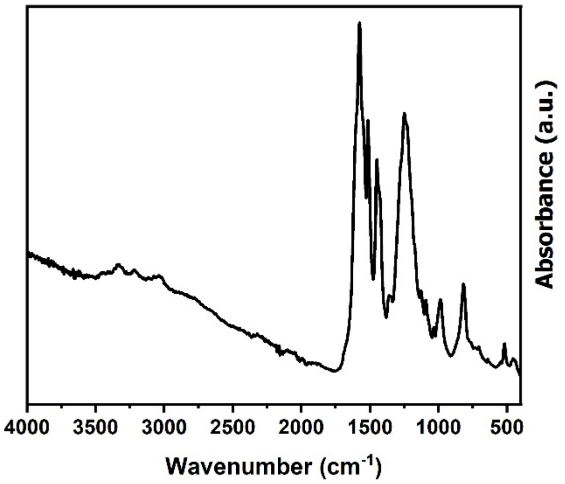

Figure S71: ATR-FTIR spectrum of **TpPa** synthesized with 2 equivalents of KOH. The keto C=O stretch is at 1600 cm^{-1}, the keto C=C stretch is at 1572 cm^{-1} and the enamine C–N stretch is at 1255 cm^{-1}.

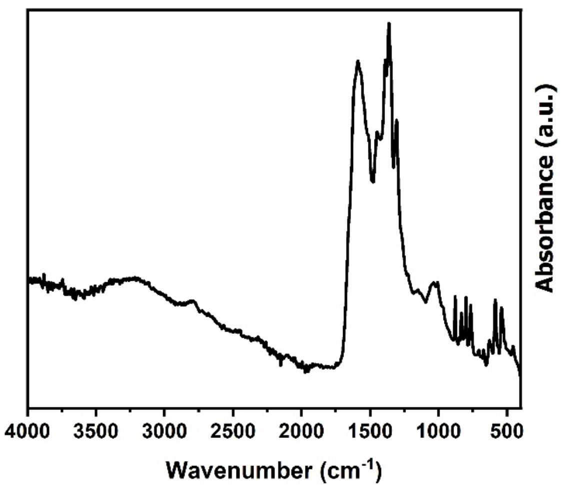

Figure S72: ATR-FTIR spectrum of **TpPa** synthesized with 40 equivalents of KOH. The keto C=O stretch is at 1612 cm^{-1}, the keto C=C stretch is at 1588 cm^{-1}. The enamine C–N stretch is not clearly visible. The spectrum is quite different from the rest. The peak at 1360 cm^{-1}, corresponding to Tp[S2], is much more pronounced than in other samples. The high KOH concentration made the solvent exchange very difficult, which may have caused this result.

5. PXRD

For selected frameworks, their crystallinity was investigated by powder XRD (see Figures S73-S81).

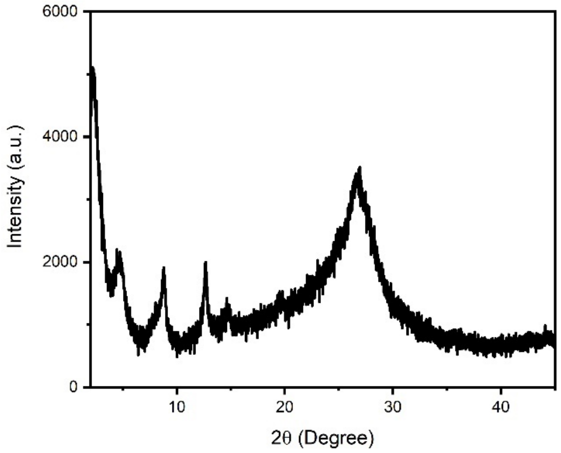

Figure S73: PXRD spectrum of **TpPa** synthesized with 10 equivalents of formic acid.

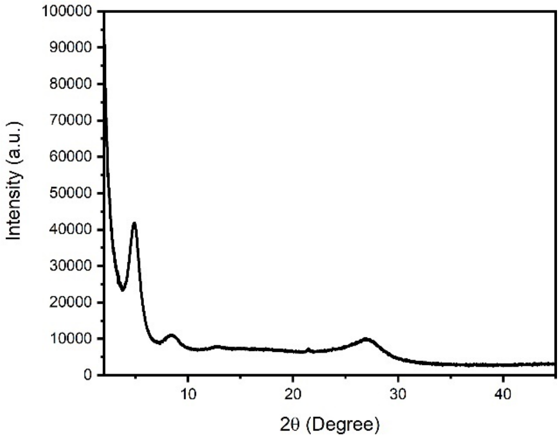

Figure S74: PXRD spectrum of **TpPa** synthesized with 2 equivalents of acetic acid.

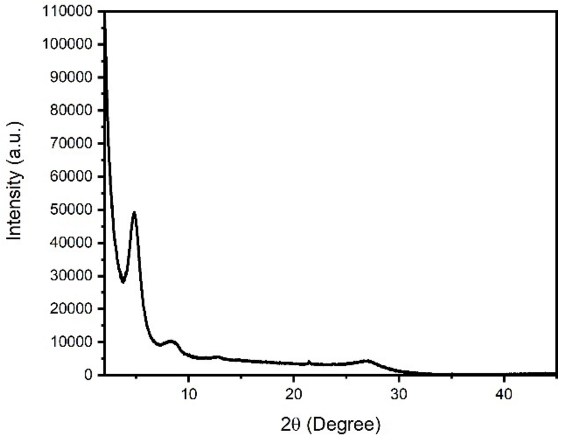

Figure S75: PXRD spectrum of **TpPa** synthesized with 10 equivalents of acetic acid.

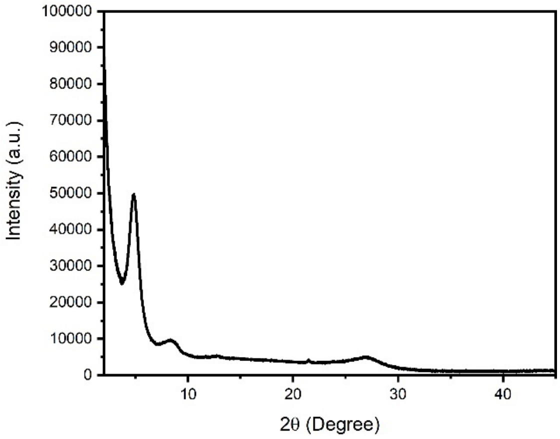

Figure S76: PXRD spectrum of **TpPa** synthesized without added acid or base.

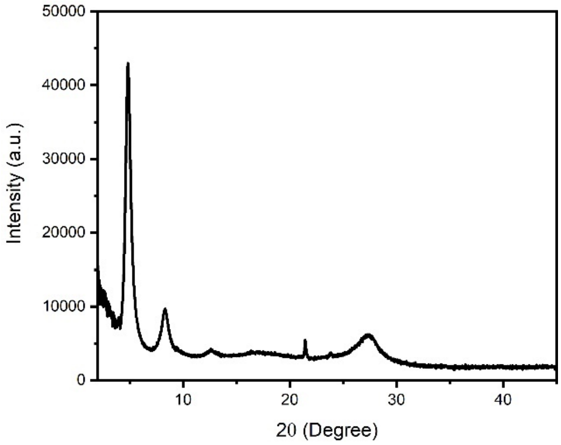

Figure S77: PXRD spectrum of **TpPa** synthesized with 2 equivalents of TEA.

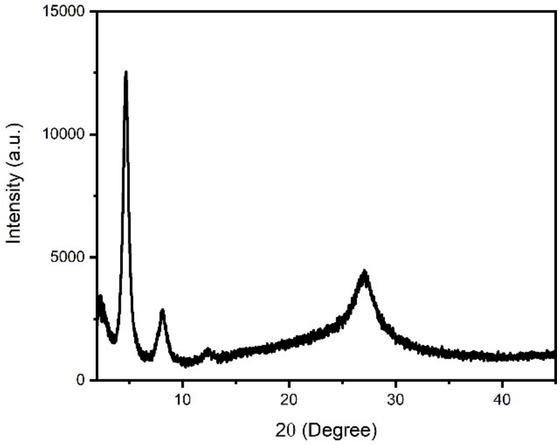

Figure S78: PXRD spectrum of **TpPa** synthesized with 10 equivalents of TEA.

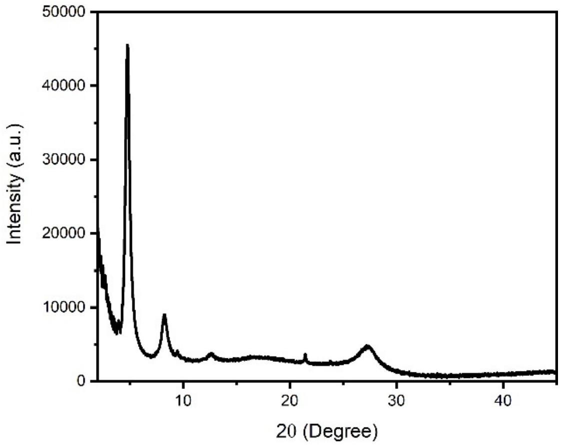

Figure S79: PXRD spectrum of **TpPa** synthesized with 2 equivalents of DIPEA.

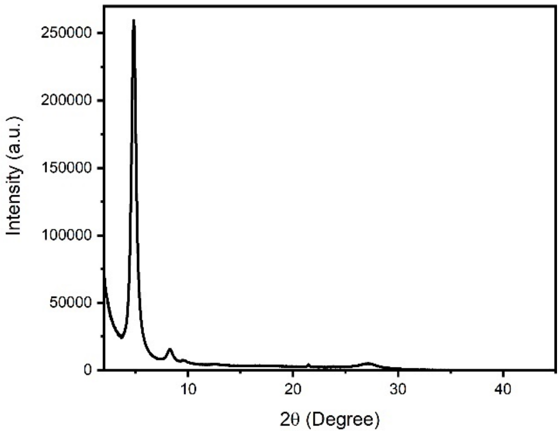

Figure S80: PXRD spectrum of **TpPa** synthesized with 10 equivalents of DIPEA.

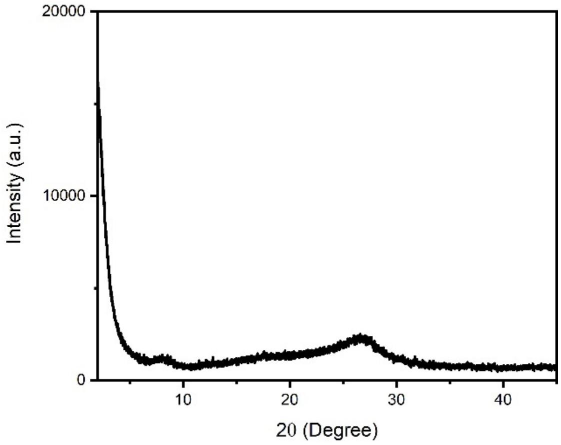

Figure S81: PXRD spectrum of **TpPa** synthesized with 2 equivalents of KOH.

6. N_2 sorption isotherms/BET graphs

6.1 Chloroacetic acid

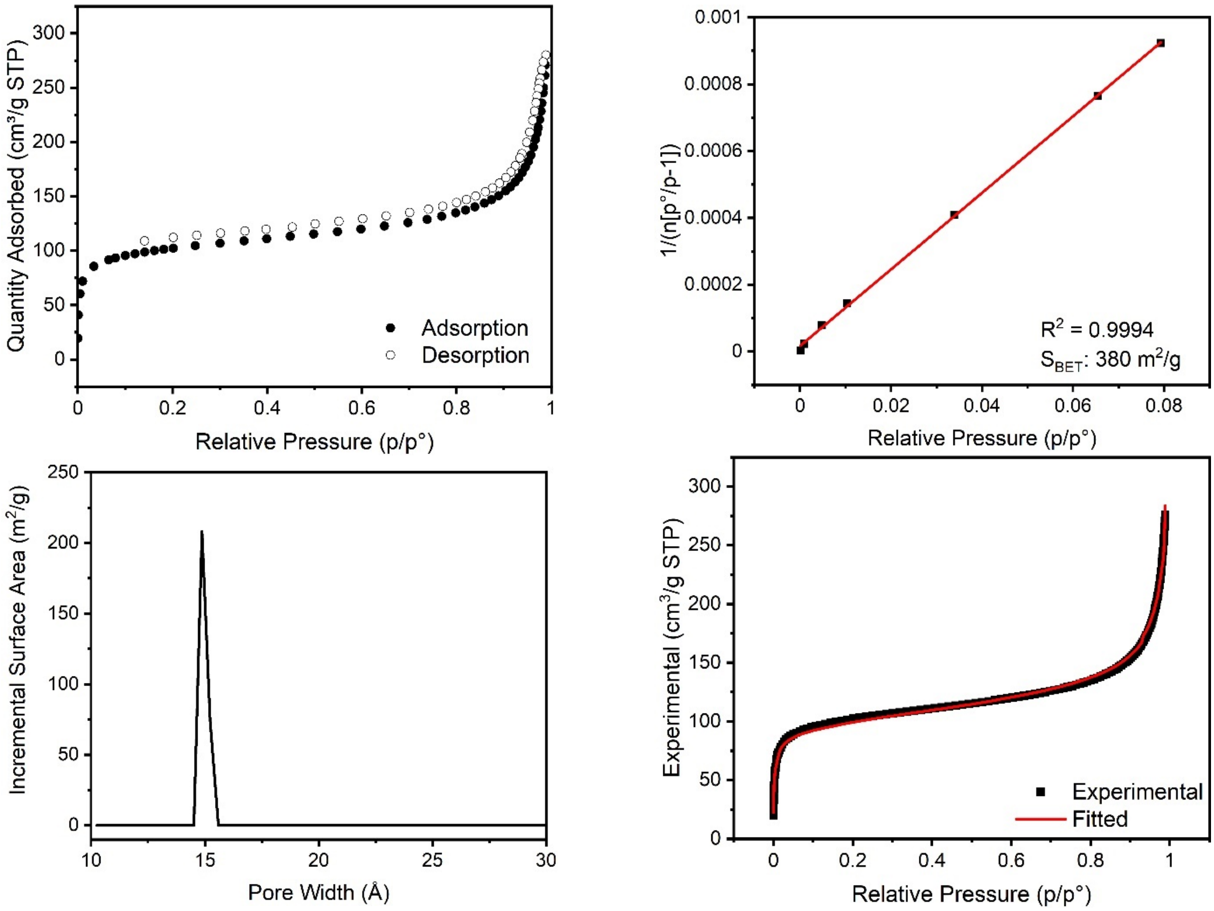

Figure S82: Adsorption measurements for **TpPa** synthesized with ⅓ equivalents of chloroacetic acid. Top left: adsorption and desorption isotherm. Top right: linear fit to calculate the BET surface area, including R^2. Bottom left: pore size distribution. Bottom right: comparison of the experimental adsorption isotherm with the theoretically modelled isotherm.

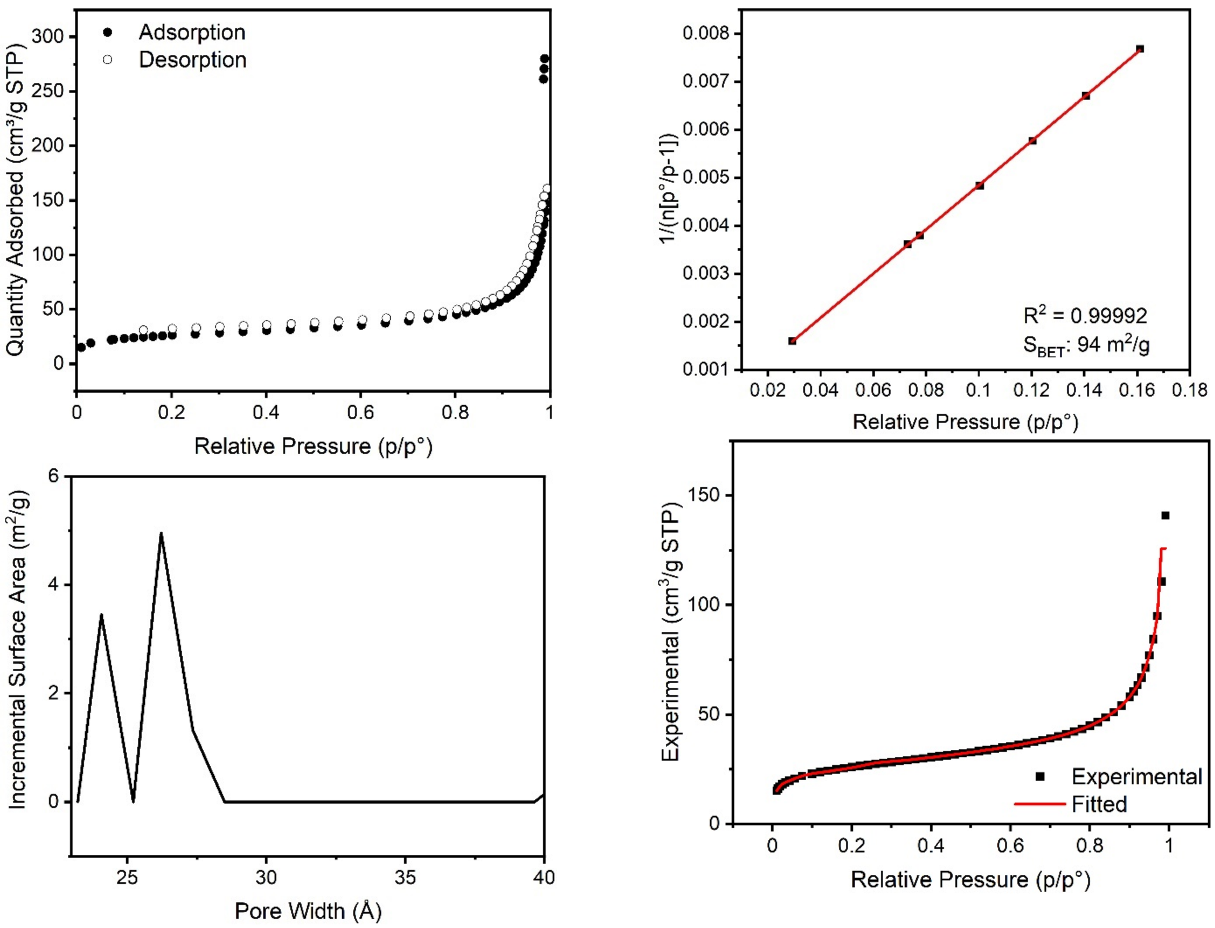

Figure S83: Adsorption measurements for **TpPa** synthesized with 1 equivalent of chloroacetic acid. Top left: adsorption and desorption isotherm. Top right: linear fit to calculate the BET surface area, including R^2. Bottom left: pore size distribution. Bottom right: comparison of the experimental adsorption isotherm with the theoretically modelled isotherm.

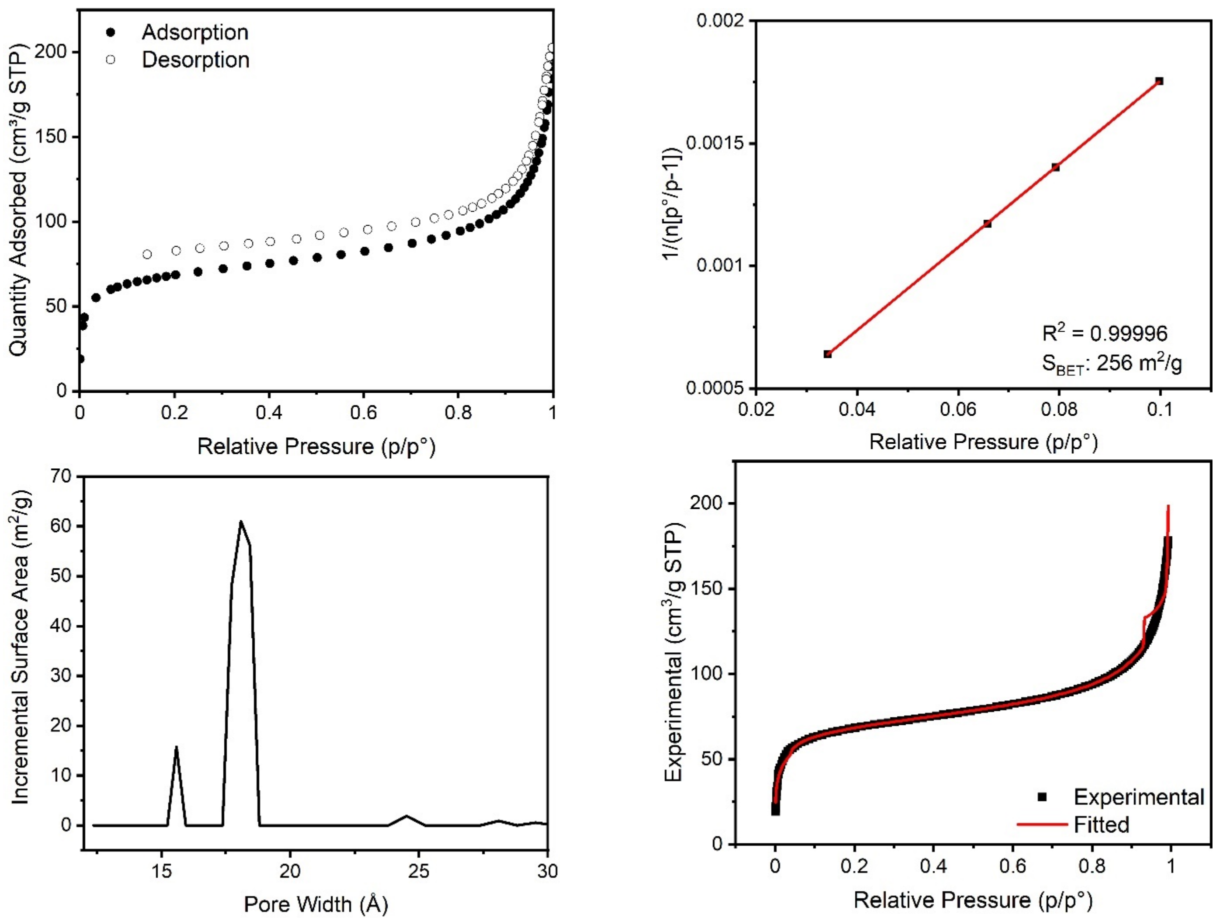

Figure S84: Adsorption measurements for **TpPa** synthesized with 2 equivalents of chloroacetic acid. Top left: adsorption and desorption isotherm. Top right: linear fit to calculate the BET surface area, including R^2. Bottom left: pore size distribution. Bottom right: comparison of the experimental adsorption isotherm with the theoretically modelled isotherm.

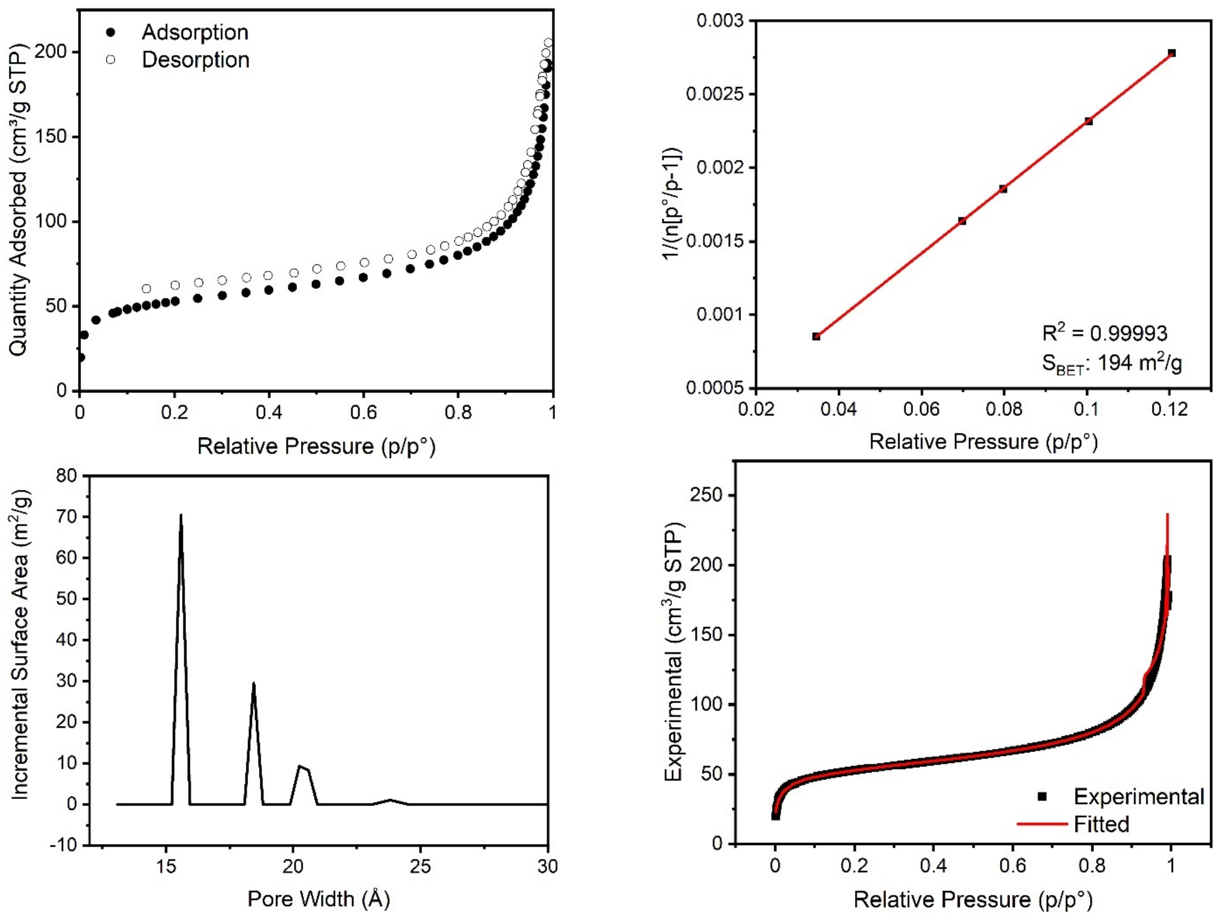

Figure S85: Adsorption measurements for **TpPa** synthesized with 10 equivalents of chloroacetic acid. Top left: adsorption and desorption isotherm. Top right: linear fit to calculate the BET surface area, including R^2. Bottom left: pore size distribution. Bottom right: comparison of the experimental adsorption isotherm with the theoretically modelled isotherm.

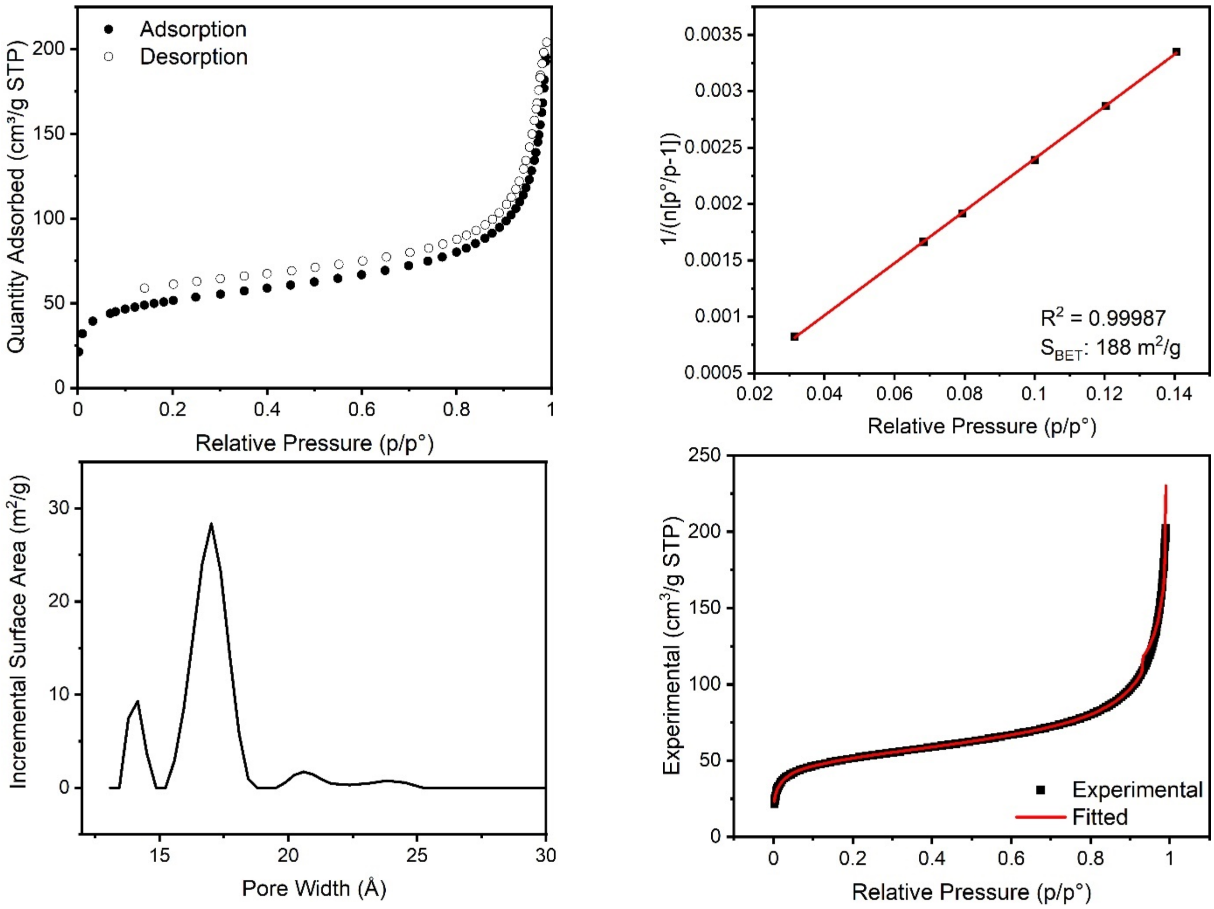

Figure S86: Adsorption measurements for **TpPa** synthesized with 20 equivalents of chloroacetic acid. Top left: adsorption and desorption isotherm. Top right: linear fit to calculate the BET surface area, including R^2. Bottom left: pore size distribution. Bottom right: comparison of the experimental adsorption isotherm with the theoretically modelled isotherm.

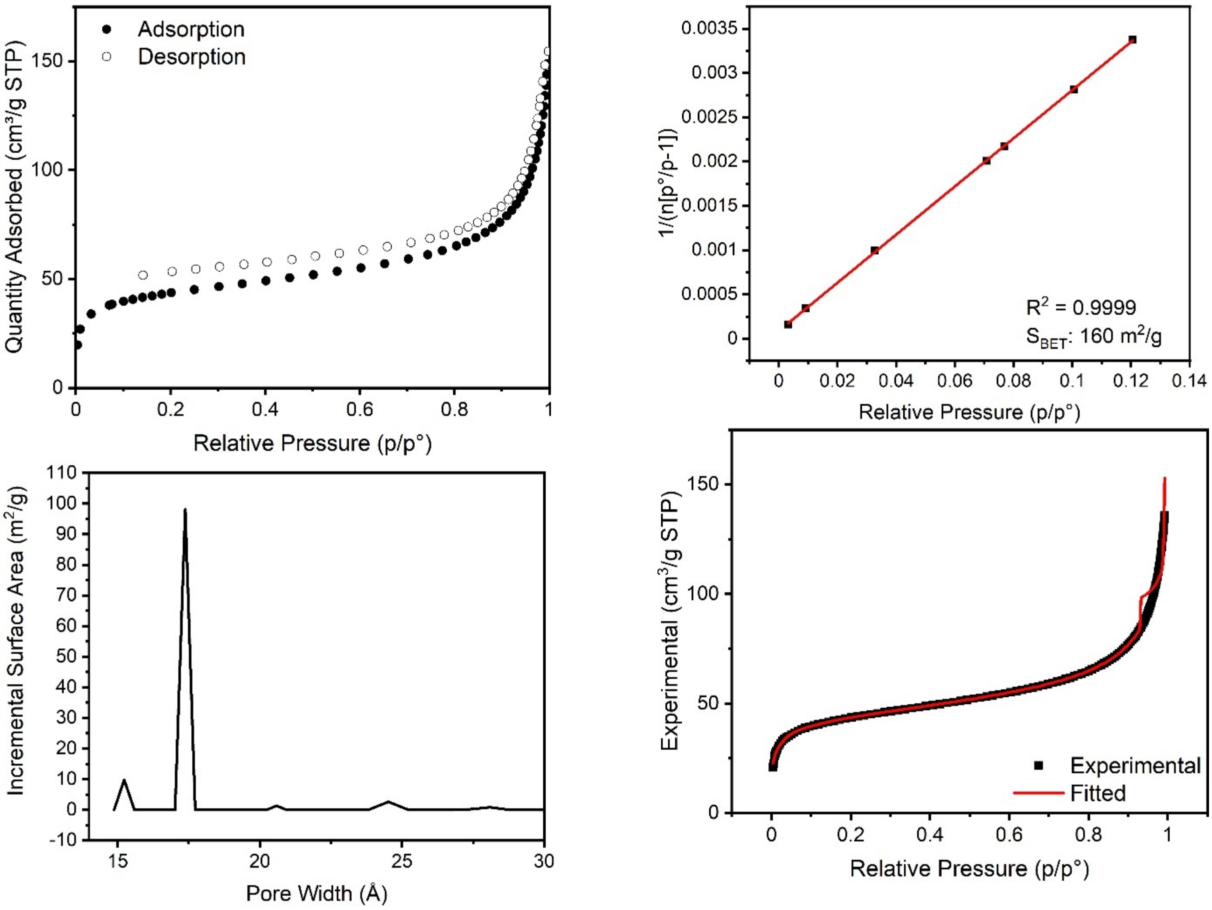

Figure S87: Adsorption measurements for **TpPa** synthesized with 40 equivalents of chloroacetic acid. Top left: adsorption and desorption isotherm. Top right: linear fit to calculate the BET surface area, including R^2. Bottom left: pore size distribution. Bottom right: comparison of the experimental adsorption isotherm with the theoretically modelled isotherm.

6.2 Formic acid

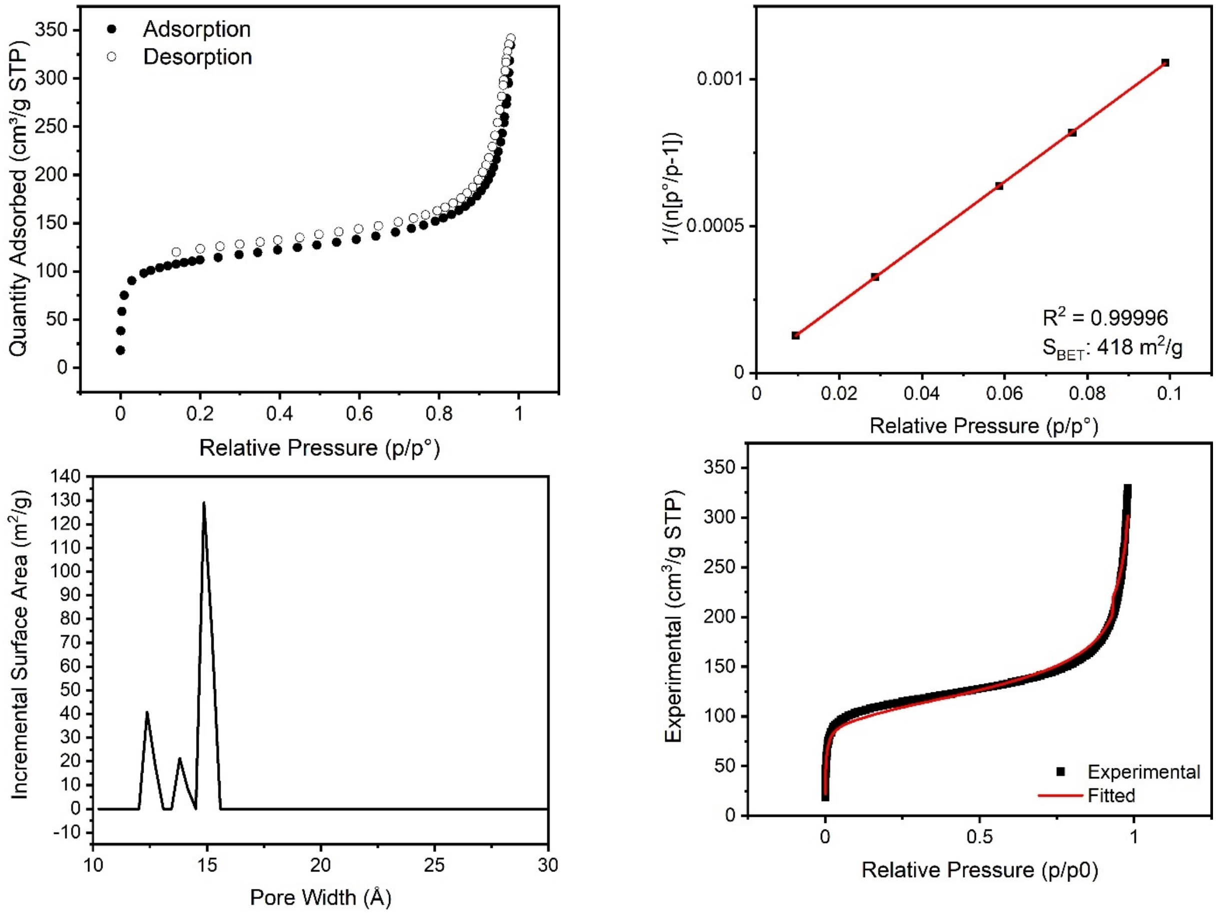

Figure S88: Adsorption measurements for **TpPa** synthesized with ⅓ equivalents of formic acid. Top left: adsorption and desorption isotherm. Top right: linear fit to calculate the BET surface area, including R^2. Bottom left: pore size distribution. Bottom right: comparison of the experimental adsorption isotherm with the theoretically modelled isotherm.

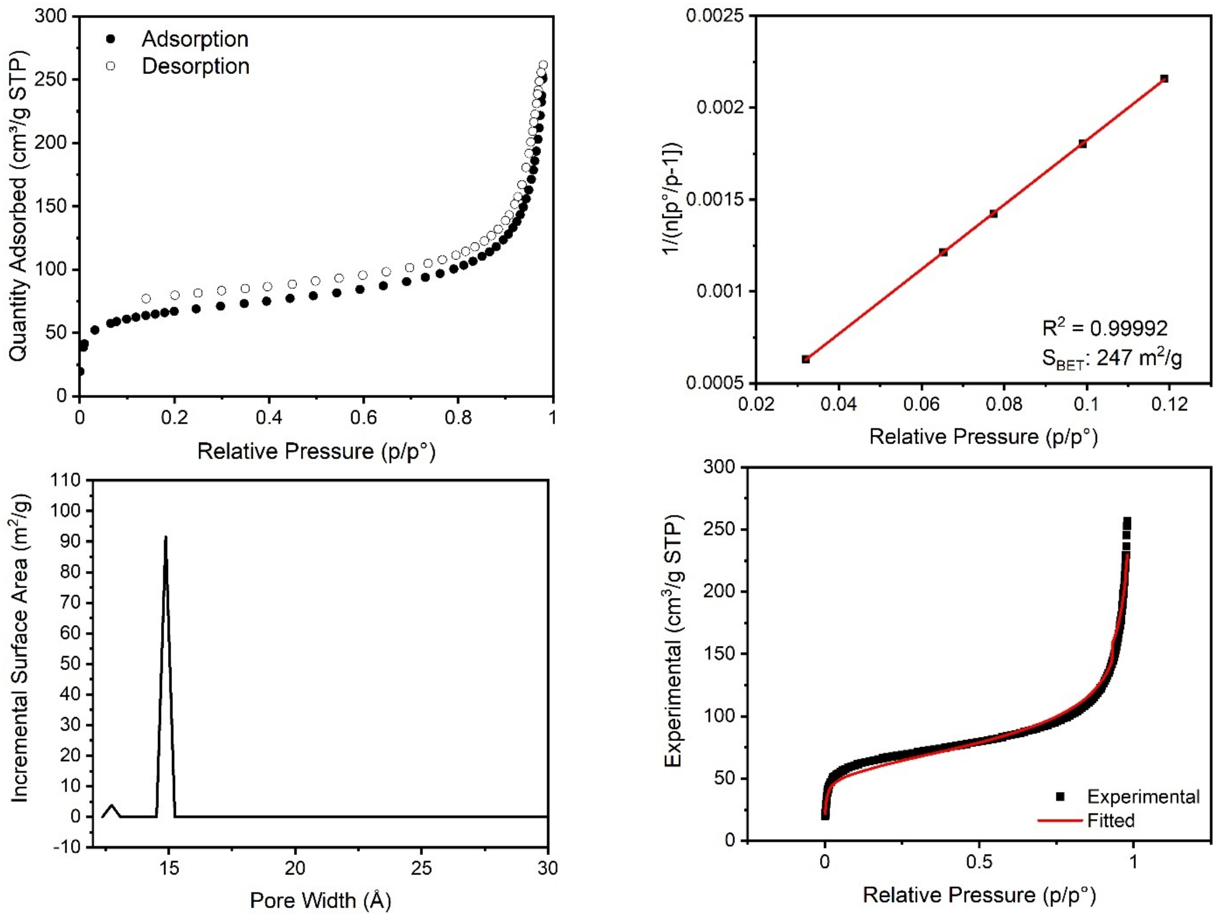

Figure S89: Adsorption measurements for **TpPa** synthesized with 1 equivalent of formic acid. Top left: adsorption and desorption isotherm. Top right: linear fit to calculate the BET surface area, including R^2. Bottom left: pore size distribution. Bottom right: comparison of the experimental adsorption isotherm with the theoretically modelled isotherm.

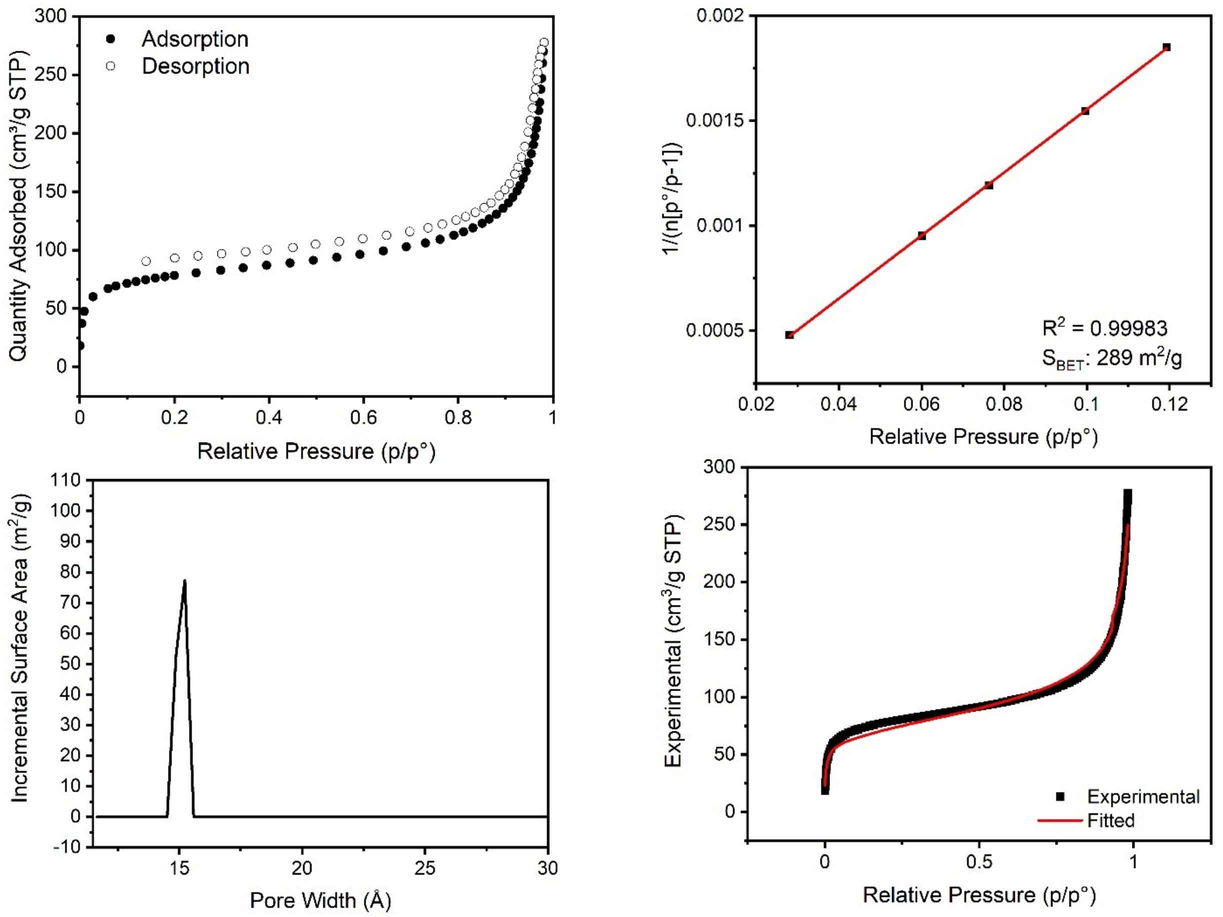

Figure S90: Adsorption measurements for **TpPa** synthesized with 2 equivalents of formic acid. Top left: adsorption and desorption isotherm. Top right: linear fit to calculate the BET surface area, including R^2. Bottom left: pore size distribution. Bottom right: comparison of the experimental adsorption isotherm with the theoretically modelled isotherm.

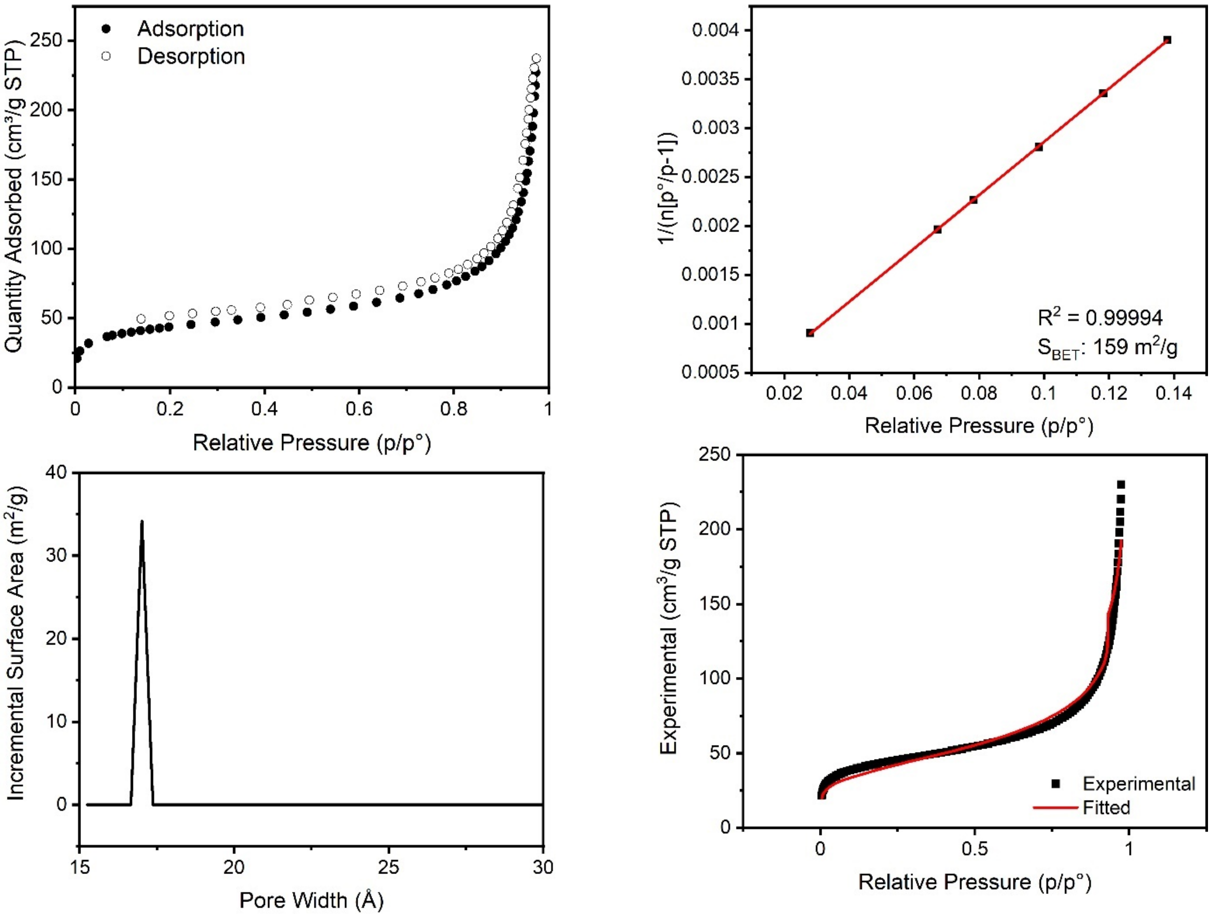

Figure S91: Adsorption measurements for **TpPa** synthesized with 10 equivalents of formic acid. Top left: adsorption and desorption isotherm. Top right: linear fit to calculate the BET surface area, including R^2. Bottom left: pore size distribution. Bottom right: comparison of the experimental adsorption isotherm with the theoretically modelled isotherm.

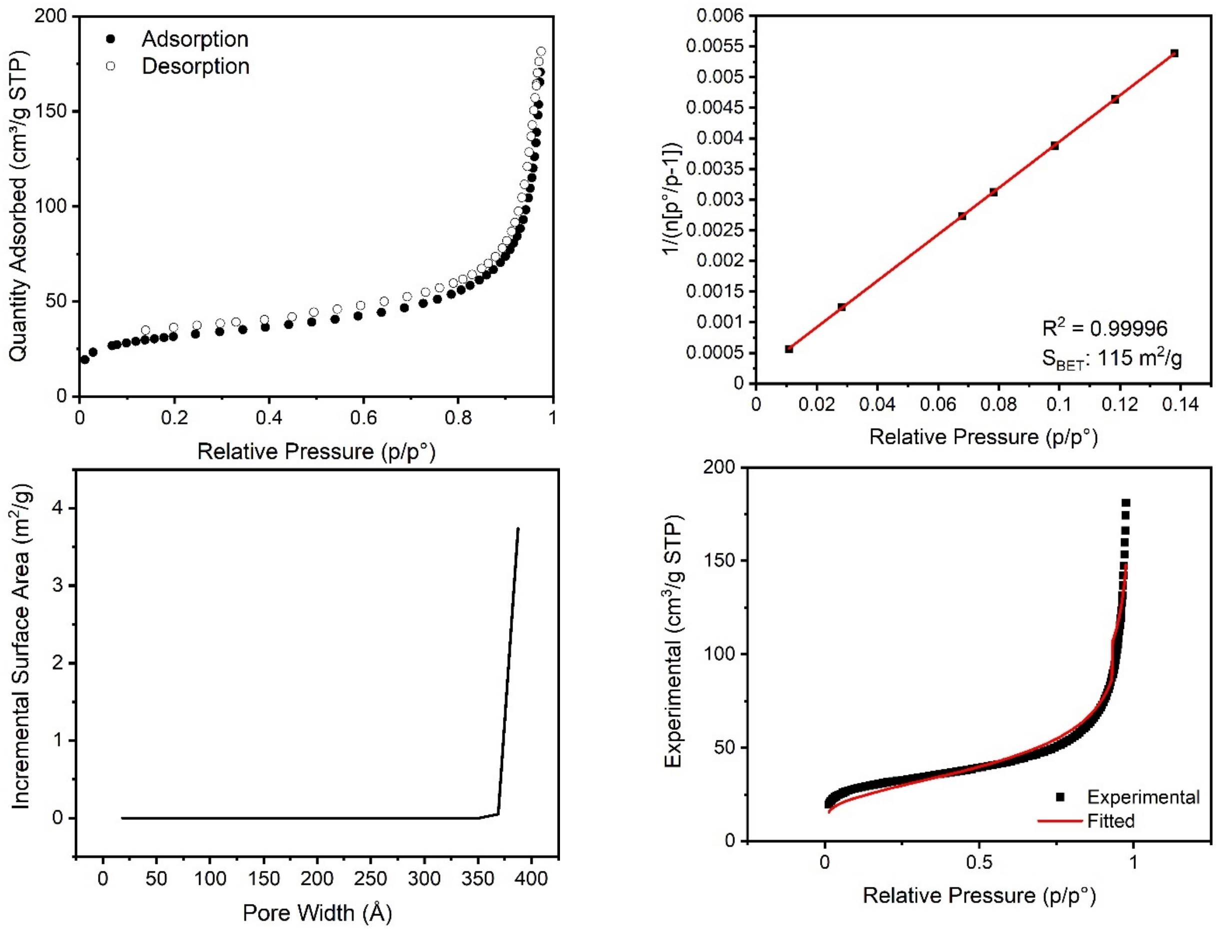

Figure S92: Adsorption measurements for **TpPa** synthesized with 20 equivalents of formic acid. Top left: adsorption and desorption isotherm. Top right: linear fit to calculate the BET surface area, including R^2. Bottom left: pore size distribution. Bottom right: comparison of the experimental adsorption isotherm with the theoretically modelled isotherm. No good DFT fit was found.

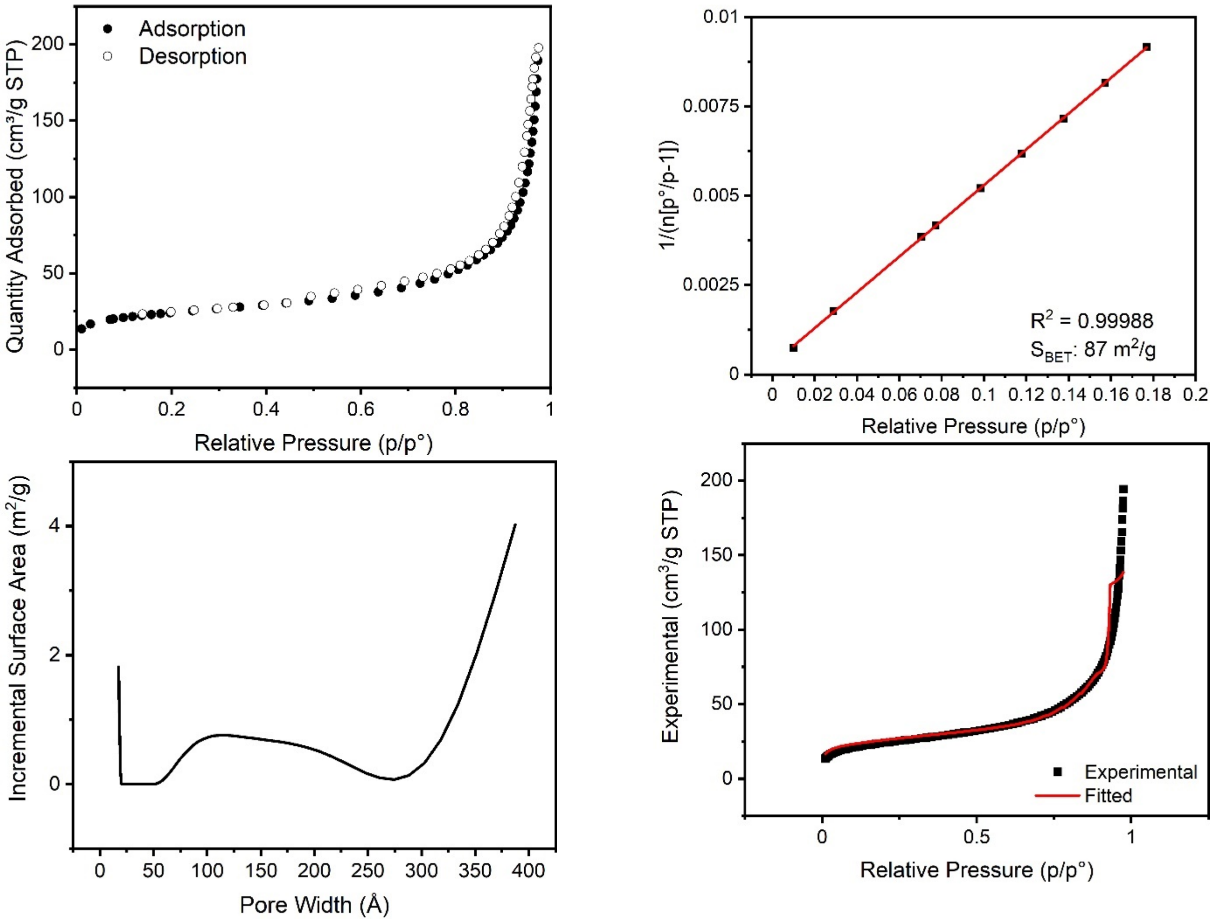

Figure S93: Adsorption measurements for **TpPa** synthesized with 40 equivalents of formic acid. Top left: adsorption and desorption isotherm. Top right: linear fit to calculate the BET surface area, including R^2. Bottom left: pore size distribution. Bottom right: comparison of the experimental adsorption isotherm with the theoretically modelled isotherm. No good DFT fit was found.

6.3 Acetic acid

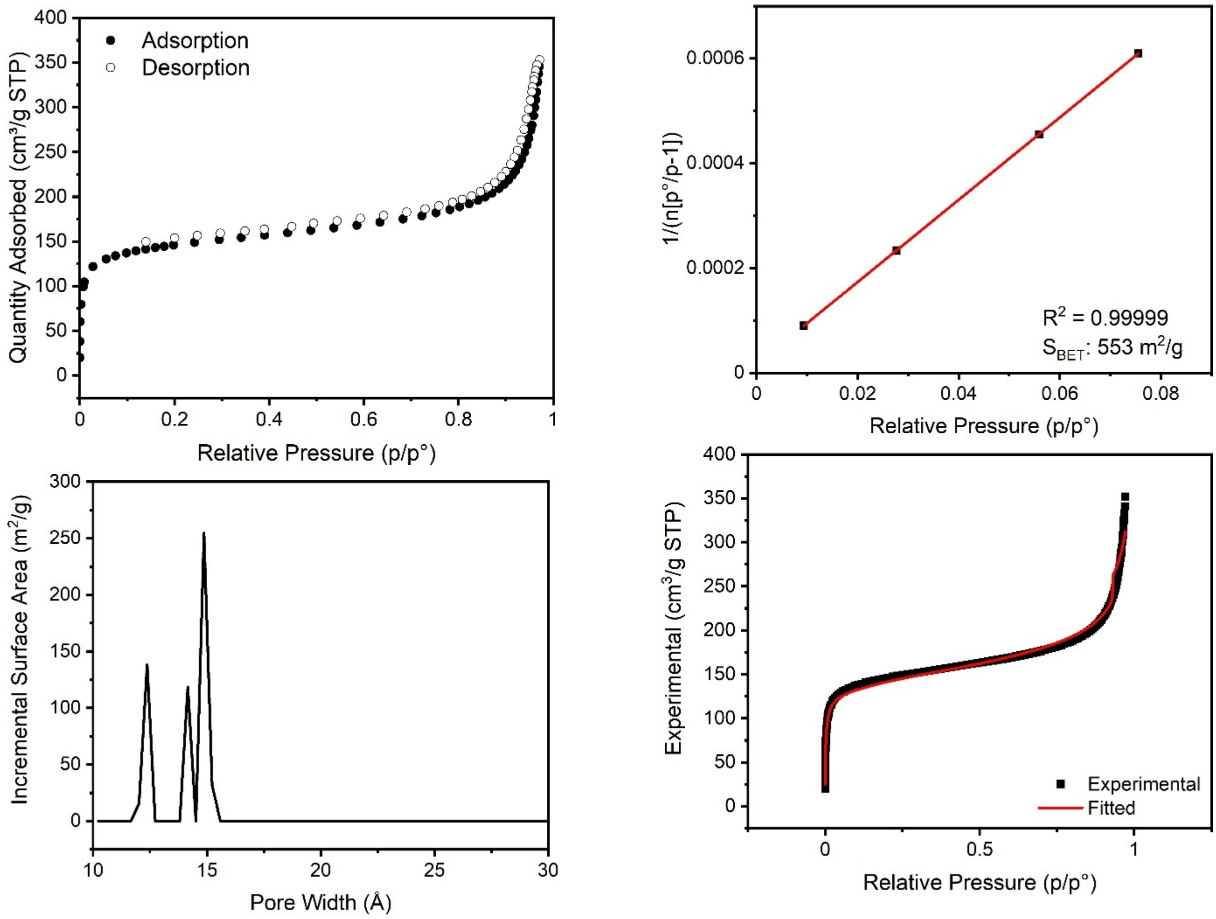

Figure S94: Adsorption measurements for **TpPa** synthesized with ⅓ equivalents of acetic acid. Top left: adsorption and desorption isotherm. Top right: linear fit to calculate the BET surface area, including R^2. Bottom left: pore size distribution. Bottom right: comparison of the experimental adsorption isotherm with the theoretically modelled isotherm.

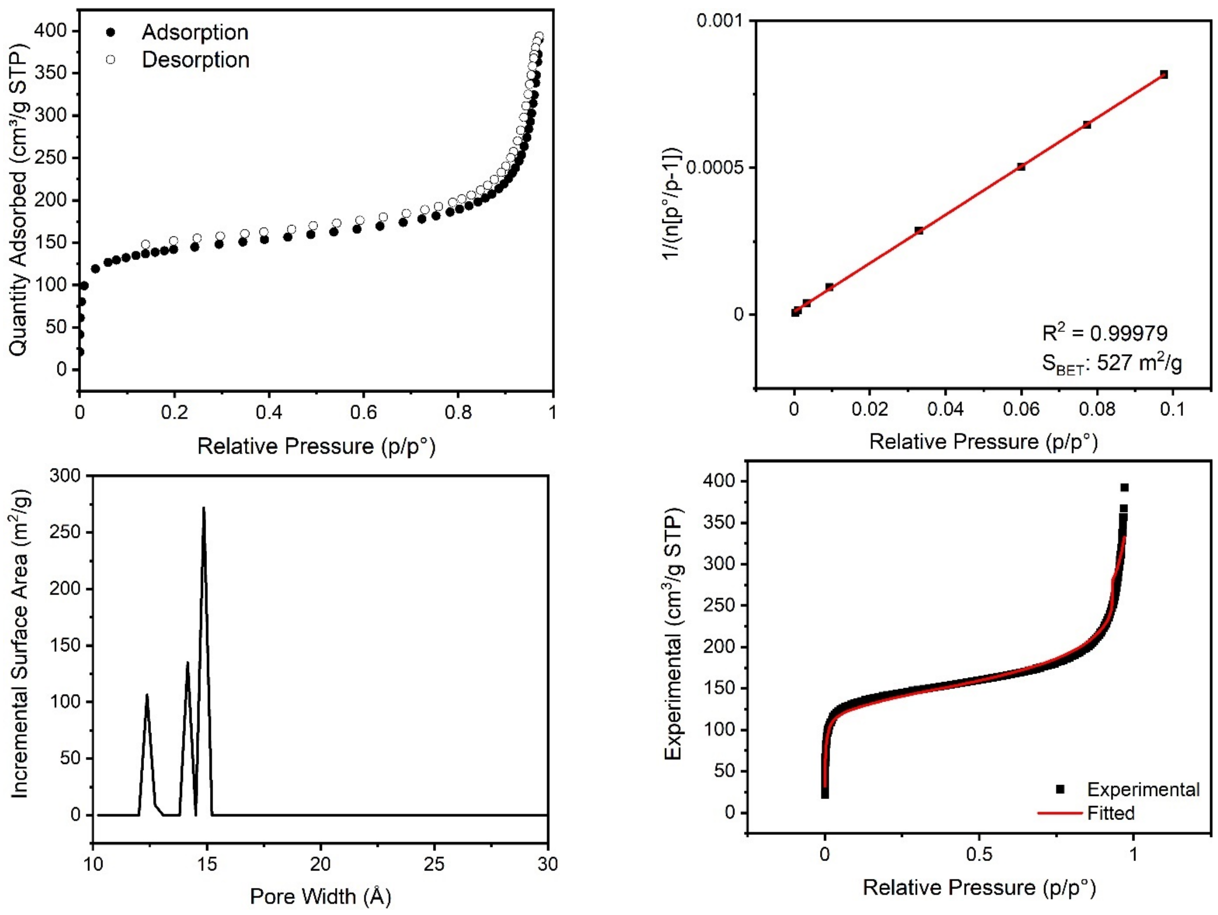

Figure S95: Adsorption measurements for **TpPa** synthesized with 1 equivalent of acetic acid. Top left: adsorption and desorption isotherm. Top right: linear fit to calculate the BET surface area, including R^2. Bottom left: pore size distribution. Bottom right: comparison of the experimental adsorption isotherm with the theoretically modelled isotherm.

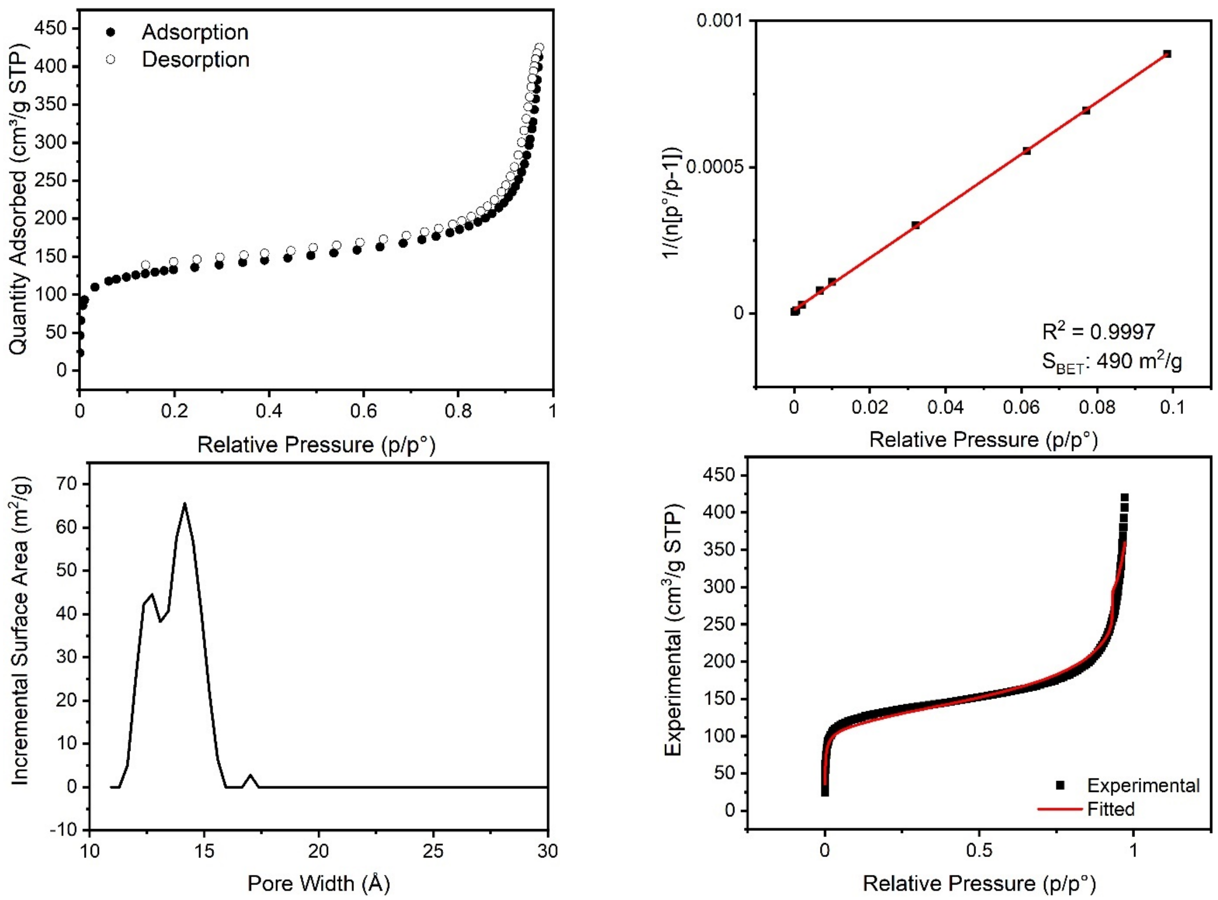

Figure S96: Adsorption measurements for **TpPa** synthesized with 2 equivalents of acetic acid. Top left: adsorption and desorption isotherm. Top right: linear fit to calculate the BET surface area, including R^2. Bottom left: pore size distribution. Bottom right: comparison of the experimental adsorption isotherm with the theoretically modelled isotherm.

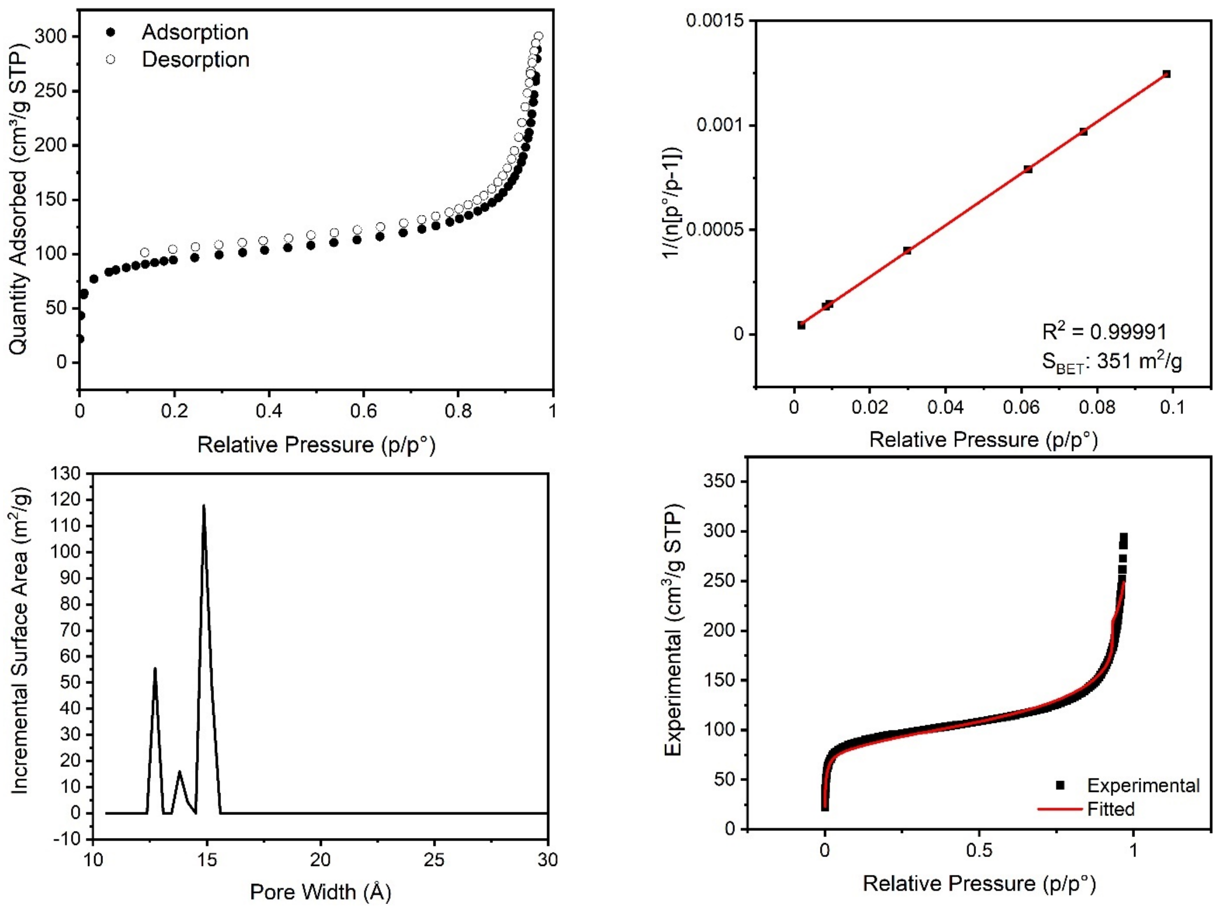

Figure S97: Adsorption measurements for **TpPa** synthesized with 10 equivalents of acetic acid. Top left: adsorption and desorption isotherm. Top right: linear fit to calculate the BET surface area, including R^2. Bottom left: pore size distribution. Bottom right: comparison of the experimental adsorption isotherm with the theoretically modelled isotherm.

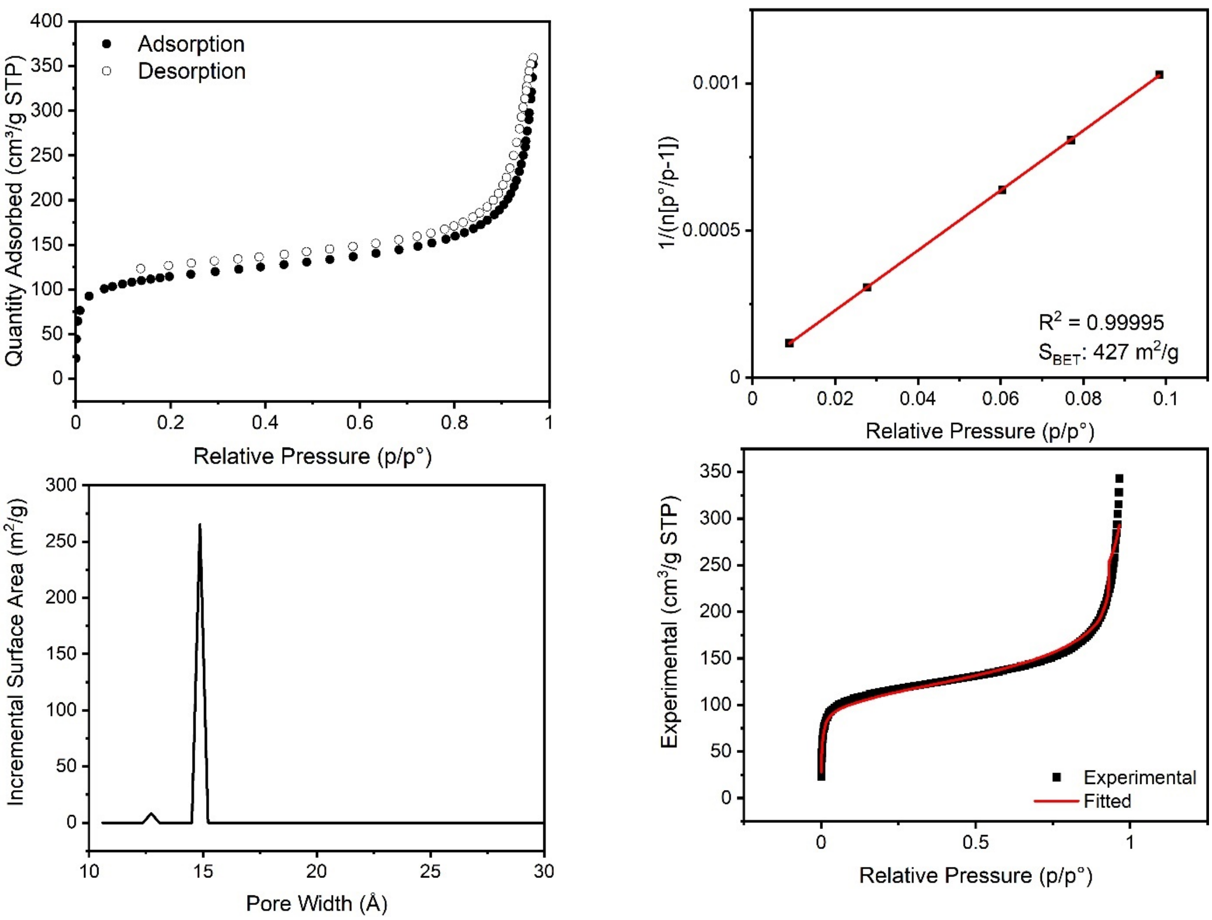

Figure S98: Adsorption measurements for **TpPa** synthesized with 20 equivalents of acetic acid. Top left: adsorption and desorption isotherm. Top right: linear fit to calculate the BET surface area, including R^2. Bottom left: pore size distribution. Bottom right: comparison of the experimental adsorption isotherm with the theoretically modelled isotherm.

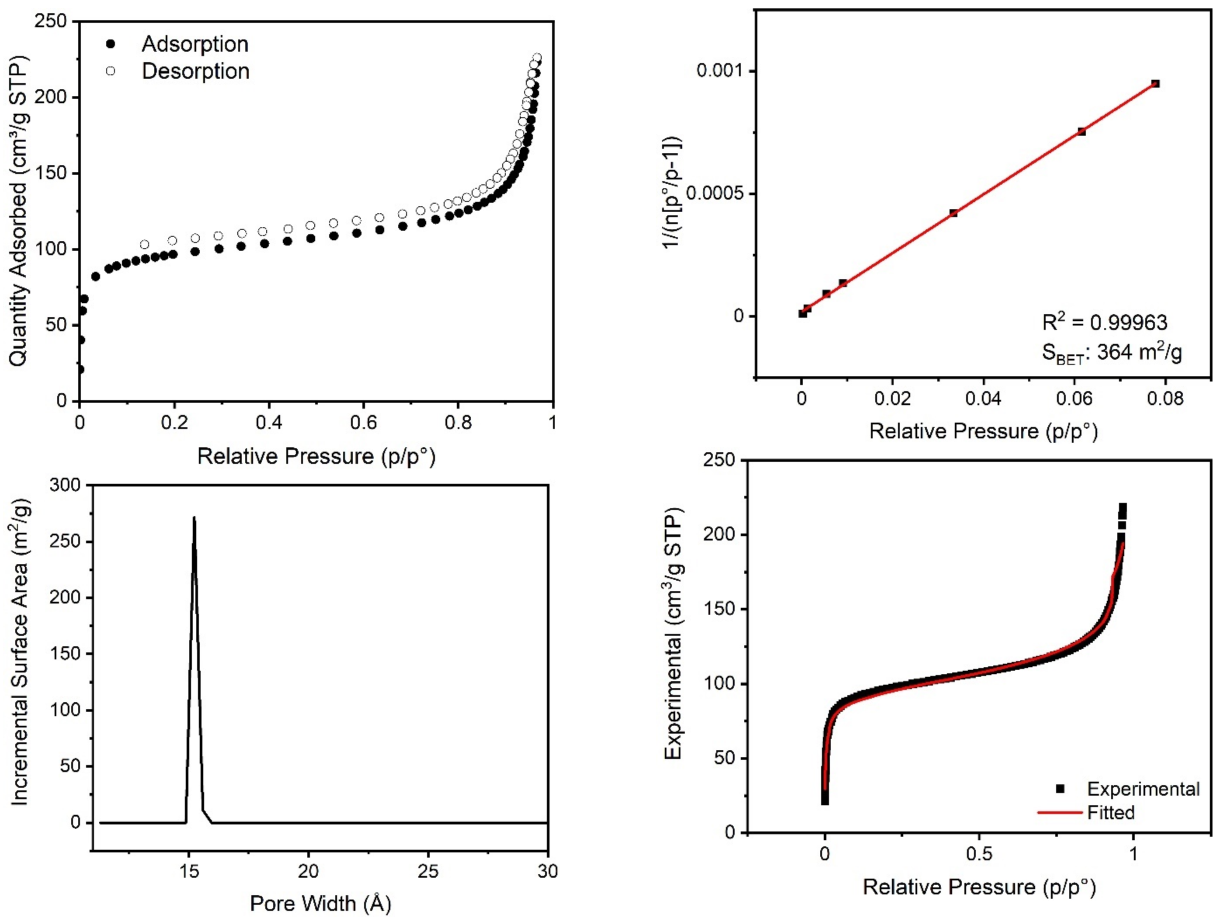

Figure S99: Adsorption measurements for **TpPa** synthesized with 40 equivalents of acetic acid. Top left: adsorption and desorption isotherm. Top right: linear fit to calculate the BET surface area, including R^2. Bottom left: pore size distribution. Bottom right: comparison of the experimental adsorption isotherm with the theoretically modelled isotherm.

6.4 Propionic acid

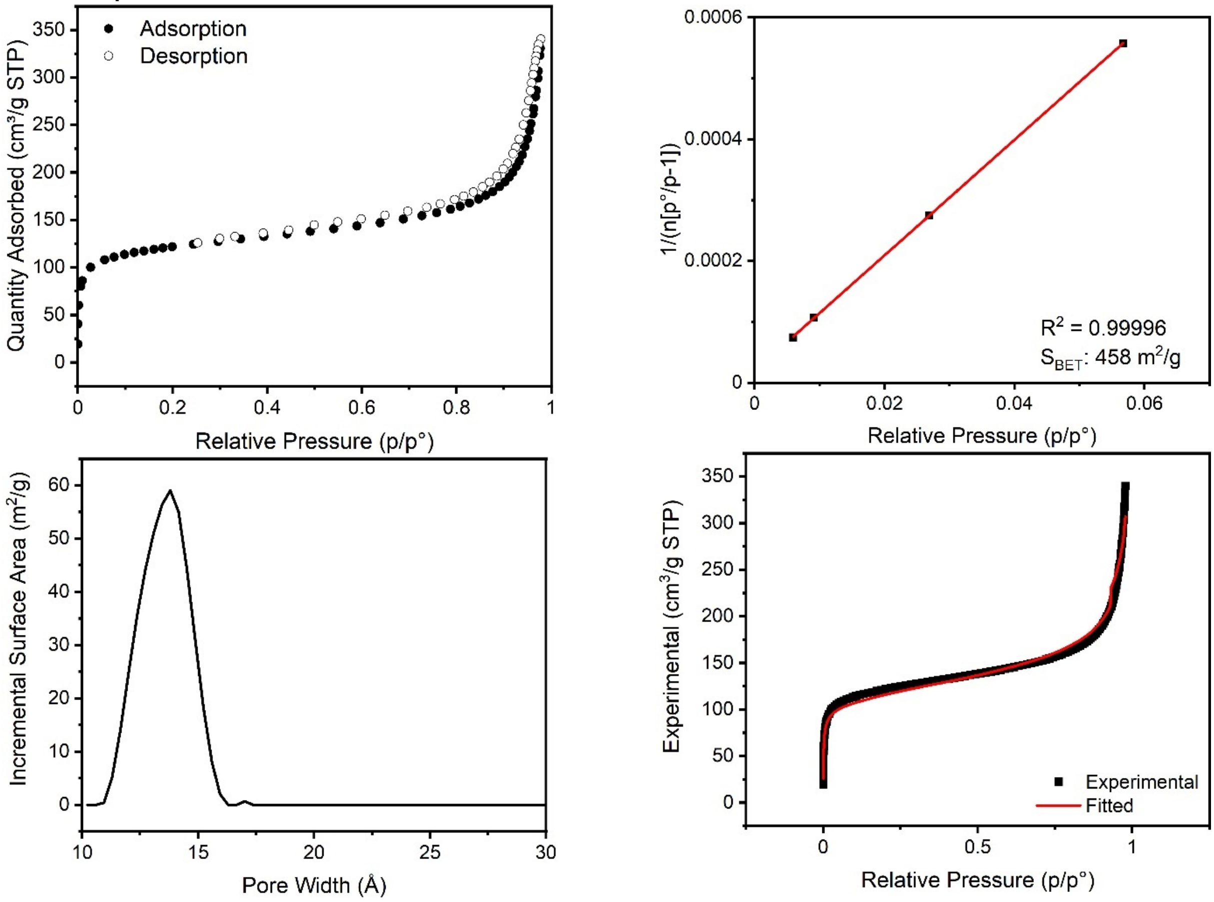

Figure S100: Adsorption measurements for **TpPa** synthesized with ⅓ equivalents of propionic acid. Top left: adsorption and desorption isotherm. Top right: linear fit to calculate the BET surface area, including R^2. Bottom left: pore size distribution. Bottom right: comparison of the experimental adsorption isotherm with the theoretically modelled isotherm.

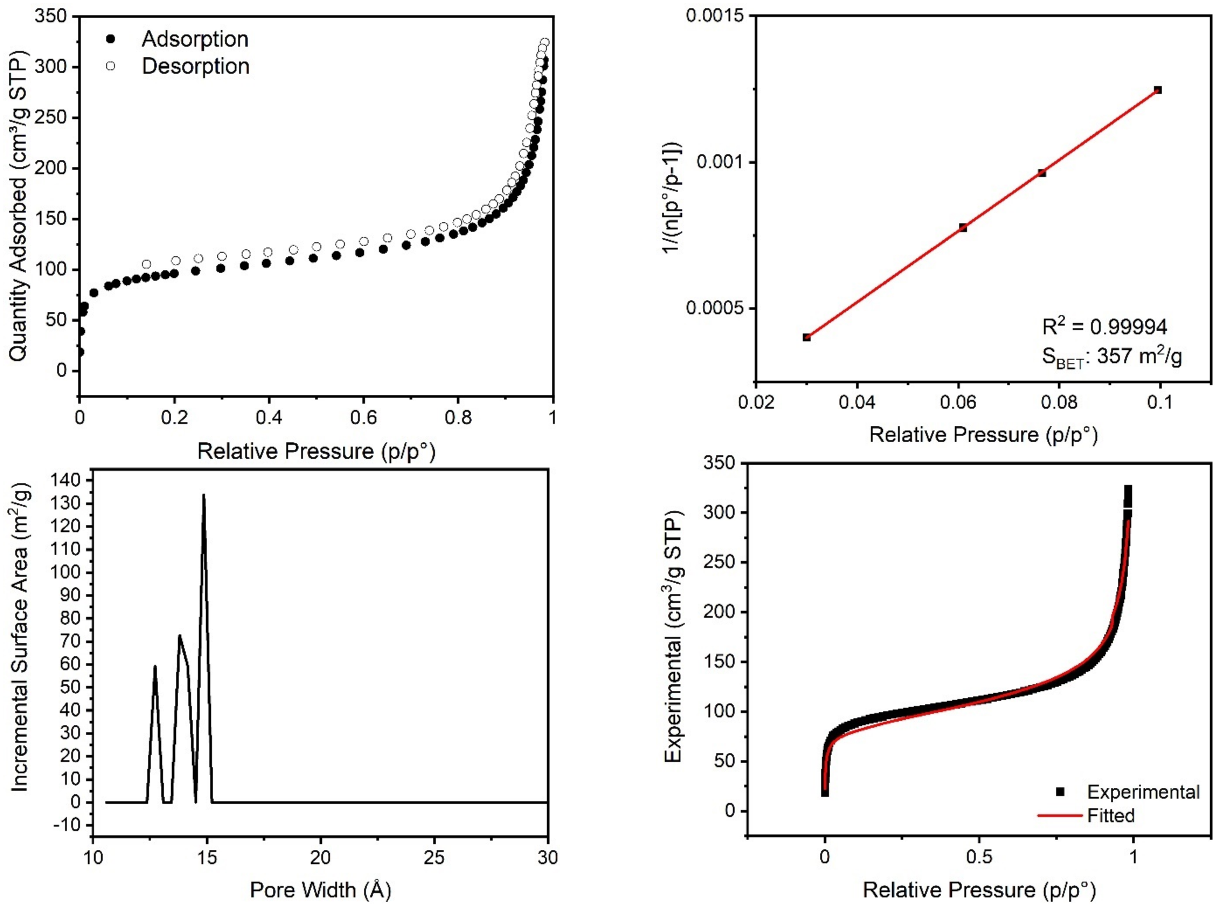

Figure S101: Adsorption measurements for **TpPa** synthesized with 1 equivalent of propionic acid. Top left: adsorption and desorption isotherm. Top right: linear fit to calculate the BET surface area, including R^2. Bottom left: pore size distribution. Bottom right: comparison of the experimental adsorption isotherm with the theoretically modelled isotherm.

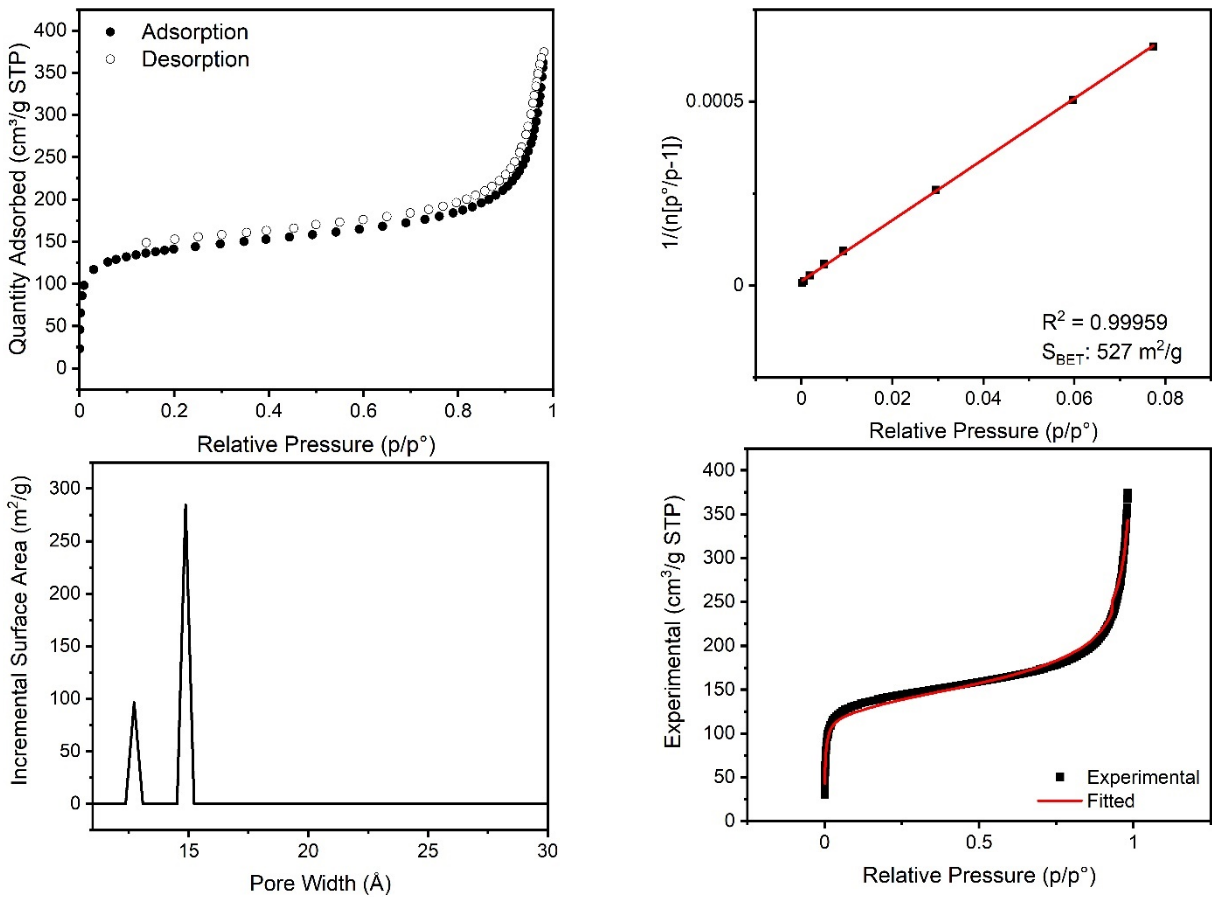

Figure S102: Adsorption measurements for **TpPa** synthesized with 2 equivalents of propionic acid. Top left: adsorption and desorption isotherm. Top right: linear fit to calculate the BET surface area, including R^2. Bottom left: pore size distribution. Bottom right: comparison of the experimental adsorption isotherm with the theoretically modelled isotherm.

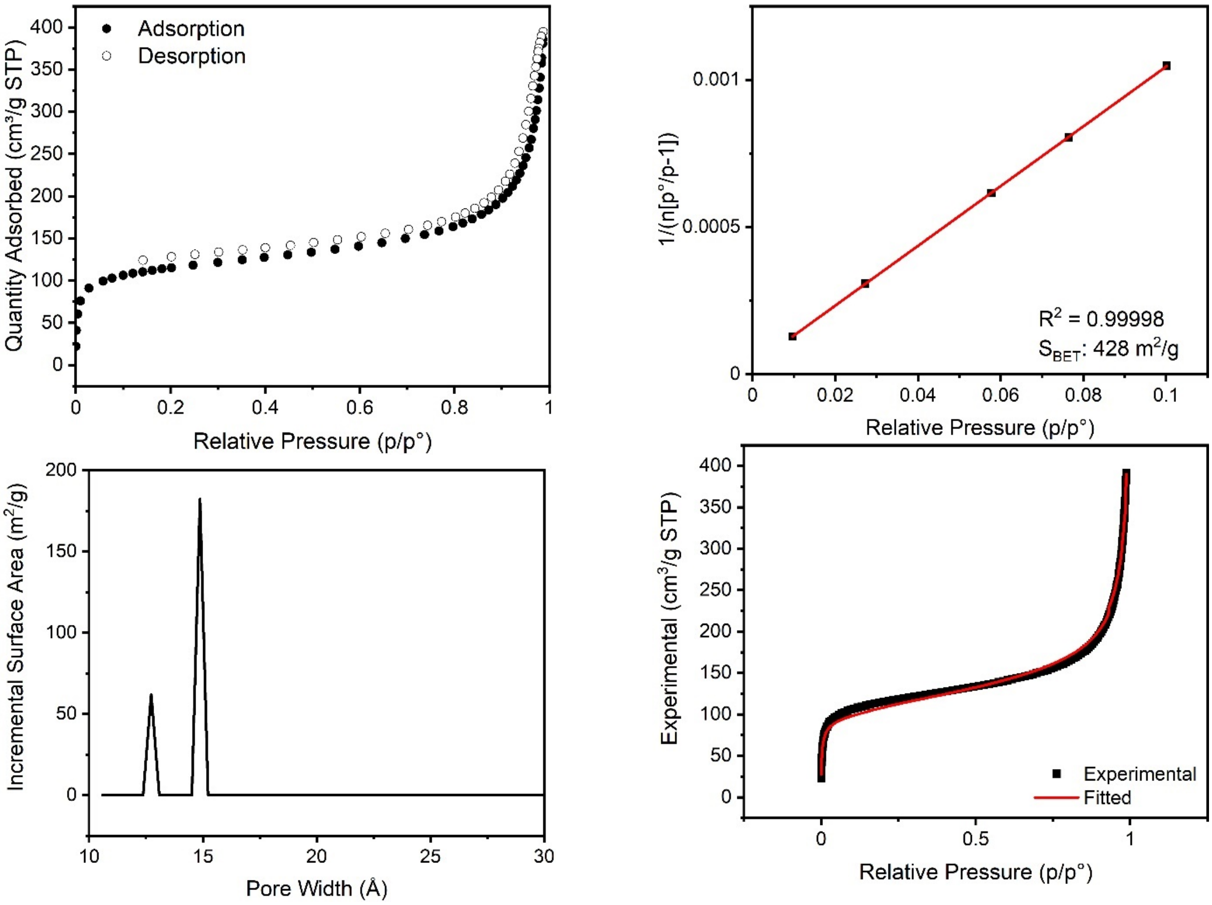

Figure S103: Adsorption measurements for **TpPa** synthesized with 10 equivalents of propionic acid. Top left: adsorption and desorption isotherm. Top right: linear fit to calculate the BET surface area, including R^2. Bottom left: pore size distribution. Bottom right: comparison of the experimental adsorption isotherm with the theoretically modelled isotherm.

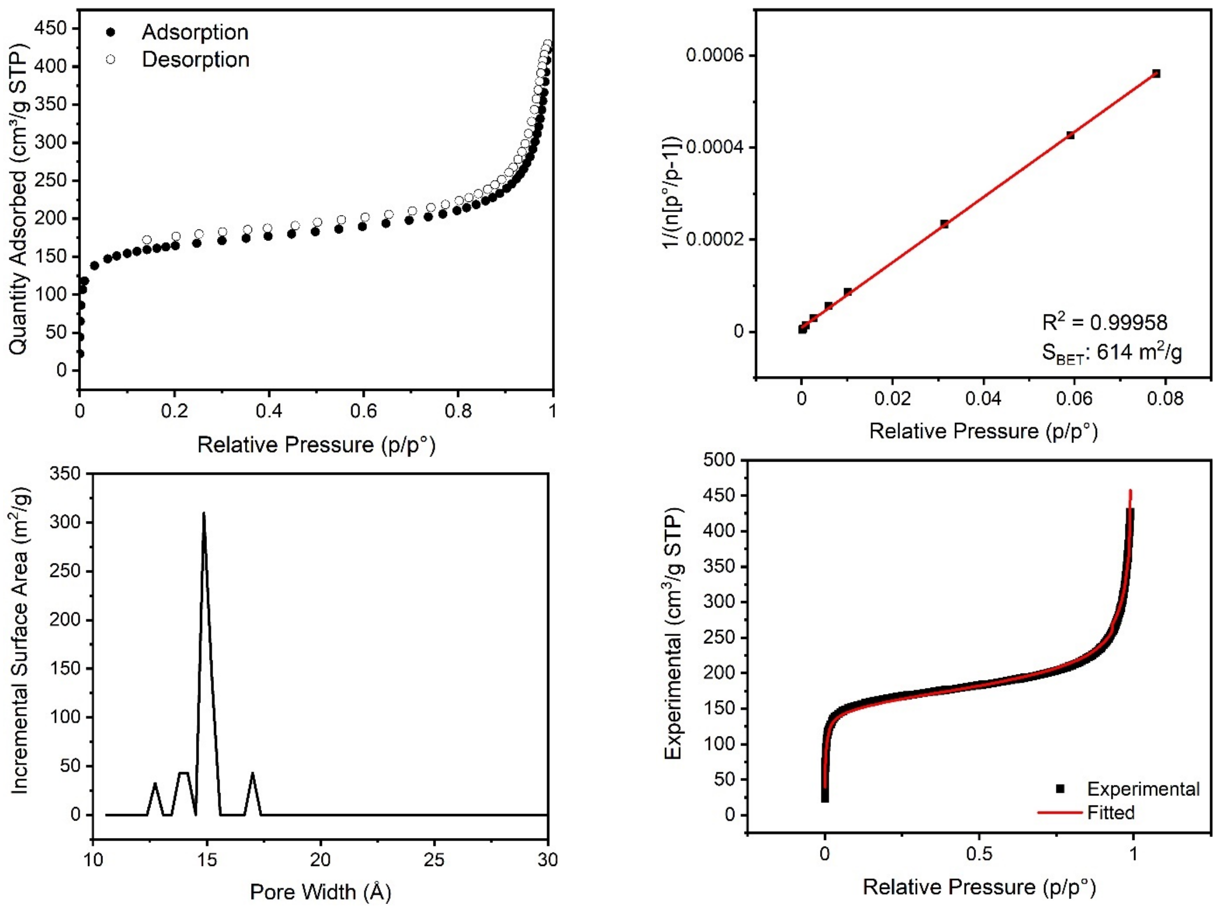

Figure S104: Adsorption measurements for **TpPa** synthesized with 20 equivalents of propionic acid. Top left: adsorption and desorption isotherm. Top right: linear fit to calculate the BET surface area, including R^2. Bottom left: pore size distribution. Bottom right: comparison of the experimental adsorption isotherm with the theoretically modelled isotherm.

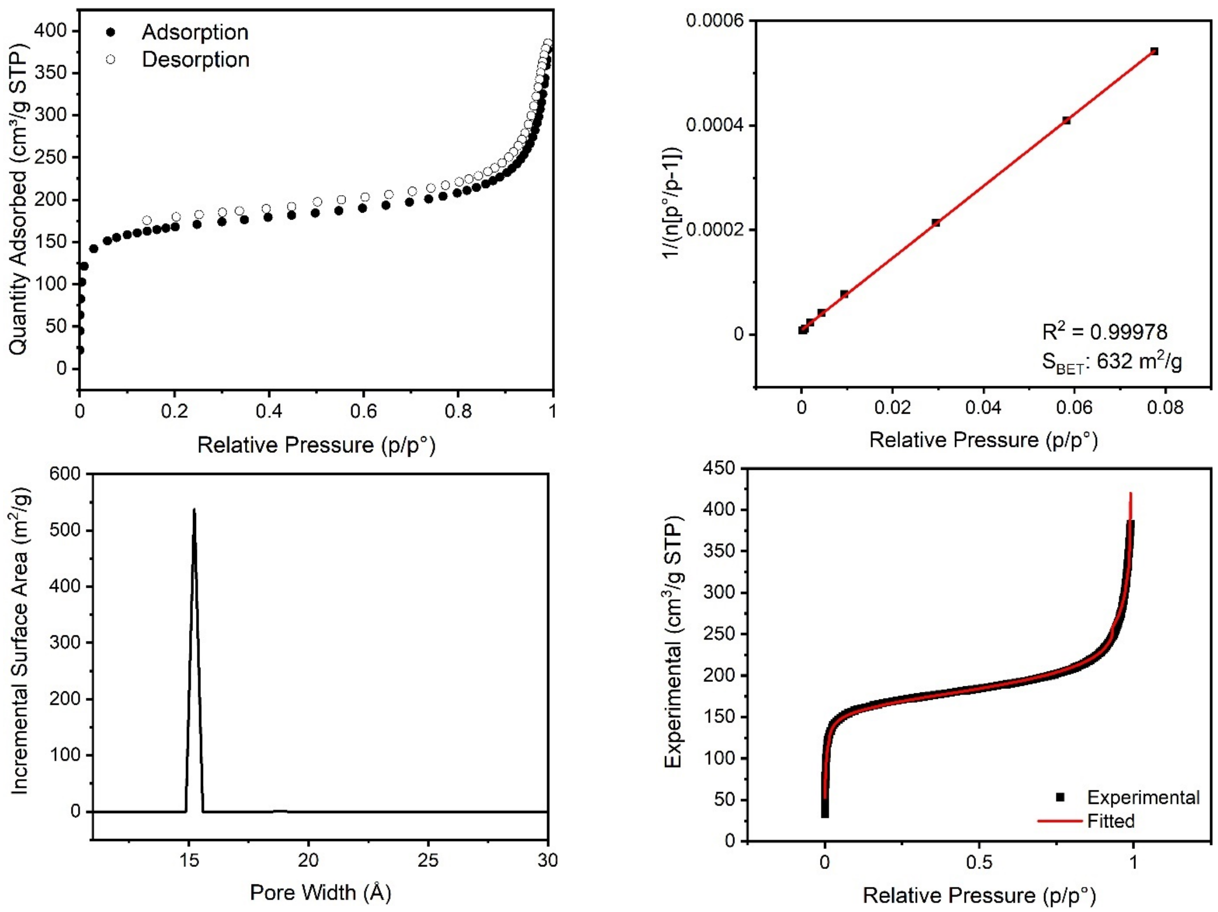

Figure S105: Adsorption measurements for **TpPa** synthesized with 40 equivalents of propionic acid. Top left: adsorption and desorption isotherm. Top right: linear fit to calculate the BET surface area, including R^2. Bottom left: pore size distribution. Bottom right: comparison of the experimental adsorption isotherm with the theoretically modelled isotherm.

6.5 No acid/base

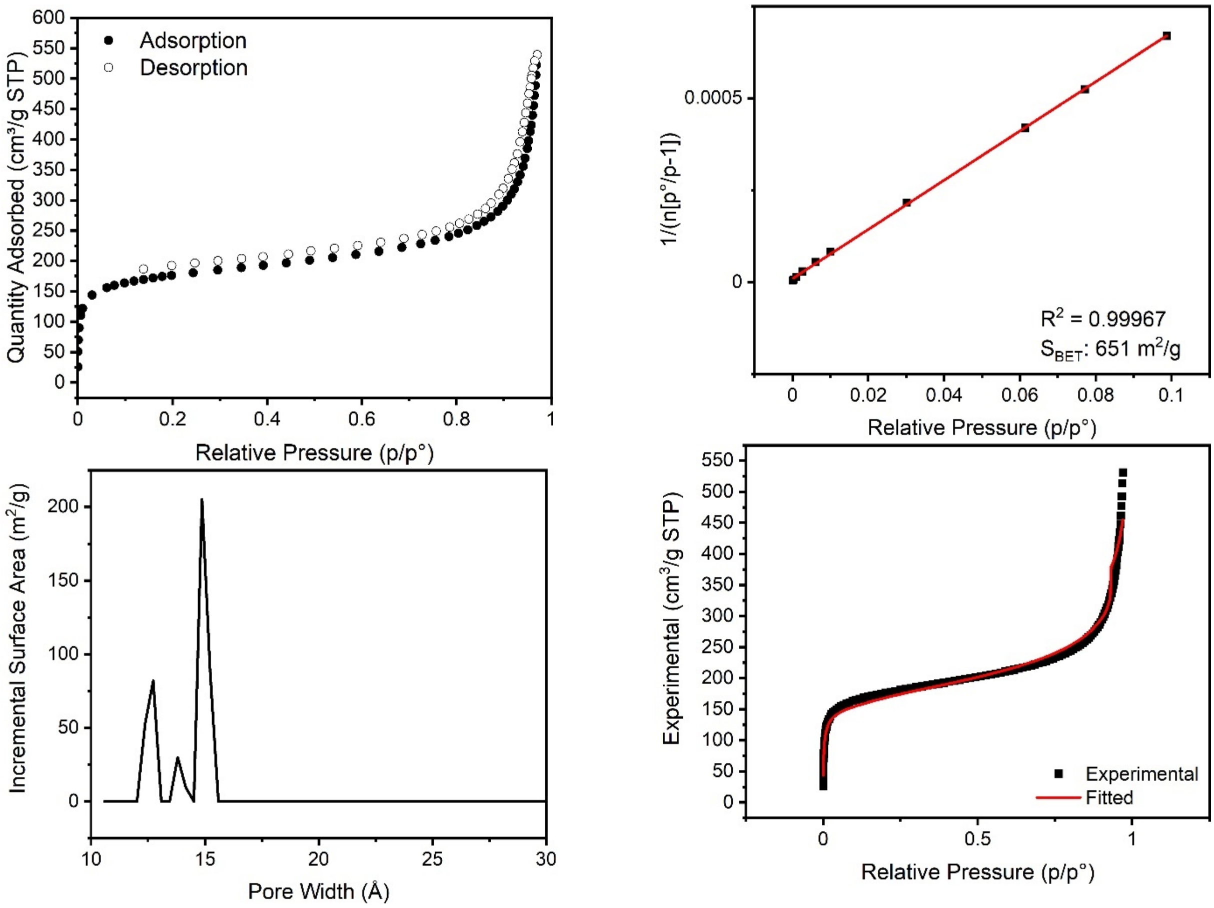

Figure S106: Adsorption measurements for **TpPa** synthesized without added acid or base, sample 1 of 6. Top left: adsorption and desorption isotherm. Top right: linear fit to calculate the BET surface area, including R^2. Bottom left: pore size distribution. Bottom right: comparison of the experimental adsorption isotherm with the theoretically modelled isotherm.

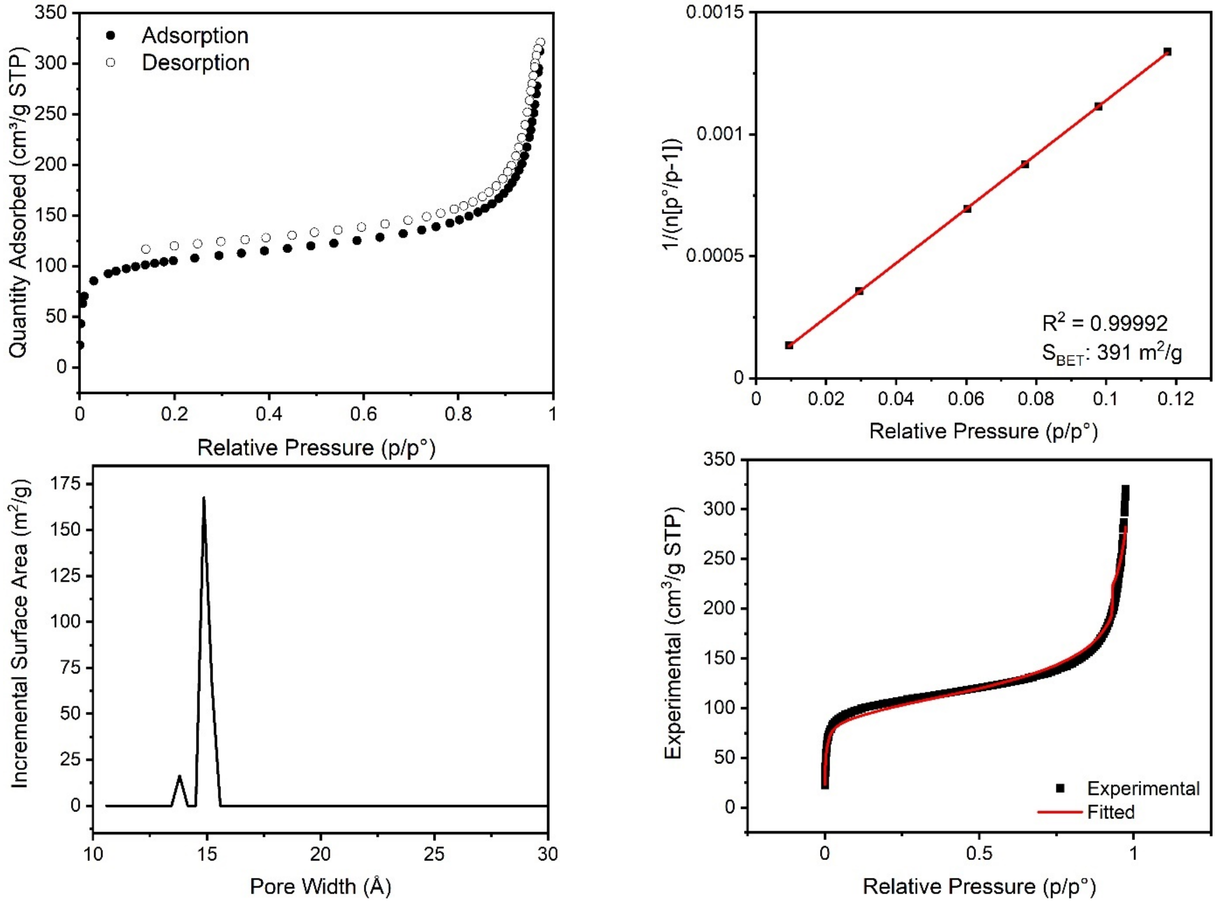

Figure S107: Adsorption measurements for **TpPa** synthesized without added acid or base, sample 2 of 6. Top left: adsorption and desorption isotherm. Top right: linear fit to calculate the BET surface area, including R^2. Bottom left: pore size distribution. Bottom right: comparison of the experimental adsorption isotherm with the theoretically modelled isotherm.

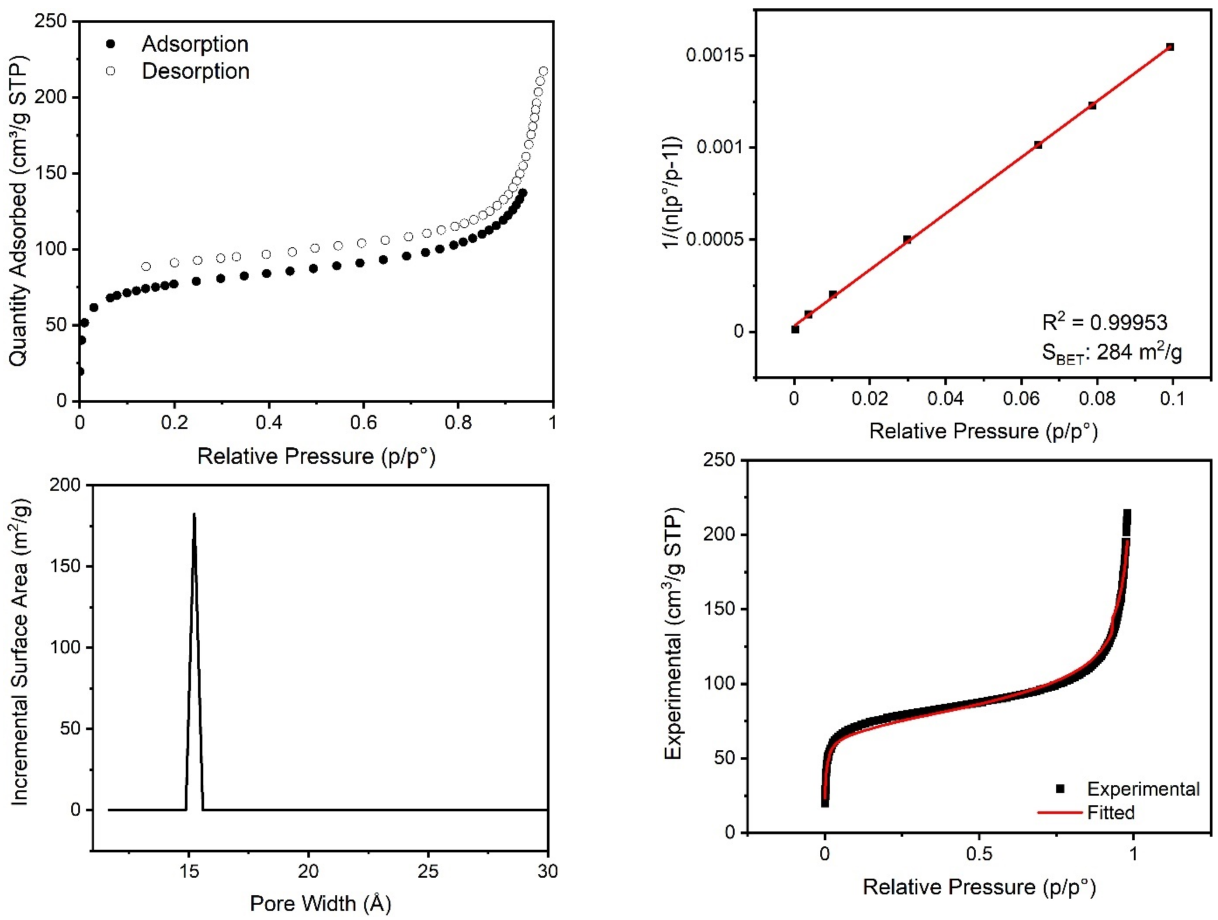

Figure S108: Adsorption measurements for **TpPa** synthesized without added acid or base, sample 3 of 6. Top left: adsorption and desorption isotherm. Top right: linear fit to calculate the BET surface area, including R^2. Bottom left: pore size distribution. Bottom right: comparison of the experimental adsorption isotherm with the theoretically modelled isotherm.

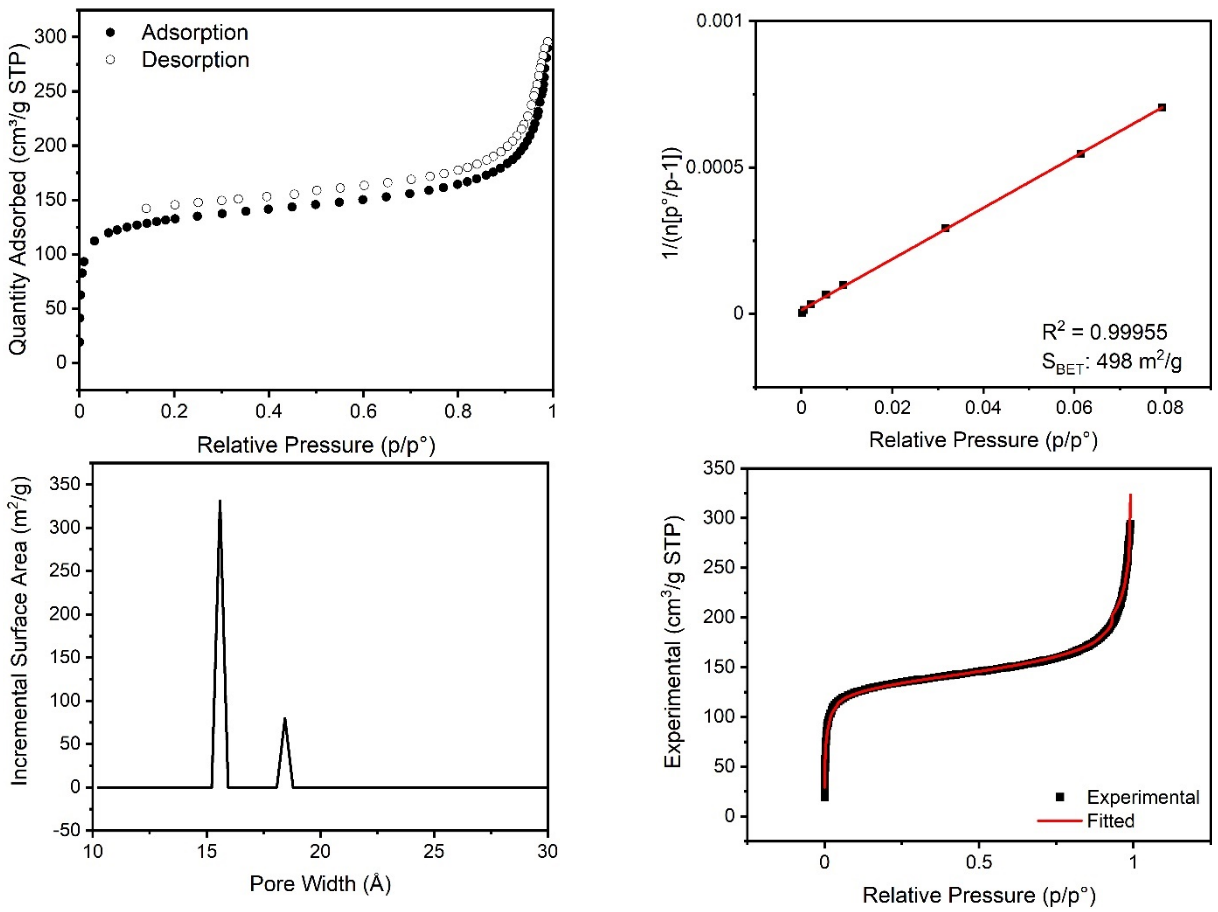

Figure S109: Adsorption measurements for **TpPa** synthesized without added acid or base, sample 4 of 6. Top left: adsorption and desorption isotherm. Top right: linear fit to calculate the BET surface area, including R^2. Bottom left: pore size distribution. Bottom right: comparison of the experimental adsorption isotherm with the theoretically modelled isotherm.

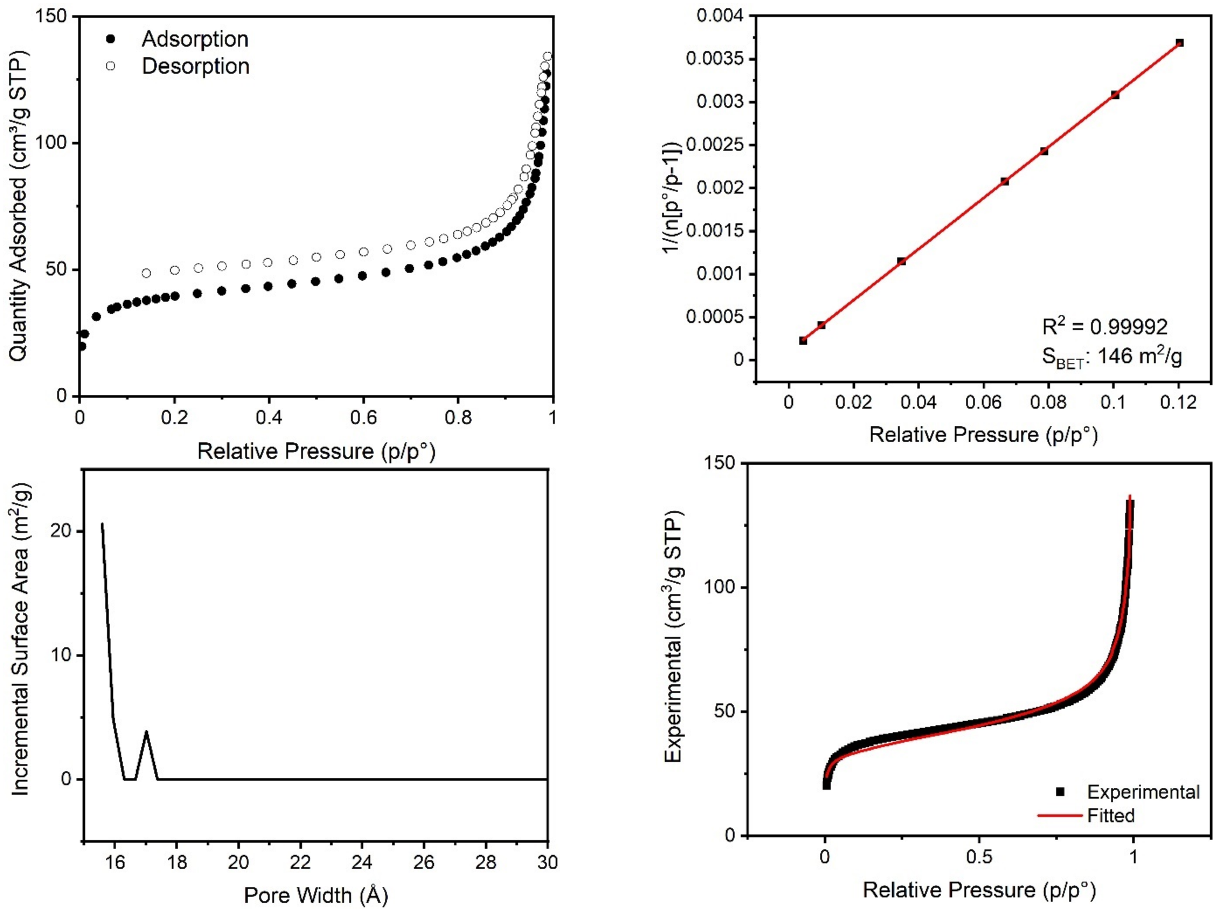

Figure S110: Adsorption measurements for **TpPa** synthesized without added acid or base, sample 5 of 6. Top left: adsorption and desorption isotherm. Top right: linear fit to calculate the BET surface area, including R^2. Bottom left: pore size distribution. Bottom right: comparison of the experimental adsorption isotherm with the theoretically modelled isotherm. No good DFT fit was found.

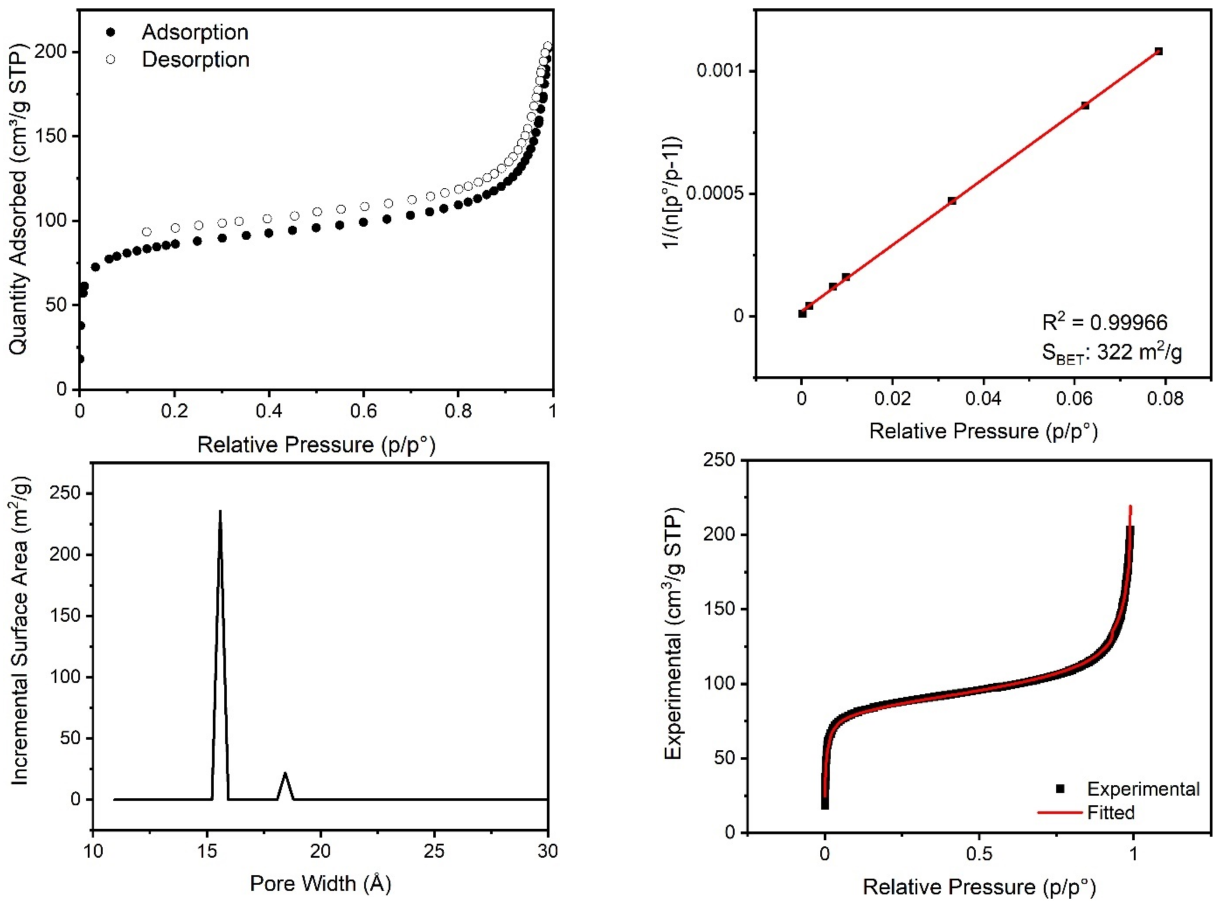

Figure S111: Adsorption measurements for **TpPa** synthesized without added acid or base, sample 6 of 6. Top left: adsorption and desorption isotherm. Top right: linear fit to calculate the BET surface area, including R^2. Bottom left: pore size distribution. Bottom right: comparison of the experimental adsorption isotherm with the theoretically modelled isotherm.

6.6 Pyridine

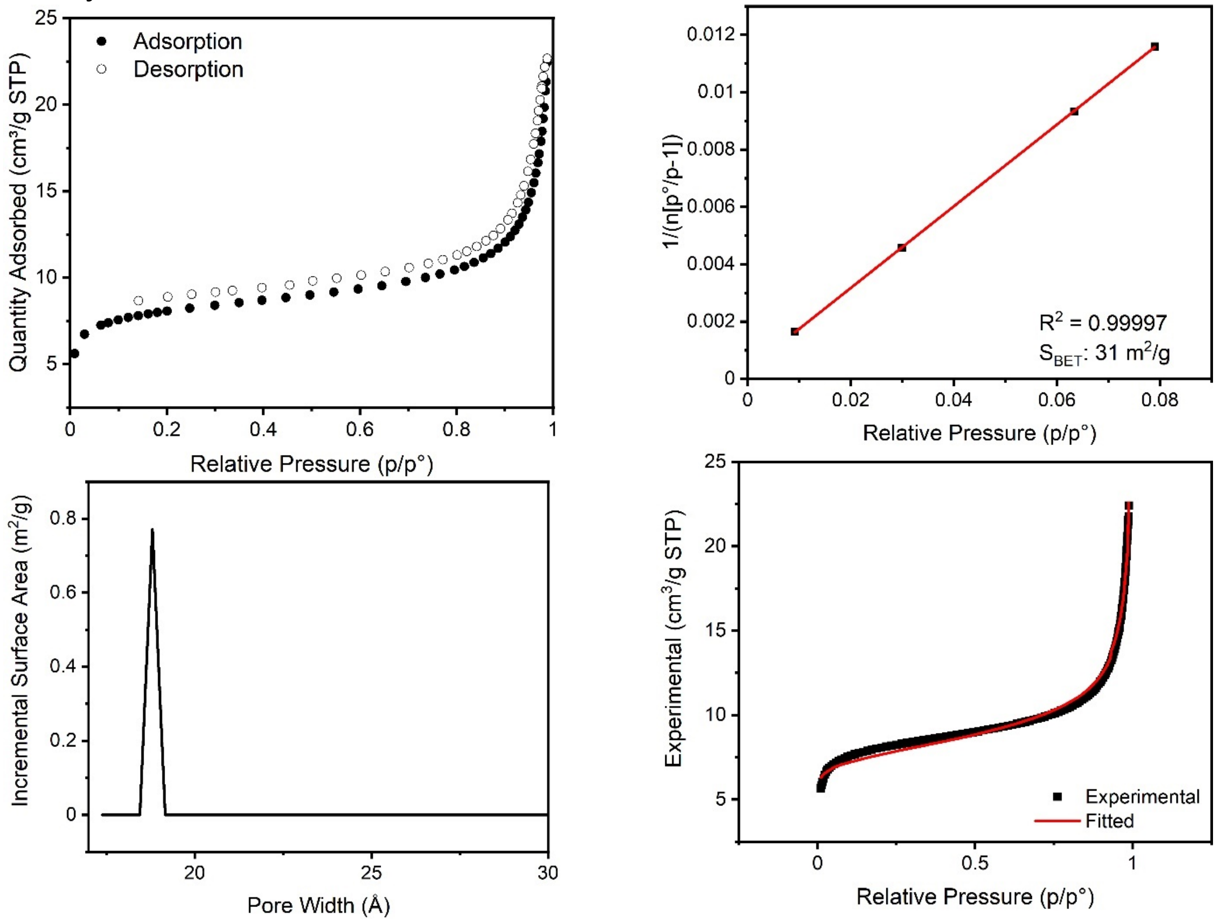

Figure S112: Adsorption measurements for **TpPa** synthesized with ⅓ equivalents of pyridine. Top left: adsorption and desorption isotherm. Top right: linear fit to calculate the BET surface area, including R^2. Bottom left: pore size distribution. Bottom right: comparison of the experimental adsorption isotherm with the theoretically modelled isotherm.

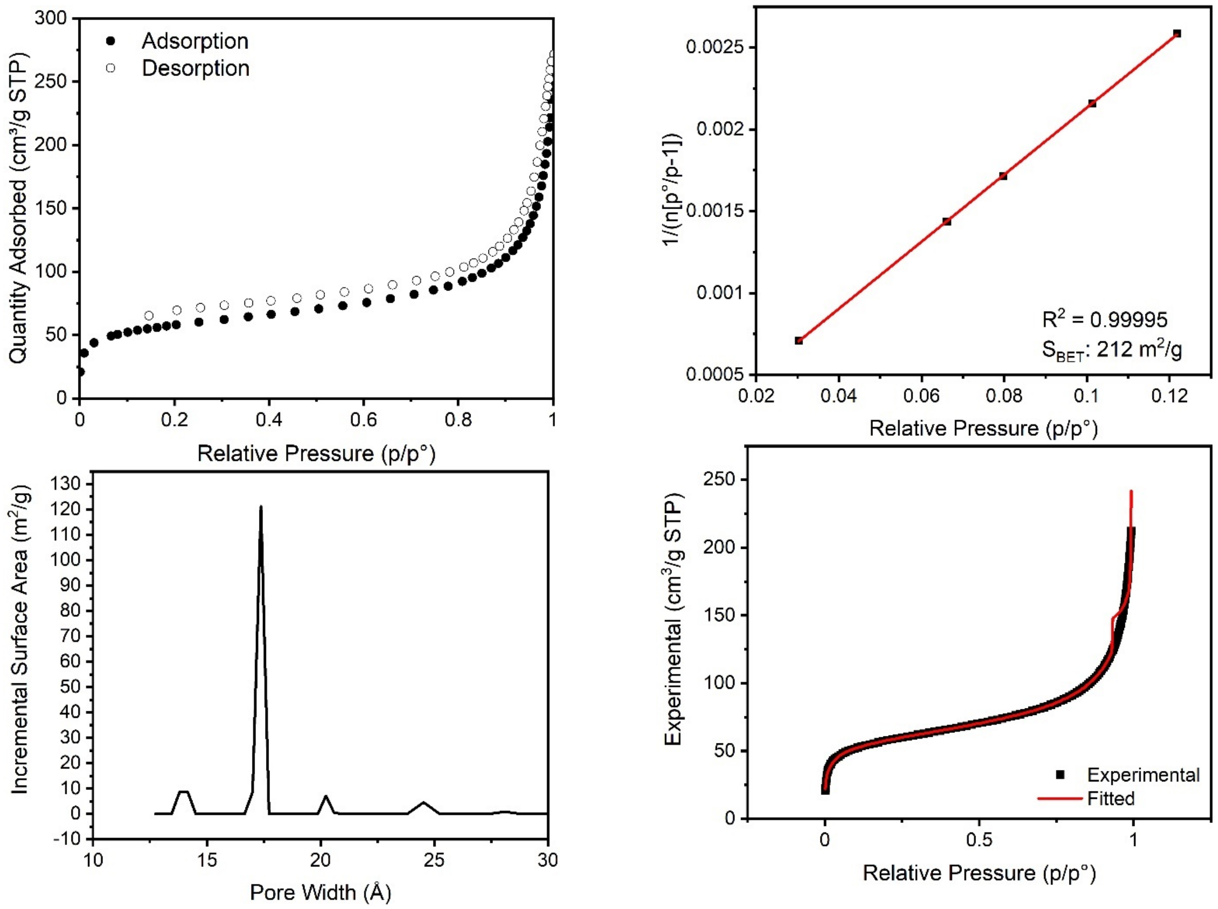

Figure S113: Adsorption measurements for **TpPa** synthesized with 1 equivalent of pyridine. Top left: adsorption and desorption isotherm. Top right: linear fit to calculate the BET surface area, including R^2. Bottom left: pore size distribution. Bottom right: comparison of the experimental adsorption isotherm with the theoretically modelled isotherm.

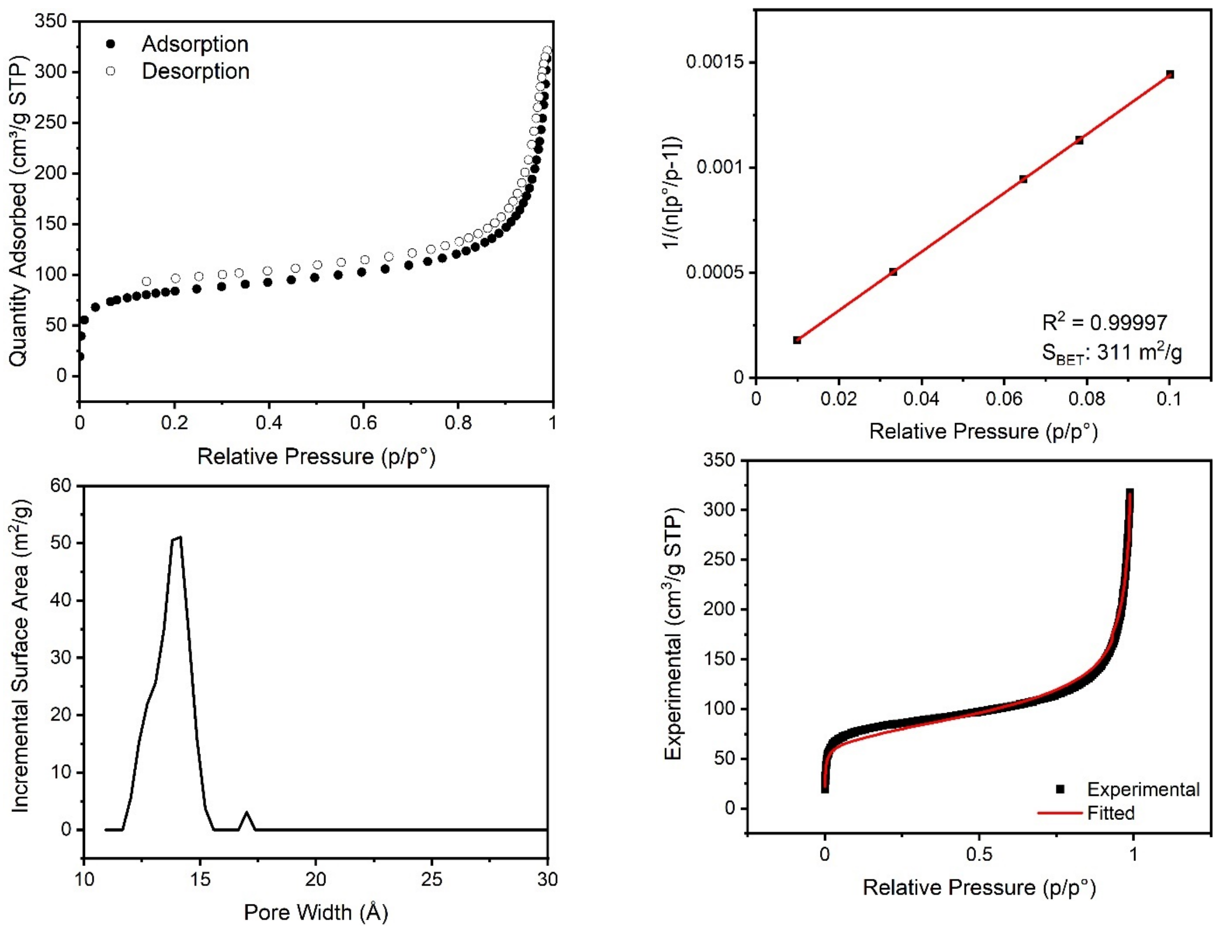

Figure S114: Adsorption measurements for **TpPa** synthesized with 2 equivalents of pyridine. Top left: adsorption and desorption isotherm. Top right: linear fit to calculate the BET surface area, including R^2. Bottom left: pore size distribution. Bottom right: comparison of the experimental adsorption isotherm with the theoretically modelled isotherm.

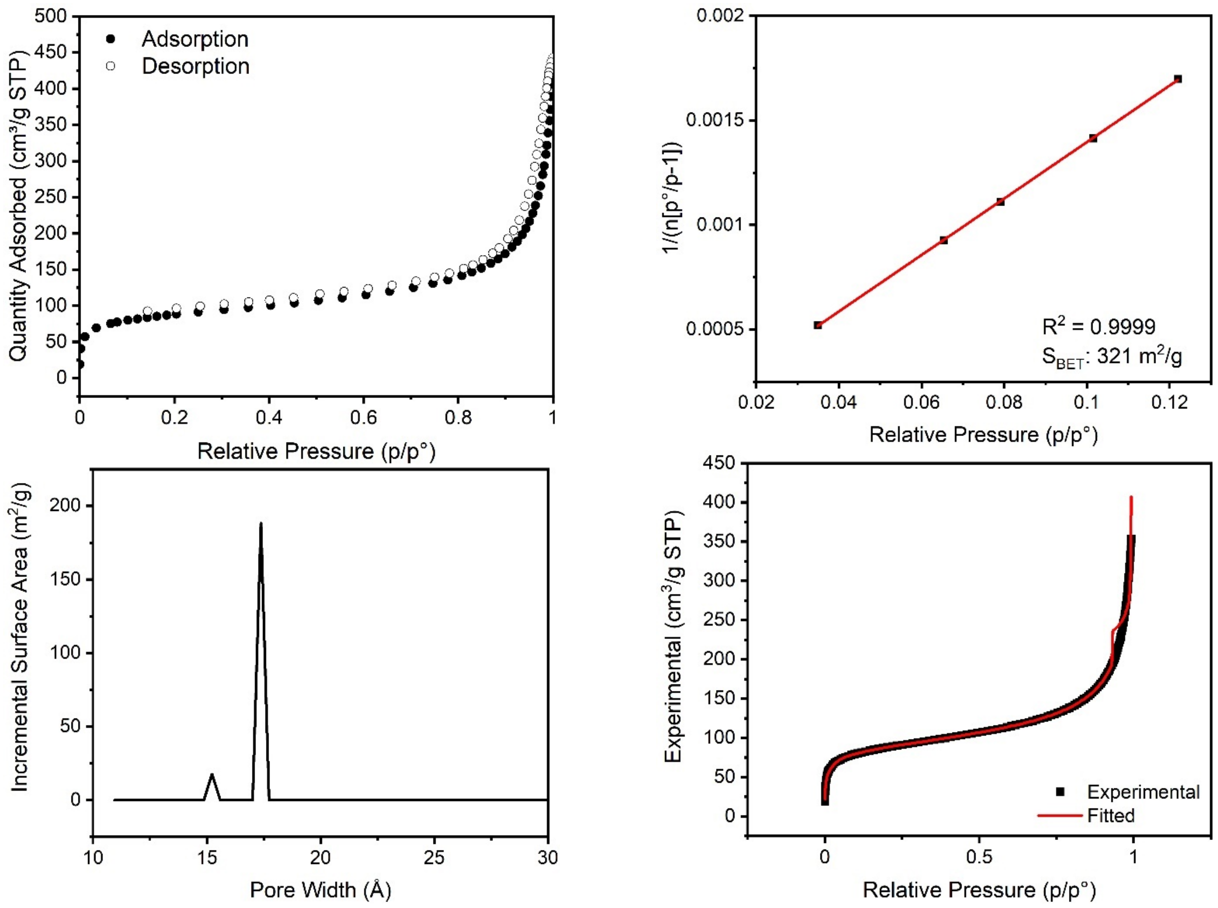

Figure S115: Adsorption measurements for **TpPa** synthesized with 10 equivalents pyridine. Top left: adsorption and desorption isotherm. Top right: linear fit to calculate the BET surface area, including R^2. Bottom left: pore size distribution. Bottom right: comparison of the experimental adsorption isotherm with the theoretically modelled isotherm.

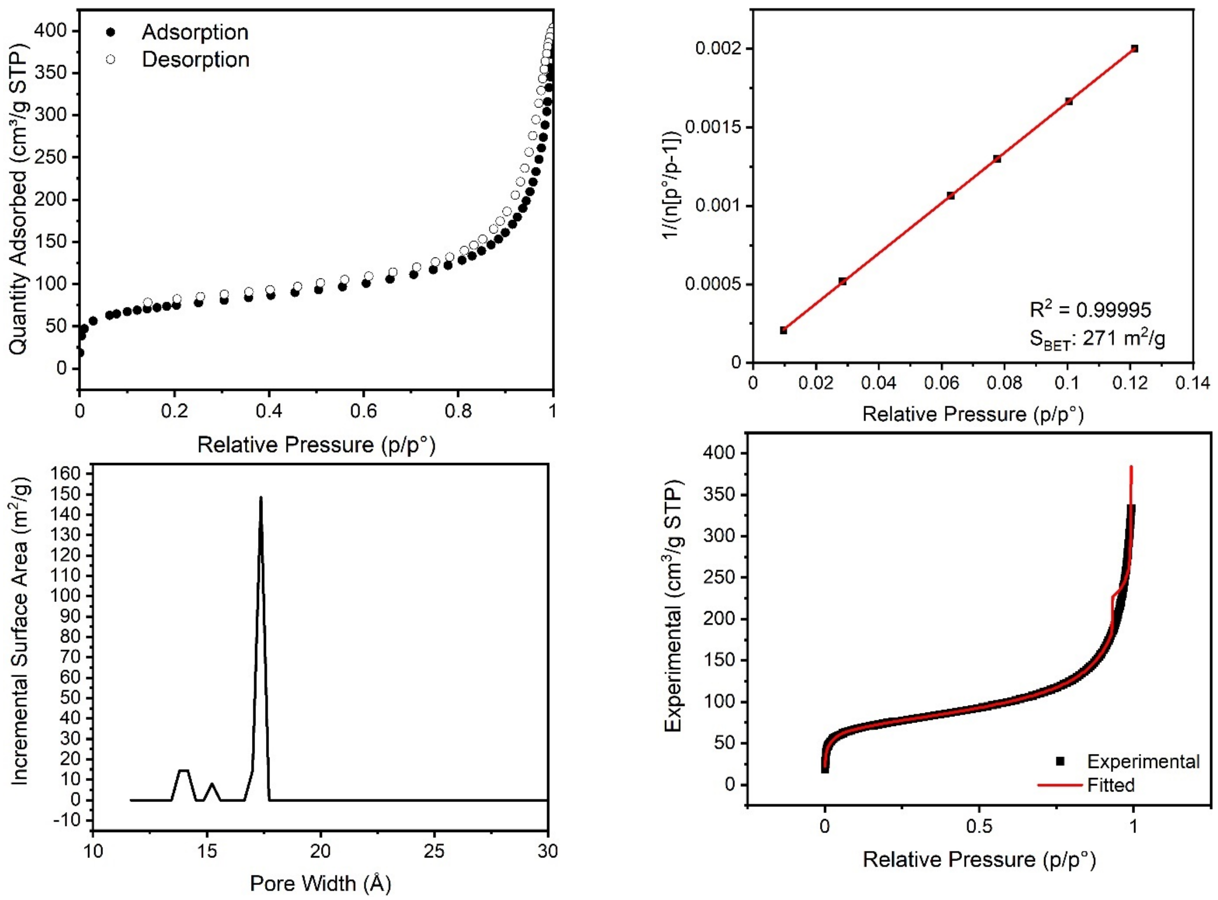

Figure S116: Adsorption measurements for **TpPa** synthesized with 20 equivalents of pyridine. Top left: adsorption and desorption isotherm. Top right: linear fit to calculate the BET surface area, including R^2. Bottom left: pore size distribution. Bottom right: comparison of the experimental adsorption isotherm with the theoretically modelled isotherm.

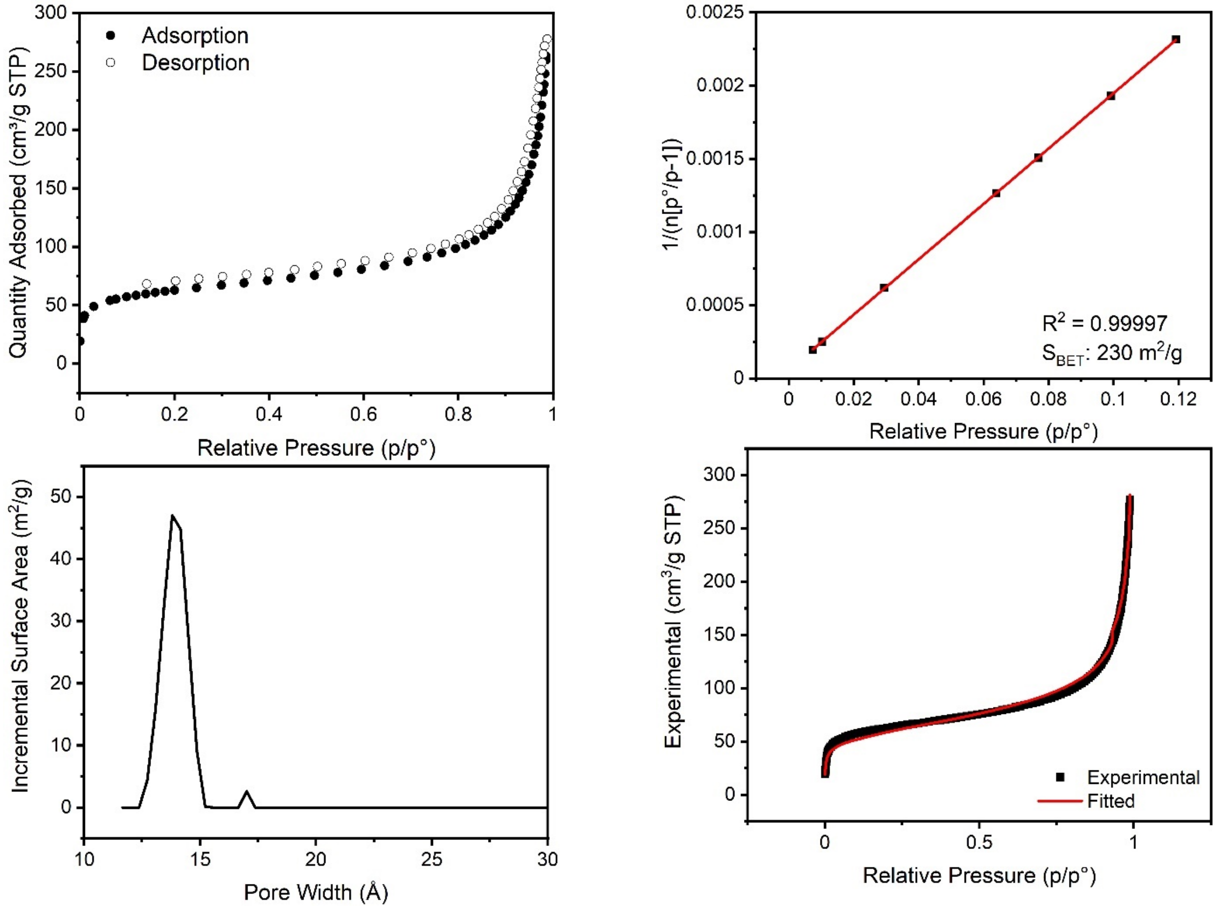

Figure S117: Adsorption measurements for **TpPa** synthesized with 40 equivalents of pyridine. Top left: adsorption and desorption isotherm. Top right: linear fit to calculate the BET surface area, including R^2. Bottom left: pore size distribution. Bottom right: comparison of the experimental adsorption isotherm with the theoretically modelled isotherm.

6.7 2,4,6-trimethylpyridine

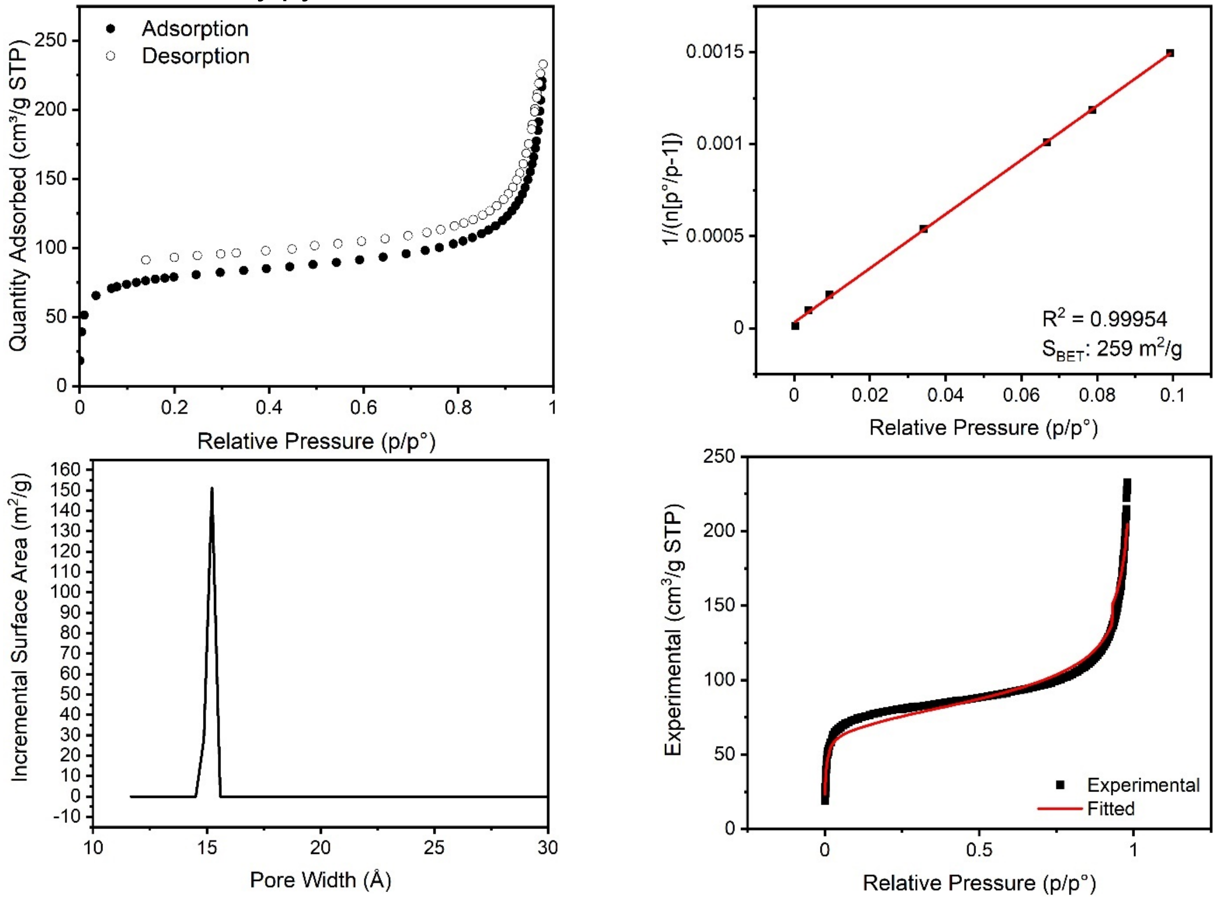

Figure S118: Adsorption measurements for **TpPa** synthesized with ⅓ equivalents of 2,4,6-trimethylpyridine. Top left: adsorption and desorption isotherm. Top right: linear fit to calculate the BET surface area, including R^2. Bottom left: pore size distribution. Bottom right: comparison of the experimental adsorption isotherm with the theoretically modelled isotherm.

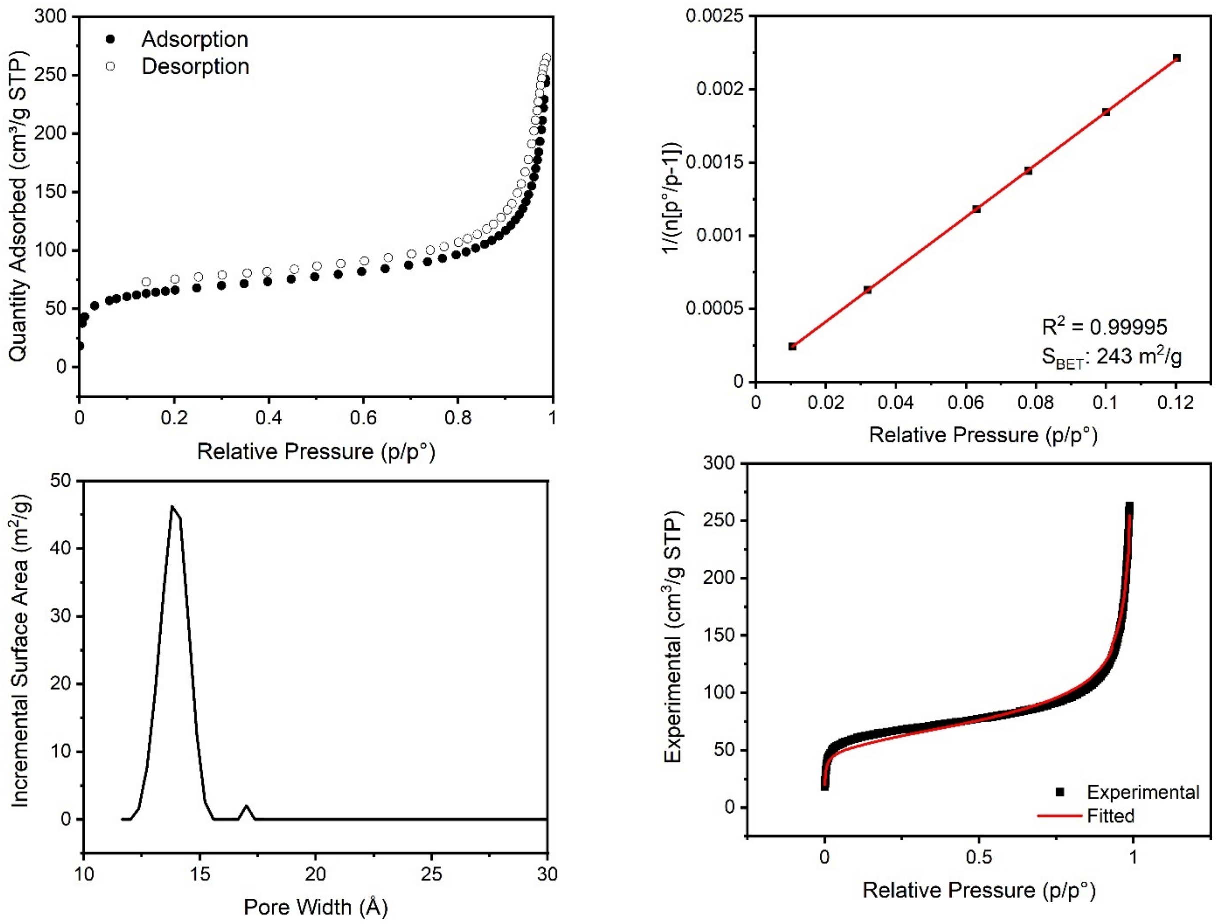

Figure S119: Adsorption measurements for **TpPa** synthesized with 1 equivalent of 2,4,6-trimethylpyridine. Top left: adsorption and desorption isotherm. Top right: linear fit to calculate the BET surface area, including R^2. Bottom left: pore size distribution. Bottom right: comparison of the experimental adsorption isotherm with the theoretically modelled isotherm.

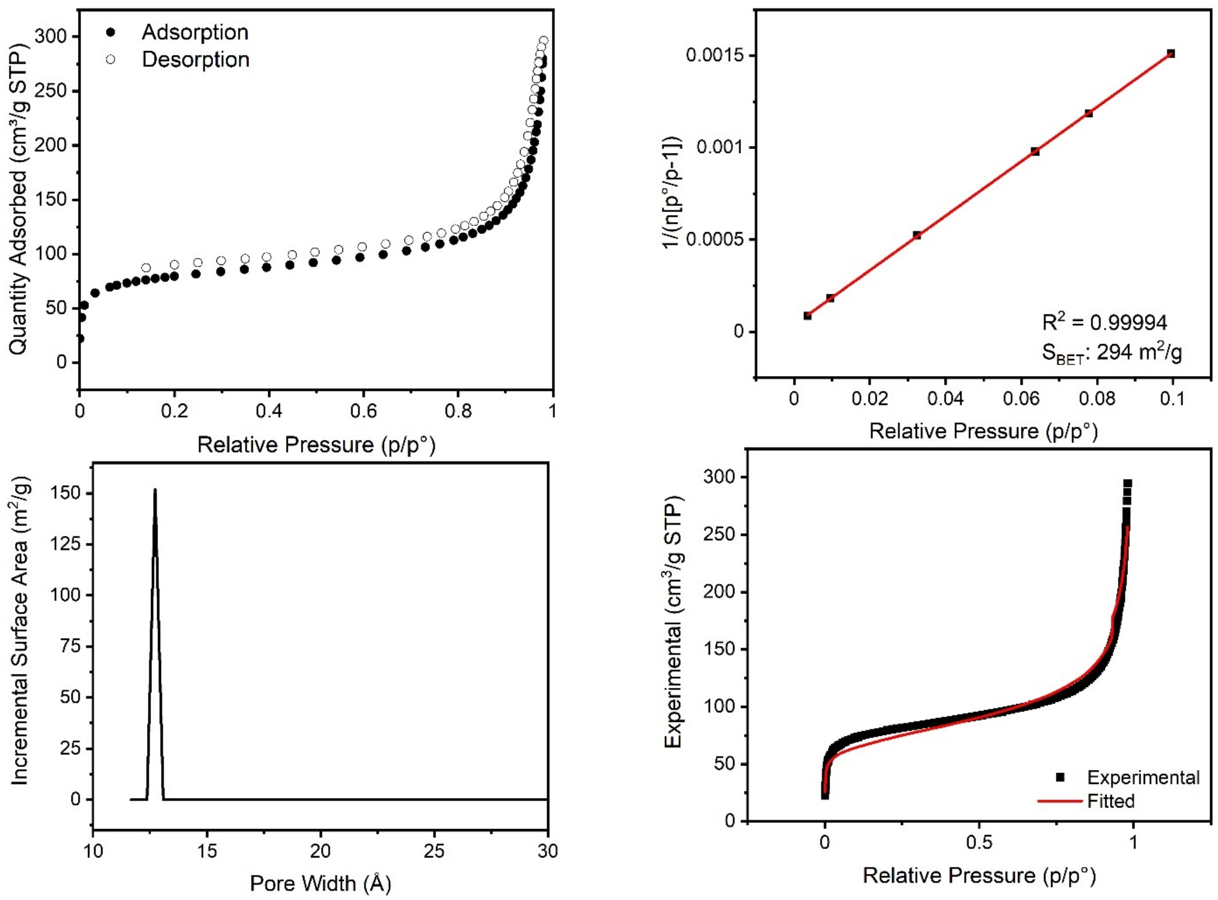

Figure S120: Adsorption measurements for **TpPa** synthesized with 2 equivalents of 2,4,6-trimethylpyridine. Top left: adsorption and desorption isotherm. Top right: linear fit to calculate the BET surface area, including R^2. Bottom left: pore size distribution. Bottom right: comparison of the experimental adsorption isotherm with the theoretically modelled isotherm.

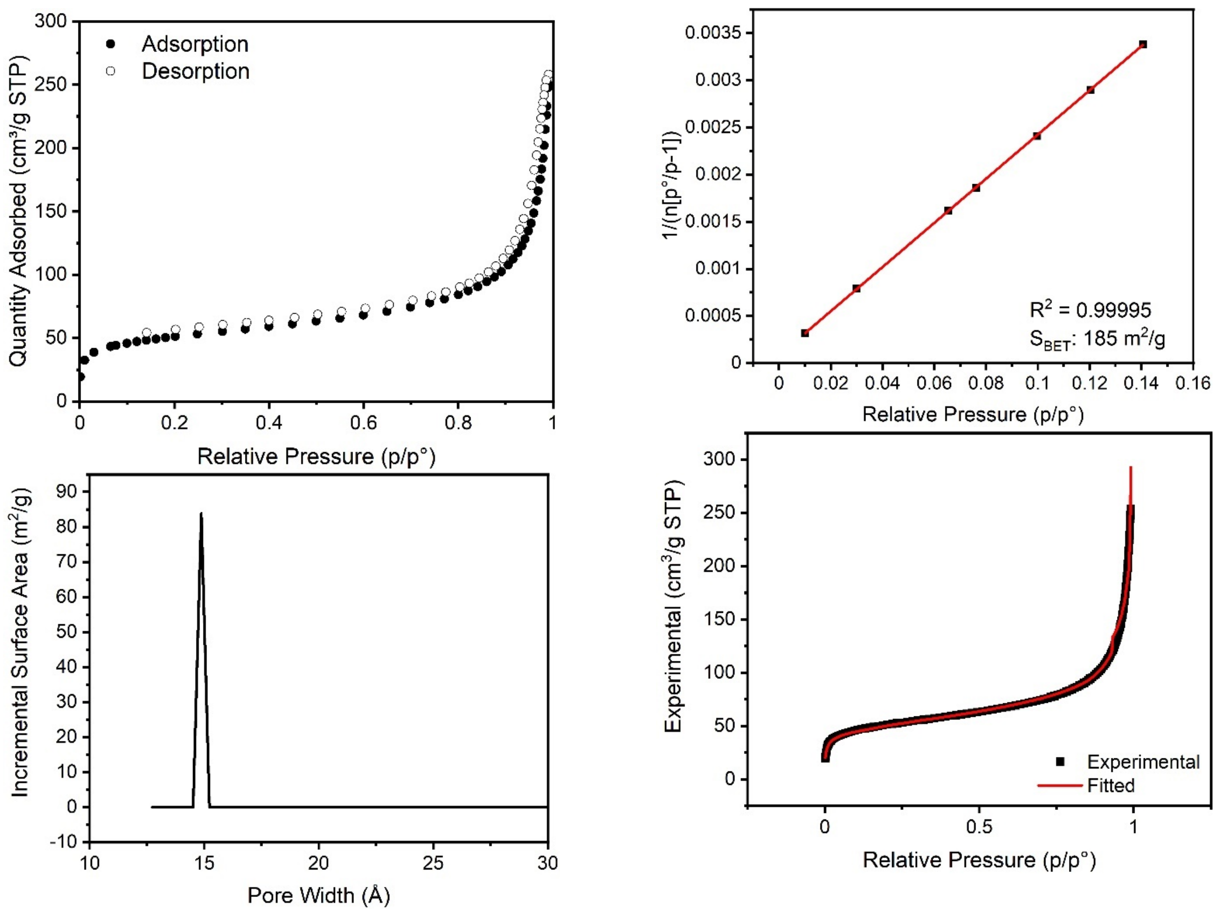

Figure S121: Adsorption measurements for **TpPa** synthesized with 10 equivalents of 2,4,6-trimethylpyridine. Top left: adsorption and desorption isotherm. Top right: linear fit to calculate the BET surface area, including R^2. Bottom left: pore size distribution. Bottom right: comparison of the experimental adsorption isotherm with the theoretically modelled isotherm.

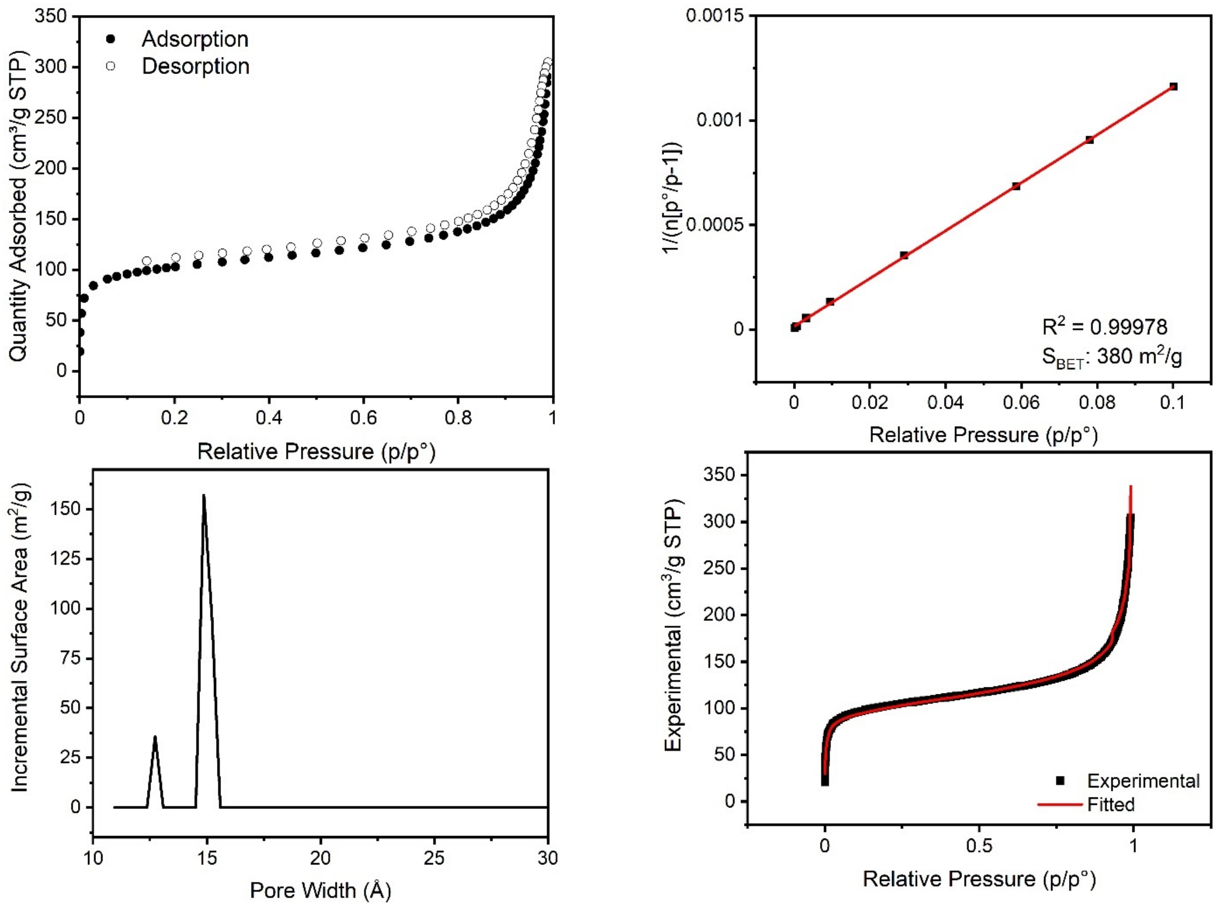

Figure S122: Adsorption measurements for **TpPa** synthesized with 20 equivalents of 2,4,6-trimethylpyridine. Top left: adsorption and desorption isotherm. Top right: linear fit to calculate the BET surface area, including R^2. Bottom left: pore size distribution. Bottom right: comparison of the experimental adsorption isotherm with the theoretically modelled isotherm.

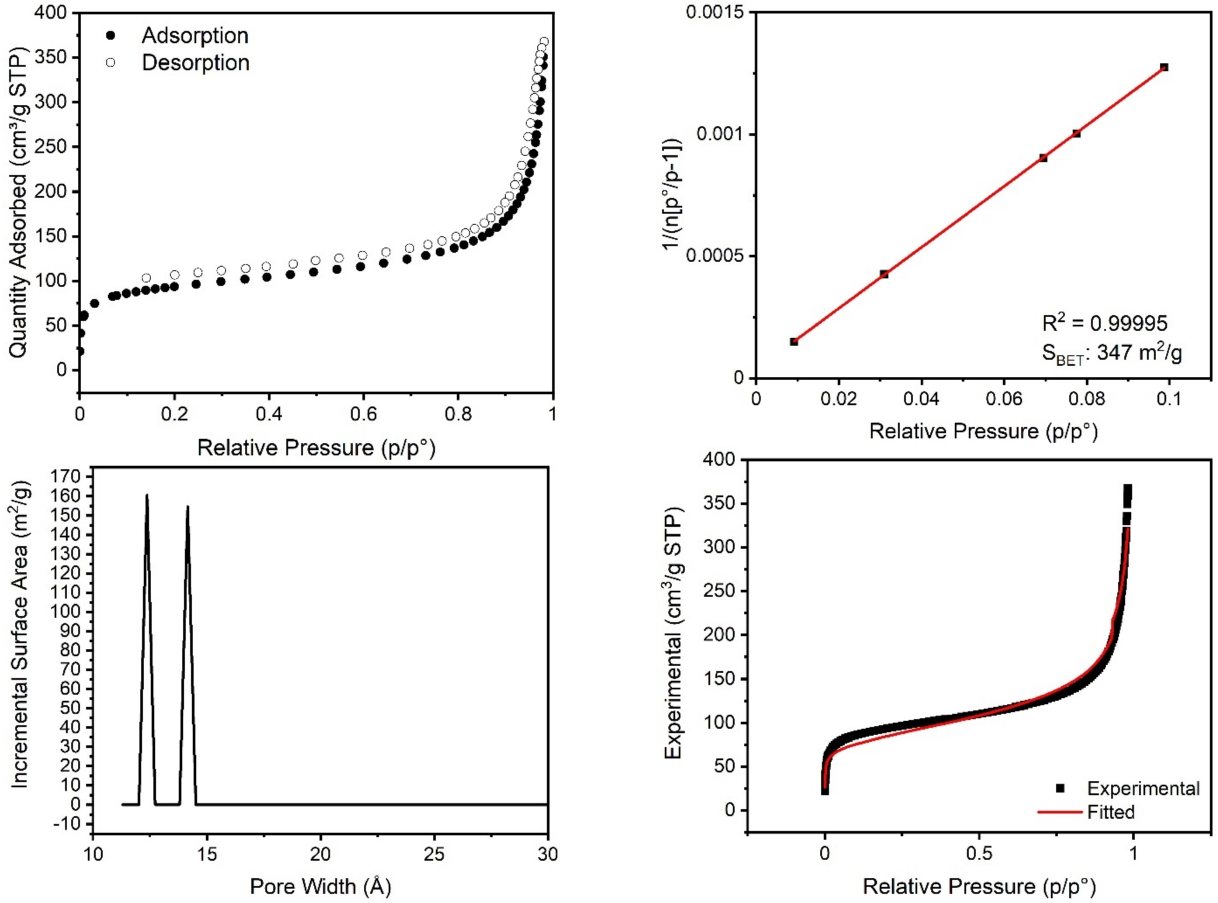

Figure S123: Adsorption measurements for **TpPa** synthesized with 40 equivalents of 2,4,6-trimethylpyridine. Top left: adsorption and desorption isotherm. Top right: linear fit to calculate the BET surface area, including R^2. Bottom left: pore size distribution. Bottom right: comparison of the experimental adsorption isotherm with the theoretically modelled isotherm.

6.8 TEA

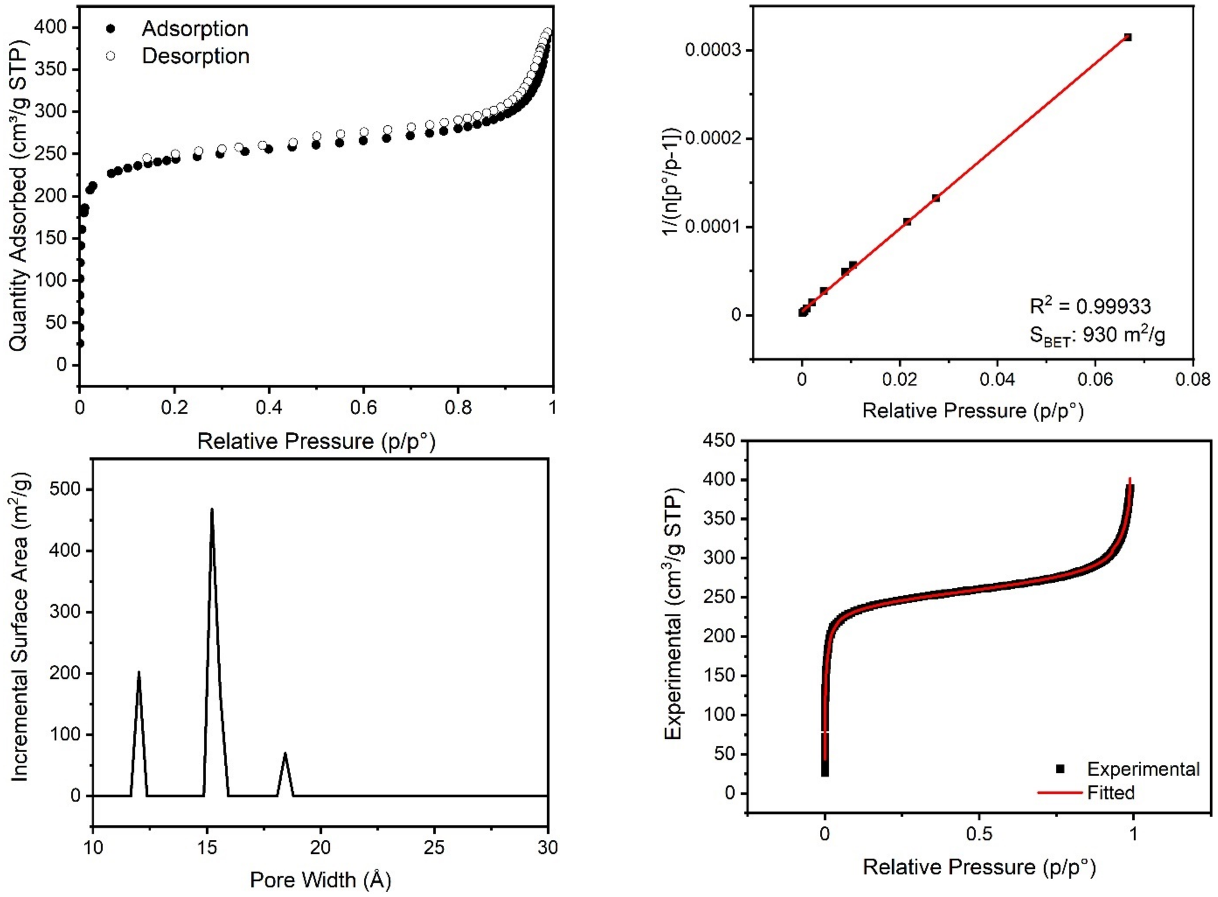

Figure S124: Adsorption measurements for **TpPa** synthesized with ⅓ equivalents of TEA. Top left: adsorption and desorption isotherm. Top right: linear fit to calculate the BET surface area, including R^2. Bottom left: pore size distribution. Bottom right: comparison of the experimental adsorption isotherm with the theoretically modelled isotherm.

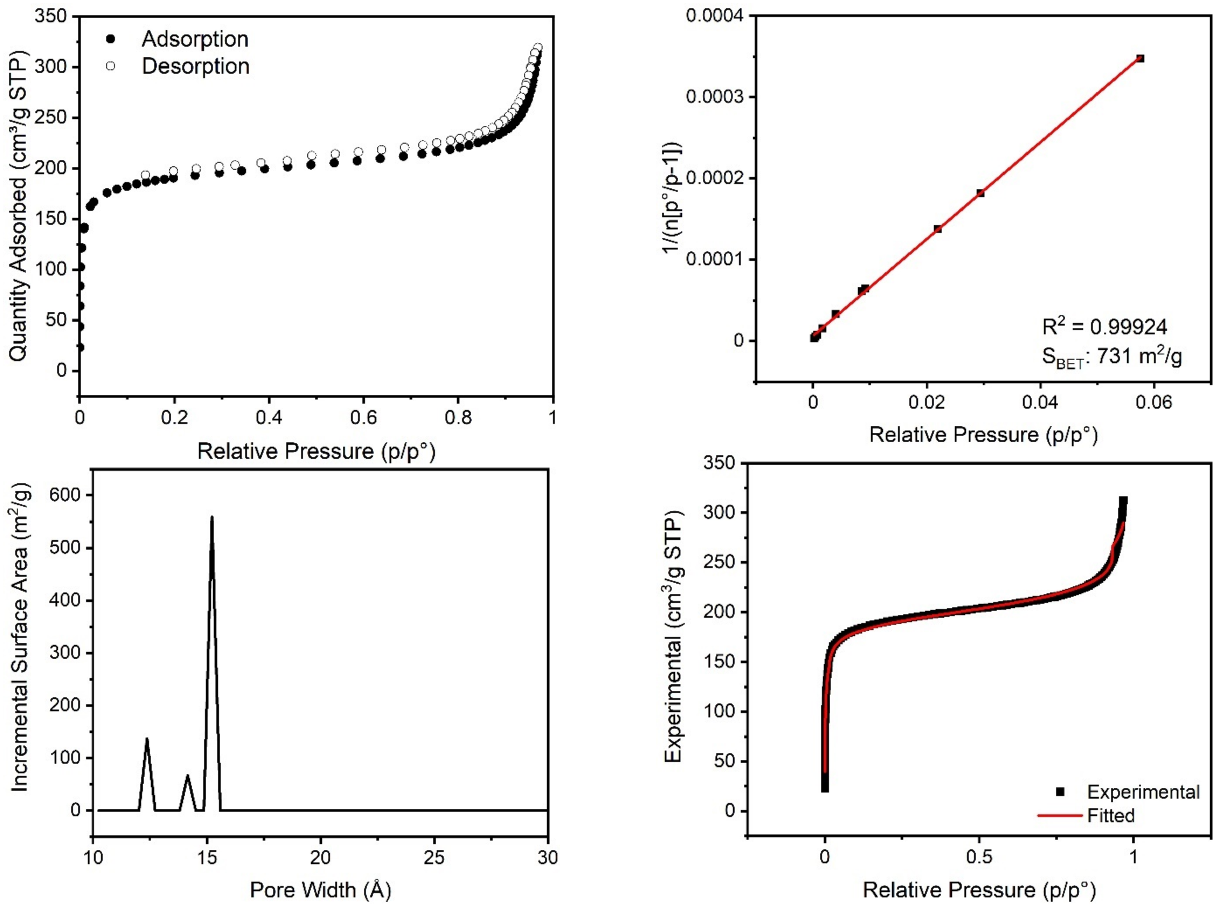

Figure S125: Adsorption measurements for **TpPa** synthesized with ⅓ equivalents of TEA (duplicate synthesis). Top left: adsorption and desorption isotherm. Top right: linear fit to calculate the BET surface area, including R^2. Bottom left: pore size distribution. Bottom right: comparison of the experimental adsorption isotherm with the theoretically modelled isotherm.

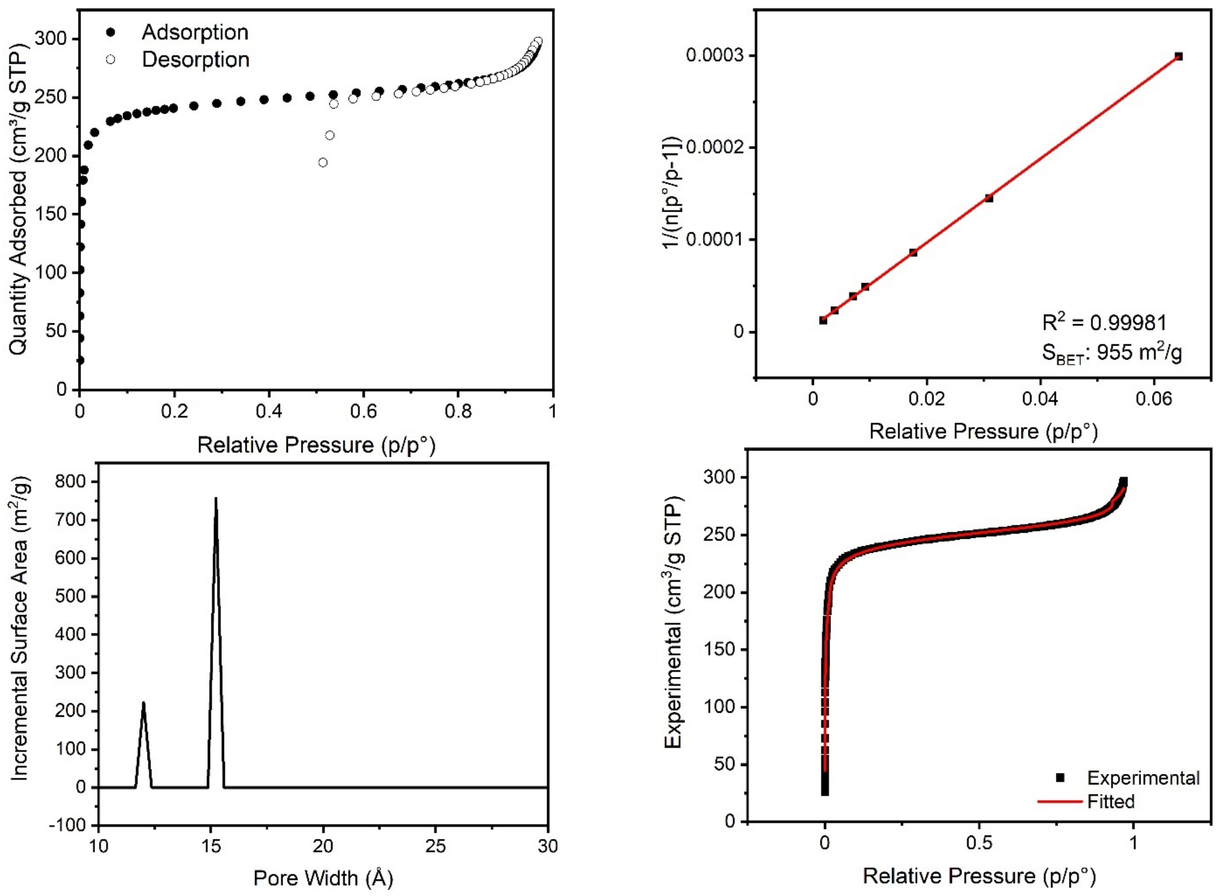

Figure S126: Adsorption measurements for **TpPa** synthesized with 1 equivalent of TEA. Top left: adsorption and desorption isotherm. Top right: linear fit to calculate the BET surface area, including R^2. Bottom left: pore size distribution. Bottom right: comparison of the experimental adsorption isotherm with the theoretically modelled isotherm.

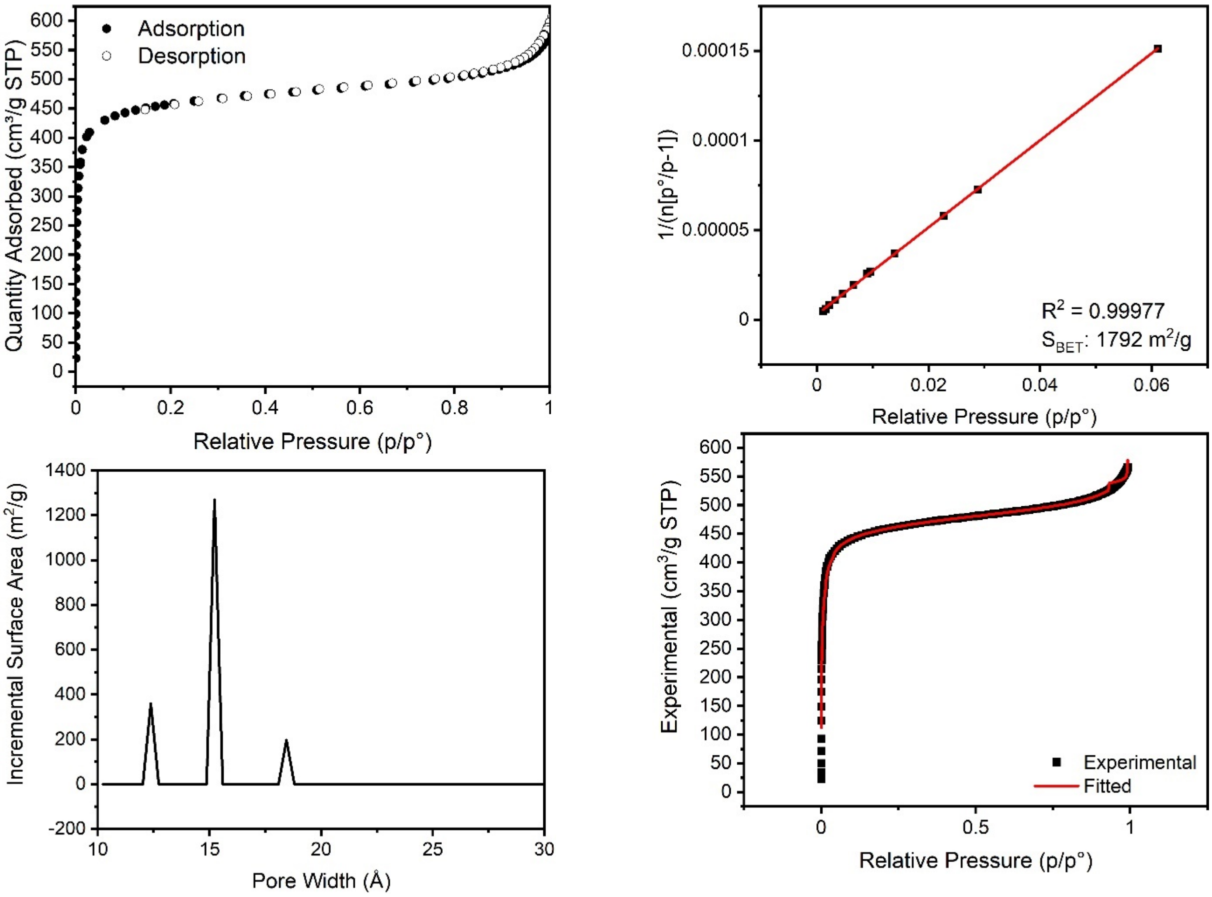

Figure S127: Adsorption measurements for **TpPa** synthesized with 1 equivalent of TEA (duplicate synthesis). Top left: adsorption and desorption isotherm. Top right: linear fit to calculate the BET surface area, including R^2. Bottom left: pore size distribution. Bottom right: comparison of the experimental adsorption isotherm with the theoretically modelled isotherm.

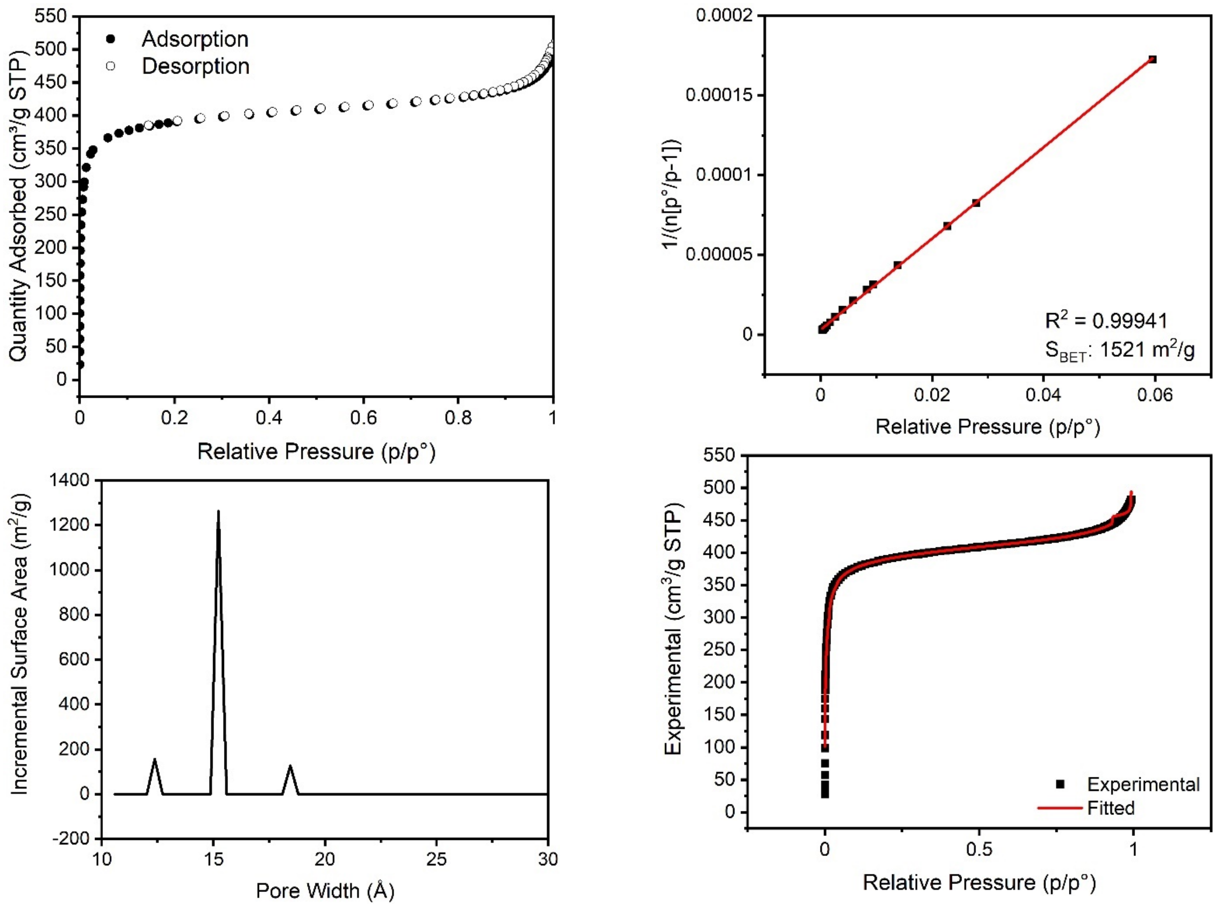

Figure S128: Adsorption measurements for **TpPa** synthesized with 2 equivalents of TEA. Top left: adsorption and desorption isotherm. Top right: linear fit to calculate the BET surface area, including R^2. Bottom left: pore size distribution. Bottom right: comparison of the experimental adsorption isotherm with the theoretically modelled isotherm.

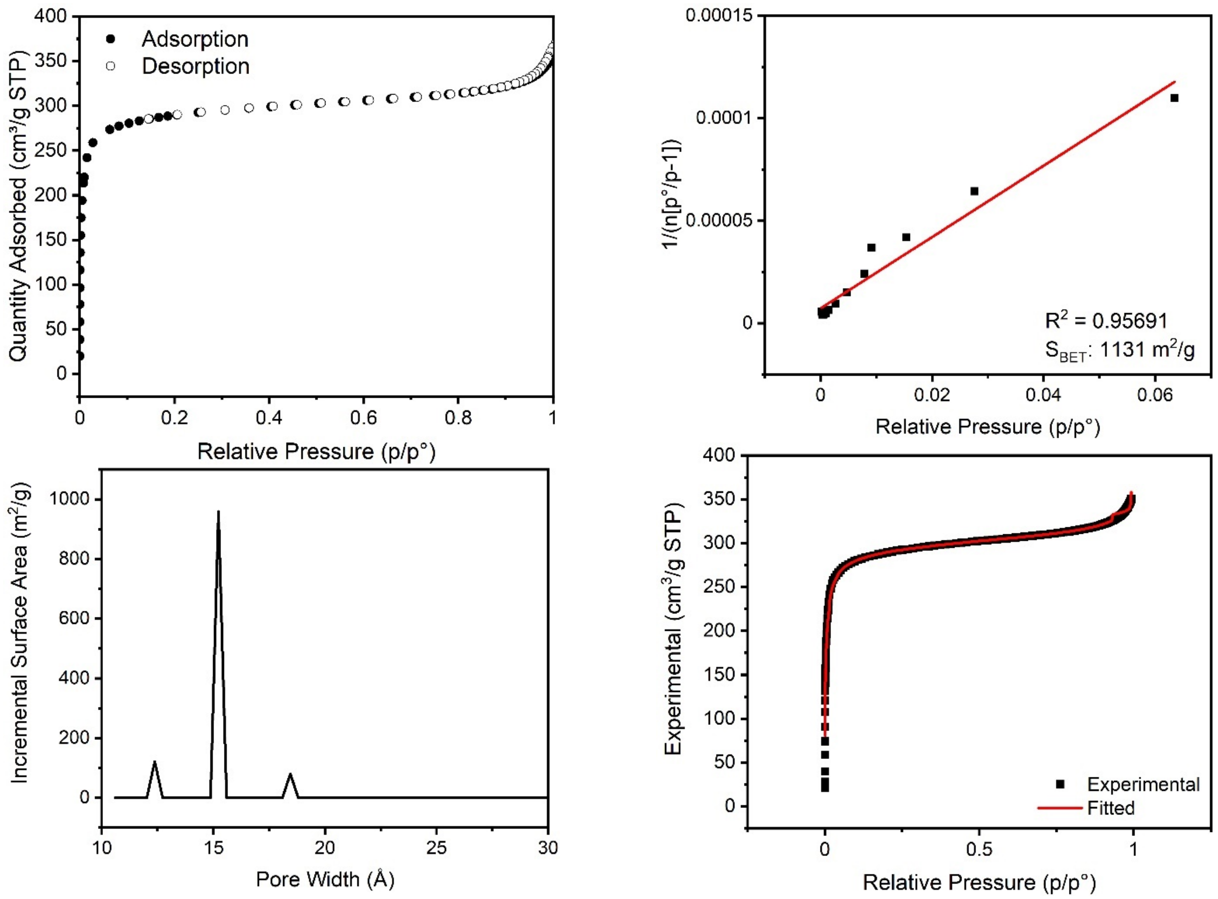

Figure S129: Adsorption measurements for **TpPa** synthesized with 2 equivalents of TEA (duplicate synthesis). Top left: adsorption and desorption isotherm. Top right: linear fit to calculate the BET surface area, including R^2. Bottom left: pore size distribution. Bottom right: comparison of the experimental adsorption isotherm with the theoretically modelled isotherm.

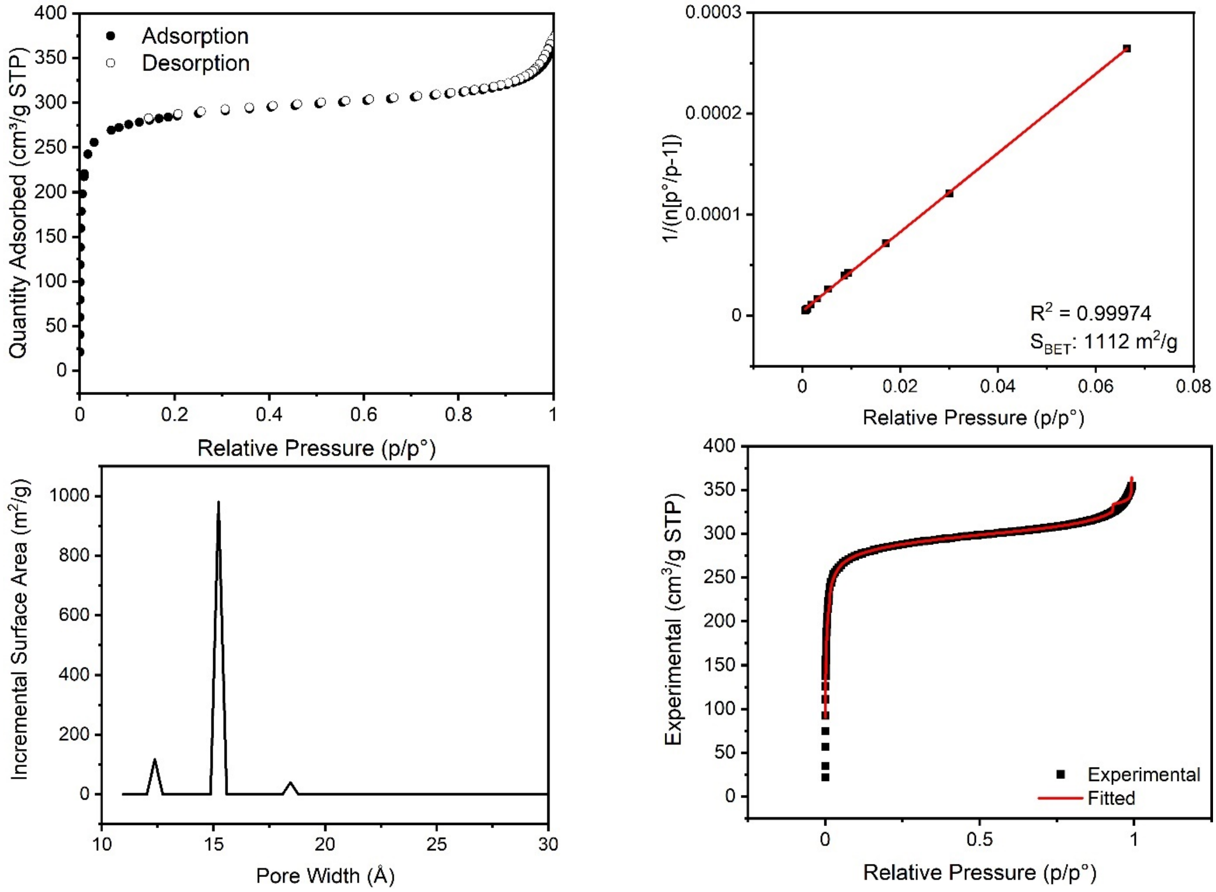

Figure S130: Adsorption measurements for **TpPa** synthesized with 10 equivalents of TEA. Top left: adsorption and desorption isotherm. Top right: linear fit to calculate the BET surface area, including R^2. Bottom left: pore size distribution. Bottom right: comparison of the experimental adsorption isotherm with the theoretically modelled isotherm.

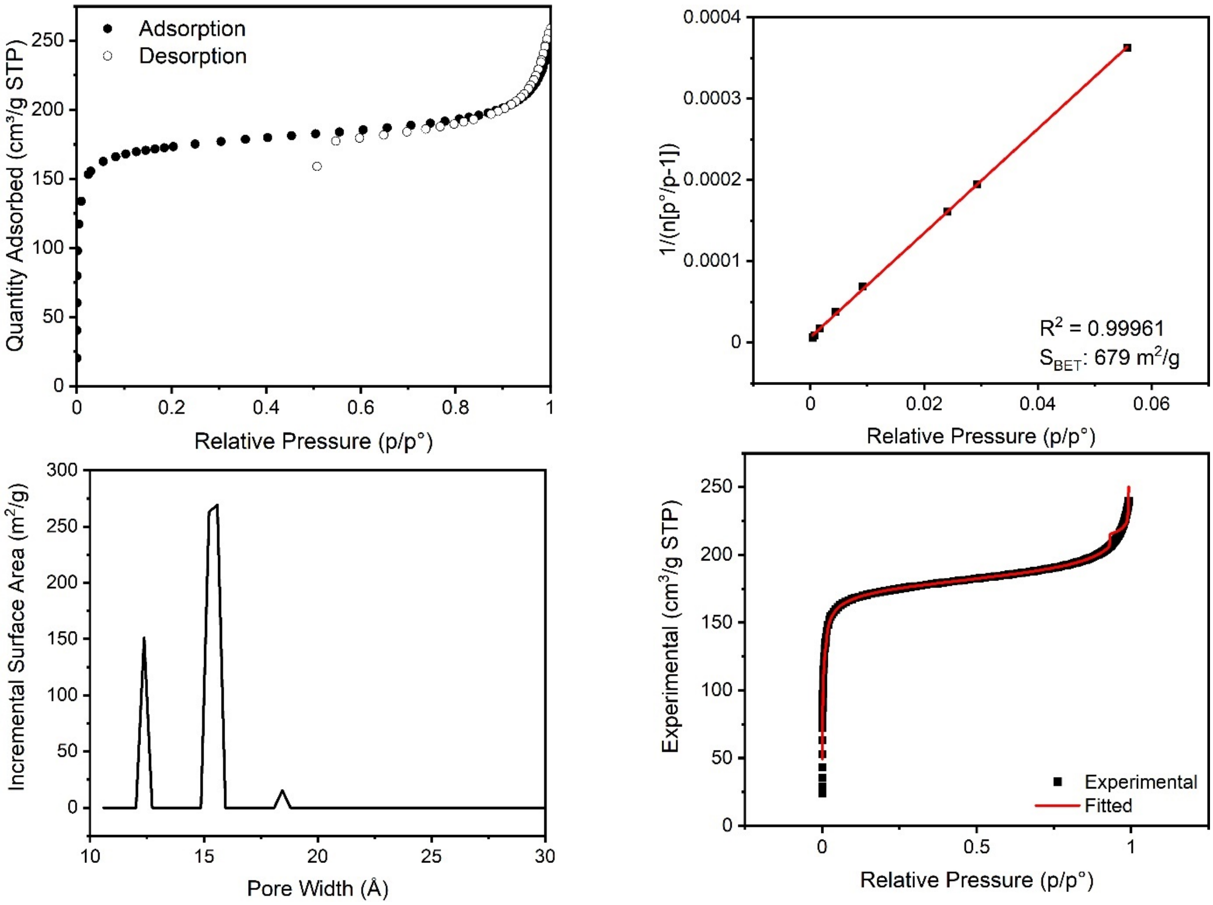

Figure S131: Adsorption measurements for **TpPa** synthesized with 10 equivalents of TEA (duplicate synthesis). Top left: adsorption and desorption isotherm. Top right: linear fit to calculate the BET surface area, including R^2. Bottom left: pore size distribution. Bottom right: comparison of the experimental adsorption isotherm with the theoretically modelled isotherm.

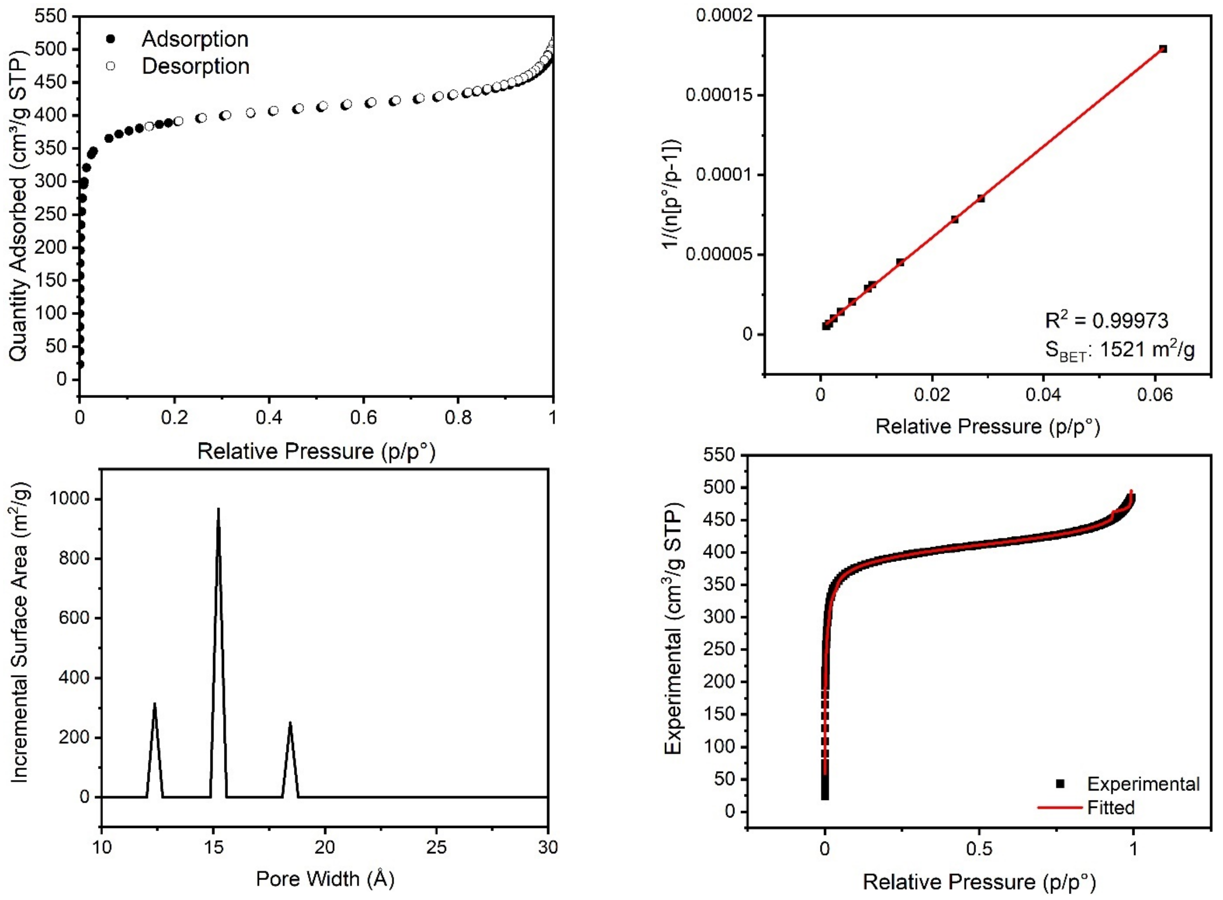

Figure S132: Adsorption measurements for **TpPa** synthesized with 20 equivalents of TEA. Top left: adsorption and desorption isotherm. Top right: linear fit to calculate the BET surface area, including R^2. Bottom left: pore size distribution. Bottom right: comparison of the experimental adsorption isotherm with the theoretically modelled isotherm.

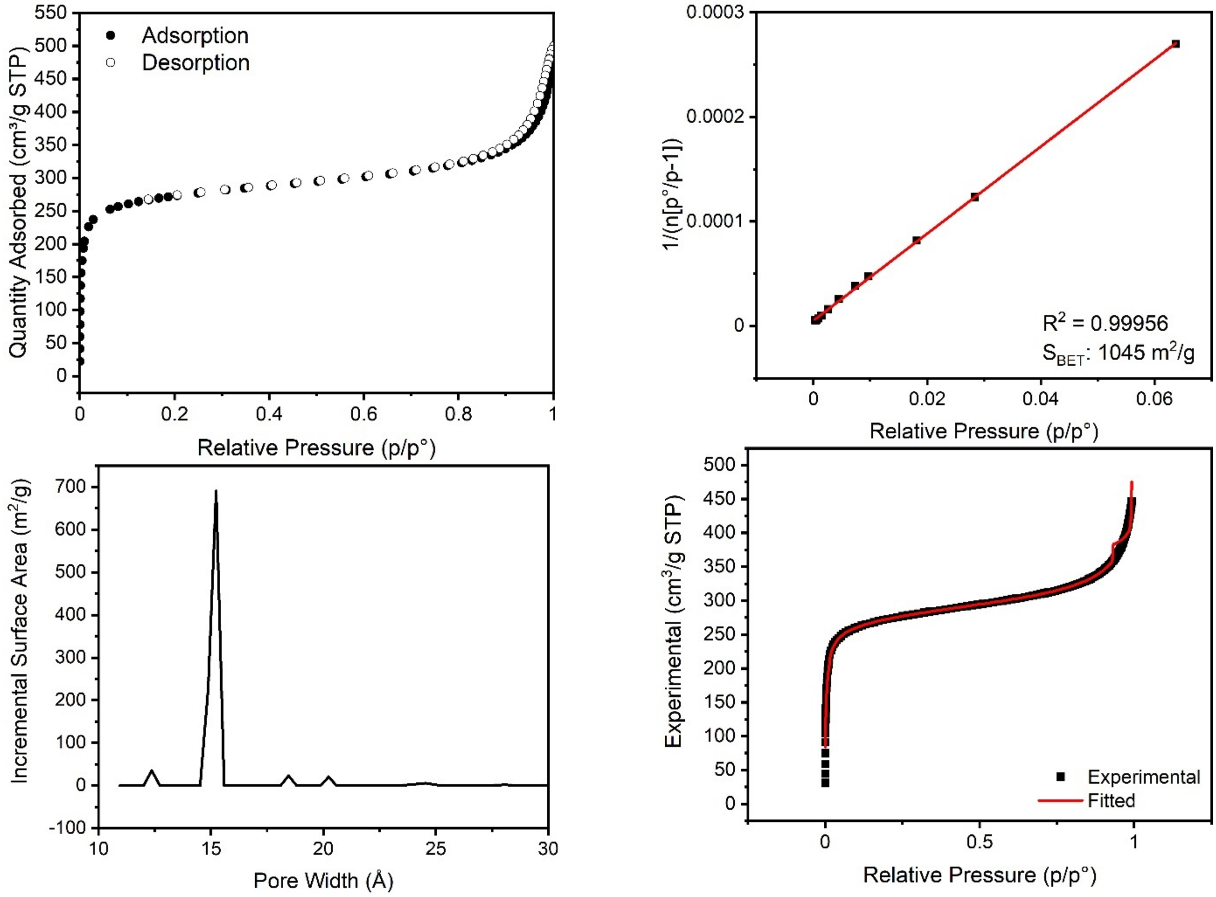

Figure S133: Adsorption measurements for **TpPa** synthesized with 20 equivalents of TEA (duplicate synthesis). Top left: adsorption and desorption isotherm. Top right: linear fit to calculate the BET surface area, including R^2. Bottom left: pore size distribution. Bottom right: comparison of the experimental adsorption isotherm with the theoretically modelled isotherm.

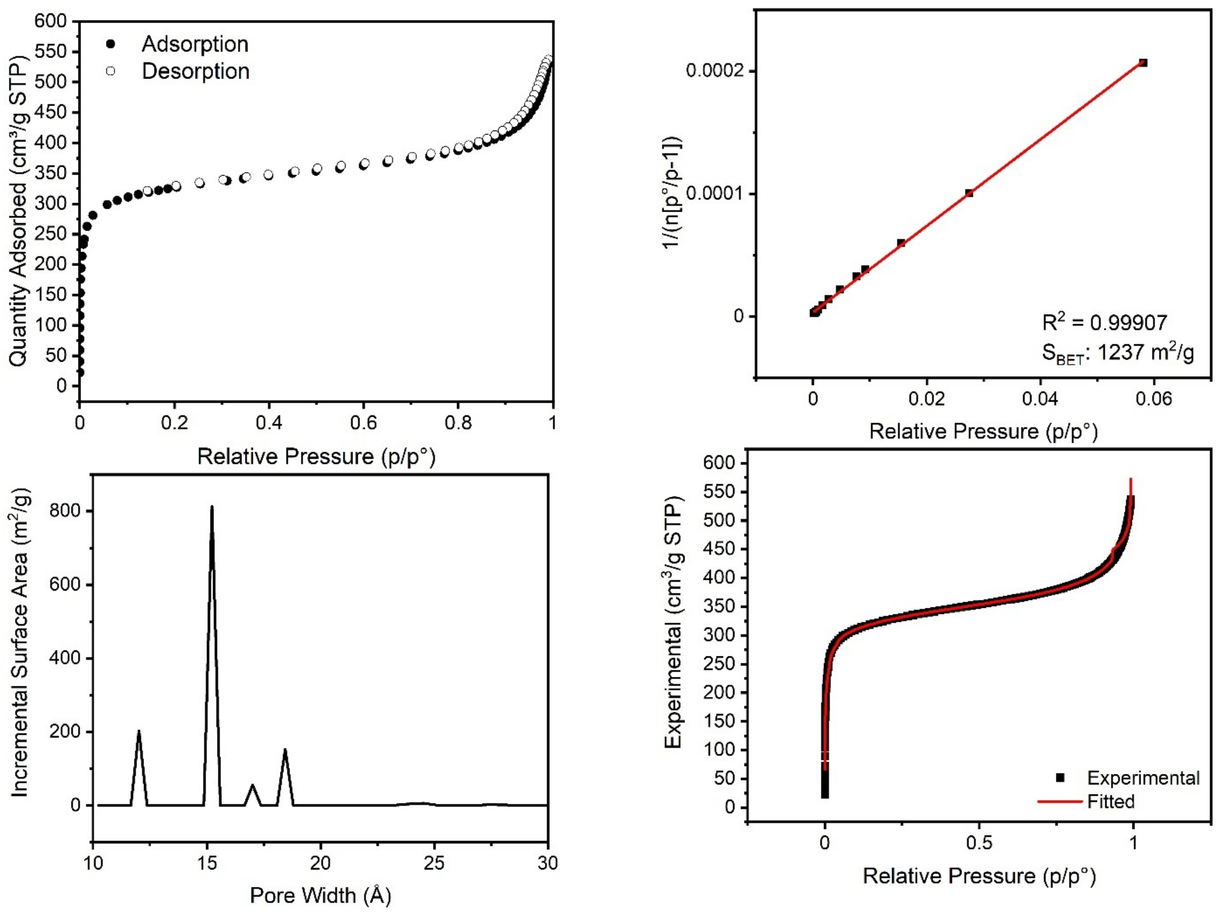

Figure S134: Adsorption measurements for **TpPa** synthesized with 40 equivalents of TEA. Top left: adsorption and desorption isotherm. Top right: linear fit to calculate the BET surface area, including R^2. Bottom left: pore size distribution. Bottom right: comparison of the experimental adsorption isotherm with the theoretically modelled isotherm.

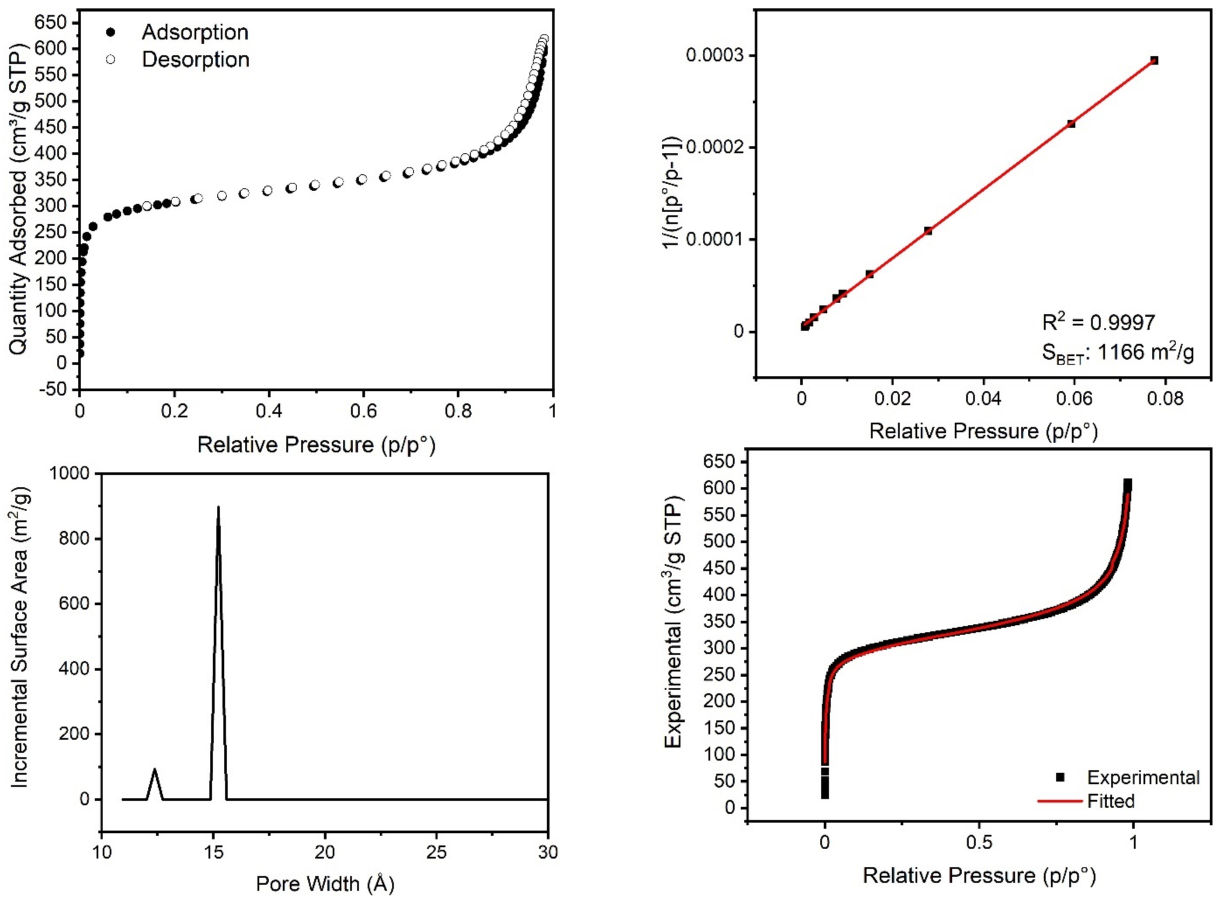

Figure S135: Adsorption measurements for **TpPa** synthesized with 40 equivalents of TEA (duplicate synthesis). Top left: adsorption and desorption isotherm. Top right: linear fit to calculate the BET surface area, including R^2. Bottom left: pore size distribution. Bottom right: comparison of the experimental adsorption isotherm with the theoretically modelled isotherm.

6.9 DIPEA

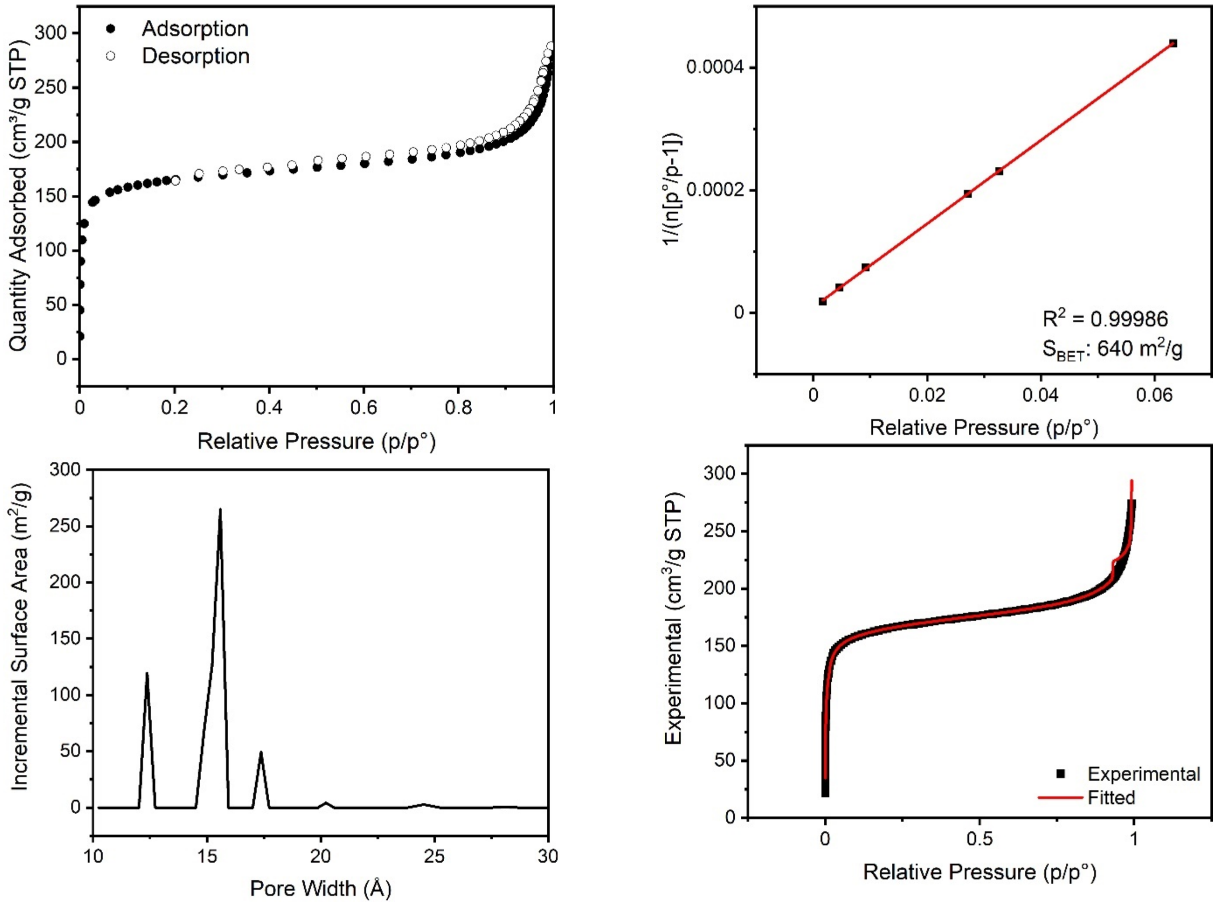

Figure S136: Adsorption measurements for **TpPa** synthesized with ⅓ equivalents of DIPEA. Top left: adsorption and desorption isotherm. Top right: linear fit to calculate the BET surface area, including R^2. Bottom left: pore size distribution. Bottom right: comparison of the experimental adsorption isotherm with the theoretically modelled isotherm.

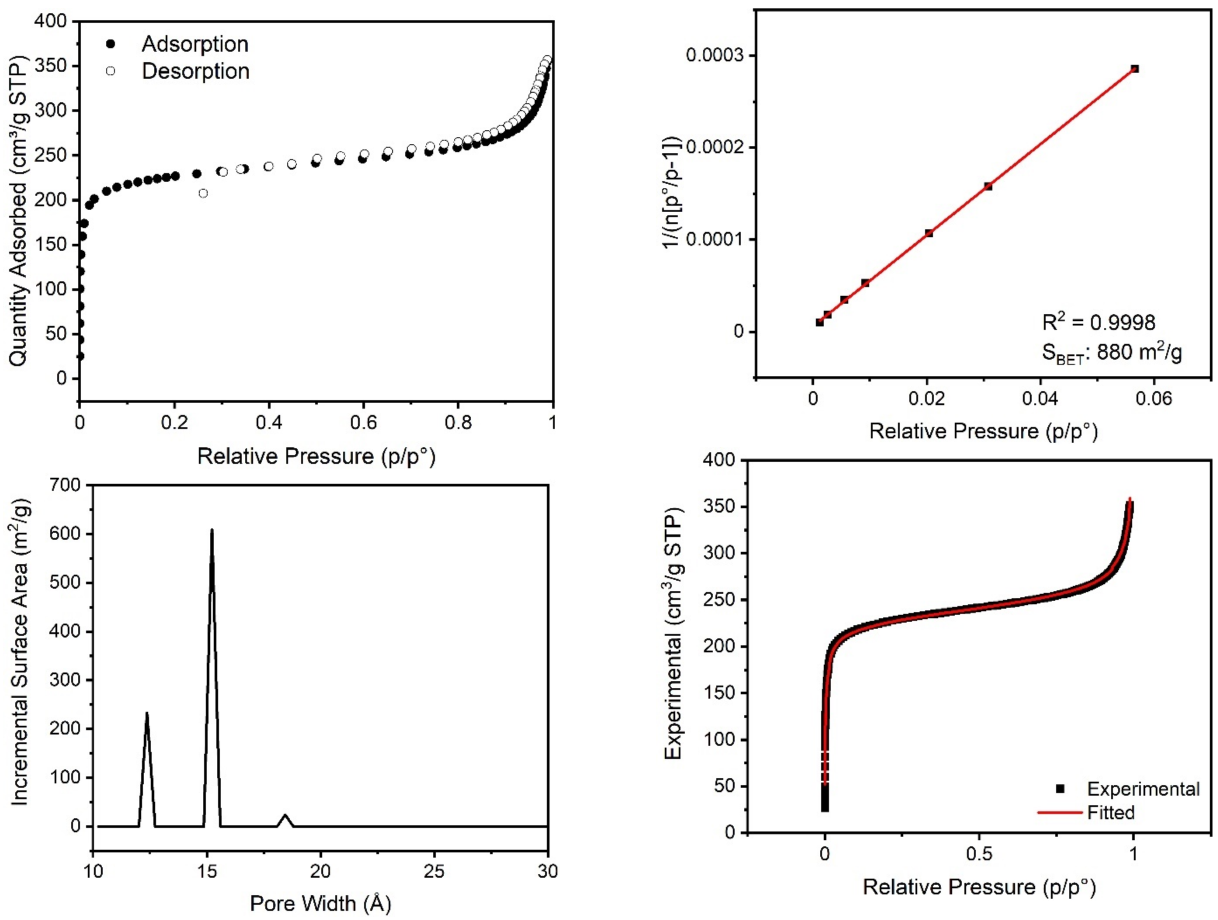

Figure S137: Adsorption measurements for **TpPa** synthesized with ⅓ equivalents of DIPEA (duplicate synthesis). Top left: adsorption and desorption isotherm. Top right: linear fit to calculate the BET surface area, including R^2. Bottom left: pore size distribution. Bottom right: comparison of the experimental adsorption isotherm with the theoretically modelled isotherm.

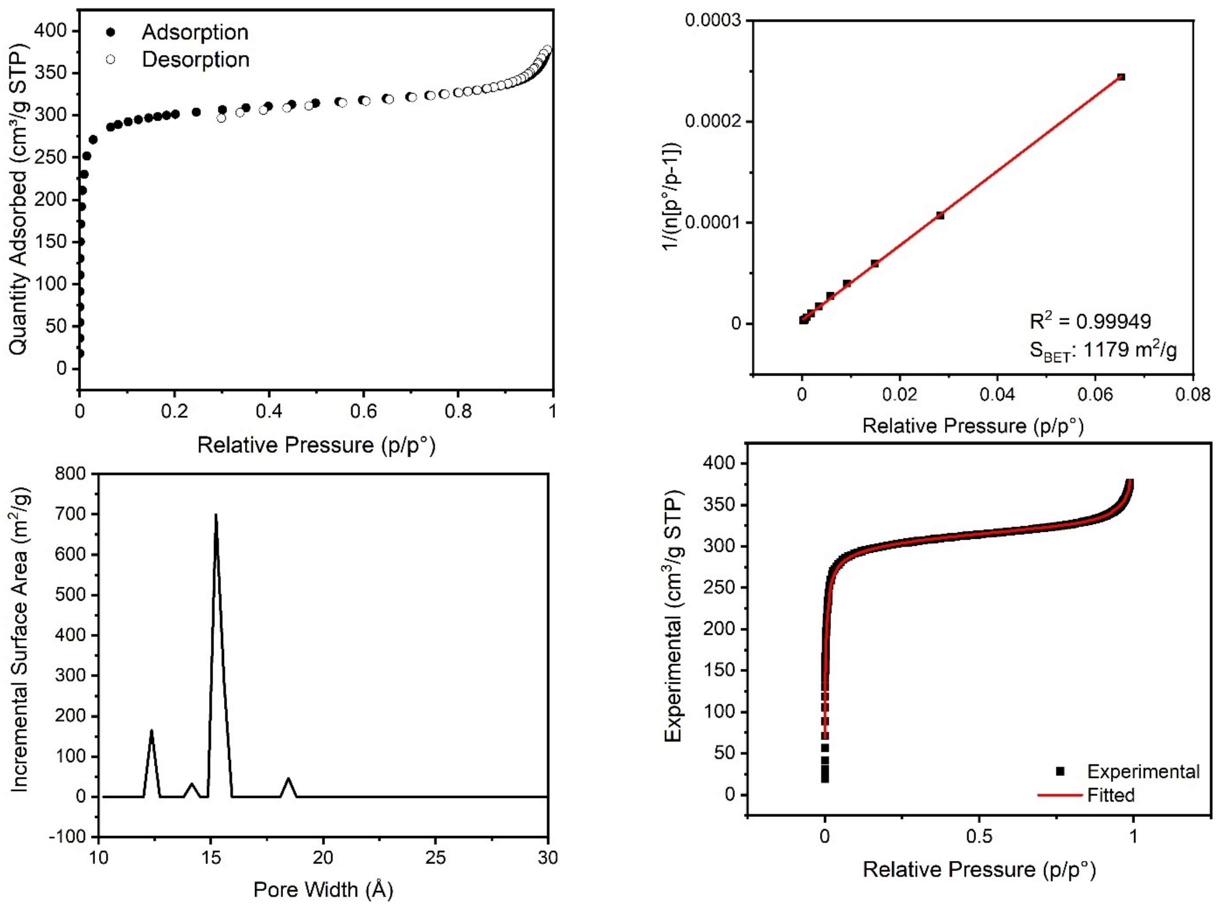

Figure S138: Adsorption measurements for **TpPa** synthesized with 1 equivalent of DIPEA. Top left: adsorption and desorption isotherm. Top right: linear fit to calculate the BET surface area, including R^2. Bottom left: pore size distribution. Bottom right: comparison of the experimental adsorption isotherm with the theoretically modelled isotherm.

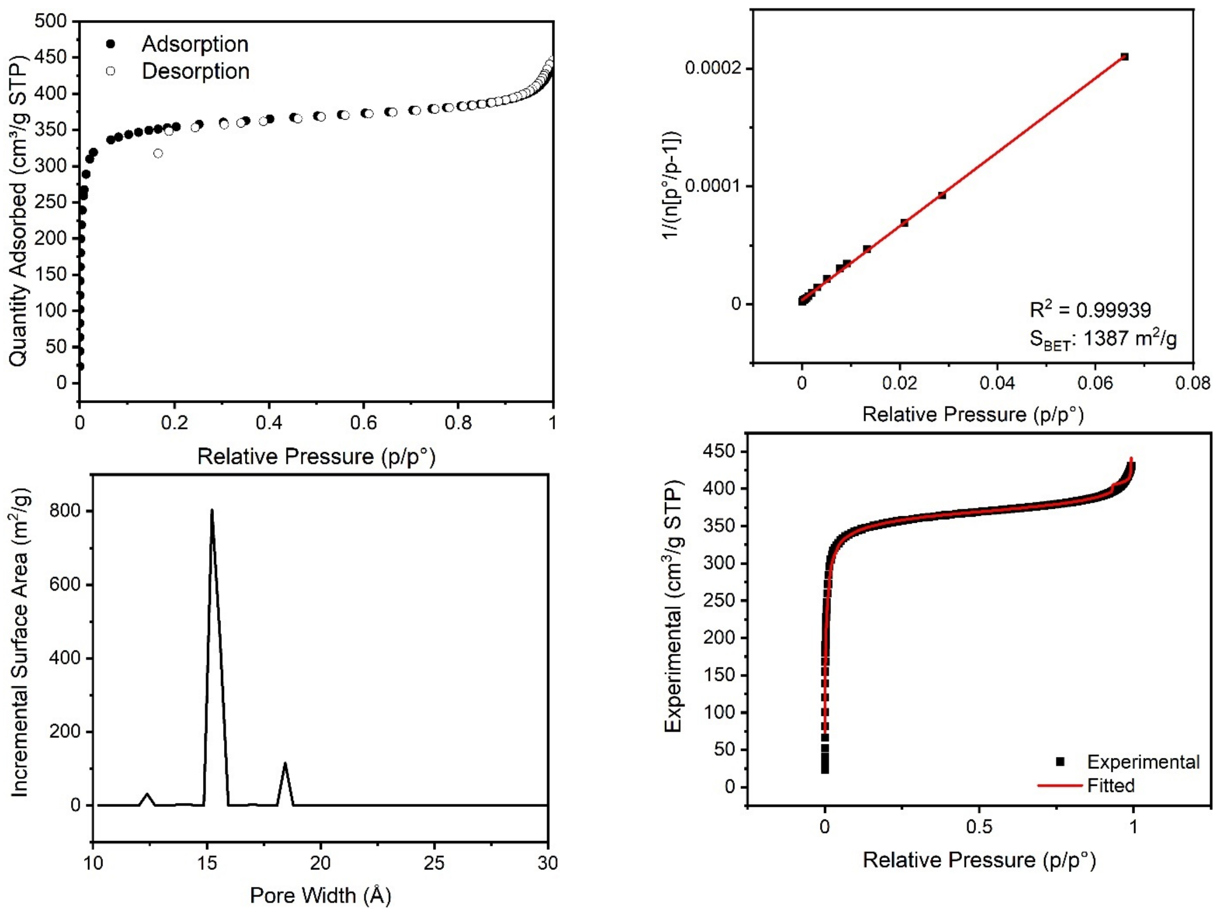

Figure S139: Adsorption measurements for **TpPa** synthesized with 1 equivalent of DIPEA (duplicate synthesis). Top left: adsorption and desorption isotherm. Top right: linear fit to calculate the BET surface area, including R^2. Bottom left: pore size distribution. Bottom right: comparison of the experimental adsorption isotherm with the theoretically modelled isotherm.

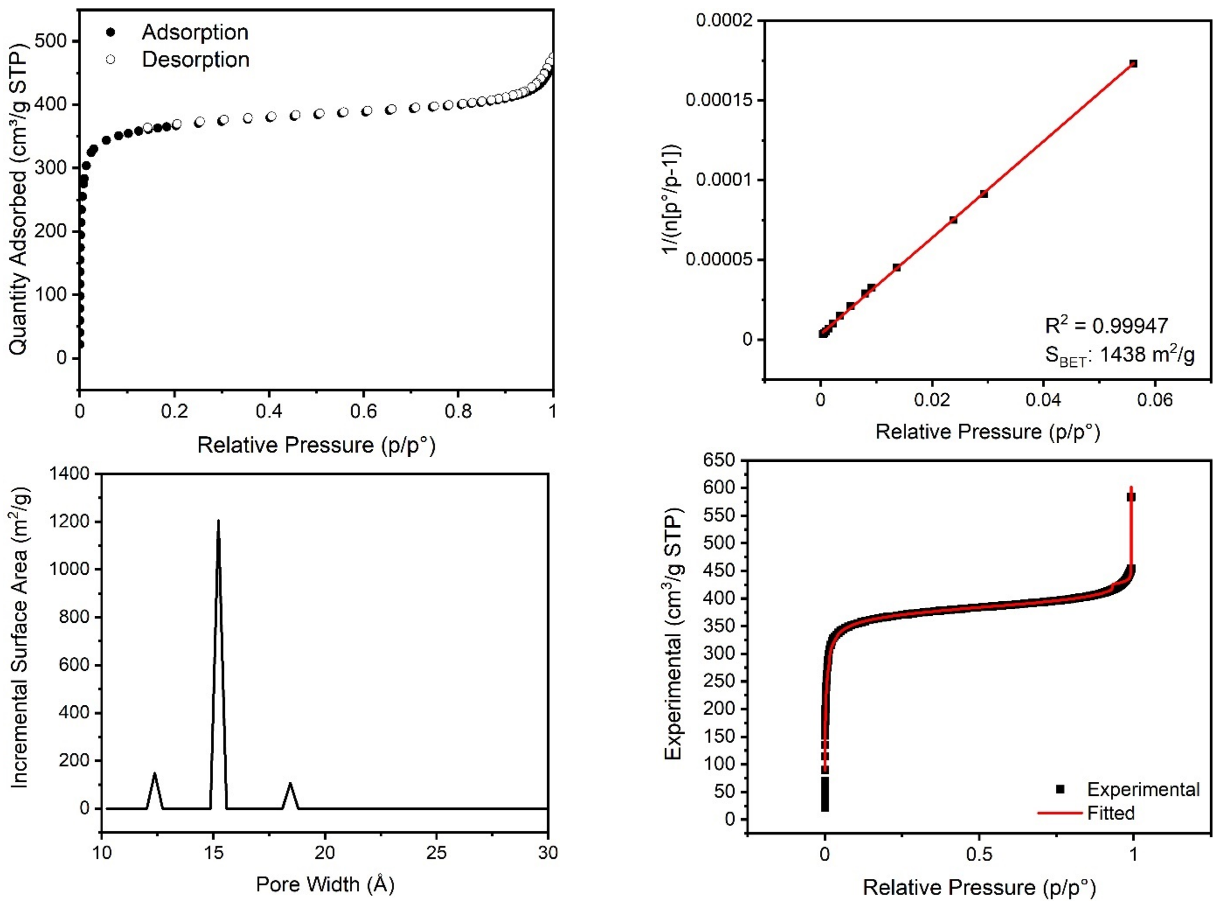

Figure S140: Adsorption measurements for **TpPa** synthesized with 2 equivalents of DIPEA. Top left: adsorption and desorption isotherm. Top right: linear fit to calculate the BET surface area, including R^2. Bottom left: pore size distribution. Bottom right: comparison of the experimental adsorption isotherm with the theoretically modelled isotherm.

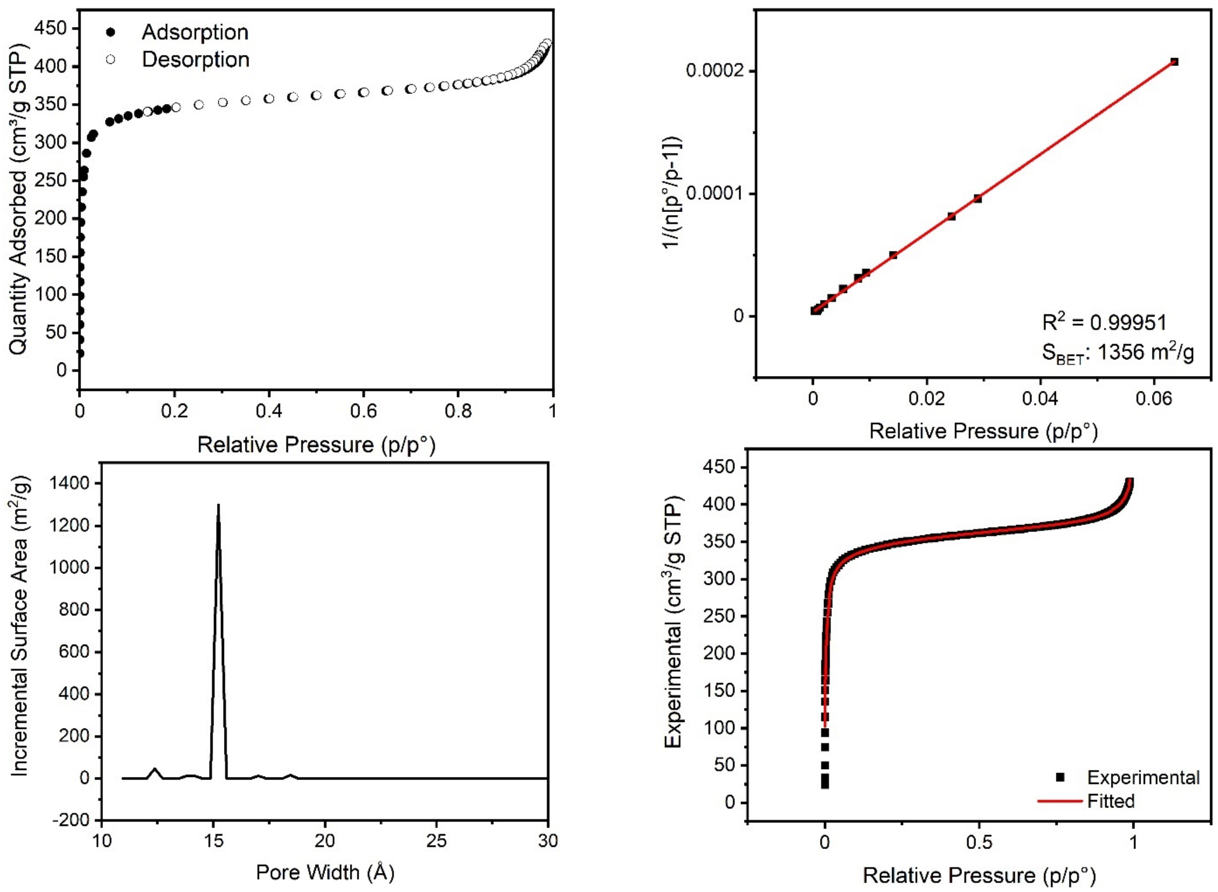

Figure S141: Adsorption measurements for **TpPa** synthesized with 2 equivalents of DIPEA (duplicate synthesis). Top left: adsorption and desorption isotherm. Top right: linear fit to calculate the BET surface area, including R^2. Bottom left: pore size distribution. Bottom right: comparison of the experimental adsorption isotherm with the theoretically modelled isotherm.

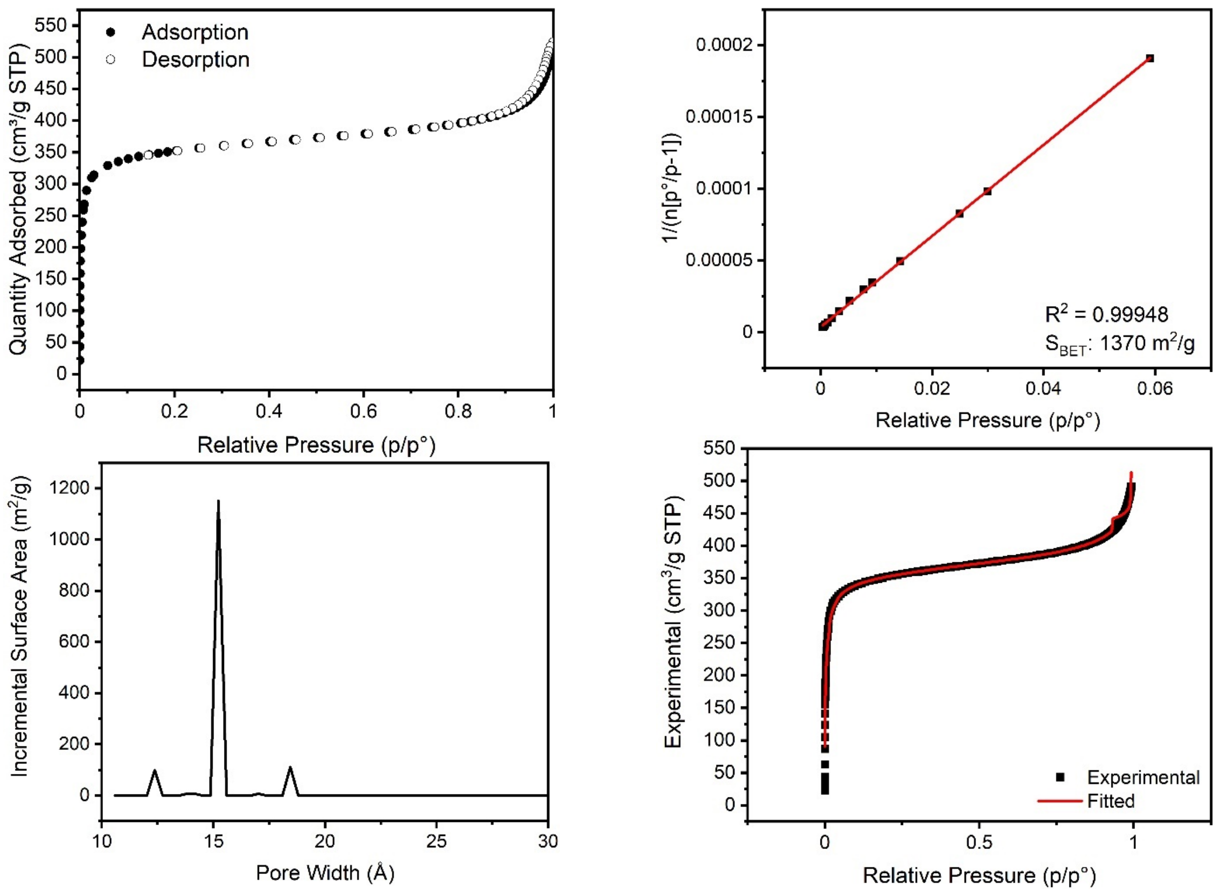

Figure S142: Adsorption measurements for **TpPa** synthesized with 10 equivalents of DIPEA. Top left: adsorption and desorption isotherm. Top right: linear fit to calculate the BET surface area, including R^2. Bottom left: pore size distribution. Bottom right: comparison of the experimental adsorption isotherm with the theoretically modelled isotherm.

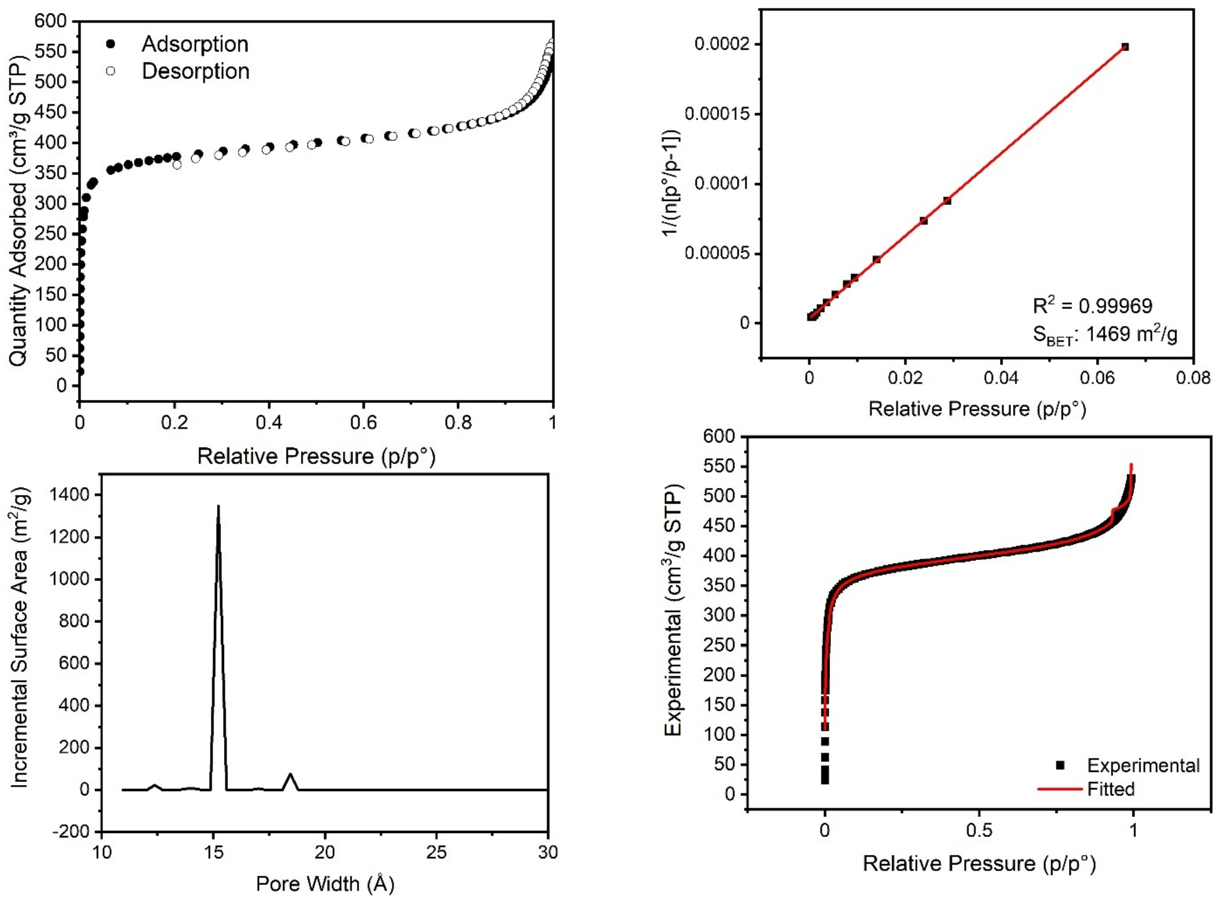

Figure S143: Adsorption measurements for **TpPa** synthesized with 10 equivalents of DIPEA (duplicate synthesis). Top left: adsorption and desorption isotherm. Top right: linear fit to calculate the BET surface area, including R^2. Bottom left: pore size distribution. Bottom right: comparison of the experimental adsorption isotherm with the theoretically modelled isotherm.

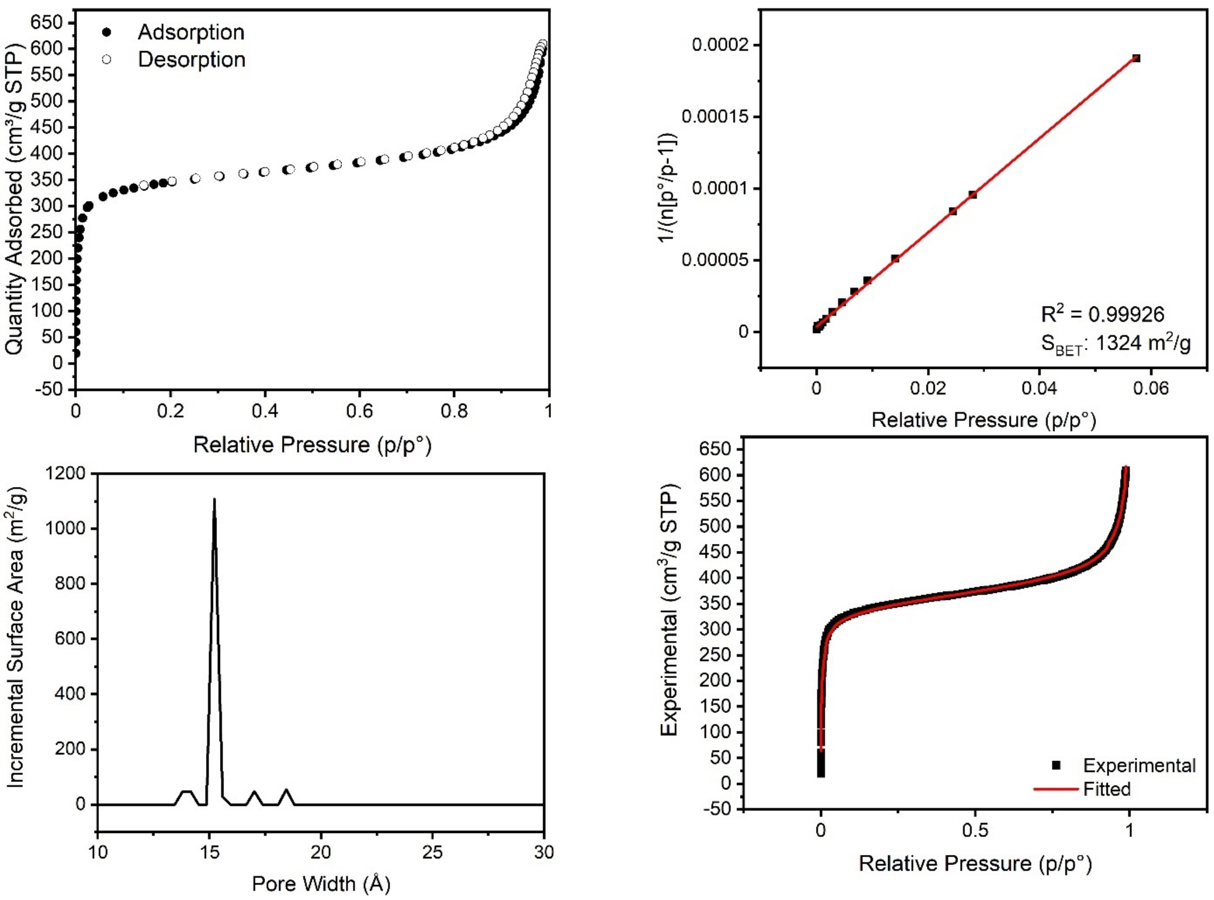

Figure S144: Adsorption measurements for **TpPa** synthesized with 20 equivalents of DIPEA. Top left: adsorption and desorption isotherm. Top right: linear fit to calculate the BET surface area, including R^2. Bottom left: pore size distribution. Bottom right: comparison of the experimental adsorption isotherm with the theoretically modelled isotherm.

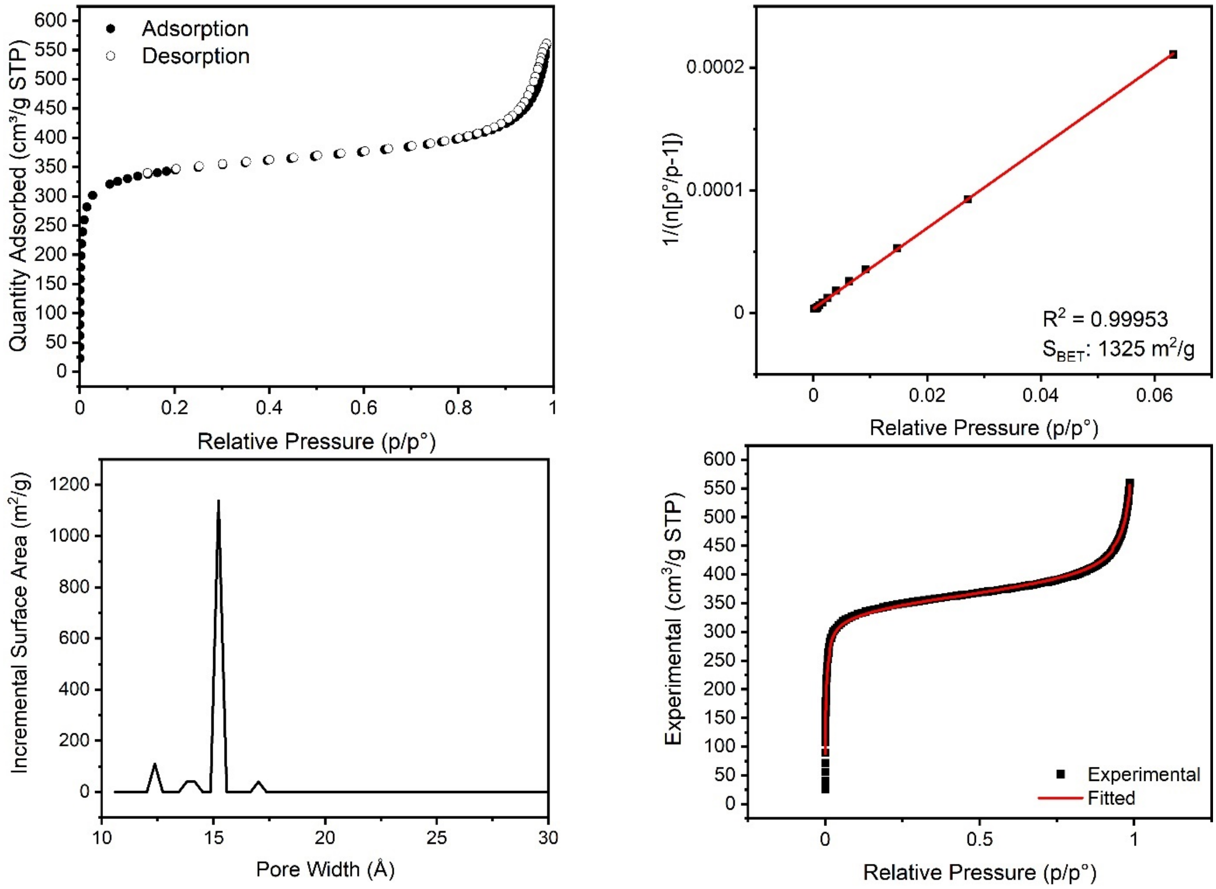

Figure S145: Adsorption measurements for **TpPa** synthesized with 20 equivalents of DIPEA (duplicate synthesis). Top left: adsorption and desorption isotherm. Top right: linear fit to calculate the BET surface area, including R^2. Bottom left: pore size distribution. Bottom right: comparison of the experimental adsorption isotherm with the theoretically modelled isotherm.

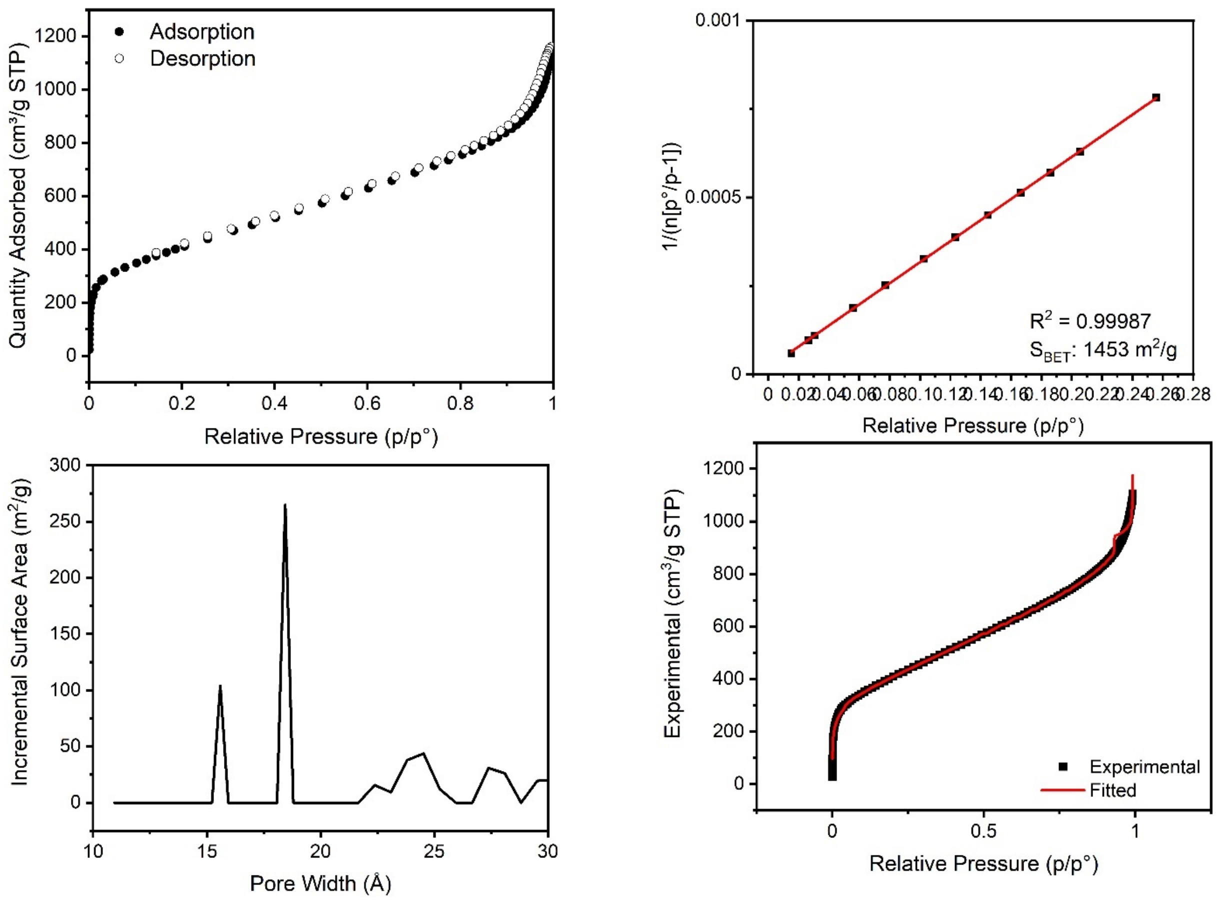

Figure S146: Adsorption measurements for **TpPa** synthesized with 40 equivalents of DIPEA. Top left: adsorption and desorption isotherm. Top right: linear fit to calculate the BET surface area, including R^2. Bottom left: pore size distribution. Bottom right: comparison of the experimental adsorption isotherm with the theoretically modelled isotherm.

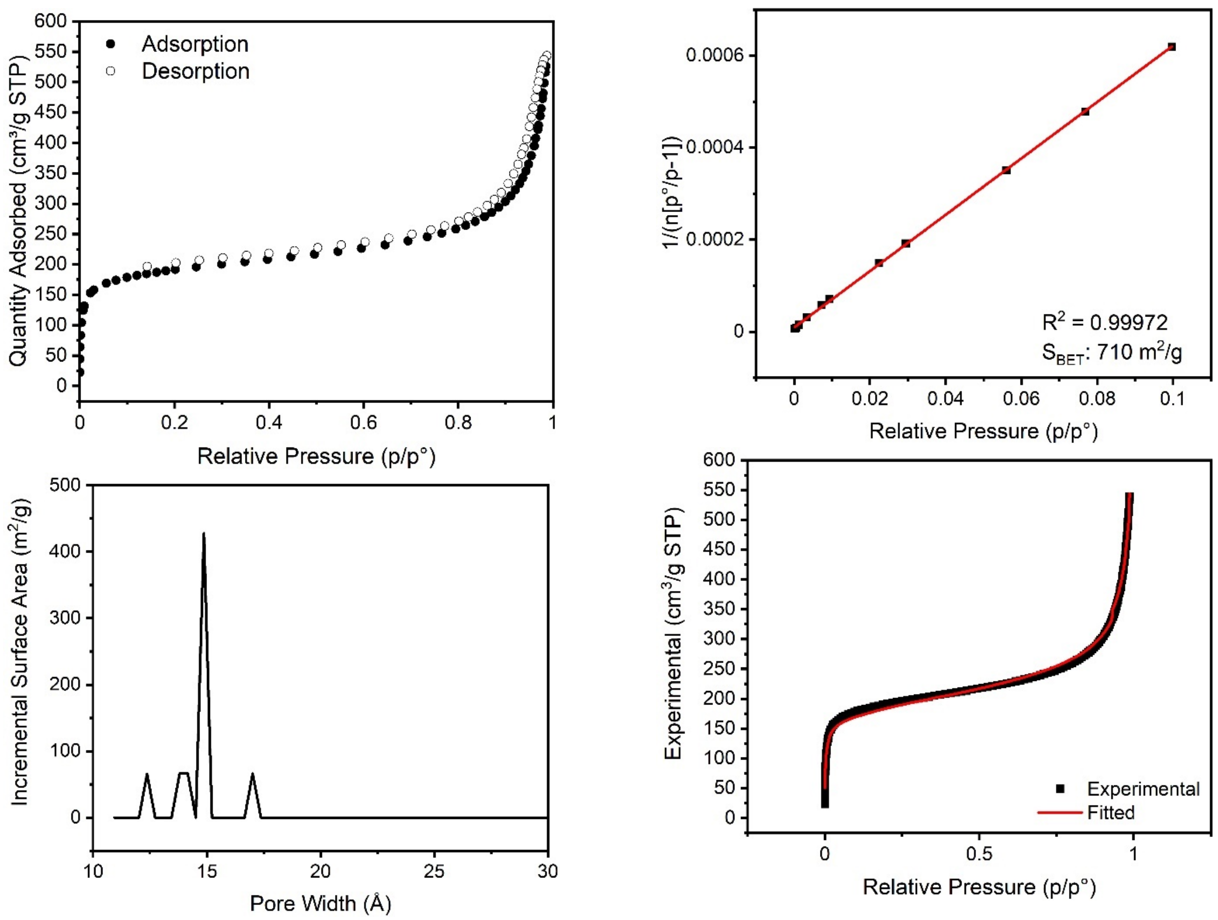

Figure S147: Adsorption measurements for **TpPa** synthesized with 40 equivalents of DIPEA (duplicate synthesis). Top left: adsorption and desorption isotherm. Top right: linear fit to calculate the BET surface area, including R^2. Bottom left: pore size distribution. Bottom right: comparison of the experimental adsorption isotherm with the theoretically modelled isotherm.

6.10 DBU

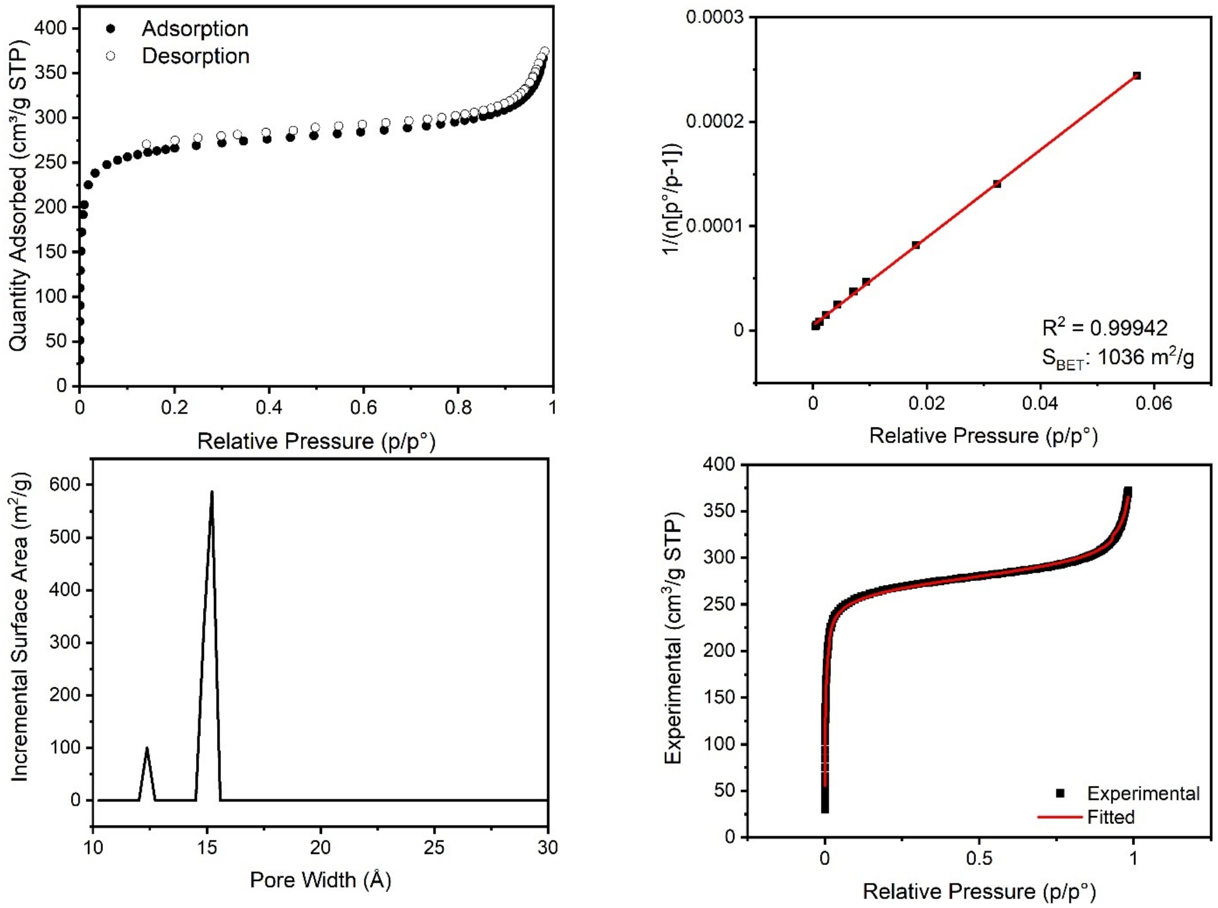

Figure S148: Adsorption measurements for **TpPa** synthesized with ⅓ equivalents of DBU. Top left: adsorption and desorption isotherm. Top right: linear fit to calculate the BET surface area, including R^2. Bottom left: pore size distribution. Bottom right: comparison of the experimental adsorption isotherm with the theoretically modelled isotherm.

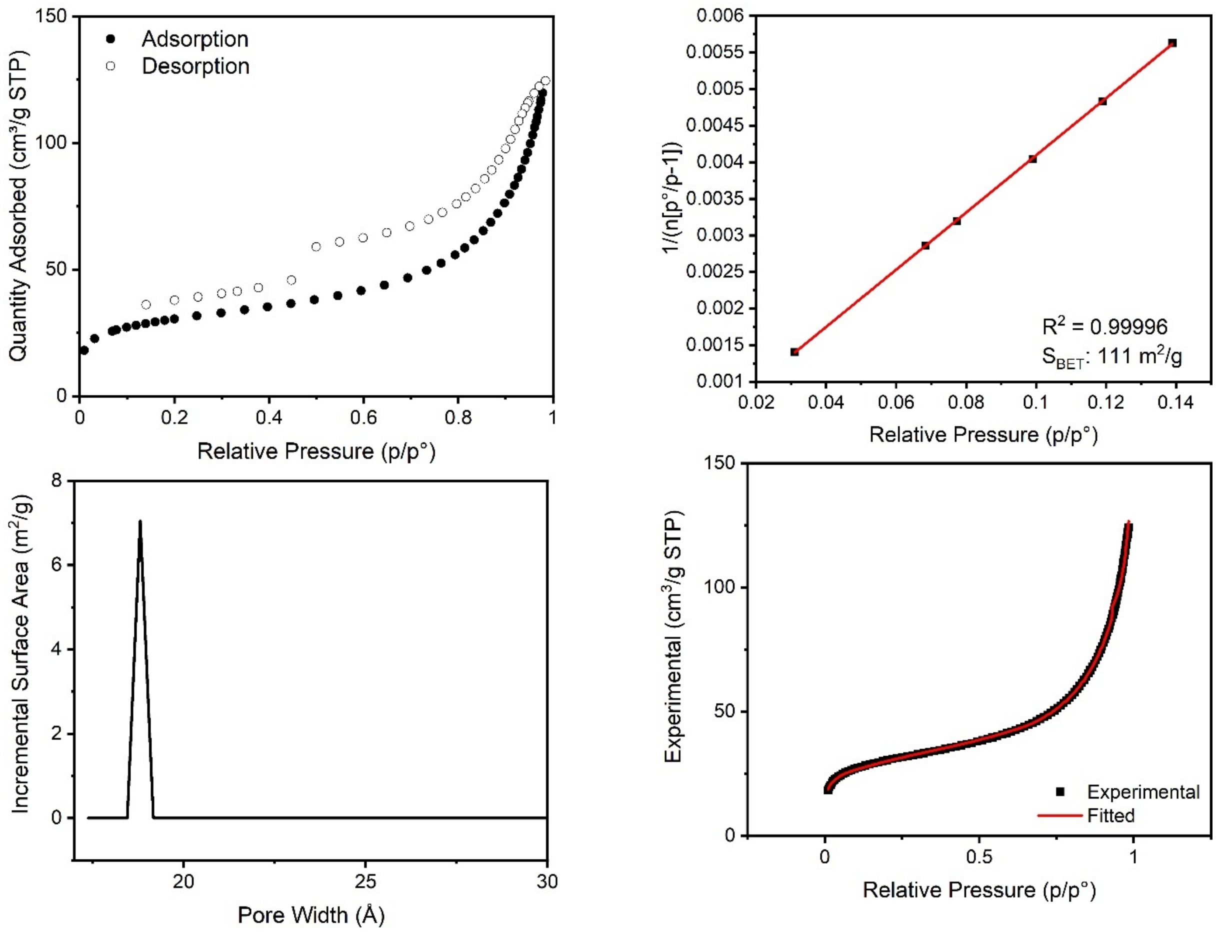

Figure S149: Adsorption measurements for **TpPa** synthesized with 2 equivalents of DBU. Top left: adsorption and desorption isotherm. Top right: linear fit to calculate the BET surface area, including R^2. Bottom left: pore size distribution. Bottom right: comparison of the experimental adsorption isotherm with the theoretically modelled isotherm.

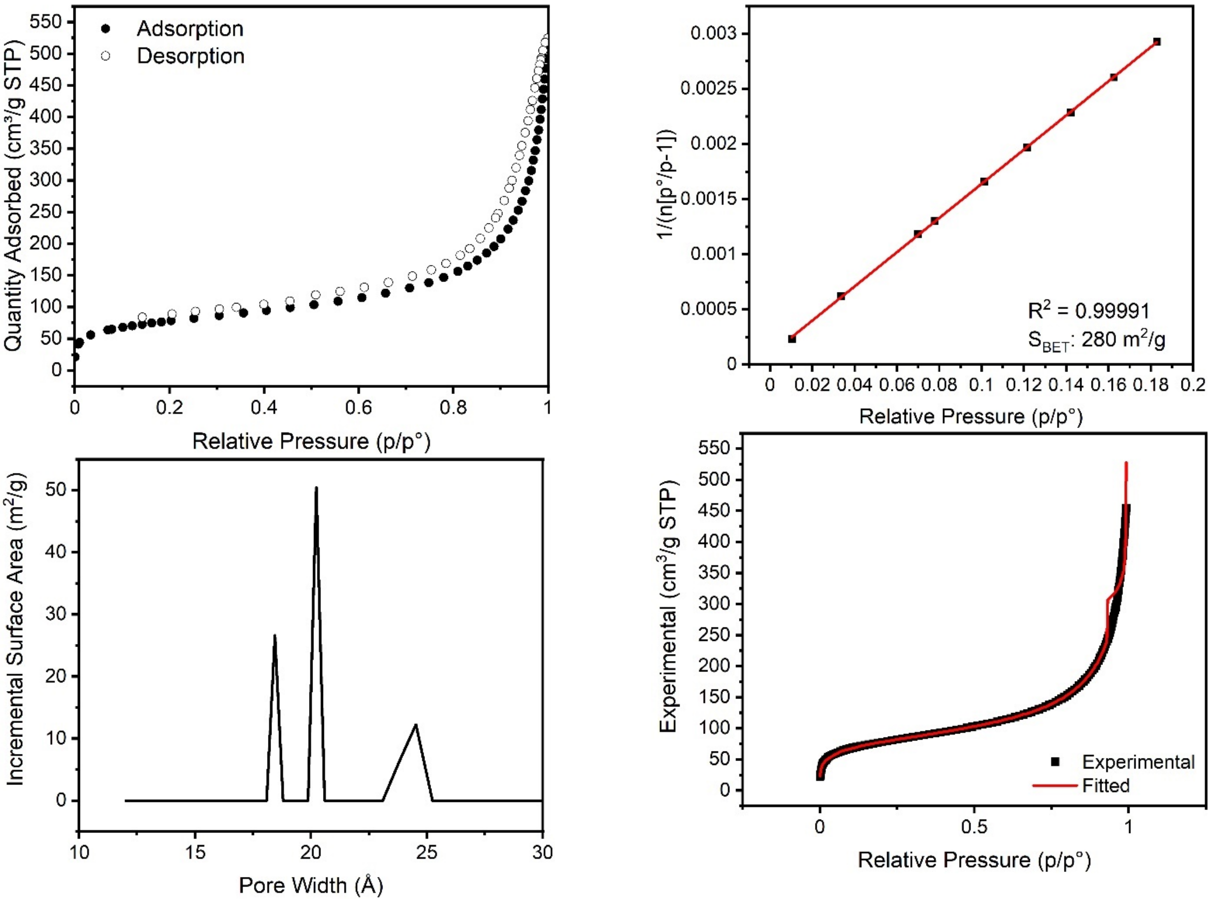

Figure S150: Adsorption measurements for **TpPa** synthesized with 2 equivalents of DBU (duplicate synthesis). Top left: adsorption and desorption isotherm. Top right: linear fit to calculate the BET surface area, including R^2. Bottom left: pore size distribution. Bottom right: comparison of the experimental adsorption isotherm with the theoretically modelled isotherm.

6.11 KOH

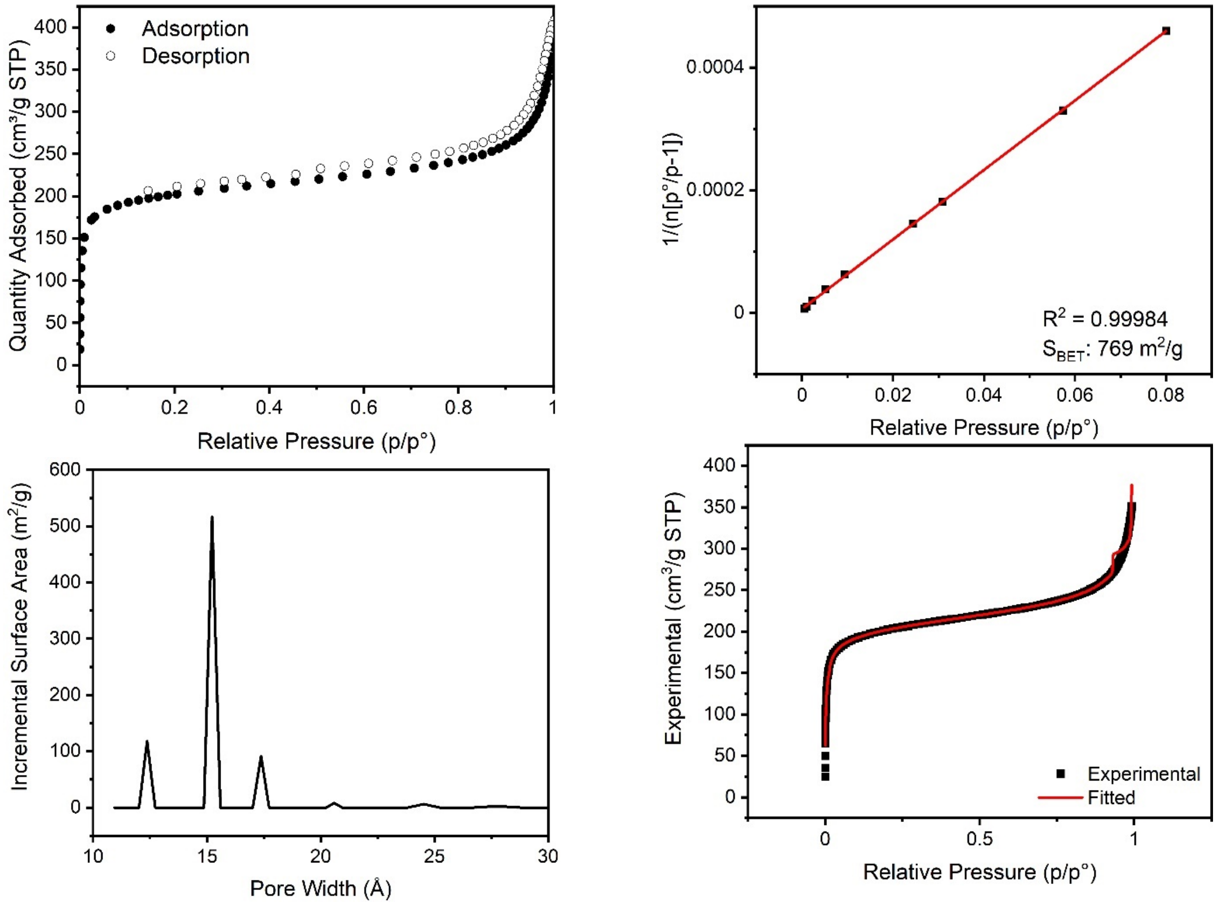

Figure S151: Adsorption measurements for **TpPa** synthesized with ⅓ equivalents of KOH. Top left: adsorption and desorption isotherm. Top right: linear fit to calculate the BET surface area, including R^2. Bottom left: pore size distribution. Bottom right: comparison of the experimental adsorption isotherm with the theoretically modelled isotherm.

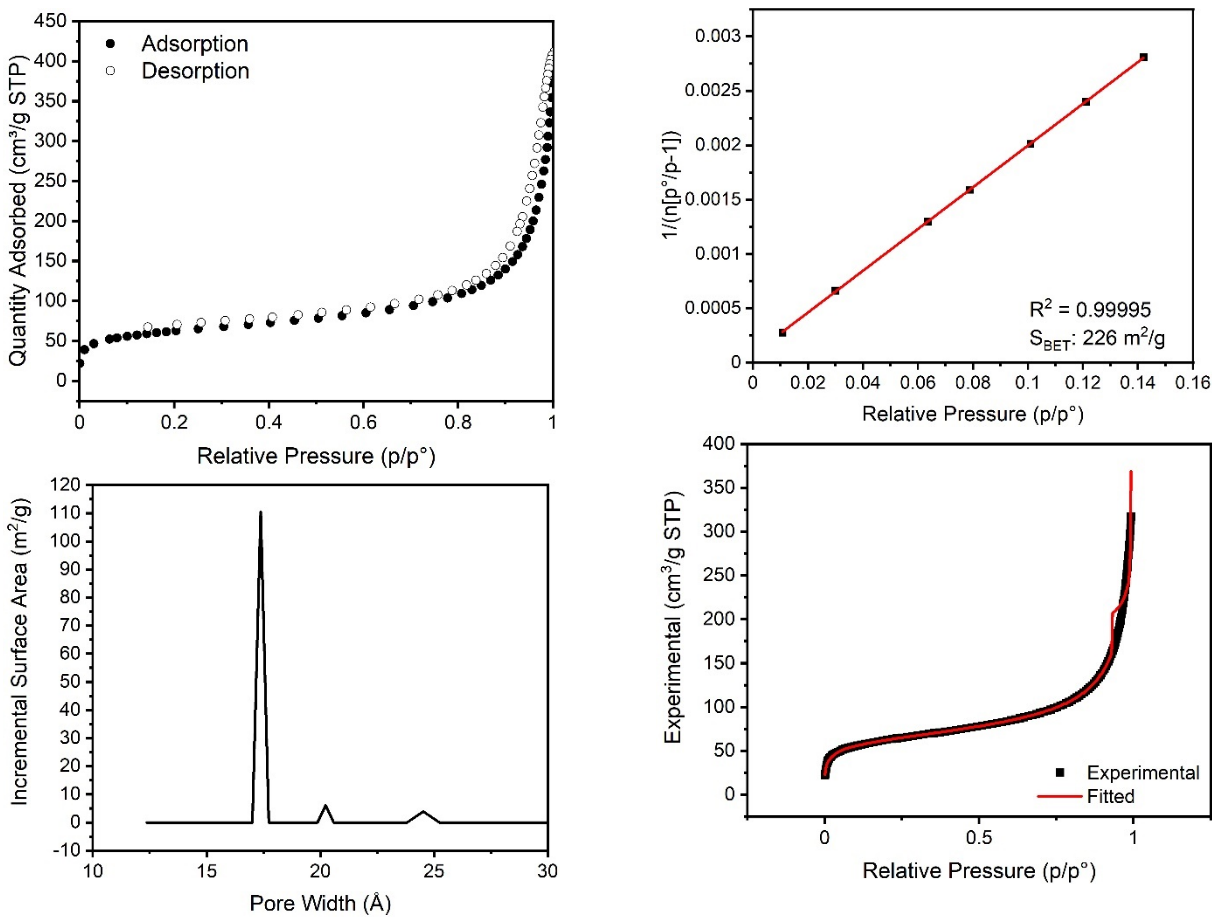

Figure S152: Adsorption measurements for **TpPa** synthesized with 2 equivalents of KOH. Top left: adsorption and desorption isotherm. Top right: linear fit to calculate the BET surface area, including R^2. Bottom left: pore size distribution. Bottom right: comparison of the experimental adsorption isotherm with the theoretically modelled isotherm.

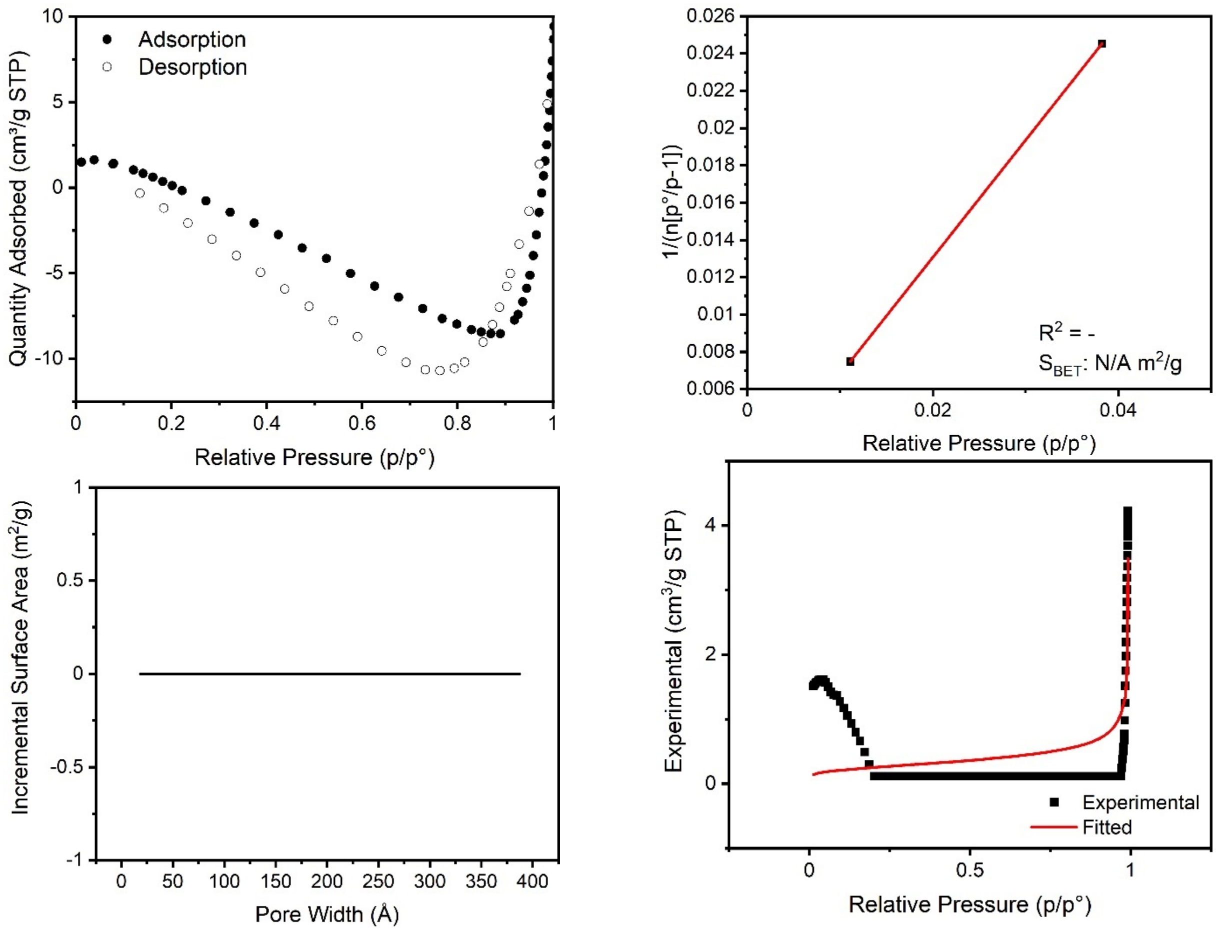

Figure S153: Adsorption measurements for **TpPa** synthesized with 2 equivalents of KOH. Top left: adsorption and desorption isotherm. Top right: linear fit to calculate the BET surface area, including R^2. Bottom left: pore size distribution. Bottom right: comparison of the experimental adsorption isotherm with the theoretically modelled isotherm. This material is no longer porous.

7. SEM

For selected frameworks, their morphology was investigated by scanning electrode microscopy. A representative image for each sample is displayed below.

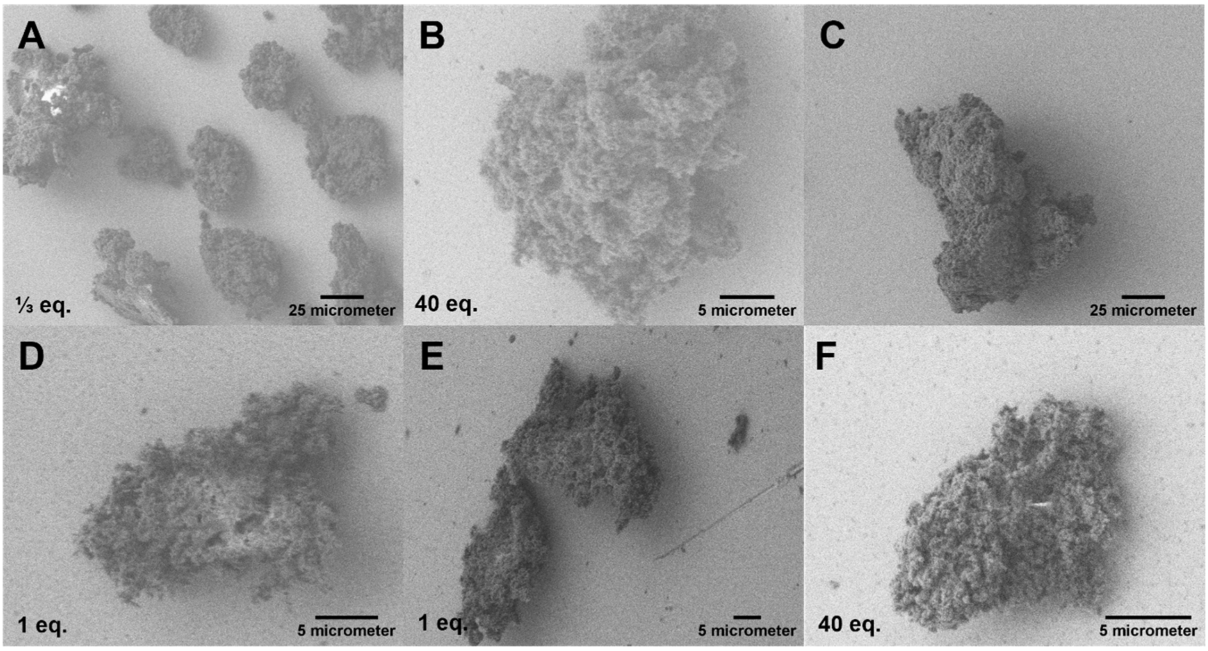

Figure S154: SEM images of **TpPa** synthesized with acetic acid (A-B), without acid or base (C) or TEA (D-F).

8. Titration of **Tp**

For the titration, 250 mg (1.19 mmol) of **Tp** was dissolved in 50.0 mL of water with an excess of NaOH (190.3 mg (4.76 mmol); 4 equivalents, so 1 equivalent excess per functional group). It was then titrated with an aqueous 0.19 M HCl solution (Figure S155). At first, the HCl reacted with the excess NaOH. Then, it reacted with the most basic phenolate (grey area), with an average pH of 10.8. After the drop in pH, **Tp** became insoluble below pH 7.7, which can be explained by the second phenolate becoming protonated. Beyond this point, the experiment was paused until the pH stabilized again, which resulted (after roughly an hour) in a pH value of 8.4. The stirred suspension still reacted with the titrated HCl, though at a much slower pace: 10-30 minutes per step was needed to allow stabilization of the pH. The entire titration curve was thus measured, revealing that the final (third) phenolate is protonated at a pH of 4.0.

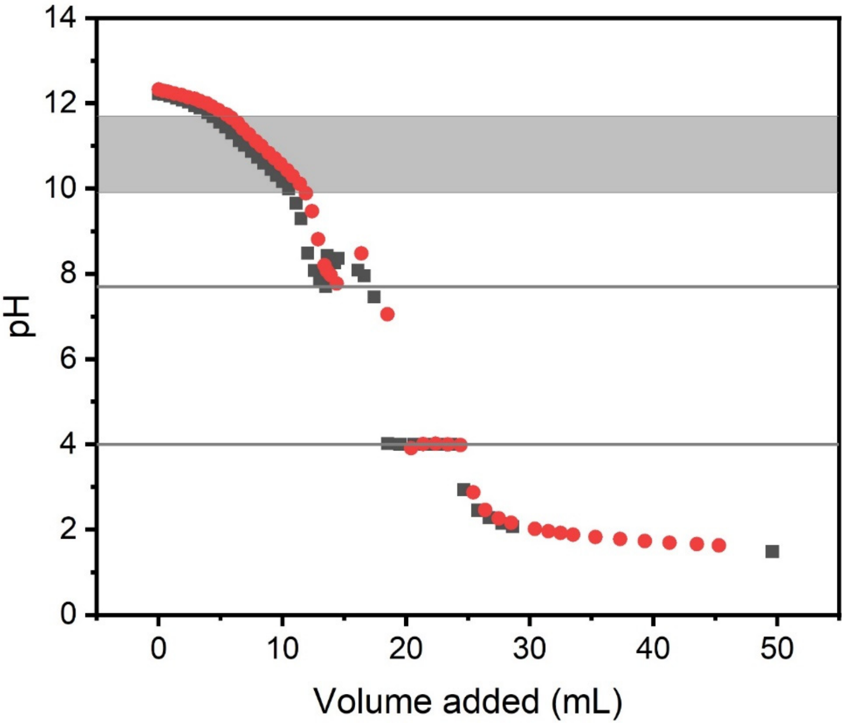

Figure S155: Titration curve of **Tp** (black) and a duplicate measurement (red).

9. References

[S1] E. Dautzenberg, M. Lam, G. Li, L. C. P. M. De Smet, *Nanoscale* **2021**, *13*, 19446-19452.

[S2] S. Kandambeth, A. Mallick, B. Lukose, M. V. Mane, T. Heine, R. Banerjee, *J. Am. Chem. Soc.* **2012**, *134*, 19524-19527.
